# Anthoteibinenes F–Q: New Sesquiterpenes from the Irish Deep-Sea Coral *Anthothela grandiflora*

**DOI:** 10.3390/md23010044

**Published:** 2025-01-17

**Authors:** Stine S. H. Olsen, Sam Afoullouss, Ezequiel Cruz Rosa, Ryan M. Young, Mark Johnson, A. Louise Allcock, Bill. J. Baker

**Affiliations:** 1 Department of Chemistry, University of South Florida, 4202 E. Fowler Avenue, CHE205, Tampa, FL 33620, USA; olsens@usf.edu (S.S.H.O.); samafoullouss@usf.edu (S.A.); ecruzrosa@usf.edu (E.C.R.); 2 School of Natural Sciences and Ryan Institute, University of Galway, University Road, H91TK33 Galway, Ireland; ryan.mark.young@gmail.com (R.M.Y.); mark.johnson@nuigalway.ie (M.J.); louise.allcock@nuigalway.ie (A.L.A.)

**Keywords:** deep-sea, coral, sesquiterpene, cadinene, *Anthothela*, *Candida*

## Abstract

New technology has opened opportunities for research and exploration of deep-water ecosystems, highlighting deep-sea coral reefs as a rich source of novel bioactive natural products. During our ongoing investigation of the chemodiversity of the Irish deep sea and the soft coral *Anthothela grandiflora,* we report 12 unreported cadinene-like functionalized sesquiterpenes, anthoteibinenes F–Q. The metabolites were isolated using both bioassay- and ^1^H NMR-guided approaches. One-/two-dimensional NMR spectroscopy and high-resolution mass spectrometry were used for structure elucidation, while a combination of NOESY NMR experiments, GIAO NMR calculations coupled with DP4+ probabilities measures, and ECD comparisons were incorporated to propose relative and absolute configurations of the anthoteibinenes. The metabolites were screened against the Respiratory Syncytial Virus (RSV), ESKAPE pathogens, five *Candida albicans* strains, and one strain of *C. auris*.

## 1. Introduction

Over 60% of marketed drugs are natural products or their derivatives, making this source of new chemodiversity significant in the continuing search for new pharmaceuticals [1]. Marine organisms from the deep sea have gained attention in recent years due to their promising secondary metabolites and new technology, like remotely operating vehicles (ROVs), making it possible to investigate these relatively unexplored areas [2,3]. Organisms found in the deep sea have the potential to produce distinctive metabolites due to the unique combination of predators and physical environment, including lack of light and low levels of oxygen [4]. Our group has recently reported five new cadinene-like sesquiterpenes from the Irish deep-sea soft coral *Anthothela grandiflora* (Octocorallia, Alcyoniidae), with unusual substitution by dimethylamine-bearing groups [5], illustrating the unique chemistry that denizens of the deep sea have to offer. Here, we report on our continuing investigation of the deep-sea coral *A. grandiflora*.

Our specimens of *A. grandiflora* were collected from the Whittard Canyon, south of Ireland, at depths between 1304 and 1814 m. A lipophilic extract was screened for biological activity to identify its biomedical potential. The screening identified the extract as a promising hit for multiple *Candida* spp. Based on its activity profile, the extract was chosen for fractionation using a ^1^H NMR-guided approach until purified metabolites were obtained. We report here the isolation of 12 new sesquiterpenes, anthoteibinenes F–Q (Anthoteibinenes derive their name from a contraction of the genus, *Anthothela*, and the Irish word for terpene, *teibín*), bearing the cadinene-like carbon scaffold. Structure elucidation of the metabolites was achieved using 1D and 2D NMR and HRESIMS/HREIMS. Relative configurations of the anthoteibinenes were determined by NOE, *J* analysis, ^1^H and ^13^C shift predictions, and DP4+ probability analysis [6,7,8,9]. Comparisons of predicted and experimental ECD spectra were used to assign the absolute configuration.

## 2. Results and Discussion

### 2.1. Isolation of the Anthoteibinenes

**Figure 1:**
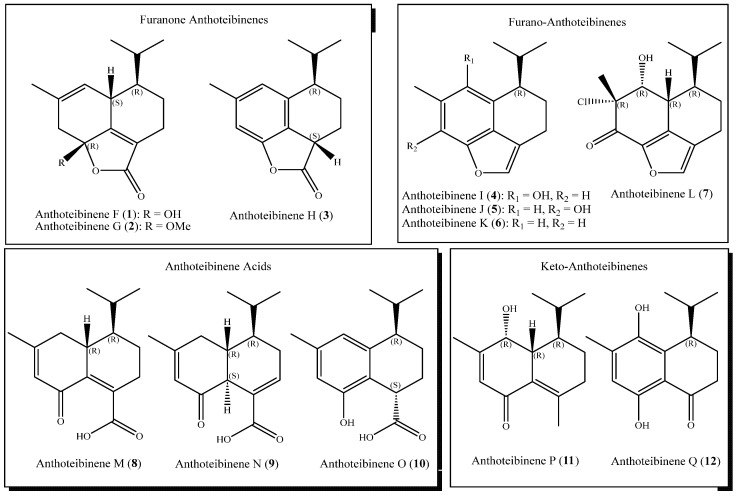
Anthoteibinenes F–Q, sesquiterpenes from the Irish deep-sea coral *Anthothela grandiflora*.

Dichloromethane and methanol extracts of freeze-dried *Anthothela grandiflora* were fractionated into six fractions, each of decreasing polarity using reversed-phase vacuum liquid chromatography (VLC). Upon fractionation, ^1^H NMR spectroscopy identified samples with multiple methyl doublets, indicative of potential terpenoid metabolites. Repeated rounds of fractionation using reversed-phase chromatography yielded 12 new cadinene-like sesquiterpenes, anthoteibinenes F–Q (Figure 1).

The twelve anthoteibinenes reported herein lack the amine feature previously reported [5] but share structural features useful for structure analysis. Anthoteibinenes F–H (**1**–**3**) each finds the cadinene-like C-14 position oxidized and cyclized into a furanone ring, while I–L (**4**–**7**) are similarly metabolized but with resultant furan rings. Anthoteibinene L (**7**) is further distinguished as the only halogenated member of the anthoteibinene family. Three anthoteibinenes, M–O (**8**–**10**), have unadorned carboxylic acids at C-14. Of the remaining anthoteibinenes, one, P (**11**), is unsubstituted at C-14, while Q (**12**) is a C-14 nor-sesquiterpene.

#### 2.1.1. The Furanone Anthoteibinenes (**1**–**3**)

Anthoteibinene F (**1**) was found with the molecular formula C_15_H_20_O_3_ (*m*/*z* 249.1503 [M + H]^+^), which requires six degrees of unsaturation. The ^13^C NMR spectrum (Table 1) corroborated the formula, displaying 15 carbon signals, including 5 quaternary carbons and 4 methine, 3 non-equivalent methylene, and 3 methyl groups. The ^1^H NMR data (Table 1) show signals for an olefinic methine at δ_H_ 5.58 (H-5); three aliphatic methines, δ_H_ 1.19 (H-7), δ_H_ 2.12 (H-11), and δ_H_ 3.00 (H-6); three non-equivalent methylene groups at δ_H_ 1.35/δ_H_ 1.91 (H-8a/H-8b), δ_H_ 2.09/δ_H_ 2.42 (H-9a/H-9b), and δ_H_ 2.50/δ_H_ 2.71 (H-3a/H-3b); and three methyl groups at δ_H_ 0.89 (H_3_-13), δ_H_ 1.03 (H_3_-12), and δ_H_ 1.77 (H_3_-15). This accounts for 19 protons, suggesting 1 exchangeable proton.

**Figure 2:**
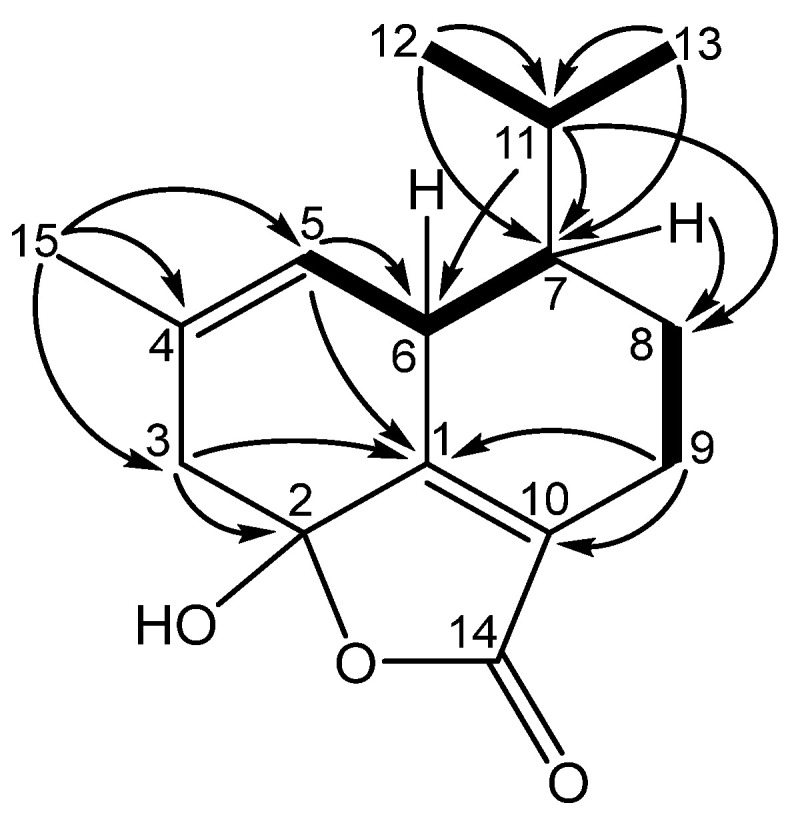
Key COSY (bold bonds) and HMBC (arrows) correlations establishing the planar structure of anthoteibinene F (**1**).

Key COSY correlations in anthoteibinene F (**1**) (Figure S5) from H-6 (δ_H_ 3.00) to H-5 (δ_H_ 5.58) and H-7 (δ_H_ 1.19) and from H_2_-8 to H_2_-9 were combined with HMBC correlations from H-7 to C-8 (δ_C_ 21.1), H_2_-9 to C-10 (δ_C_ 127.1), and C-1 (δ_C_ 162.0) to establish a spin system comprising nearly half of the carbon atoms (Figure 2). COSY correlations from H-11 to both H_3_-12 and H_3_-13 suggested the presence of the isopropyl group observed in previously reported anthoteibinenes; HMBC correlations from H_3_-12 and H_3_-13 to C-11 (δ_C_ 26.8) and C-7 (δ_C_ 45.3) established its attachment to C-7. This was corroborated by HMBC correlations from H-11 to C-6 (δ_C_ 35.5), C-7 (δ_C_ 45.3), and C-8 (δ_C_ 21.1). The growing spin system could be further extended by observation of methyl singlet (H_3_-15) HMBC correlations to C-3 (δ_C_ 43.4), C-4 (δ_C_ 131.1), and C-5 (δ_C_ 120.3). The olefinic proton H-5 displays an HMBC correlation to C-6 and C-1, revealing a six-membered ring as part of the spin system. A second, fused, six-membered ring could be established by observation of H-3a HMBC correlations to C-1 and C-2 (δ_C_ 102.0). The chemical shift of C-2 recommends it as a hemi/ketal, which, taken with the last open valence on C-10, accommodates the remaining unaccounted carbonyl carbon, C-14 (δ_C_ 170.2), as a furanone ring. Hemiketal C-2 is completed with the remaining unaccounted oxygen atom and its aforementioned exchangeable proton, resulting in the planar structure for anthoteibinene F (**1**).

Anthoteibinene G (**2**) was found with the same carbon skeleton as anthoteibinene F (**1**). The molecular formula of C_16_H_22_O_3_ (HRESIMS proton adduct ion at *m*/*z* 263.1658) and NMR data (Table S2) of **2** identified the additional CH_3_ group by the appearance of methoxy signals [δ_H_ 3.22 (s), δ_C_ 50.9 (CH_3_-16)]. H_3_-16 displayed an HMBC correlation to C-2, establishing anthoteibinene G (**2**) as the methoxy ketal of anthoteibinene F (**1**).

The molecular formula of anthoteibinene H (**3**), C_15_H_18_O_2_ (HREIMS [M]^+^ *m*/*z* 230.1308), was supported by the ^13^C NMR data (Table 2), which displayed 15 carbon signals, 6 of which were indicative of aromaticity. The multiplicity-edited HSQC spectrum, taken with the ^13^C NMR spectrum, suggested that **3** had one carbonyl group (δ_C_ 177.8, C-14); four additional quaternary carbons at δ_C_ 120.4 (C-1), δ_C_ 153.4 (C-2), δ_C_ 135.9 (C-4), and δ_C_ 141.7 (C-6); five methine carbons at δ_C_ 113.7 (C-3), δ_C_ 120.8 (C-5), δ_C_ 43.3 (C-7), δ_C_ 37.4 (C-10), and δ_C_ 30.7 (C-11); two non-equivalent methylene carbons, δ_C_ 21.2 (C-8) and δ_C_ 24.8 (C-9); and three methyl groups, δ_C_ 21.7 (C-12), δ_C_ 18.0 (C-13), and δ_C_ 21.3 (C-15).

Analysis of the COSY and HMBC spectra of anthoteibinene H (**3**) was sufficient to assign the planar structure. A singlet aromatic methyl group (H_3_-15: δ_H_ 2.13) displayed HMBC correlations to three aromatic carbons, positioning C-4 as the methyl-bearing aromatic carbon with adjacent protonated carbons C-3 and C-5. HMBC correlations from H-3 (δ_H_ 6.17) to C-1 and C-2, as well as from H-5 (δ_H_ 6.60) to C-1, established an aromatic ring sub-structure. H-5 was further correlated in the HMBC spectrum to C-7. H-7 (δ_H_ 2.64) displayed COSY correlations to H-11 (δ_H_ 2.21), H-11 to H_3_-12 (δ_H_ 1.02), and H_3_-13 (δ_H_ 0.76), establishing an isopropyl group on C-7. The remaining carbons could be assembled by observation of COSY correlations from H-7 to H_2_-8a (δ_H_ 1.94) and H_2_-8b (δ_H_ 1.56), HMBC correlations from H-10 (δ_H_ 4.05) to C-1, C-8, C-9, and C-14, resulting in a bicyclic structure with isopropyl and carboxylate substituents mirroring those found in anthoteibinenes F and G (**1**, **2**). Remaining elements of the molecular formula require oxygen at C-2, cyclized to C-14 as a lactone.

#### 2.1.2. The Furano-Anthoteibinenes (**4**–**7**)

Anthoteibinenes I-L (**4**–**7**) are related as furan-bearing metabolites. A cyclohexane ring for all three is evident, starting with the now-familiar isopropyl group and observing COSY and HMBC correlations as was found in the other anthoteibinenes: COSY among the H_3_-12/H_3_-13 to H_2_-9 segment, HMBC from H-7 to aromatic C-1, C-5, and C-6, and H-9 to C-1, C-10, and C-14 (see Table 3 for shifts). The aromatic ring of **4** (C_15_H_18_O_2_ from HRESIMS *m/z* 231.1389, [M + H]^+^) could be completed by observation of HMBC correlations from the phenolic proton at δ_H_ 7.92 to C-4 (δ_C_ 123.1), C-5 (δ_C_ 147.0), and C-6 (δ_C_ 120.1), establishing the position of the phenol group on C-5, from an aromatic methyl at δ_H_ 2.25 (H_3_-15) to δ_C_ 109.4 (C-3), C-4, and C-5, and from aromatic proton δ_H_ 7.05 (H-3) to δ_C_ 125.2 (C-1), C-4, and C-5; the planar structure was completed by observation of and HMBC correlations between δ_H_ 7.44 (H-14) and δ_C_ 146.8 (C-2) establishing a benzofuran ring system. Anthoteibinene J (**5**) ([M + H]^+^ *m/z* 231.1389) was isomeric to **4** and could be assigned as the C-3 phenol by observation of HMBC correlations from δ_H_ 6.74 (H-5) to C-3 (δ_C_ 137.5), C-4 (δ_C_ 119.9), and C-7 (δ_C_ 41.9), as well as δ_H_ 2.59 (H-7) to the protonated aromatic carbon δ_C_ 122.2 (C-5). And a third benzofuran, anthoteibinene K (**6**) (C_15_H_18_O_2_ from HREIMS *m/z* 214.1363), was found as the related metabolite lacking phenolic groups observed in **4** and **5**. This was established by observation of three aromatic protons, δ_H_ 7.19 (H-3), δ_H_ 6.95 (H-5), and δ_H_ 7.60 (H-14), the first two of which correlated in the HMBC with an aromatic methyl at δ_C_ 21.8 (C-15). H-14 was found to correlate with an oxygen-bearing aromatic carbon at δ_C_ 152.9 (C-2) as well as quaternary aromatic carbons δ_C_ 124.9 (C-1) and C-10 (δ_C_ 116.5). The 2D structure of **6** matches the published metabolite acorafuran, isolated from the flowering plant *Acorus calamus* [10]. Acorafuran was published without assignment of the absolute configuration. The optical rotations of **6** and acorafuran were antipodal (${\left[\alpha \right]}_{D}^{22}$ + 24 (methanol) and ${\left[\alpha \right]}_{578}$ − 161 (chloroform), respectively), suggesting they are likely enantiomers, although the different magnitudes are not comparable due to the different experimental conditions.

The HREIMS spectrum of anthoteibinene L (**7**) displayed an *m/z* 282.1039 with a 3:1 isotopic pattern indicative of chlorine, supported by the calculated molecular formula of *m*/*z* 282.1028 for C_15_H_19_^35^ClO_3_ and fragmentation indicative of chlorine loss. In addition to the isopropyl-substituted cyclohexane spin system observed in other metabolites described herein, the ^1^H NMR data (Table 3) support one olefinic proton at δ_H_ 7.45 (H-14) with COSY correlations to δ_H_ 2.49 and δ_H_ 2.83 (H-9b, H-9a) and HMBC correlations to δ_C_ 140.0 (C-1), δ_C_ 142.0 (C-2), and δ_C_ 123.3 (C-10), extending the spin system to a furan ring, as demonstrated for anthoteibinenes I-K (**4**–**6**). Further extending the spin system is a resonance at δ_H_ 4.35 (H-5) with COSY correlation to δ_H_ 3.49 (H-6) and HMBC correlation to C-1 in the furan ring, ketone δ_C_ 177.4 (C-3), and quaternary deshielded carbon δ_C_ 69.2 (C-4). The deshielded carbons C-4 and C-5 (δ_C_ 78.6) are the only open valences for the remaining heteroatoms, O and Cl, with the most deshielded position (C-5) assigned as the hydroxy-bearing carbon.

#### 2.1.3. The Anthoteibinene Acids (**8**–**10**)

**Figure 3:**
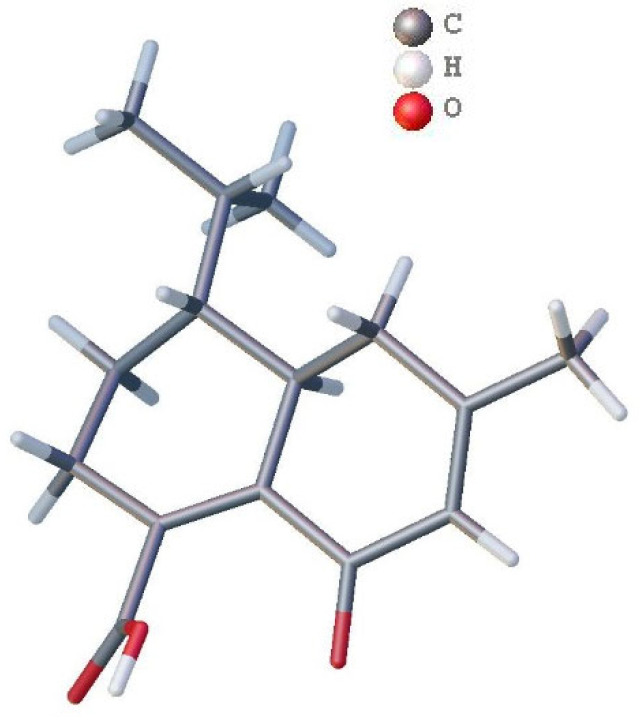
Wire frame plot of anthoteibinene M (**8**).

Anthoteibinene M (**8**) ([M + H]^+^ *m/z* 249.1499) has substantial NMR signal and correlation map overlap with the NMR data of anthoteibinene F (**1**). Differences are observed, however, in the ^13^C NMR shift (Table 4) of C-2 (δ_C_ 102.0 in **1**; 190.6 in **8**), and in the nature of C-5 and C-3, as assigned by the 2D NMR spectra. The noted differences between the two metabolites indicate that the ketal of **1** has undergone hydrolysis to the ketone, with concomitant isomerization of the Δ^4^ olefin from **1** to the conjugated Δ^3^ olefin in **8**, all of which are supported by the NMR data (Table 4). Crystals of **8** obtained from 1:1 hexanes/ethyl acetate were subject to X-ray crystallography, confirming the structure and revealing the absolute configuration as 6*R*,7*R* (Figure 3).

Anthoteibinene N (**9**) provided an [M + H]^+^ of *m/z* 249.1500, establishing it as isomeric to anthoteibinene M (**8**). The ^1^H NMR data (Table 4) revealed a new, deshielded, olefinic proton at δ_H_ 7.10 (H-9) with COSY correlations to δ_H_ 2.22 (H-8a) and δ_H_ 1.97 (H-8b), the latter of which further correlates to δ_H_ 1.67 (H-7). H-7 has HMBC correlations to δ_C_ 41.7 (C-1), δ_C_ 38.6 (C-6), δ_C_ 27.1 (C-11), and δ_C_ 14.1 (C-13). COSY correlations of δ_H_ 2.18 (H-6) to H-1 (δ_H_ 3.63), H-5a (δ_H_ 2.62), H-5b (δ_H_ 2.48), and H-7 (δ_H_ 1.67) are indicative of the isomerization of the Δ^1(10)^ observed in **8** as now Δ^9^. This was confirmed by HMBC correlations from H-9 to C-1, δ_C_ 35.1 (C-7), δ_C_ 25.2 (C-8), and δ_C_ 170.9 (C-14). HMBC correlations from H-5b to C-1, δ_C_ 125.6 (C-3), δ_C_ 159.3 (C-4), C-6, and δ_C_ 24.5 (C-15) confirm the second olefin to be Δ^3^ as in **8**.

Anthoteibinene O (**10**) was found with an experimental mass of *m/z* 249.1499 ([M + H]^+^) for a molecular formula of C_15_H_20_O_3_. The NMR spectral data (Table S11) indicated substantial similarities to those of anthoteibinene H (**3**) with an average carbon Δδ of < 1.0 ppm and Δδ_max_ = 3.9 (C-14, δ_C_ 177.8 in **3**). Protons were similarly matched with Δδ of < 0.01 ppm and Δδ_max_ = 0.28 (H-10, δ_H_ 3.74 in **10**). Analysis of the 2D NMR data (Table S11) provided the same carbon skeleton, establishing anthoteibinene O (**10**) as the hydrolysis product of anthoteibinene H (**3**).

#### 2.1.4. The Keto-Anthoteibinenes (**11**, **12**)

Anthoteibinene P (**11**) was isolated as a white film with a molecular formula of C_15_H_22_O_2_ established by analysis of the HRESIMS proton adduct ion at *m/z* 235.1711. The 1D and 2D spectral data (Table S12) were found to match of that tatarinowin A [11] from *Acorus tatarinowii*, a plant used in traditional Chinese medicine (TCM). Anthoteibinene P (**11**) and tatarinowin A are enantiomeric, with our isolate measuring ${\left[\alpha \right]}_{D}^{22}$ + 53.3 and the TCM metabolite ${\left[\alpha \right]}_{D}^{22}$ − 55.3.

Anthoteibinene Q (**12**) appears as a nor-sesquiterpene, with formula C_14_H_18_O_3_ (HRESIMS *m/z* 234.1255 for the proton adduct). The ^1^H and ^13^C NMR spectral data (Table 5) provide evidence for 14 carbons and 16 protons, indicating two exchangeable protons. The 2D NMR data (Table S13) established an aromatic western ring, with an aromatic methyl group at δ_H_ 2.29 (H_3_-14) displaying HMBC correlations to δ_C_ 116.9 (C-3), δ_C_ 135.3 (C-4), and δ_C_ 143.0 (C-5), and with aromatic proton δ_H_ 6.65 (H-3) correlating to δ_C_ 114.6 (C-1), 157.2 (C-2), δ_C_ 143.0 (C-5), and δ_C_ 17.4 (C-14). Both C-2 and C-5 appear oxygenated based on their shifts, which is not incompatible with other anthoteibinene metabolites. The fused ring comprising C-7 through C-10 is only surprising as C-10 is found as a ketone (δ_C_ 204.4) and it is devoid of an attached carboxylate as seen in previous anthoteibinenes, accounting for the nor-terpenoid formula, as the isopropyl group also appears intact.

#### 2.1.5. Configurational Analysis

Establishing the stereochemical relationships in the anthoteibinenes varied in complexity, depending on the number of chiral centers and overlapping NMR signals. A combination of NOESY correlations and coupling constants were used to determine relative stereochemistry. When NOE correlations and coupling constants were inconclusive, relative configurations were determined by comparing experimental to calculated ^1^H and ^13^C chemical shifts of energy-minimized conformers of all possible stereoisomers. Then, experimental and calculated ECD spectra were used to establish the absolute stereochemistry of the reported compounds.

Anthoteibinene F (**1**) has three chiral centers, C-2, C-6, and C-7. The NOESY spectrum in DMSO-*d*_6_ (Figure S13) revealed correlations between H-6/H_3_-13, indicating an anti configuration for H-6 and H-7, as previously established for anthoteibinene M (**8**) by XRD. The hemiketal OH group on C-2 was established as *syn* to H-6 from NOESY correlation between the hydroxy proton and H-6. Similarly, the NOESY spectrum of anthoteibinene G (**2**) displayed correlations between H-6/H_3_-13 and H-6/H_3_-16, indicating **1** has the same relative configuration as **2**.

**Figure 4:**
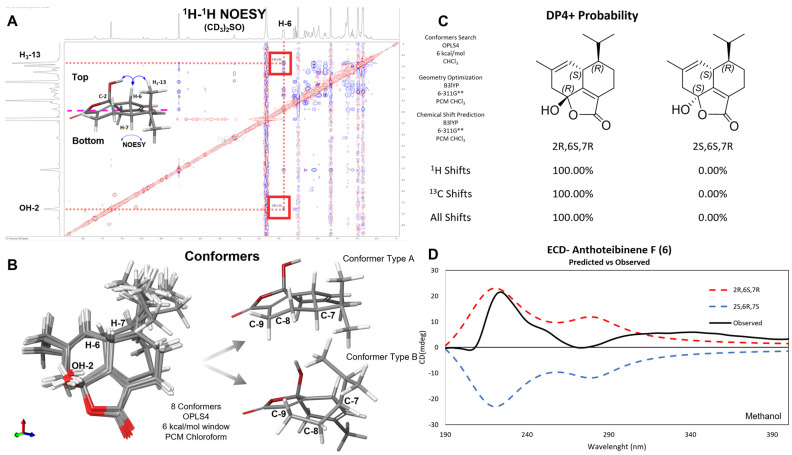
Stereochemical analysis of anthoteibinene F (**1**). (**A**) NOESY spectrum of **1**, with key NOE correlations highlighted. (**B**) Conformers of **1** found using OPLS4 highlighting two main conformer types. (**C**) DP4+ probability of two possible diastereomers of **1**. (**D**) Experimental ECD (black) spectrum vs. calculated spectra for 2*R*,6*S*,7*R* (red/upper broken trace) and 2*S*,6*R*,7*S* (blue/lower broken trace).

The relative configuration of **1** as 2*R*,6*S*,7*R* was confirmed by calculating the chemical shifts of four diastereomeric configurations using density functional theory (DFT) and the gauge-invariant atomic orbital (GIAO) model. Conformer searches were performed using OPLS4, searching a 6.0 kcal/mol window. Boltzmann-weighted average ^1^H and ^13^C chemical shift predictions were made using the PCM/B3LYP/6-311G **//B3LYP/6-311G ** level of theory. The predicted chemical shifts were compared to the experimental data using DP4+ probability scores [6], resulting in a 100% probability of 2*R*,6*S*,7*R* or 2*S*,6*R*,7*S*, when using all NMR data and both scaled and unscaled chemical shifts (Figure 4).

The absolute configuration of anthoteibinene F (**1**) was assigned by comparing experimental Electronic Circular Dichroism (ECD) and predicted ECD spectra for both enantiomers. The OPLS4 force field and mixed torsional/low-mode sampling were used to search for conformers in a 5.0 Kcal/mol window. ECD predictions were computed using Time-Dependent Density Functional Theory (TD-DFT) at the B3LYP-D3/LACVP**//B3LYP-D3/LACVP** level. Anthoteibinenes F and G (**1**, **2**) both showed positive Cotton effects at 220 nm, consistent with the 2*R*,6*S*,7*R* absolute configurations (Figure 4). This workflow was first validated by assigning the absolute configuration of anthoteibinene M (**8**), which was verified by X-ray crystallography (Figure 3).

**Figure 5:**
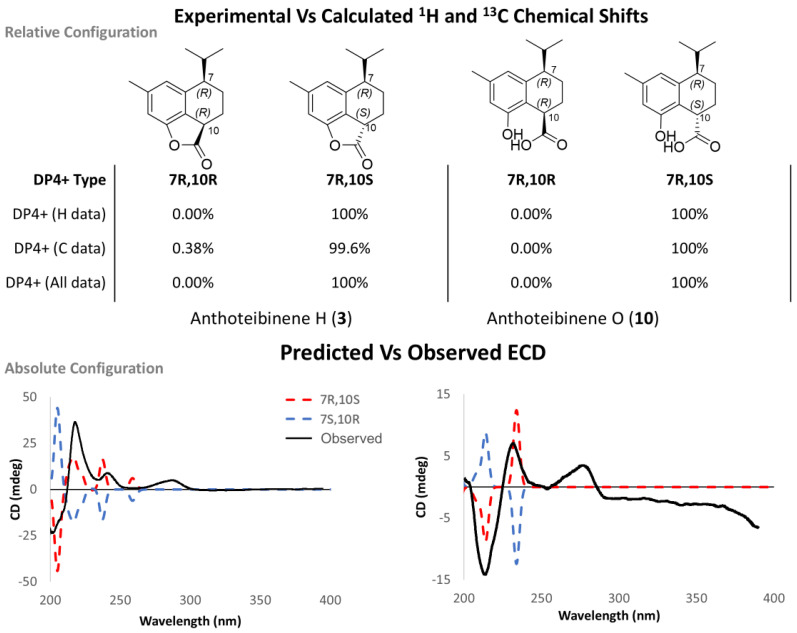
**(Top**) DP4+ probability of 7*R*,10*R* vs. 7*R*,10*S* of **3** and **10**. (**Bottom**) Experimental ECD (black) and predicted ECD (7*R*,10*R*, blue) and (7*R*,10*S*, red) spectra of **3** and **10**.

The anthoteibinenes H (**3**) and O (**10**) share a stereochemical relationship whereby two stereocenters are found in a 1,4 relationship on a cyclohexane ring. Determining these relative configurations was challenging due to the lack of discriminatory NOE correlations and indistinguishable coupling constants as a result of key signal overlaps in ^1^H NMR spectra (Tables S3 and S11, respectively). To distinguish between the two possible relative configurations syn and anti, with respect to H-7 and H-10, a comparison of predicted chemical shifts for both configurations, using the same workflow as **1**, resulted in a 100% DP4+ probability of H-7 and H-10 being in the anti configuration (Figure 5), when using unshielded predicted ^1^H and ^13^C chemical shifts. An absolute configuration of 7*R*,10*S* was deduced from an examination of predicted and experimental ECD spectra for all three compounds (Figure 5).

**Figure 6:**
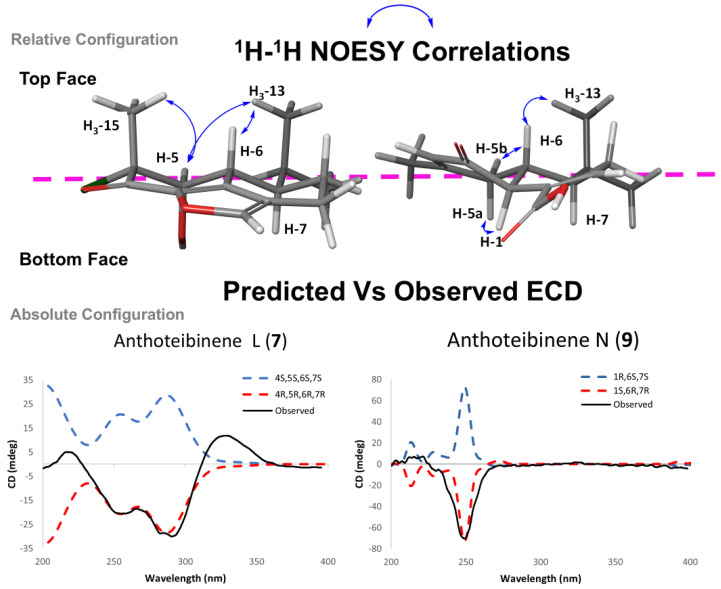
(**Top**) NOESY correlations establishing relative configuration of **7** and **9**, respectively, displayed on low-energy conformers. (**Bottom**) Experimental ECD (black) spectrum vs. calculated spectra for respective enantiomers (red/blue broken trace).

Anthoteibinene N (**9**) displayed key NOESY correlations from H_3_-13/H-6 and H-5b placing H-6 and the isopropyl group *syn* to one another. The relative configuration of H-1 was assigned *anti* to H-6, implied by NOE correlations from H-5a to H-1. An absolute configuration of 1*S*,6*R*,7*R* was determined by ECD spectral analysis (Figure 6).

Anthoteibinene L (**7**) has four chiral centers, C-4, C-5, C-6, and C-7. Clear NOE correlations from H_3_-13 to H-5 and H-6 place H-5 and H-6 syn to one another and anti to H-7. The relative configuration of C-4 was assigned through NOE correlations between H_3_-15/H-5, putting the methyl group on the same face as H-5. The NOESY spectrum (Figure S73) revealed a correlation from H-6 to the methylene at δ_H_ 1.53 (H-8b) and a weak correlation to δ_H_ 2.83 (H-9a), assigning H-8a and H-9a to the bottom face (Figure 6). An absolute configuration of 4*R*,5*R*,6*R*,7*R* was deduced from analysis of experimental and calculated ECD spectra (Figure 6).

Anthoteibinene P (**11**) has three chiral centers, C-5, C-6, and C-7. NOESY correlations between H-5/H-11 and H-6/H_3_-13 assigned H-5 and H-6 as syn and H-6/H-7 as anti. An absolute configuration of 5*R*,6*R*,7*R* was assigned through interpretation of predicted and observed ECD spectra. Tong et al. assigned H-5 and H-6 of tatarinowin A as anti, resulting an absolute configuration of **11** as 5*S*,6*R*,7*R* [11].

An absolute configuration of 7*R* was assigned to compounds **4**–**6** by comparing predicted ECD spectra for both enantiomers to the observed ECD spectra (Figures S45, S56 and S65).

#### 2.1.6. Biological Activity of Anthoteibinenes

The newly isolated terpenes were screened for biological activity in several bioassays, including multiple strains of *Candida albicans* and *C. auris.* Anthoteibinene I (**4**) and anthoteibinene J (**5**) were the only compounds with antifungal activity when tested at a concentration of 50 μg/mL against the six *Candida albicans* strains: MYA-2876, ATCC-18804, ATCC-28121, ATCC-76485, and ATCC-90029, as well as one *C. auris* strain: AR0385. Anthoteibinene J (**5**) displayed inhibition with an IC_50_ of 7.0 μg/mL for the 90,021 strain, while anthoteibinene I (**4**) lost inhibition when tested at concentrations lower than 50 μg/mL for all strains. Anthoteibinene K (**6**) has the same backbone as the two molecules but lacks the phenol functional group. Anthoteibinene K (**6**) showed no inhibition when tested, suggesting the phenol and its position to be vital for activity toward *Candida*, as the only difference between the molecules is the presence and the placement of the phenol between C-3 and C-5. Table 6 shows the IC_50_ (µg/mL) values for the six strains tested for anthoteibinene J (**5**) calculated using Prism. Fluconazole (positive), DMSO (negative), and triplicate drug-free, yeast-free wells served as controls. Antifungal activity has been shown for essential oils containing cadinene-like compounds, similarly to the anthoteibinenes; however, the studies lack bioactivity in the isolated compounds [12,13,14].

## 3. Materials and Methods

### 3.1. General Experimental Procedures

Optical rotations were measured using an AutoPol IV digital polarimeter (Rudolph Research, Hackettstown, NJ, USA) at 589 nm with a 1 dm or 0.1 dm path length cell. UV/Vis and ECD spectra were measured in MeOH using a JASCO Model J-1500 Circular Dichroism Optical Rotatory Dispersion (Jasco Products Company, Oklahoma City, OK). NMR spectra were acquired using a Bruker Neo 400 MHz broadband spectrophotometer (Bruker Scientific LLC, Billerica, MA) with a cryoprobe, a Varian Inova 500 MHz spectrophotometer (Agilent, Santa Clara, CA, USA), or a Bruker Neo 600 MHz broadband spectrophotometer. The residual solvent peaks were used as an internal chemical shift reference (CDCl_3_: δ_C_ 77.0; δ_H_ 7.27, (CD_3_)_2_SO: δ_C_ 39.5; δ_H_ 2.50). High-resolution mass spectrometry–liquid chromatography data were obtained on an Agilent 6540 LC-MS QTOF coupled to an Agilent Jet-stream electrospray ionization detector (Santa Clara, CA, USA). H_2_O (A) and 0.1% FA in CH_3_CN (B) were used as mobile phases on a Phenomenex Kinetex C18 column (2.6 mm, 100 Å, 150 × 3 mm: 0.5 mL/min). High-resolution mass spectrometry–gas chromatography data were obtained on an Agilent 7890A GC using a Zebron ZB-5HT Inferno (30 m × 0.25 mm, 0.25 mm film thickness) column coupled to an Agilent 7200 accurate-mass QTOF with electron impact ionization. Reversed-phase HPLC was performed on a Shimadzu LC20-AT system equipped with a photodiode array detector (M20A) using a preparative Phenomenex C18 column (5 mm, 100 Å, 250 × 21.2 mm: 10 mL/min) or a semi-preparative Phenomenex C18 column (10 mm, 100 Å, 250 × 10 mm: 4 mL/min. The methanol and acetonitrile used for column chromatography were obtained from Fisher Co. and were HPLC grade (>99% purity), while the H_2_O was distilled and filtered.

### 3.2. Biological Materials, Extraction, and Isolation

Specimens of *Anthothela grandiflora* were collected at depths between 1304 and 1814 m along the Irish continental margin during a 2017 cruise using the ROV *Holland I* deployed from the Irish national research vessel R/V *Celtic Explorer*. Specimens were stored in bioboxes on the ROV and immediately pooled, logged, labeled, and frozen at −80 °C when the ROV was recovered to the vessel. Specimens were freeze-dried on return to land and then stored until analysis at −20 °C.

The freeze-dried coral (734.7 g) was crushed and exhaustively extracted in DCM (1) via Soxhlet before being extracted with MeOH (2) at ambient temperature. The extracts were separately mixed with 50 g of C18 and eluted into six fractions of decreasing polarity using RP-C18 Vacuum Liquid Chromatography (VLC). The DCM extract (25.5 g) was eluted with (A) 50% MeOH/H_2_O, (B) 75% MeOH/H_2_O, (C) 100% MeOH, (D) 25% DCM/MeOH, (E) 50% DCM/MeOH, and (F) 100% DCM.

Fraction 1B (180 mg) was reconstituted in MeOH, filtered, and fractionated using semipreparative C18 HPLC using a linear gradient of 20–100% ACN/H_2_O for 90 min. Fractions were collected by UV peak and purified further with a gradient of 50–100% MeOH/H_2_O for 60 min. The fractions were determined to be pure by ^1^H NMR spectroscopy, resulting in the following pure compounds: anthoteibinene L (**7**: 1.0 mg), anthoteibinene P (**11**: 5.0 mg), and anthoteibinene Q (**12**: 2.8 mg). The same method was used for the initial fractionation of 1E (695 mg), resulting in anthoteibinene F (**1**: 6 mg) and anthoteibinene M (**8**: 1.1 mg).

Fractions 1C (2526 mg) and 1D (3767 mg) went through a 90% aqueous MeOH/hexane partition to remove large amounts of fats. The methanol fractions were dried to yield 500 mg and 650 mg, respectively. Both fractions went through fractionation as described for 1B and resulted in anthoteibinene I (**4**: 3.0 mg), anthoteibinene J (**5**: 3.3 mg), and anthoteibinene K (**6**: 12.5 mg) from fraction 1C and anthoteibinene G (**2**: 3.0 mg), anthoteibinene H (**3**: 2.0 mg), anthoteibinene N (**9**: 0.8 mg), and anthoteibinene O (**10**: 1.9 mg) from fraction 1D.

### 3.3. Spectroscopic Data for the Anthoteibinenes (1–12)

Anthoteibinene F (**1**): white film; ${\left[\alpha \right]}_{D}^{22}$ − 70 (*c* 0.1, MeOH); UV (MeOH) λ_max_ (log ε) 206.5 nm (1.50); ^1^H (600 MHz) and ^13^C (150 MHz) NMR data, Table S1; HRESIMS *m/z* 249.1503 [M + H]^+^ (calcd for C_15_H_21_O_3_, 249.1496; Δ 2.81 ppm).

Anthoteibinene G (**2**): colorless oil; ${\left[\alpha \right]}_{D}^{22}$ − 97 (*c* 0.3, MeOH); UV (MeOH) λ_max_ (log ε) 206.5 nm (1.50); ^1^H (600 MHz) and ^13^C (150 MHz) NMR data, Table S2; HRESIMS *m/z* 263.1658 [M + H]^+^ (calcd for C_16_H_23_NO_3_, 263.1653; Δ 1.90 ppm).

Anthoteibinene H (**3**): white film; ${\left[\alpha \right]}_{D}^{22}$ + 3 (*c* 0.3, MeOH); UV (MeOH) λ_max_ (log ε) 211.5 nm (1.42); ^1^H (400 MHz) and ^13^C (100 MHz) NMR data, Table S3; HREIMS *m/z* 230.1308 [M]^+^ (calcd for C_15_H_18_O_2_, 230.1312; Δ −1.74 ppm).

Anthoteibinene I (**4**): colorless oil; ${\left[\alpha \right]}_{D}^{22}$ − 5 (*c* 0.1, MeOH); UV (MeOH) λ_max_ (log ε) 209.0, 255.5 (sh) nm (1.53, 1.16); ^1^H (600 MHz) and ^13^C (150 MHz) NMR data, Table S4; HRESIMS *m/z* 231.1389 [M + H]^+^ (calcd for C_15_H_19_O_2_, 231.1391; Δ −0.87 ppm).

Anthoteibinene J (**5**): colorless oil; ${\left[\alpha \right]}_{D}^{22}$ + 1 (*c* 0.3, MeOH); UV (MeOH) λ_max_ (log ε) 223.0, 254.0 (sh) nm (1.56, 1.44); ^1^H (600 MHz) and ^13^C (150 MHz) NMR data, Table S5; HRESIMS *m/z* 231.1392 [M + H]^+^ (calcd for C_15_H_19_O_2_, 231.1392; Δ 0.43 ppm).

Anthoteibinene K (**6**): colorless oil; ${\left[\alpha \right]}_{D}^{22}$ + 24 (*c* 1.0, MeOH); UV (MeOH) λ_max_ (log ε) 213.4, 251.7 (sh) nm (1.42, 0.85); ^1^H (400 MHz) and ^13^C (100 MHz) NMR data, Table S6; HREIMS *m/z* 214.1358 [M]^+^ (calcd for C_15_H_18_O, 214.1363; Δ −2.40 ppm).

Anthoteibinene L (**7**): white film; ${\left[\alpha \right]}_{D}^{22}$ − 33 (*c* 0.3, MeOH); UV (MeOH) λ_max_ (log ε) 198.7 nm (0.45); ^1^H (600 MHz) and ^13^C (150 MHz) NMR data, Table S7; HRESIMS *m/z* 283.1117 [M + H]^+^ (calcd for C_15_H_19_^35^ClO_3_, 283.1101; Δ 5.66 ppm); 285.1084 [M + H]^+^ (calcd for C_15_H_19_^37^ClO_3_, 285.1071; Δ 4.39 ppm).

Anthoteibinene M (**8**): white film; ${\left[\alpha \right]}_{D}^{22}$ + 72 (*c* 0.1, MeOH); UV (MeOH) λ_max_ (log ε) 203.5, 252.5 (sh) nm (1.39, 1.17); ^1^H (600 MHz) and ^13^C (150 MHz) NMR data, Table S8; HRESIMS *m/z* 249.1499 [M + H]^+^ (calcd for C_15_H_21_O_3_, 249.1496; Δ 1.20 ppm).

Anthoteibinene N (**9**): white film; ${\left[\alpha \right]}_{D}^{22}$ − 120 (*c* 0.2, MeOH); UV (MeOH) λ_max_ (log ε) 201.2 nm (0.71); ^1^H (400 MHz) and ^13^C (100 MHz) NMR data, Table S10; HRESIMS *m/z* 249.1500 [M + H]^+^ (calcd for C_15_H_21_O_3_, 249.1496; Δ 1.61 ppm).

Anthoteibinene O (**10**): white film; ${\left[\alpha \right]}_{D}^{22}$ + 20 (*c* 0.3, MeOH); UV ((MeOH) λ_max_ (log ε) 212.9, 284.2 (sh) nm (1.42, 0.05); ^1^H (400 MHz) and (100 MHz) NMR data, Table S11; HRESIMS *m/z* 249.1499 [M + H]^+^ (calcd for C_15_H_21_O_3_, 249.1496; Δ 1.20 ppm).

Anthoteibinene P (**11**): white film; ${\left[\alpha \right]}_{D}^{22}$ + 53 (*c* 0.15, MeOH); UV ((MeOH) λ_max_ (log ε) 198.5 nm (0.45); ^1^H (400 MHz) and ^13^C (100 MHz) NMR data, Table S12; HRESIMS *m/z* 235.1711 [M + H]^+^ (calcd for C_15_H_23_O_2_, 235.1704; Δ 2.98 ppm).

Anthoteibinene Q (**12**): white film; ${\left[\alpha \right]}_{D}^{22}$ + 10 (*c* 0.3, MeOH); UV (MeOH) λ_max_ (log ε) 210.4, 238.2 (sh), 274.5 (sh) nm (0.53, 0.19, 0.41); ^1^H (600 MHz) and ^13^C (150 MHz) NMR data, Table S13; HREIMS *m/z* 234.1255 [M]^+^ (calcd for C_14_H_18_O_3_, 234.1261; Δ −2.75 ppm).

### 3.4. Antifungal Activity

In vitro antifungal activity was tested against five strains of *Candida albicans* (MYA-2876, ATCC-18804, ATCC-28121, ATCC-76458, and ATCC-90029) and one strain of *Candida auris* (AR0385) following the Reference Method for Broth Dilution Antifungal Susceptibility Testing of Yeasts [15]. Organisms were subcultured on Sabouraud dextrose agar and incubated at 35 °C for 24 h. Inoculum was prepared by picking five 24 h old colonies of 1 mm in diameter and suspending them in Sabouraud dextrose broth. The resulting suspension was then vortexed for 15 s and the optical density at 600 nm (OD600) adjusted to 1.00. This resulted in a stock solution of 10^6^ cells/mL. A working solution was made by diluting the stock solution at a 1:100 dilution followed by a 1:20 dilution, resulting in a concentration of 5.0 × 10^2^ cells/mL.

A stock solution of anthoteibinene J (**5**) was prepared at 2.5 mg/mL in DMSO. This was used to prepare 9 working solutions ranging in concentration from 1.25 mg/mL to 4.88 µg/mL. Triplicate aliquots of 1.0 µL were further transferred onto a 96-well plate. A positive standard of fluconazole, a negative control (1.0 µL of DMSO), and a triplicate of drug-free, yeast-free wells were also plated. Yeast inoculate (99 µL) was added to each well, resulting in concentrations ranging from 25 µg/mL to 48.8 µg/mL. Cultures were then incubated at 35 °C for 24 h. Yeast growth was measured using optical density at 600 nm with a Biotek 800 TS plate reader. The optical density of each well was compared to the negative control to establish the growth of yeast present within each well. IC_50_ was determined by plotting the log of concentration to the percentage of growth of yeast using a nonlinear regression in Prism.

### 3.5. Computational Methods

All molecular mechanics and quantum mechanics calculations were conducted using the boltzmann_averaged_properties.py script, integrating Macromodel and Jaguar (version 2023-1, Schrodinger LLC, New York, NY, USA).

#### 3.5.1. Electronic Circular Dichroism Spectral Predictions

Conformation searches for each enantiomer utilized OPLS4 to generate low-energy conformers within a 5 kcal/mol energy window, in the liquid phase, incorporating a water PCM solvation model. Conformers underwent geometry optimization and subsequent relative thermal free energies (ΔG) at 298.15 K, using DFT at the B3LYP-D3/LACVP** level. Geometry optimization was carried out using a methanol PCM solvation model, while single-point energy calculations were conducted using a PBF solvent model for improved energy calculation accuracy. Conformers with negative vibrational frequencies were removed. ECD spectra for each conformer were computed using TD-DFT at the B3LYP-D3/LACVP** level, using 20 excited states generated by Tamm–Dancoff approximation. Boltzmann conformer populations were used to create a weighted averaged ECD spectrum. ECD spectra for anthoteibinene L (**7**) were calculated at the ωB97x-D3/6-311G+**//ωB97x-D3/6-311G+** level due to the presence of a heavy atom. Experimental and predicted spectra were visualized using Excel. A comparison between experimental and predicted UV spectra was used to determine wavelength corrections and to match the intensities of signals. Metabolites were calculated as neutral molecules.

#### 3.5.2. GAIO NMR Predictions

An OPLS4 module was used for conformation generation with “mixed torsional/low-mode sampling” in the OPLS4 force field. A 6 kcal/mol energy window and a maximum of 3000 steps were employed for the search. Geometry optimization using Jaguar (version 2023-3, Schrodinger LLC) was performed at the B3lYP/6-113G** level in solvent phase using PCM (chloroform). ^1^H and ^13^C NMR chemical shifts were calculated at the same level, using gauge-invariant atomic orbital (GIAO) shielding constant calculations. A PBF solvent model was used for the calculation of free energies at B3lYP/6-113G** level. Boltzmann populations were computed and used to average NMR chemical shifts. Scaled and unscaled ^1^H and ^13^C NMR chemical shifts were used when calculated using the DP4+ excel sheet provided by Grimblat et al. [6]. Exchangeable protons were not included in DP4+ comparisons.

### 3.6. X-Ray Crystallography

Anthoteibinene M (**8**) was crystallized from a 50/50 mixture of hexanes and ethyl acetate in a refrigerator with limited oxygen for two days. X-ray diffraction data were measured on a Bruker D8 Venture PHOTON II CMOS diffractometer (Bruker, Madison, WI, USA) equipped with a Cu Kα INCOATEC ImuS micro-focus source (λ = 1.54178 Å). Indexing was performed using APEX4 (Difference Vectors method) [16]. Data integration and reduction were performed using SaintPlus [17]. Absorption correction was performed by the multi-scan method implemented in SADABS [18]. Space groups were determined using XPREP implemented in APEX3 [19]. Structures were solved using SHELXT and refined using SHELXL-2019/1 (full-matrix least squares on F2) through the OLEX2 interface program [20,21]. The ellipsoid plot was made with Olex2 [21]. Hydrogen atoms of −OH groups and H_2_O molecules were freely refined.

## 4. Conclusions

The unexplored deep-sea coral *Anthothela grandiflora* revealed new cadinene-like sesquiterpenes, anthoteibinenes F–Q, using both a bioassay-guided and ^1^H NMR-guided approach, the latter targeting the two doublet methyl groups of the isopropyl group with olefinic and aromatic signals. The compounds showed a variety of two- and three-ring systems and functional groups varying among alcohols, acetals, hemiacetals, ketones, esters, carboxylic acids, and amides. We were unable to find examples of natural-product cadinene-like metabolites with halogenation; only one example of a C-14 nor-cadinene was found [22], though that example lacked experimental data. Based on the co-occurrence of similar metabolites reported in the literature and the lack of inter-conversion of our isolated metabolites, we believe that they are all natural products [23,24]. One of the compounds showed moderate antifungal activity. The research shown here represent the unique biodiversity offered by the relatively unexplored deep sea. These results highlight the importance for further research of deep-sea organisms and its potential for the discovery of novel chemical compounds for the drug discovery field.

## Figures and Tables

**Table 1:** NMR data for anthoteibinene F (**1**) (600 (^1^H) and 150 (^13^C) MHz, CDCl_3_).

Position	δ_C_, Type	δ_H_, Mult. (*J* in Hz)	gCOSY	gHMBC
1	162.0, C			
2	102.0, C			
3a	43.4, CH_2_	2.50, d (17.5)	3b, 5, 15	2, 4, 5
3b		2.71, d (17.5)	3a	1, 2, 4, 5, 15
4	131.1, C			
5	120.3, CH	5.58, br s	3b, 6, 15	1, 3, 6, 15
6	35.5, CH	3.00, dd (2.1, 9.1)	7, 15	1, 5, 7
7	45.3, CH	1.19, dddd (2.1, 2.1, 9.1, 12.4)	6, 8a, 8b, 11	5, 6, 11, 12, 13
8a	21.1, CH_2_	1.91, dddd (2.1, 2.1, 5.0, 13.4)	7, 8b, 9b	6, 7, 10, 11
8b		1.35, dddd (5.0, 12.4, 12.4, 13.4)	7, 8a, 9a, 9b	6, 7, 9
9a	20.3, CH_2_	2.42, ddd (2.1, 5.0, 18.0)	8b, 9b	1, 7, 10
9b		2.09, o/l *	8a, 8b, 9b	1, 10
10	127.1, C			
11	26.8, CH	2.12, o/l *	7, 12, 13	6, 7, 12, 13
12	21.6, CH_3_	1.03, d (6.9)	11	7, 11, 13
13	15.8, CH_3_	0.89, d (6.9)	11	7, 11, 12
14	170.2, C			
15	23.5, CH_3_	1.77, s	3b, 5, 6	3, 4, 5

* Overlapping ^1^H NMR signals; 2D assignments based on proximity likelihood.

**Table 2:** NMR data for anthoteibinenes G and H (**2**, **3**) (400 (^1^H) and 100 (^13^C) MHz, CDCl_3_).

Position	2		3	
	δ_C_, Type	δ_H_, Mult. (*J* in Hz)	δ_C_, Type	δ_H_, Mult. (*J* in Hz)
1	160.6, C		120.4, C	
2	105.4, C		153.4, C	
3a	42.5, CH_2_	2.73, d (17.5)	113.7, CH	6.17, s
3b		2.41, d (17.5)		
4	131.4, C		135.9, C	
5	120.2, CH	5.55, s	120.8, CH	6.60, s
6	36.1, CH	2.86, dd (2.1, 9.1)	141.7, C	
7	45.5, CH	1.20, dddd (2.1, 2.1, 9.1, 12.4)	43.3, CH	2.64, ddd (5.0, 6.5, 6.4)
8a	21.3, CH_2_	1.93, dddd (2.1, 2.1, 5.0, 13.4)	21.2, CH_2_	1.94, dddd (3.0, 5.0, 8.5, 13.0)
8b		1.35, dddd (5.0, 12.4, 12.4, 13.4)		1.56, dddd (2.8, 6.5, 9.8, 13.0)
9a	20.6, CH_2_	2.48, ddd (2.1, 5.0, 18.0)	24.8, CH_2_	1.74, dddd (3.0, 6.5, 9.8, 13.0)
9b		2.13, o/l *		2.14, o/l *
10	129.4, C		37.4, CH	4.05, t (7.0)
11	26.8, CH	2.13, o/l *	30.7, CH	2.21, octet (6.4)
12	21.6, CH_3_	1.03, d (6.9)	21.7, CH_3_	1.02, d (6.8)
13	15.9, CH_3_	0.90, d (6.9)	18.0, CH_3_	0.76, d (6.8)
14	170.1, C		177.8, C	
15	23.5, CH_2_	1.75, s	21.2, CH_3_	2.13, s
16	50.9, CH_3_	3.22, s		

* Overlapping ^1^H NMR signals; 2D assignments based on proximity likelihood.

**Table 3:** NMR data for anthoteibinenes I–L (**4**–**7**).

Position	4		5		6		7	
	δ_C_, Type ^a^	δ_H_, Mult. (*J* in Hz) ^b^	δ_C_, Type ^a^	δ_H_, Mult. (*J* in Hz) ^b^	δ_C_, Type ^c^	δ_H_, Mult. (*J* in Hz) ^d^	δ_C_, Type ^e^	δ_H_, Mult. (*J* in Hz) ^f^
1	125.2, C		126.7, C		124.9, C		140.0, C	
2	146.8, C		141.7, C		152.9, C		142.0, C	
3	109.4, CH	7.05, s	137.5, C		108.4, CH	7.19, s	177.4, C	
4	123.1, C		119.9, C		134.0, C		69.2, C	
5	147.0, C		122.2, C	6.74, s	120.6, CH	6.95, s	78.6, CH	4.35, br d (2.9)
6	120.1, C		125.0, C		135.1, C		36.7, C	3.49, dd (2.8, 11.6)
7	37.5, CH	2.98, ddd (4.8, 5.2, 6.8)	41.9, CH	2.59, ddd (4.6, 5.8, 6.6)	42.4, CH	2.76, o/l *	39.9, CH	1.70, dddd (1.9, 2.2, 11.6, 11.6)
8a	25.7 CH_2_	2.23, o/l *	25.1 CH_2_	1.86, o/l *	24.9, CH_2_	1.96, m (2H)	22.3, CH_2_	2.01, br dddd (1.9, 5.4, 11.6, 12.1)
8b		1.54, dddd (4.8, 6.0, 12.5, 13.0)		1.81, o/l *				1.53, dddd (5.0, 11.6, 12.1, 12.6)
9a	15.9, CH_2_	2.66, m (2H)	17.7, CH_2_	2.65, ddd (5.6, 5.8, 16.1)	17.7, CH_2_	2.88, m	19.4, CH_2_	2.83, br ddd (5.4, 12.6, 16.6)
9b				2.76, ddd (4.9, 8.0, 16.0)		2.78, o/l *		2.49, ddd (5.0, 12.1, 16.6)
10	116.2, C		117.1, C		116.5, C		123.3, C	
11	31.1, C	1.72, octet (6.8)	29.0, C	2.03, octet (6.6)	28.8, CH	2.17, octet (6.9)	26.3, CH	2.09, d sept (2.2, 6.9)
12	20.7, CH_3_	0.94, d (6.7)	21.3, CH_3_	0.97, d (6.8)	21.2, CH_3_	1.08, d (6.8)	21.2, CH_3_	1.07, d (6.9)
13	21.2, CH_3_	0.91, d (6.7)	18.9, CH_3_	0.89, d (6.8)	18.8, CH_3_	0.99, d (6.8)	15.8, CH_3_	0.97, d (6.9)
14	137.6, CH	7.44, s	137.1, CH	7.50, s	137.3, CH	7.60, s	145.2, CH_3_	7.45, s
15	17.8, CH_3_	2.25, s *	16.2, CH_3_	2.24, s	21.8, CH_3_	2.50, s	22.8, CH_3_	1.88, s
OH		7.92, s		9.29, br s				

^a^150 MHz, (CD_3_)_2_SO; ^b^ 600 MHz, (CD_3_)_2_SO; ^c^ 100 MHz, (CD_3_)_2_SO; ^d^ 400 MHz, (CD_3_)_2_SO; ^e^ 150 MHz, CDCl_3_; ^f^ 600 MHz, CDCl_3_; * Overlapping ^1^H NMR signals, 2D assignments based on proximity likelihood.

**Table 4:** NMR data (CDCl_3_) for anthoteibinenes M–O (**8**–**10**).

Position	8		9		10	
	δ_C_, Type ^a^	δ_H_, Mult. (*J* in Hz) ^b^	δ_C_, Type ^c^	δ_H_, Mult. (*J* in Hz) ^d^	δ_C_, Type ^c^	δ_H_, Mult. (*J* in Hz) ^d^
1	137.0, C		47.1, CH	3.63, br d (3.9)	118.6, C	
2	190.6, C		199.3, C		153.8, C	
3	127.0, CH	6.05, s	125.6, CH	5.89, s	113.9, CH	6.41, s
4	165.0, C		159.3, C		136.9, C	
5a	37.5, CH_2_	2.59, dd (5.3, 17.8) *	32.2, CH_2_	2.62, br d (18.5)	121.8, CH	6.63, s
5b		2.18, dd (11.3, 17.8)		2.48, d (18.5)		
6	39.3, CH	2.65, o/l *	36.8, CH	2.18, o/l *	141.8, C	
7	44.9, CH	1.34, o/l *	35.1, CH	1.67, dddd (4.6, 4.6, 11.0, 11.0)	42.8, CH	2.55, ddd (2.0, 5.1, 10.5)
8a	19.9, CH_2_	1.75, br m	25.2, CH_2_	2.22, o/l *	20.8, CH_2_	1.87, br m
8b		1.31, o/l *		1.97, o/l *		1.65, br m
9a	28.7, CH_2_	2.53, o/l *	141.4, CH	7.10, br s	24.1, CH_2_	1.93, o/l *
9b						2.09, o/l
10	140.4, C		129.3, C		40.2, CH	3.74, br t (5.8)
11	27.1, CH	1.90, dsept (2.0, 6.7)	27.1, CH	1.83, octet (6.6)	31.6, CH	2.13, o/l
12	21.5, CH_3_	1.01, d (6.7)	20.6, CH_3_	0.89, d (6.8)	21.8, CH_3_	1.00, d (6.8)
13	16.2, CH_3_	0.82, d (6.7)	14.1, CH_3_	0.78, d (6.8)	18.5, CH_3_	0.76, d (6.8)
14	171.4, C		170.9, C		181.7, C	
15	24.5, CH_3_	2.03, s	24.5, CH_3_	1.99, s *	21.3, CH_3_	2.19, s

^a^ 150 MHz; ^b^ 500 MHz; ^c^ 100 MHz; ^d^ 400 MHz; * Overlapping ^1^H NMR signals, 2D assignments based on proximity likelihood.

**Table 5:** NMR data (CDCl_3_) for anthoteibinenes P and Q (**11, 12**).

Position	11		12	
	δ_C_, Type ^a^	δ_H_, Mult. (*J* in Hz) ^b^	δ_C_, Type ^c^	δ_H_, Mult. (*J* in Hz) ^d^
1	125.7, C		114.6, C	
2	190.6, C		157.2, C	
3	129.4, CH	5.94, s	116.9, CH	6.65, s
4	158.5, C		135.3, C	
5	68.6, CH	4.01, br d (2.6)	143.0, C	
6	44.2, CH	2.59, br dd (2.6, 9.8)	132.6, C	
7	39.4, CH	1.80, dddd (2.5, 2.8, 9.8, 12.5)	38.1, CH	2.87, ddd (3.1, 3.1, 9.5)
8a	19.9, CH_2_	1.72, dddd (2.5, 5.4, 11.0, 12.5)	33.3, CH_2_	2.29, o/l *
8b		1.22, dddd (5.5, 11.0, 12.5, 12.5)		2.04, dddd (5.3, 5.3, 14.0, 14.0)
9a	34.2, CH_2_	2.22, m	24.7, CH_2_	2.79, ddd (5.8, 14.0, 19.1)
9b				2.55, ddd (1.6, 5.3, 19.1)
10	153.1, C		204.4, C	
11	27.2, CH	1.95, d sept (2.8, 6.9)	31.3, CH	1.87, d sept (2.4, 6.7)
12	21.6, CH_3_	1.03, d (6.9)	21.3, CH_3_	1.10, d (6.5)
13	16.2, CH_3_	0.82, d (6.9)	21.2, CH_3_	0.91, d (6.7)
14	22.5, CH_3_	2.10, s *	17.4, CH_3_	2.29, s *
15	22.0, CH_3_	2.10, s *		
OH				12.21

^a^ 100 MHz; ^b^ 400 MHz; ^c^ 150 MHz; ^d^ 600 MHz; * Overlapping ^1^H NMR signals, 2D assignments based on proximity likelihood.

**Table 6:** Antifungal activity (IC_50_) of anthoteibinene J (**5**) toward *Candida* spp.

Strain	5 (µg/mL)	Fluconazole (µg/mL)
*C. albicans* MYA-2876	9.1 ± 0.64	0.25 ± 0.04
*C. albicans* ATCC-18804	7.7 ± 0.71	0.29 ± 0.09
*C. albicans* ATCC-28121	7.7 ± 0.79	0.61 ± 0.09
*C. albicans* ATCC-76485	8.2 ± 0.78	0.92 ± 0.26
*C. albicans* ATCC-90029	7.0 ± 0.81	0.19 ± 0.04
*C. auris* AR0385	10.0 ± 1.20	0.92 ± 0.26

## Data Availability

The NMR data for the following compounds have been deposited in the Natural Products Magnetic Resonance Database (NP-MRD; www.np-mrd.org) and can be found at NP0332688 (anthoteibinene F), NP0332689 (anthoteibinene G), NP0332690 (anthoteibinene H), NP0332691 (anthoteibinene I), NP0332692 (anthoteibinene J), NP0332693 (anthoteibinene K), NP0332694 (anthoteibinene L), NP0332695 (anthoteibinene M), NP0332696 (anthoteibinene N), NP0332697 (anthoteibinene O), NP0332698 (anthoteibinene P), and NP0332699 (anthoteibinene Q). The X-ray metadata have been deposited at the Cambridge Crystallographic Data Centre, deposition number 2345725. Other data not found in the Supplementary Materials will be available upon request to the corresponding author.

## Supplementary Materials

The following supporting information can be downloaded at: https://www.mdpi.com/article/10.3390/md23010044/s1, Figure S1–S124 and Table S1–S34: ^1^H, ^13^C, COSY, HSQC, and HMBC NMR spectra, as well as HRMS and ECD spectra of anthoteibinenes F–Q (**1**–**12**).
