# Supplementary material for: Anthoteibinenes F–Q: New Sesquiterpenes from the Irish Deep-Sea Coral Anthothela grandiflora
Source: Mar Drugs. 2025 Jan 17;23(1):44. doi: 10.3390/md23010044 (PMC11767152; doi:10.3390/md23010044)
Supplement: Supplementary file 1 [file marinedrugs-23-00044-s001.zip › marinedrugs-3379888-supplementary.pdf]

Anthoteibinenes F-Q, new sesquiterpenes from the Irish Deep-sea Coral *Anthothela grandiflora*

Stine S. H. Olsen,[1] Sam Afoullouss,[1] Ezequiel Cruz Rosa,[1] Ryan M. Young,[2] Mark Johnson,[2] A. Louise Allcock[2] and Bill. J. Baker[1]*

[1]*Department of Chemistry, University of South Florida, 4202 E. Fowler Avenue, CHE205, Tampa, Florida 33620, USA*

[2]*School of Natural Sciences and Ryan Institute, University of Galway, University Road, H91TK33 Galway*, Ireland

Table of Contents

Table S1 – NMR Data for Anthoteibinene F (**1**) 4
Table S2 – NMR Data for Anthoteibinene G (**2**) 12
Table S3 – NMR Data for Anthoteibinene H (**3**) 18
Table S4 – NMR Data for Anthoteibinene I (**4**) 24
Table S5 – NMR Data for Anthoteibinene J (**5**) 30
Table S6 – NMR Data for Anthoteibinene K (**6**) 36
Table S7 – NMR Data for Anthoteibinene L (**7**) 41
Table S8 – NMR Data for Anthoteibinene M (**8**) 47
Table S9 – Crystal Data and Structure Refinement for Anthoteibinene M (**8**) 52
Table S10 – NMR Data for Anthoteibinene N (**9**) 54
Table S11 – NMR Data for Anthoteibinene O (**10**) 59
Table S12 – NMR Data for Anthoteibinene P (**11**) 64
Table S13 – NMR Data for Anthoteibinene Q (**12**) 70
Table S14 – Predicted chemical shifts of possible anthoteibinene F (**1**) diastereomers 75
Table S15 – DP4+ probabilities of anthoteibinene F (**1**) diastereomers 76
Table S16 – Predicted chemical shifts of possible anthoteibinene H (**3**) diastereomers 77
Table S17 – DP4+ probabilities of anthoteibinene H (**3**) diastereomers 78
Table S18 – Predicted chemical shifts of possible anthoteibinene O (**10**) diastereomers 79
Table S19 – DP4+ probabilities of anthoteibinene O (**10**) diastereomers 80
Table S20 – Predicted conformers of anthoteibinene F (**1**), with calculated properties for ECD spectra predictions 81
Table S21 – Predicted conformers of anthoteibinene G (**2**), with calculated properties for ECD spectra predictions 82
Table S22 – Predicted conformers of anthoteibinene H (**3**), with calculated properties for ECD spectra predictions 83
Table S23 – Predicted conformers of anthoteibinene I (**4**), with calculated properties for ECD spectra predictions 84
Table S24 – Predicted conformers of anthoteibinene J (**5**), with calculated properties for ECD spectra predictions 85
Table S25 – Predicted conformers of anthoteibinene K (**6**), with calculated properties for ECD spectra predictions 86
Table S26 – Predicted conformers of anthoteibinene L (**7**), with calculated properties for ECD spectra predictions 87
Table S27 – Predicted conformers of anthoteibinene M (**8**), with calculated properties for ECD spectra predictions 88
Table S28 – Predicted conformers of anthoteibinene N (**9**), with calculated properties for ECD spectra predictions 89
Table S29 – Predicted conformers of anthoteibinene O (**10**), with calculated properties for ECD spectra predictions 90
Table S30 – Predicted conformers of anthoteibinene P (**11**), with calculated properties for ECD spectra predictions 91
Table S31 – Predicted conformers of anthoteibinene Q (**12**), with calculated properties for ECD spectra predictions 92
Table S32 – Predicted conformers properties of anthoteibinene F (**1**), for NMR chemical shift calculations 93
Table S33 – Predicted conformers properties of anthoteibinene H (**3**), for NMR chemical shift calculations 93
Table S34 – Predicted conformers properties of anthoteibinene O (**10**), for NMR chemical shift calculations 93

Figure S1 – Structure of **1** 4

Figure S2 – ^{1}H NMR spectrum (600 MHz, $CDCl_3$) of **1** 5
Figure S3 – ^{13}C NMR spectrum (150 MHz, $CDCl_3$) of **1** 5
Figure S4 – ^{13}C NMR spectrum zoomed (150 MHz, $CDCl_3$) of **1** 6
Figure S5 – COSY NMR spectrum (600 MHz, $CDCl_3$) of **1** 6
Figure S6 – HSQC NMR spectrum (600 MHz, $CDCl_3$) of **1** 7
Figure S7 – HMBC NMR spectrum (600 MHz, $CDCl_3$) of **1** 7
Figure S8 – NOESY NMR spectrum (600 MHz, $CDCl_3$) of **1** 8
Figure S9 – ^{1}H NMR spectrum (600 MHz, $(CD_3)_2SO$) of **1** 8
Figure S10 – ^{1}H NMR spectrum zoomed (600 MHz, $(CD_3)_2SO$) of **1** 9
Figure S11 – ^{13}C NMR spectrum (150 MHz, $(CD_3)_2SO$) of **1** 9
Figure S12 – ^{13}C NMR spectrum zoomed (150 MHz, $(CD_3)_2SO$) of **1** 10
Figure S13 – NOESY NMR spectrum (600 MHz, $(CD_3)_2SO$) of **1** 10
Figure S14 – HRESIMS analysis of **1** 11
Figure S15 – ECD analysis of **1** 11
Figure S16 – Structure of **2** 12
Figure S17 – ^{1}H NMR spectrum (600 MHz, $CDCl_3$) of **2** 13
Figure S18 – ^{13}C NMR spectrum (150 MHz, $CDCl_3$) of **2** 13
Figure S19 – ^{13}C NMR spectrum zoomed (150 MHz, $CDCl_3$) of **2** 14
Figure S20 – COSY NMR spectrum (600 MHz, $CDCl_3$) of **2** 14
Figure S21 – HSQC NMR spectrum (600 MHz, $CDCl_3$) of **2** 15
Figure S22 – HMBC NMR spectrum (600 MHz, $CDCl_3$) of **2** 15
Figure S23 – NOESY NMR spectrum (600 MHz, $CDCl_3$) of **2** 16
Figure S24 – HRESIMS analysis of **2** 16
Figure S25 – ECD spectra of **2** 17
Figure S26 – Structure of **3** 18
Figure S27 – ^{1}H NMR spectrum (400 MHz, $CDCl_3$) of **3** 19
Figure S28 – ^{13}C NMR spectrum (100 MHz, $CDCl_3$) of **3** 19
Figure S29 – ^{13}C NMR spectrum zoomned (100 MHz, $CDCl_3$) of **3** 20
Figure S30 – COSY NMR spectrum (400 MHz, $CDCl_3$) of **3** 20
Figure S31 – HSQC NMR spectrum (400 MHz, $CDCl_3$) of **3** 21
Figure S32 – HMBC NMR spectrum (400 MHz, $CDCl_3$) of **3** 21
Figure S33 – NOESY NMR spectrum (400 MHz, $CDCl_3$) of **3** 22
Figure S34 – GC-MS analysis of **3** 22
Figure S35 – ECD analysis of **3** 23
Figure S36 – Structure of **4** 24
Figure S37 – ^{1}H NMR spectrum (600 MHz, $(CD_3)_2SO$) of **4** 25
Figure S38 – ^{13}C NMR spectrum (150 MHz, $(CD_3)_2SO$) of **4** 25
Figure S39 – ^{13}C NMR spectrum zoomed (150 MHz, $(CD_3)_2SO$) of **4** 26
Figure S40 – COSY NMR spectrum (600 MHz, $(CD_3)_2SO$) of **4** 26
Figure S41 – HSQC NMR spectrum (600 MHz, $(CD_3)_2SO$) of **4** 27
Figure S42 – HMBC NMR spectrum (600 MHz, $(CD_3)_2SO$) of **4** 27
Figure S43 – NOESY NMR spectrum (600 MHz, $(CD_3)_2SO$) of **4** 28
Figure S44 – HRESIMS analysis of **4** 28
Figure S45 – ECD analysis of **4** 29
Figure S46 – Structure of **5** 30
Figure S47 – ^{1}H NMR spectrum (600 MHz, $(CD_3)_2SO$) of **5** 31
Figure S48 – ^{1}H NMR spectrum zoomed (600 MHz, $(CD_3)_2SO$) of **5** 31
Figure S49 – ^{13}C NMR spectrum (150 MHz, $(CD_3)_2SO$) of **5** 32
Figure S50 – ^{13}C NMR spectrum zoomed (150 MHz, $(CD_3)_2SO$) of **5** 32
Figure S51 – COSY NMR spectrum (600 MHz, $(CD_3)_2SO$) of **5** 33
Figure S52 – HSQC NMR spectrum (600 MHz, $(CD_3)_2SO$) of **5** 33
Figure S53 – HMBC NMR spectrum (600 MHz, $(CD_3)_2SO$) of **5** 34
Figure S54 – NOESY NMR spectrum (600 MHz, $(CD_3)_2SO$) of **5** 34
Figure S55 – HRESIMS analysis of **5** 35
Figure S56 – ECD analysis of **5** 35
Figure S57 – Structure of **6** 36
Figure S58 – ^{1}H NMR spectrum (400 MHz, $(CH_3)_2SO$) of **6** 37
Figure S59 – ^{13}C NMR spectrum (100 MHz, $(CH_3)_2SO$) of **6** 37
Figure S60 – ^{13}C NMR spectrum zoomed (100 MHz, $(CH_3)_2SO$) of **6** 38
Figure S61 – COSY NMR spectrum (400 MHz, $(CH_3)_2SO$) of **6** 38
Figure S62 – HSQC NMR spectrum (400 MHz, $(CH_3)_2SO$) of **6** 39
Figure S63 – HMBC NMR spectrum (400 MHz, $(CH_3)_2SO$) of **6** 39
Figure S64 – GC-MS analysis of **6** 40
Figure S65 – ECD analysis of **6** 40
Figure S66 – Structure of **7** 41
Figure S67 – ^{1}H NMR spectrum (600 MHz, $CDCl_3$) of **7** 42
Figure S68 – ^{13}C NMR spectrum (150 MHz, $CDCl_3$) of **7** 42
Figure S69 – ^{13}C NMR spectrum zoomed (150 MHz, $CDCl_3$) of **7** 43
Figure S70 – COSY NMR spectrum (600 MHz, $CDCl_3$) of **7** 43

Figure S71 – HSQC NMR spectrum (600 MHz, $CDCl_3$) of **7** 44
Figure S72 – HMBC NMR spectrum (600 MHz, $CDCl_3$) of **7** 44
Figure S73 – NOESY NMR spectrum (600 MHz, $CDCl_3$) of **7** 45
Figure S74 – HREIMS analysis of **7** 45
Figure S75 – ECD analysis of **7** 46
Figure S76 – Structure of **8** 47
Figure S77 – ^{1}H NMR spectrum (600 MHz, $CDCl_3$) of **8** 48
Figure S78 – ^{1}H NMR spectrum zoomed (600 MHz, $CDCl_3$) of **8** 48
Figure S79 – ^{13}C NMR spectrum (150 MHz, $CDCl_3$) of **8** 49
Figure S80 – ^{13}C NMR spectrum zoomed (150 MHz, $CDCl_3$) of **8** 49
Figure S81 – COSY NMR spectrum (600 MHz, $CDCl_3$) of **8** 50
Figure S82 – HSQC NMR spectrum (600 MHz, $CDCl_3$) of **8** 50
Figure S83 – HMBC NMR spectrum (600 MHz, $CDCl_3$) of **8** 51
Figure S84 – NOESY NMR spectrum (600 MHz, $CDCl_3$) of **8** 51
Figure S85 – HRESIMS analysis of **8** 52
Figure S86 – Ellipsoid plot of **8**. Anisotropic displacement parameters were drawn at 50% probability level. 53
Figure S87 – ECD analysis of **8** 53
Figure S88 – Structure of **9** 54
Figure S89 – ^{1}H NMR spectrum (400 MHz, $CDCl_3$) of **9** 55
Figure S90 – ^{13}C NMR spectrum (100 MHz, $CDCl_3$) of **9** 55
Figure S91 – COSY NMR spectrum (400 MHz, $CDCl_3$) of **9** 56
Figure S92 – HSQC NMR spectrum (400 MHz, $CDCl_3$) of **9** 56
Figure S93 – HMBC NMR spectrum (400 MHz, $CDCl_3$) of **9** 57
Figure S94 – NOESY NMR spectrum (400 MHz, $CDCl_3$) of **9** 57
Figure S95 – HRESIMS analysis of **9** 58
Figure S96 – ECD analysis of **9** 58
Figure S97 – Structure of **10** 59
Figure S98 – ^{1}H NMR spectrum (400 MHz, $CDCl_3$) of **10** 60
Figure S99 – ^{13}C NMR spectrum (100 MHz, $CDCl_3$) of **10** 60
Figure S100 – COSY NMR spectrum (400 MHz, $CDCl_3$) of **10** 61
Figure S101 – HSQC NMR spectrum (400 MHz, $CDCl_3$) of **10** 61
Figure S102 – HMBC NMR spectrum (400 MHz, $CDCl_3$) of **10** 62
Figure S103 – NOESY NMR spectrum (400 MHz, $CDCl_3$) of **10** 62
Figure S104 – HRESIMS analysis of **10** 63
Figure S105 – ECD analysis of **10** 63
Figure S106 – Structure of **11** 64
Figure S107 – ^{1}H NMR spectrum (400 MHz, $CDCl_3$) of **11** 65
Figure S108 – ^{13}C NMR spectrum (100 MHz, $CDCl_3$) of **11** 65
Figure S109 – ^{13}C NMR spectrum zoomed (100 MHz, $CDCl_3$) of **11** 66
Figure S110 – COSY NMR spectrum (400 MHz, $CDCl_3$) of **11** 66
Figure S111 – HSQC NMR spectrum (400 MHz, $CDCl_3$) of **11** 67
Figure S112 – HMBC NMR spectrum (400 MHz, $CDCl_3$) of **11** 67
Figure S113 – NOESY NMR spectrum (400 MHz, $CDCl_3$) of **11** 68
Figure S114 – HRESIMS analysis of **11** 68
Figure S115 – ECD analysis of **11** 69
Figure S116 – Structure of **12** 70
Figure S117 – ^{1}H NMR spectrum (600 MHz, $CDCl_3$) of **12** 71
Figure S118 – ^{13}C NMR spectrum (150 MHz, $CDCl_3$) of **12** 71
Figure S119 – ^{13}C NMR spectrum zoomed (150 MHz, $CDCl_3$) of **12** 72
Figure S120 – COSY NMR spectrum (600 MHz, $CDCl_3$) of **12** 72
Figure S121 – HSQC NMR spectrum (600 MHz, $CDCl_3$) of **12** 73
Figure S122 – HMBC NMR spectrum (600 MHz, $CDCl_3$) of **12** 73
Figure S123 – HREIMS analysis of **12** 74
Figure S124 – ECD analysis of **12** 74

Table S1 – NMR Data for Anthoteibinene F (**1**) (600 (^{1}H) and 150 (^{13}C) MHz, ^{a}$CDCl_3$, ^{b}$(CD_3)_2SO$)

pos	$^a\delta_C$, type	$^b\delta_C$, type	$^a\delta_H$ (*J* in Hz)	$^b\delta_H$ (*J* in Hz)	^{b}gCOSY	^{b}gHMBC	Key ^{a}NOESY	Key ^{b}NOESY
**1**	162.0, C	162.4, C						
**2**	102.0, C	102.5, C						
**3a**	43.4, CH_2	43.4, CH_2	2.50, d (17.5)	2.58, d (17.1)	3b	1, 2, 4, 5, 15		
**3b**			2.71, d (17.5)	2.30, d (17.1)	3a, 5, 15	2, 4, 5		
**4**	131.1, C	131.2, C						
**5**	120.3, CH	120.0, CH	5.58, br s	5.55, s	3b, 6, 15	1, 3, 6, 15		
**6**	35.5, CH	35.2, CH	3.00, dd (2.1, 9.1)	2.88, br d (2.2, 9.2)	7, 15	1, 5, 7	8b, 13	8b, 11, 13, OH
**7**	45.3, CH	44.7, CH	1.19, dddd (2.1, 2.1, 9.1, 12.4)	1.12, dddd (2.2, 2.2, 9.2, 12.4)	6, 8a, 8b, 11	5, 6, 11, 12, 13		8a, 9b
**8a**	21.1, CH_2	20.9, CH_2	1.91, dddd (2.1, 2.1, 5.0, 13.4)	1.81, dddd (2.0, 2.0, 5.5, 13.2)	7, 8b, 9b	6, 7, 10, 11	11, 12	12
**8b**			1.35, dddd (5.0, 12.4, 12.4, 13.4)	1.23, dddd (5.3, 12.2, 12.2, 12.9)	7, 8a, 9a, 9b	6, 7, 9	6, 13	13
**9a**	20.3, CH_2	19.8, CH_2	2.42, ddd (2.1, 5.0, 18.0)	2.25 ddd (2.5, 2.5, 17.7)	8b, 9b	1, 7, 10		
**9b**			2.09, o/l*	1.99 ddd, (4.5, 12.1, 12.1)	8a, 8b, 9a	1, 10		
**10**	127.1, C	125.1, C						
**11**	26.8, CH	26.3, CH	2.12, o/l*	2.10, d sept (2.2, 6.7)	7, 12, 13	6, 7, 12, 13		
**12**	21.6, CH_3	21.4, CH_3	1.03, d (6.9)	0.98, d (6.9)	11	7, 11, 13	8a	8a
**13**	15.8, CH_3	15.8, CH_3	0.89, d (6.9)	0.84, d (6.9)	11	7, 11, 12	8b	8b
**14**	170.2, C	169.9, C						
**15**	23.5, CH_3	23.2, CH_3	1.77, s	1.67, s	3b, 5, 6	3, 4, 5		
**OH**				7.30, d (0.7)		1, 2, 3		6

*Overlapping ^{1}H NMR signals, 2D assignments based on proximity likelihood

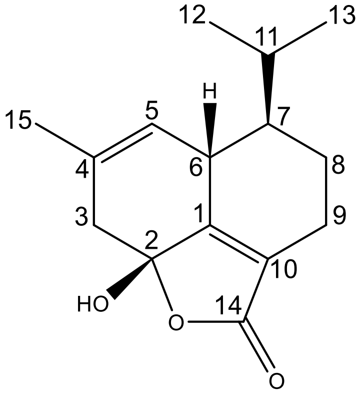

Figure S1 – Structure of **1**

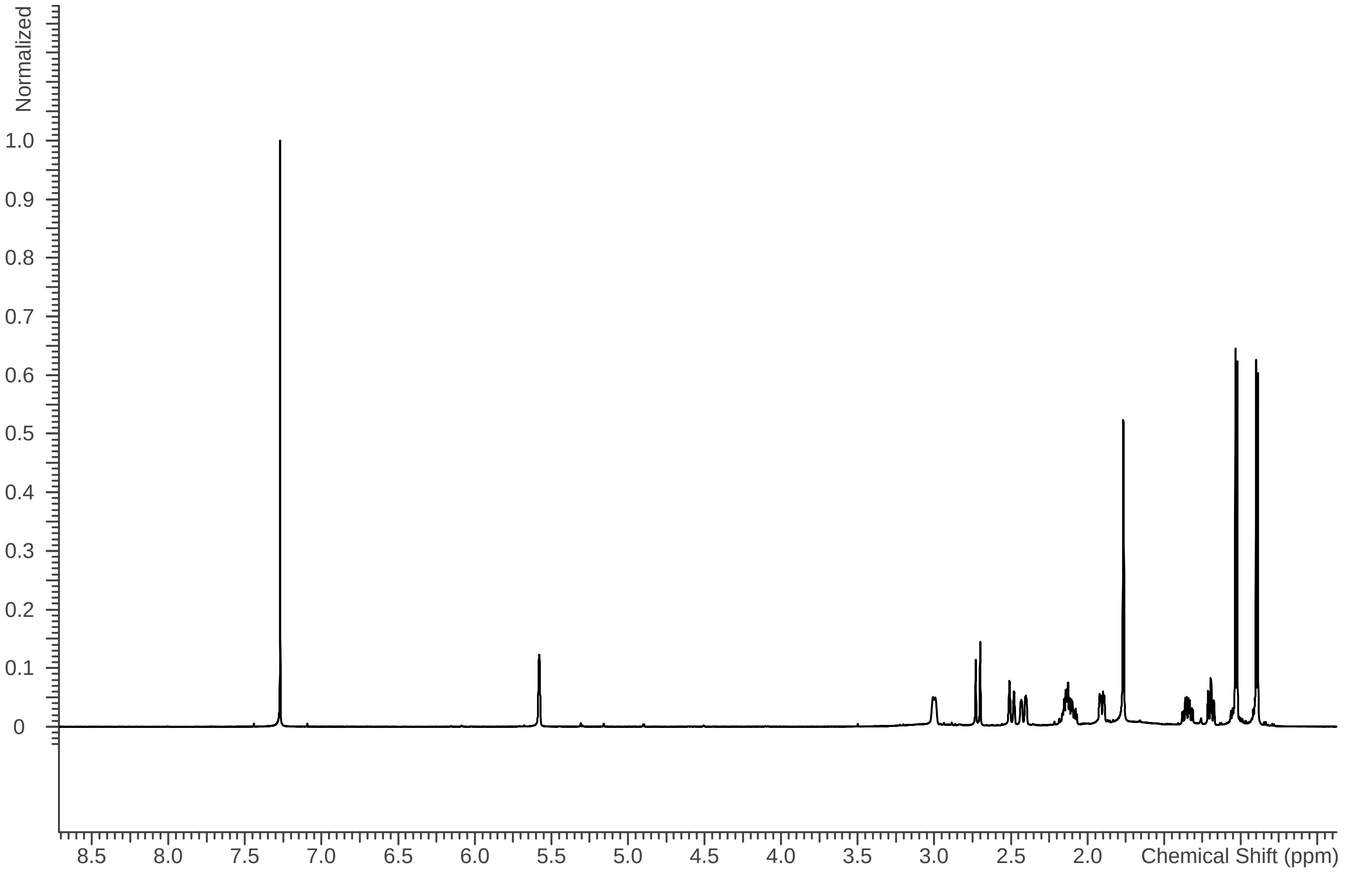

Figure S2 – ^{1}H NMR spectrum (600 MHz, $CDCl_3$) of **1**

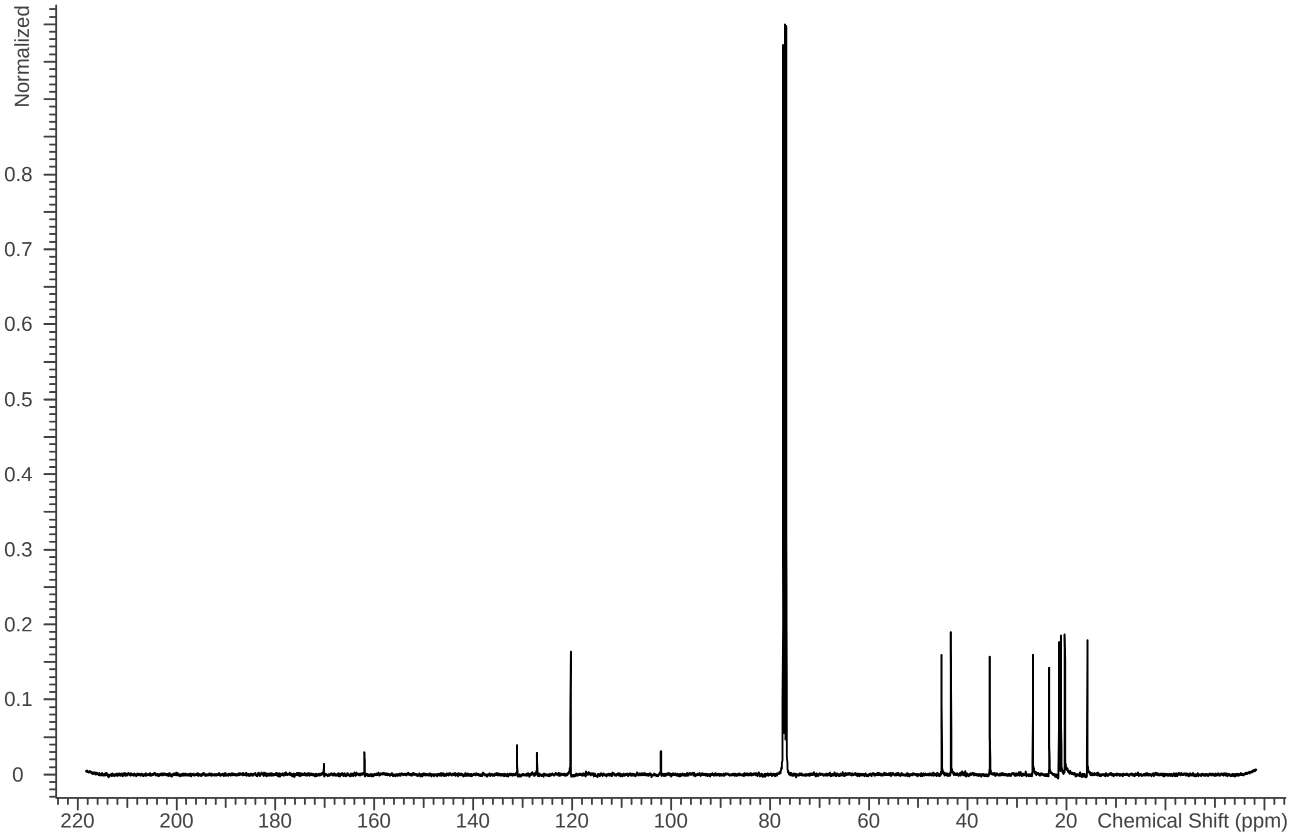

Figure S3 – ^{13}C NMR spectrum (150 MHz, $CDCl_3$) of **1**

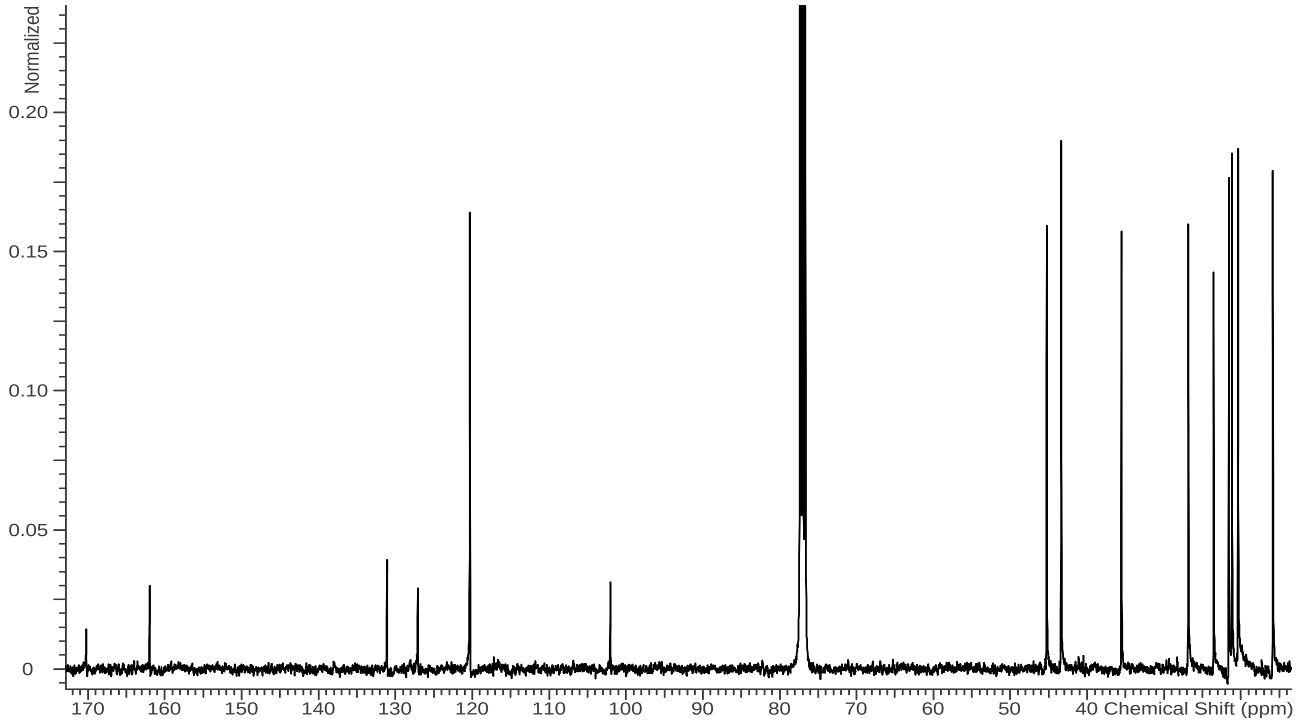

Figure S4 – ^{13}C NMR spectrum zoomed (150 MHz, $CDCl_3$) of **1**

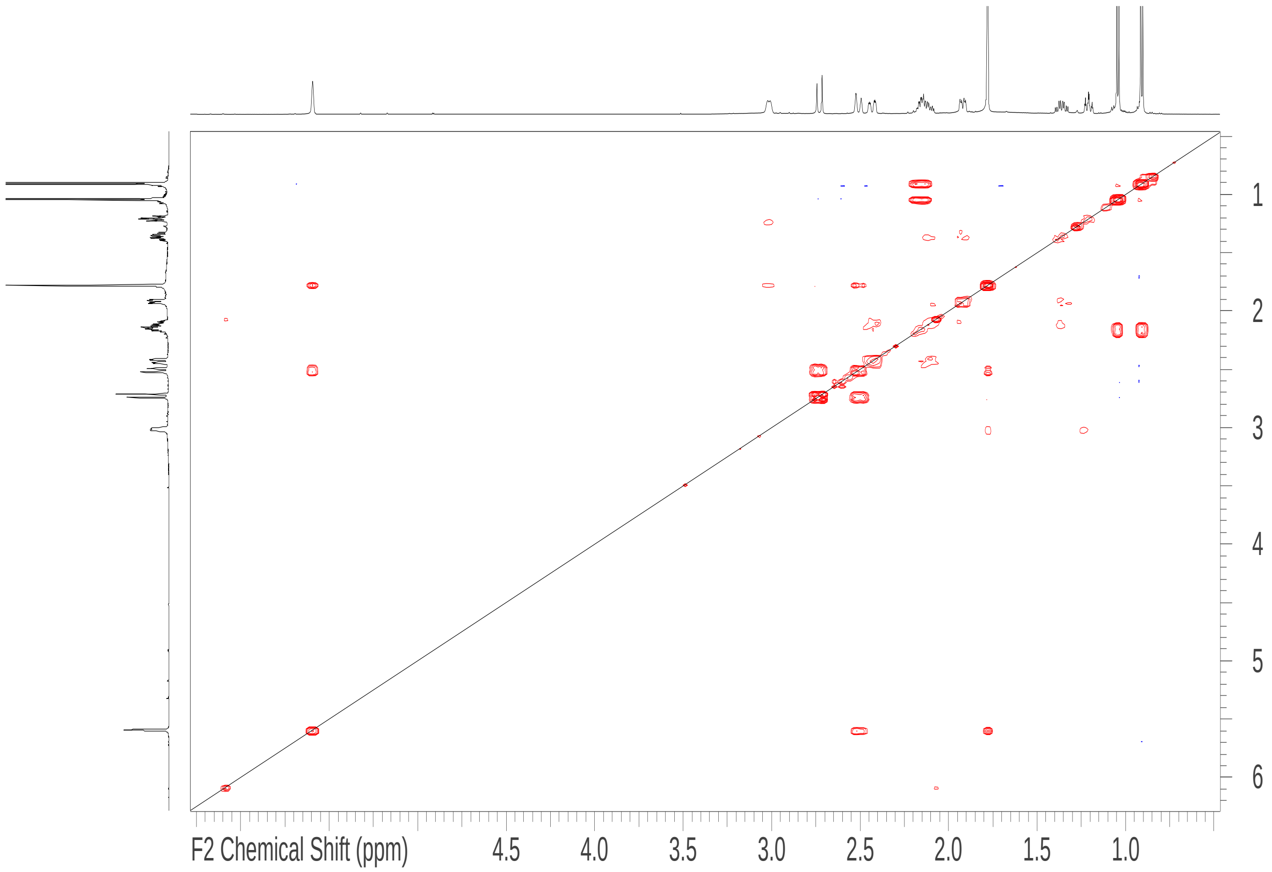

Figure S5 – COSY NMR spectrum (600 MHz, $CDCl_3$) of **1**

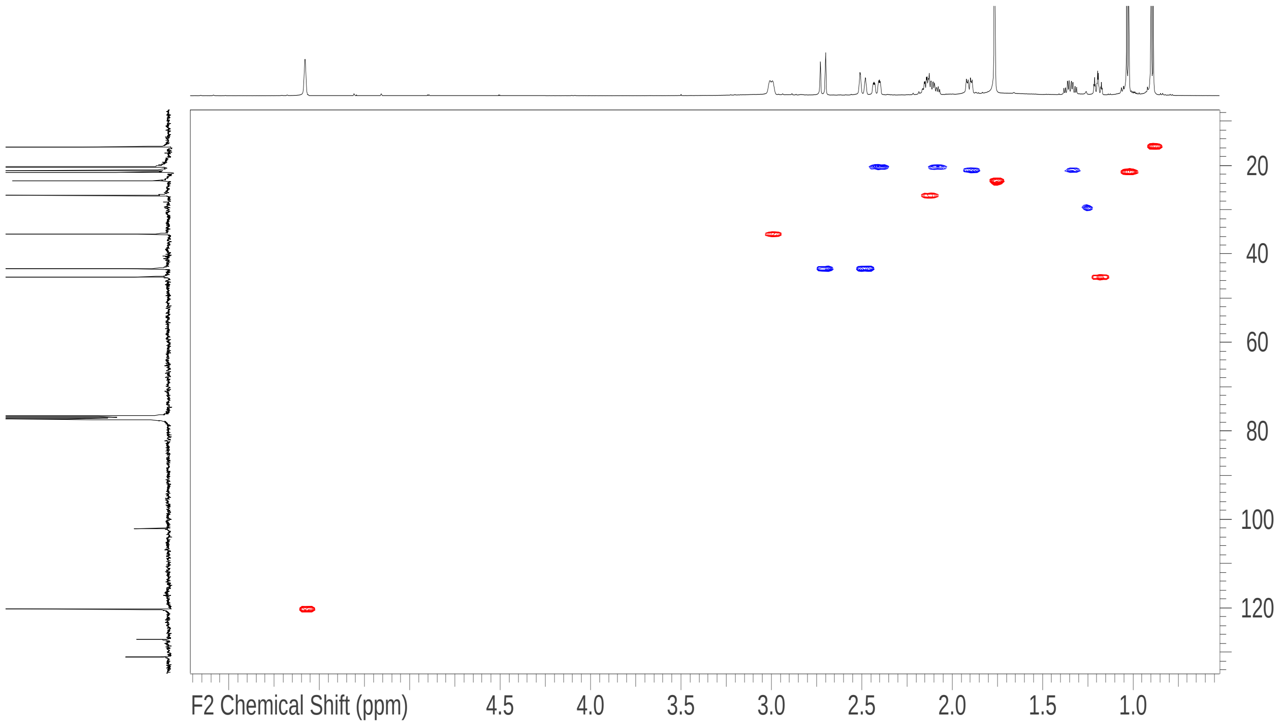

Figure S6 – HSQC NMR spectrum (600 MHz, $CDCl_3$) of **1**

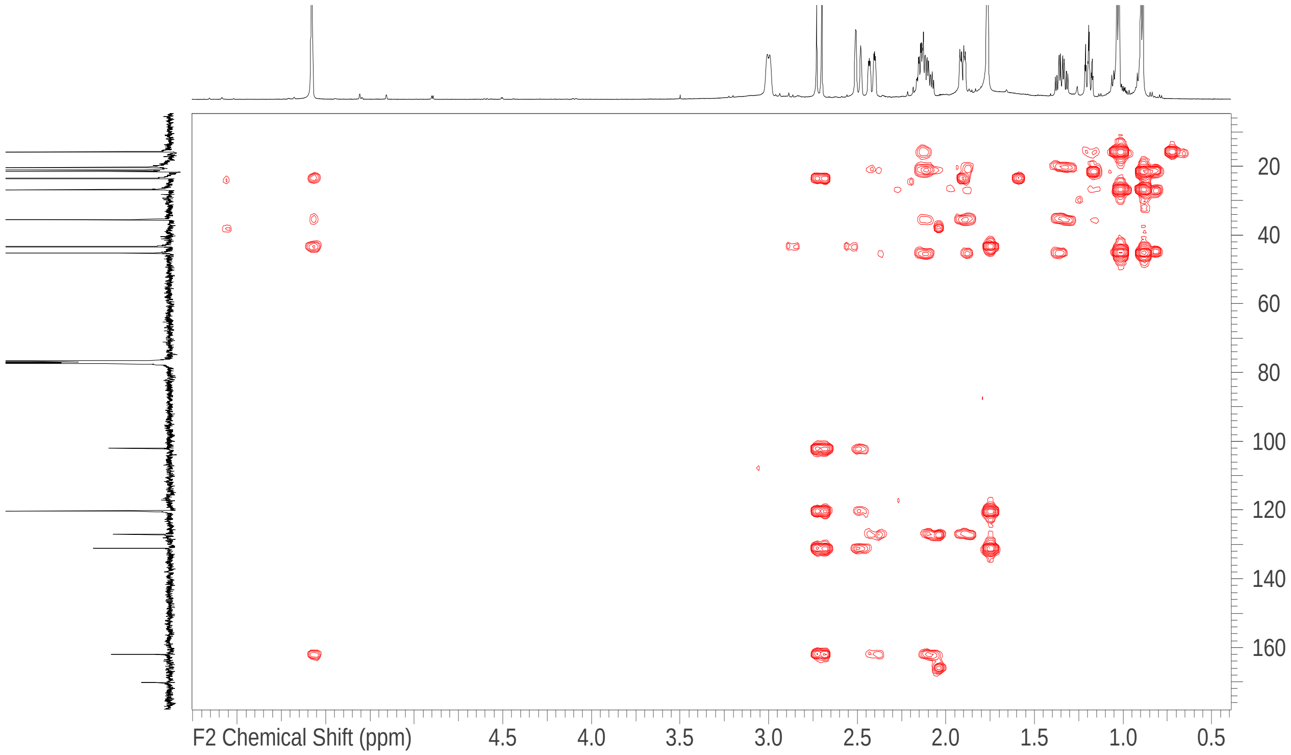

Figure S7 – HMBC NMR spectrum (600 MHz, $CDCl_3$) of **1**

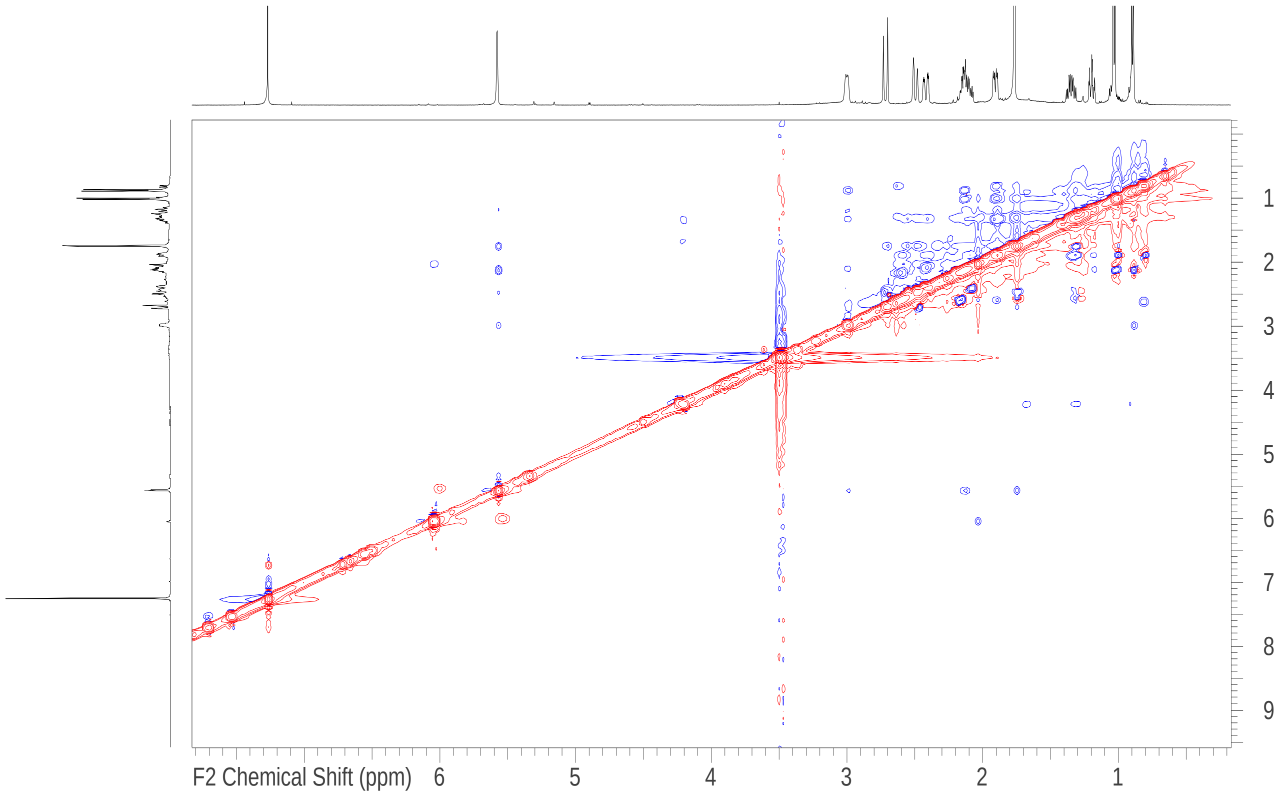

Figure S8 – NOESY NMR spectrum (600 MHz, $CDCl_3$) of **1**

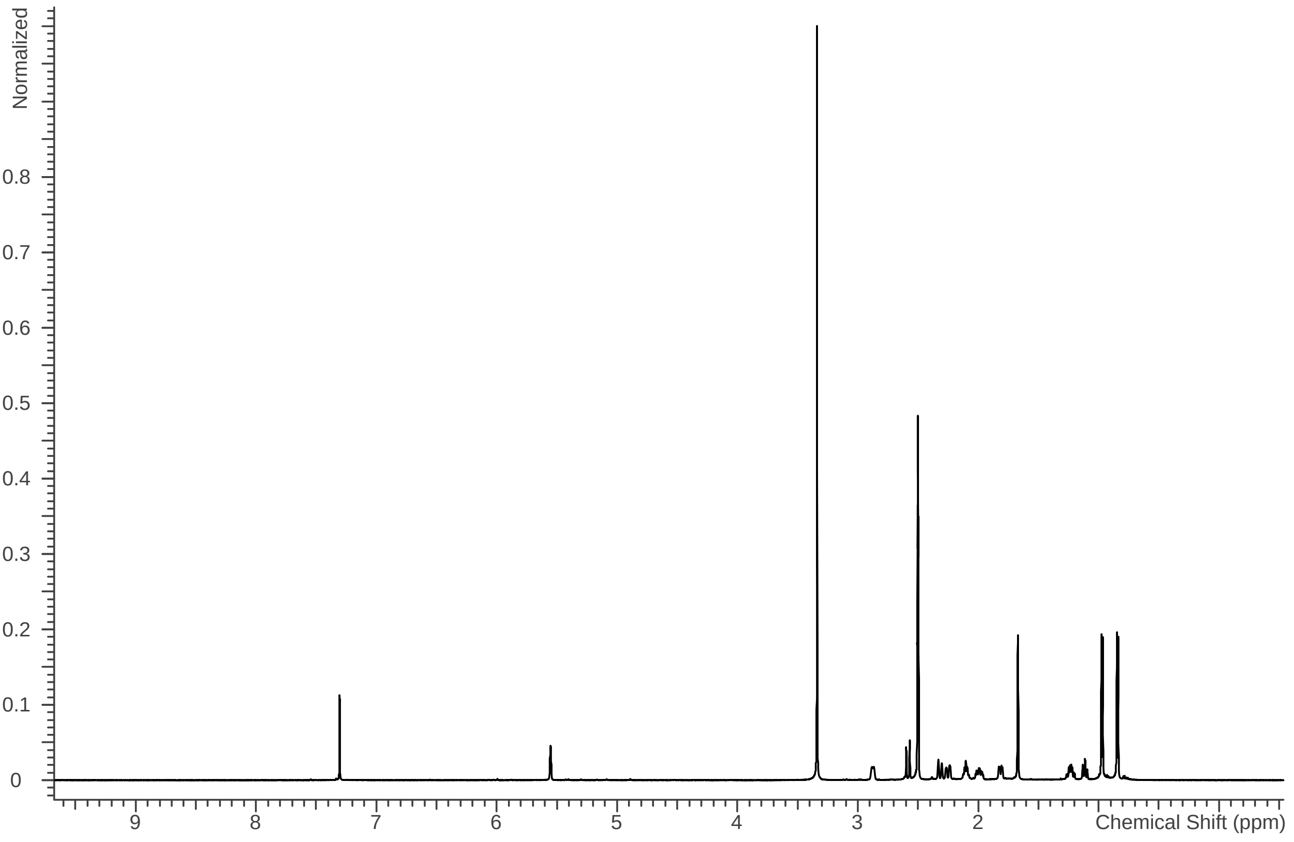

Figure S9 – ^1H NMR spectrum (600 MHz, $(CD_3)_2SO$) of **1**

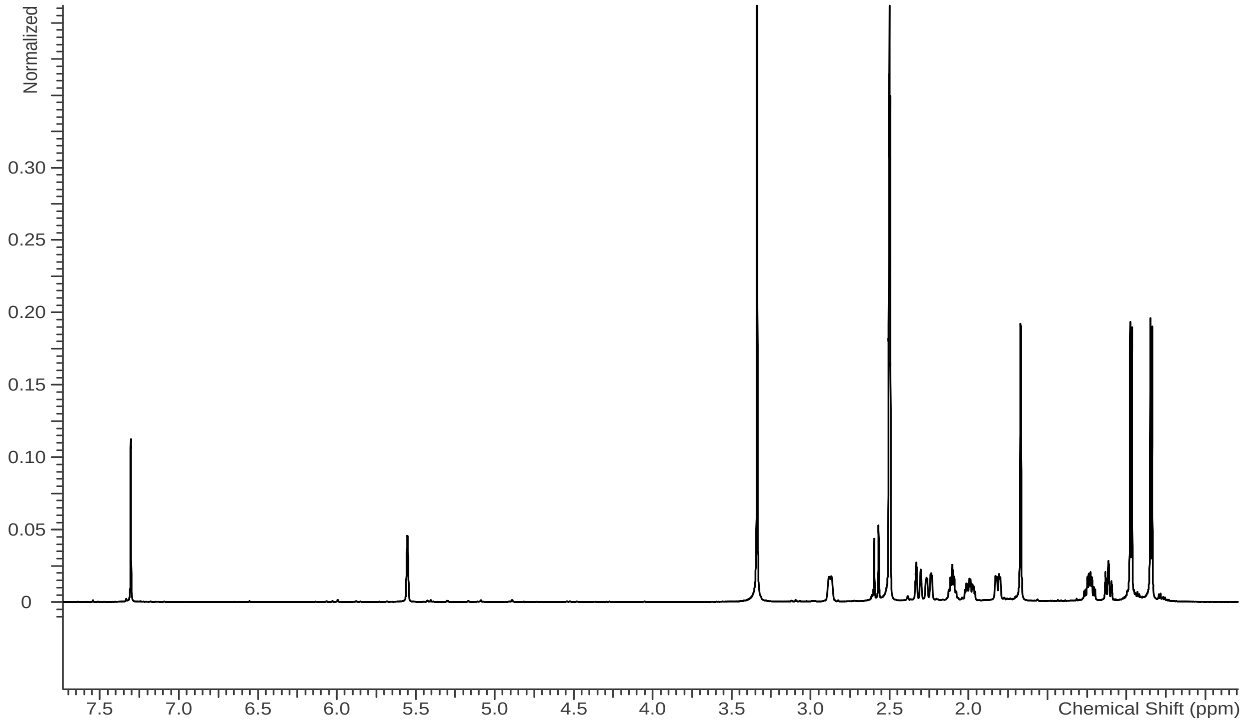

Figure S10 – ^{1}H NMR spectrum zoomed (600 MHz, $(CD_3)_2SO$) of **1**

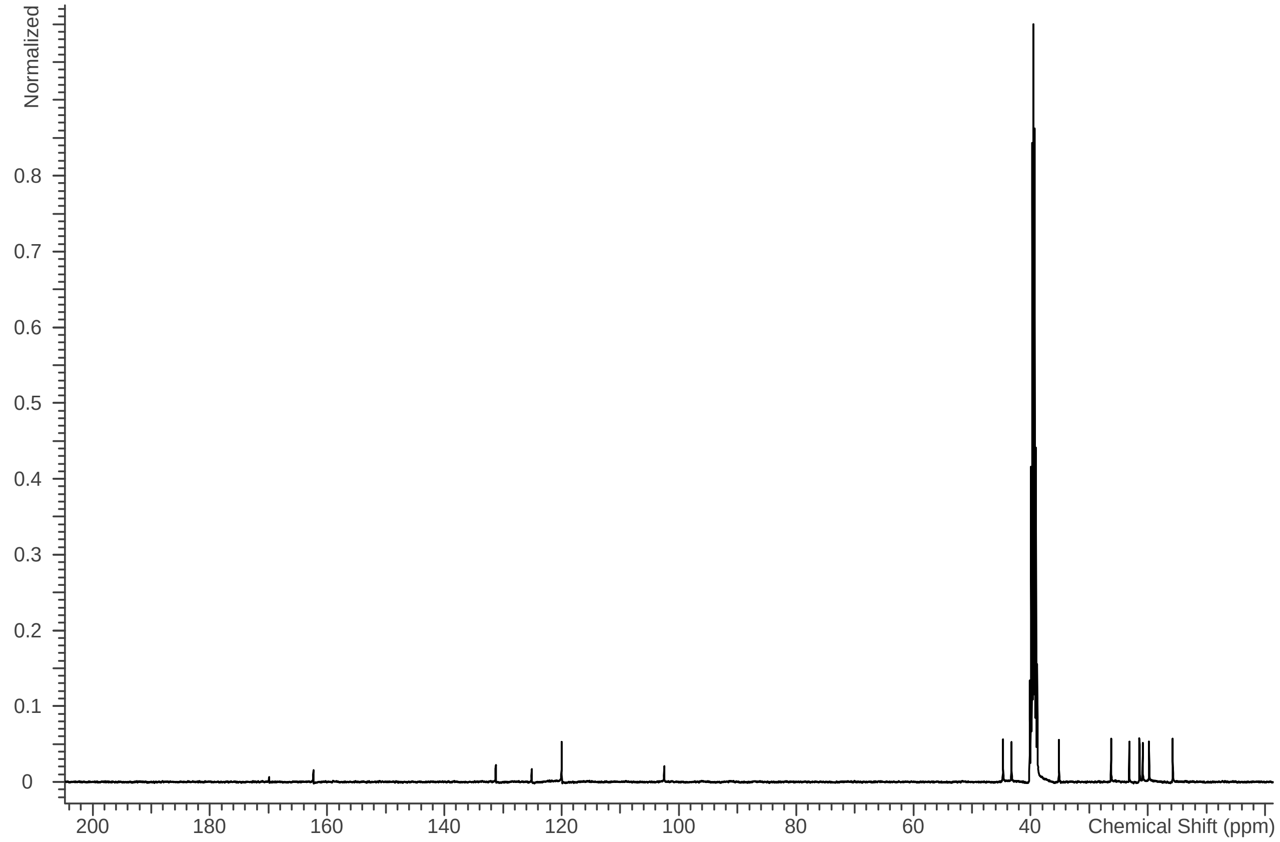

Figure S11 – ^{13}C NMR spectrum (150 MHz, $(CD_3)_2SO$) of **1**

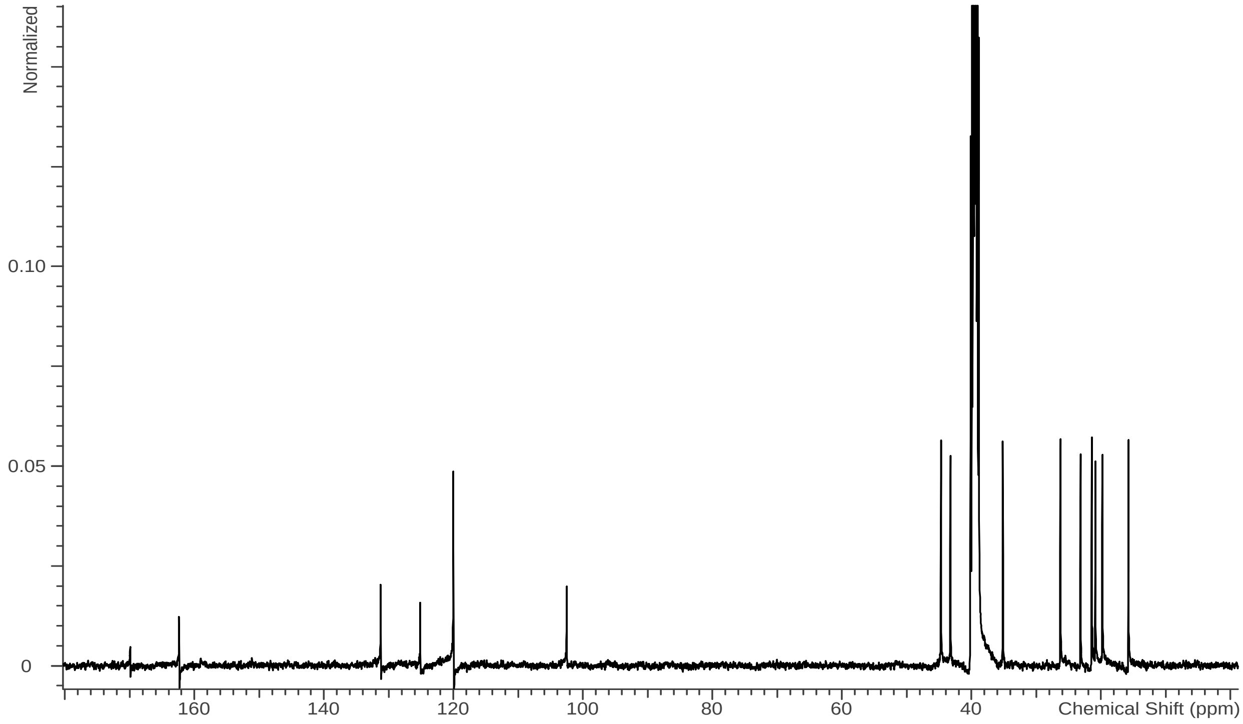

Figure S12 – ^{13}C NMR spectrum zoomed (150 MHz, $(CD_3)_2SO$) of **1**

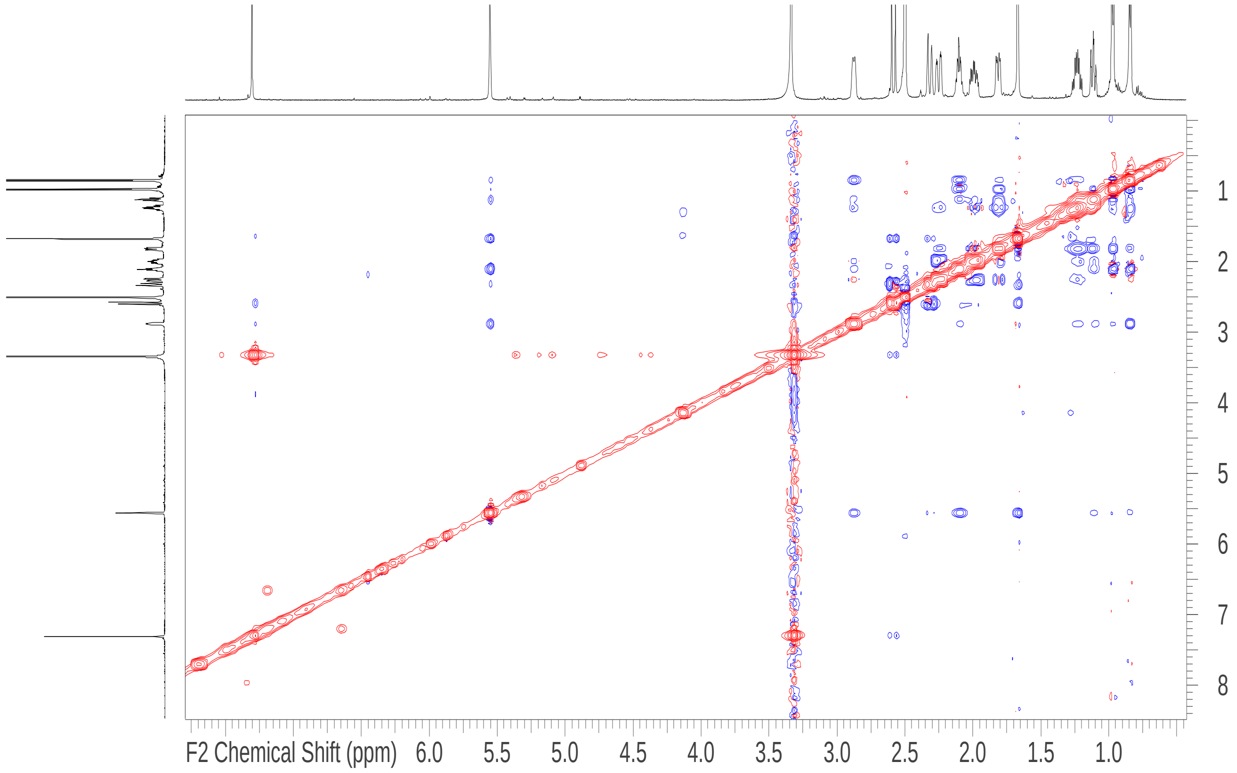

Figure S13 – NOESY NMR spectrum (600 MHz, $(CD_3)_2SO$) of **1**

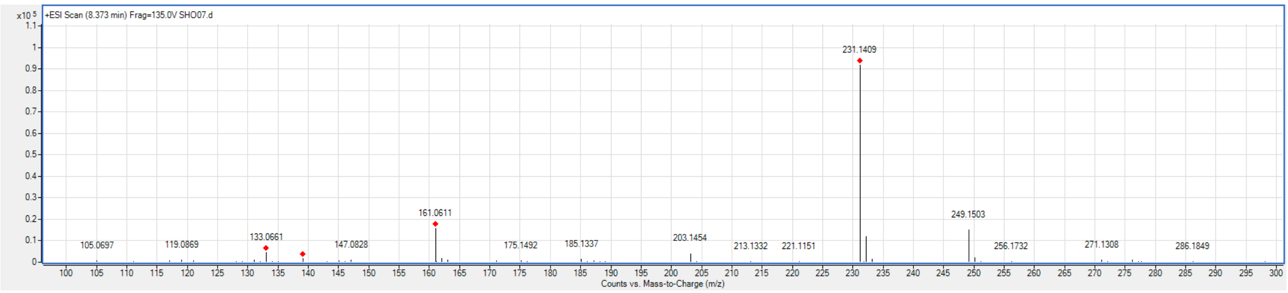

Figure S14 – HRESIMS analysis of **1**

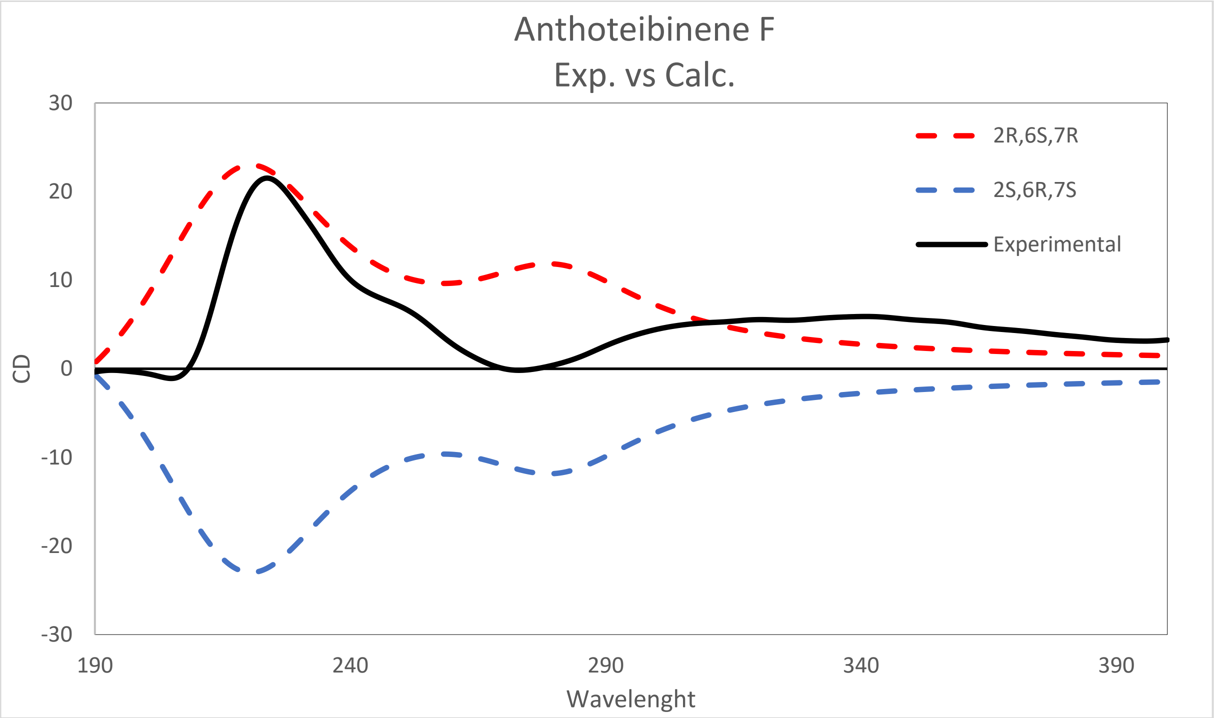

Figure S15 – ECD analysis of **1**

Table S2 – NMR Data for Anthoteibinene G (**2**) (600 (^{1}H) and 150 (^{13}C) MHz, $CDCl_3$)

pos	δC, type	δH	gCOSY	gHMBC	Key NOESY
**1**	160.6, C				
**2**	105.4, C				
**3a**	42.5, CH_2	2.73, d (17.5)	3b	1, 2, 4, 5, 6, 15	
**3b**		2.41, d (17.5)	3a, 5, 15	2, 4	
**4**	131.4, C				
**5**	120.2, CH	5.55, s	3b, 6, 15	1, 3, 15	
**6**	36.1, CH	2.86, dd (2.1, 9.1)	5, 7, 15		13, 8b
**7**	45.5, CH	1.20, dddd (2.1, 2.1, 9.1, 12.4)	6, 8a, 8b, 11	5, 6, 8, 11, 12, 13	
**8a**	21.3, CH_2	1.93, dddd (2.1, 2.1, 5.0, 13.4)	7, 8b, 9b	6, 7, 9, 10, 11	12
**8b**		1.35, dddd (5.0, 12.4, 12.4, 13.4)	7, 8a, 9a, 9b	6, 7, 9	6, 13
**9a**	20.6, CH_2	2.48, ddd (2.1, 5.0, 18.0)	8b, 9b	1, 7, 10, 14	
**9b**		2.13, o/l*	9a, 8a, 8b	1, 7, 8, 10	
**10**	129.4, C				
**11**	26.8, CH	2.13, o/l*	6, 7, 12, 13	6, 7, 8, 12, 13	
**12**	21.6, CH_3	1.03, d (6.9)	11, 13	7, 11, 13	7
**13**	15.9, CH_3	0.90, d (6.9)	11, 12	7, 11, 12	6, 8b
**14**	170.1, C				
**15**	23.5, CH_2	1.75, s	3b, 5, 6	3, 4, 5	
**16**	50.9, CH_3	3.22, s		2	

*Overlapping ^{1}H NMR signals, 2D assignments based on proximity likelihood

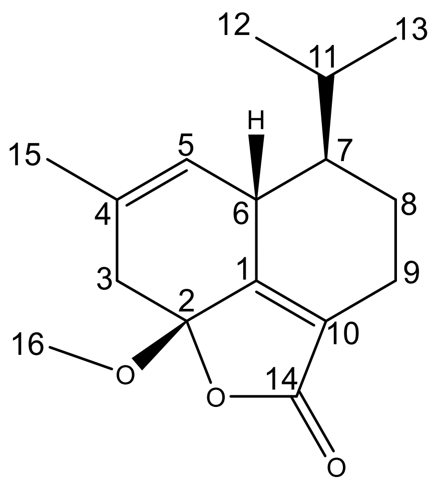

Figure S16 – Structure of **2**

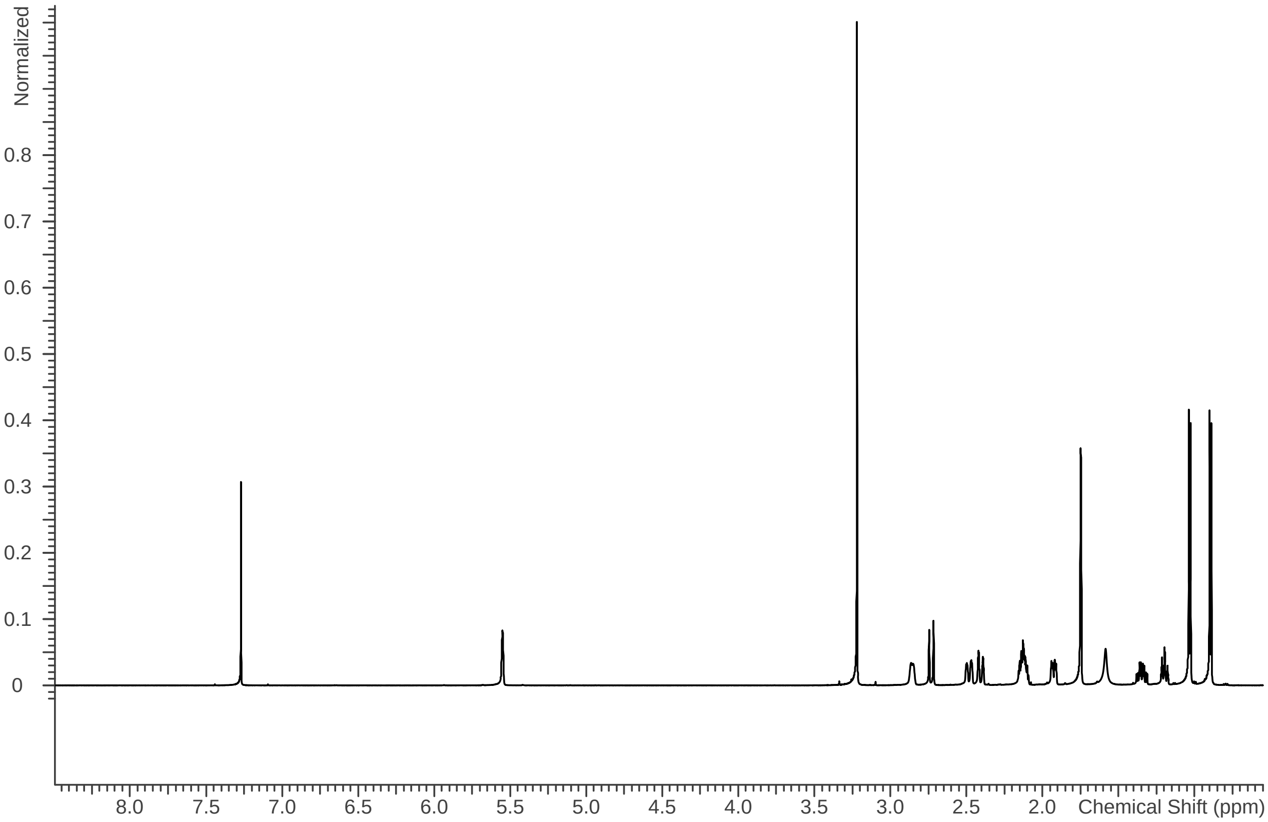

Figure S17 – ^{1}H NMR spectrum (600 MHz, $CDCl_3$) of **2**

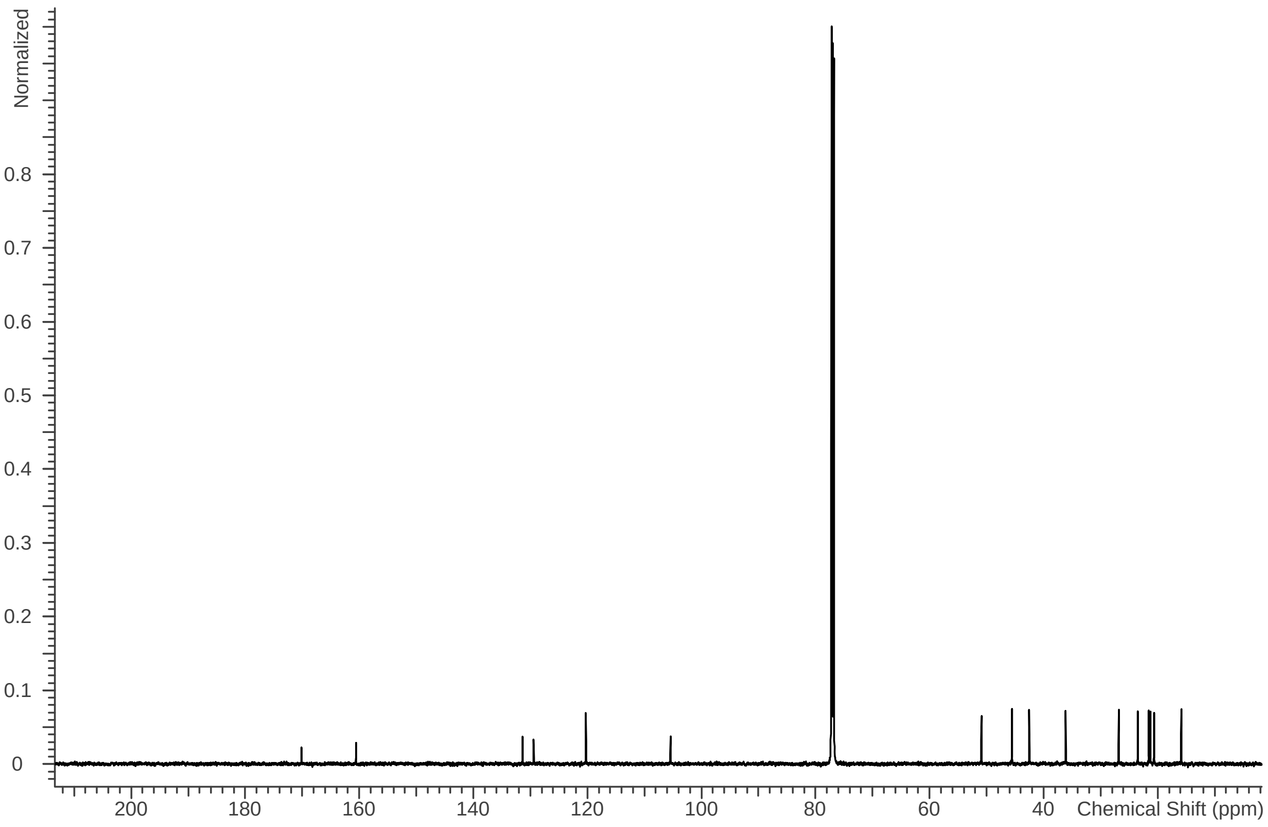

Figure S18 – ^{13}C NMR spectrum (150 MHz, $CDCl_3$) of **2**

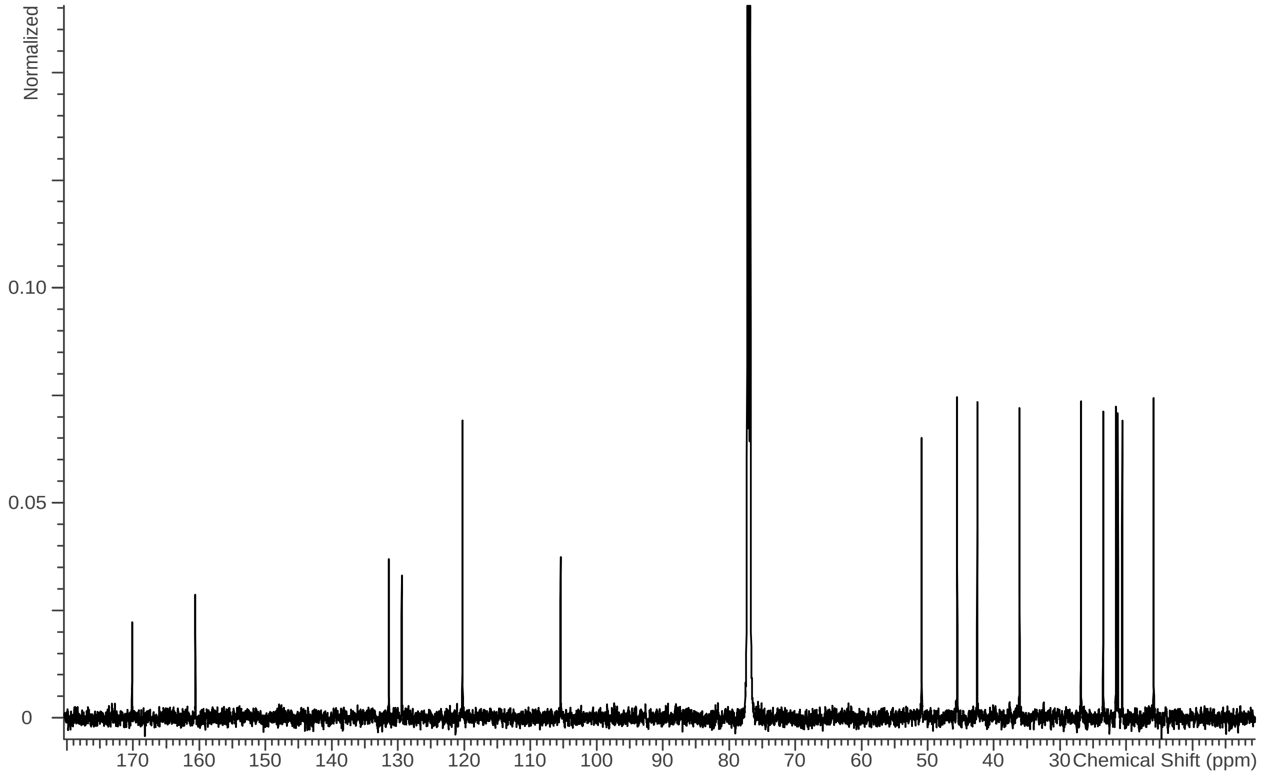

Figure S19 – ^{13}C NMR spectrum zoomed (150 MHz, $CDCl_3$) of **2**

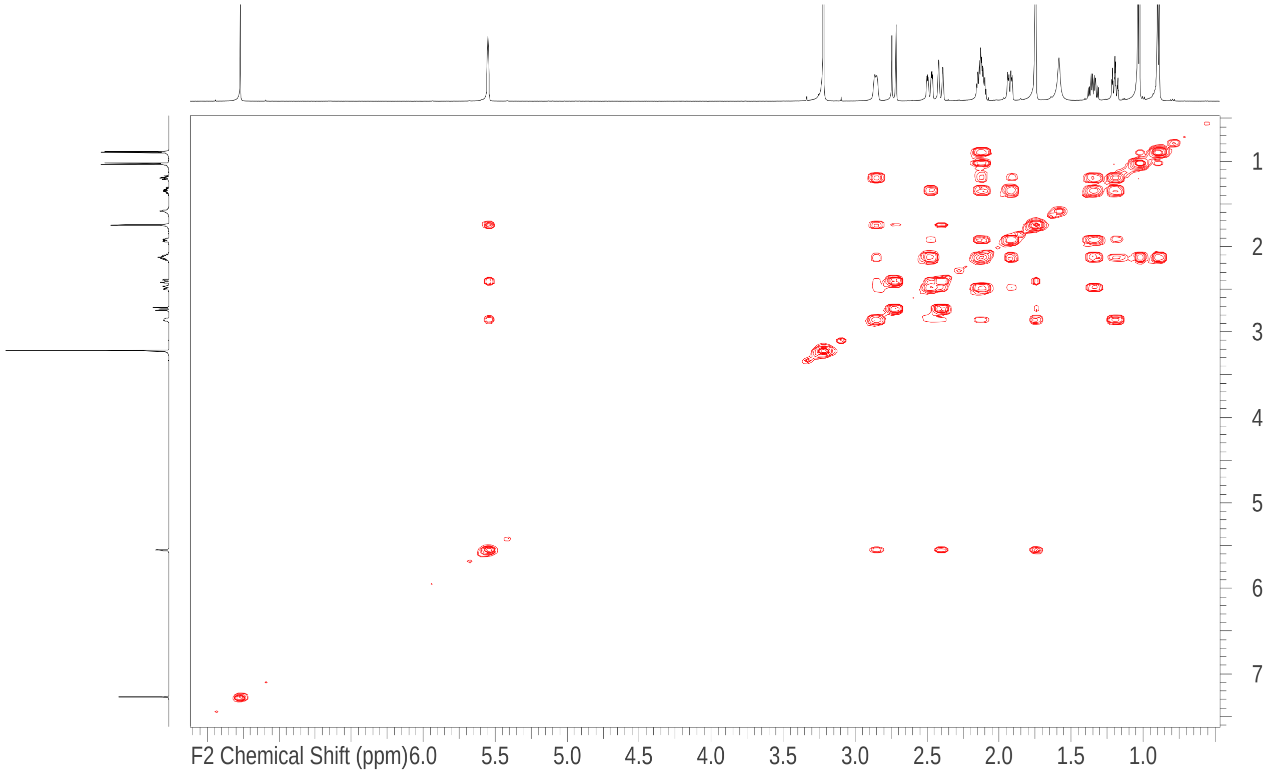

Figure S20 – COSY NMR spectrum (600 MHz, $CDCl_3$) of **2**

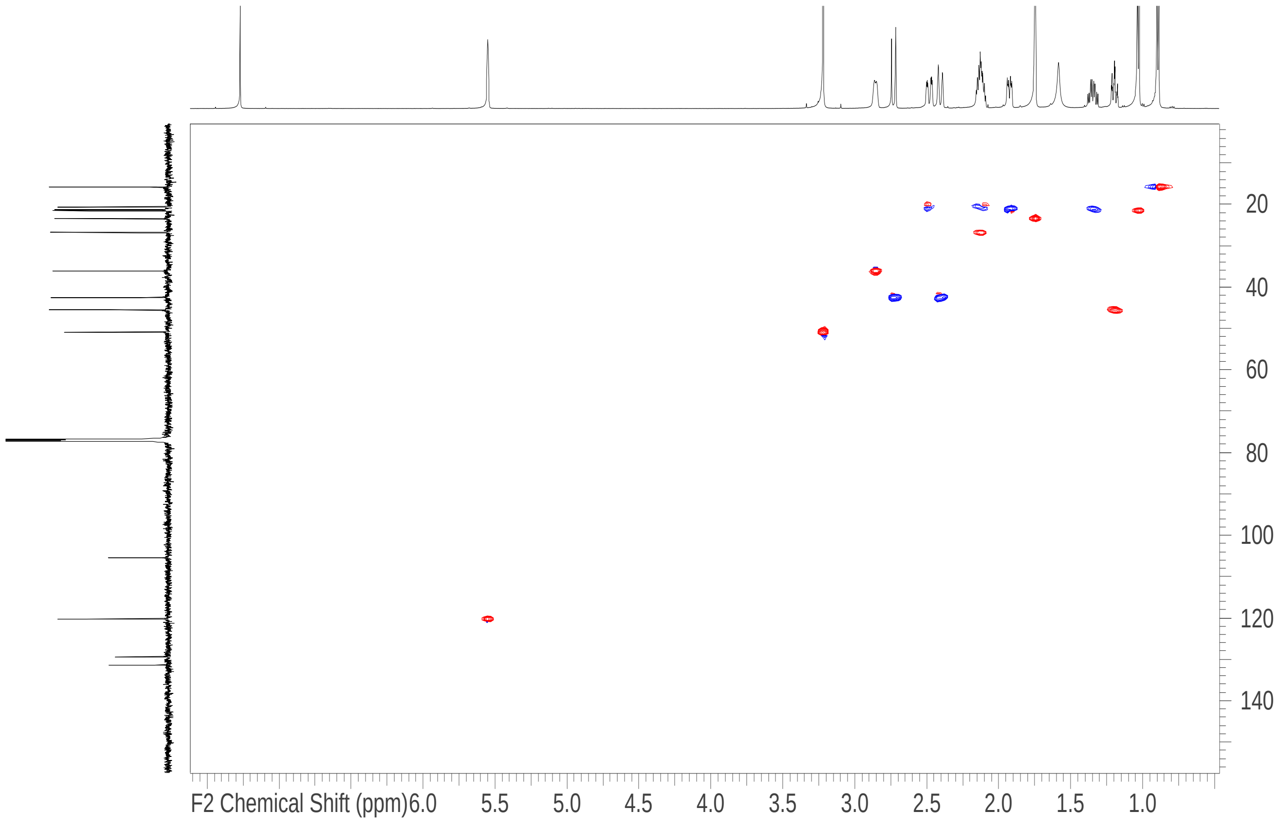

Figure S21 – HSQC NMR spectrum (600 MHz, $CDCl_3$) of **2**

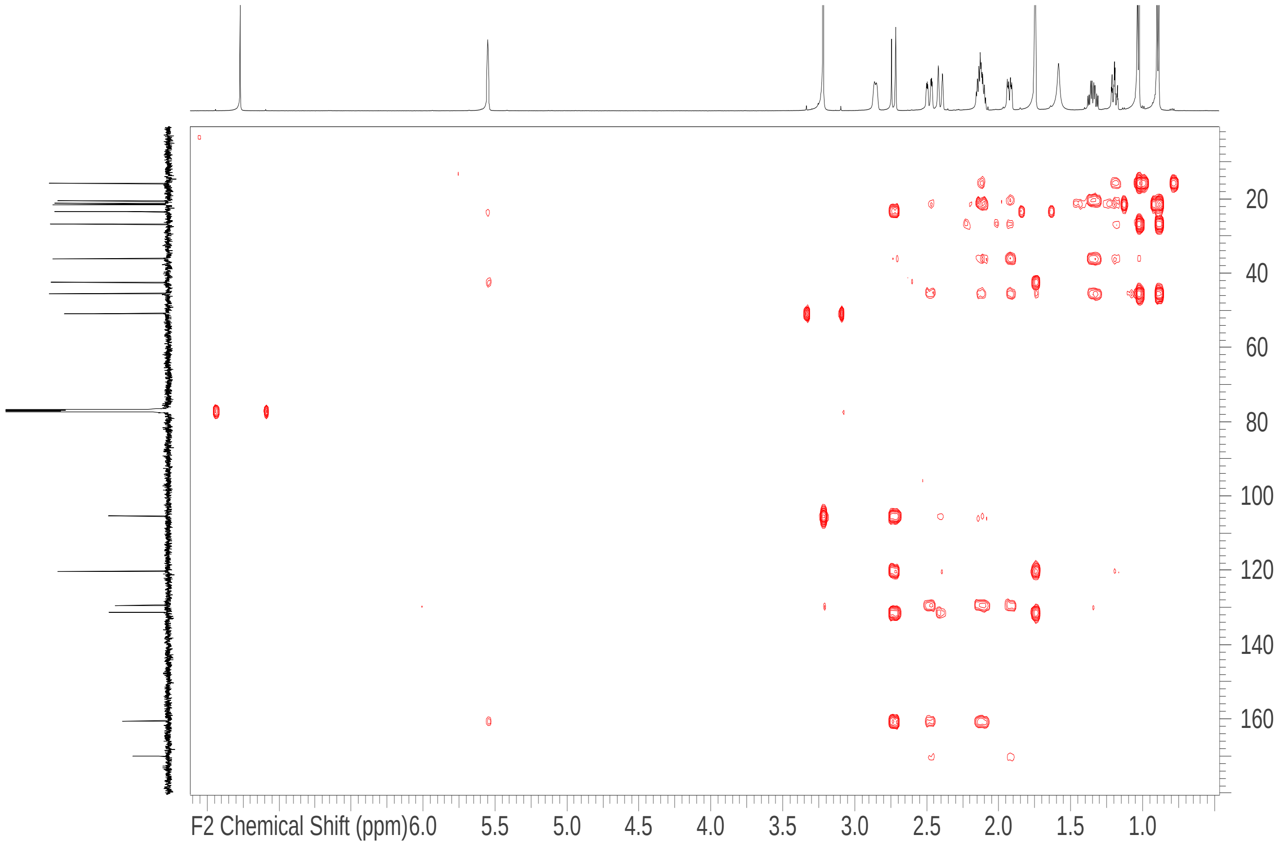

Figure S22 – HMBC NMR spectrum (600 MHz, $CDCl_3$) of **2**

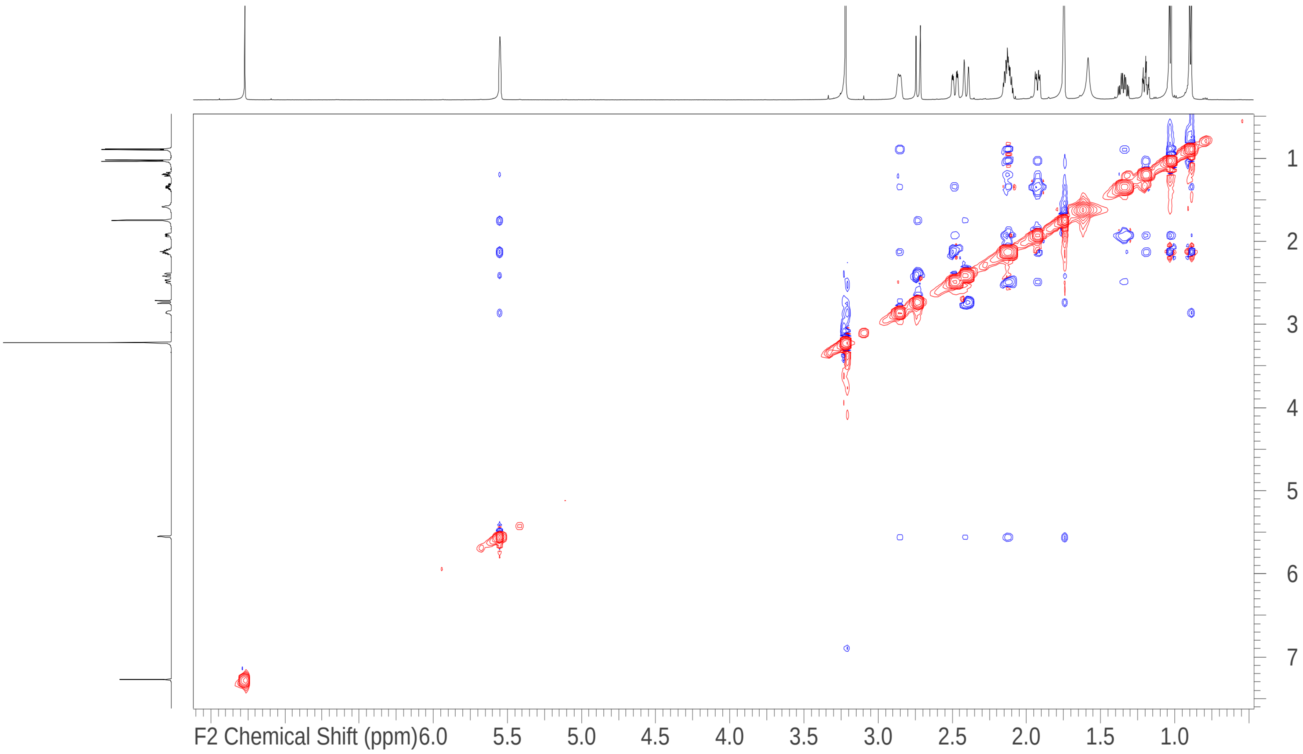

Figure S23 – NOESY NMR spectrum (600 MHz, $CDCl_3$) of **2**

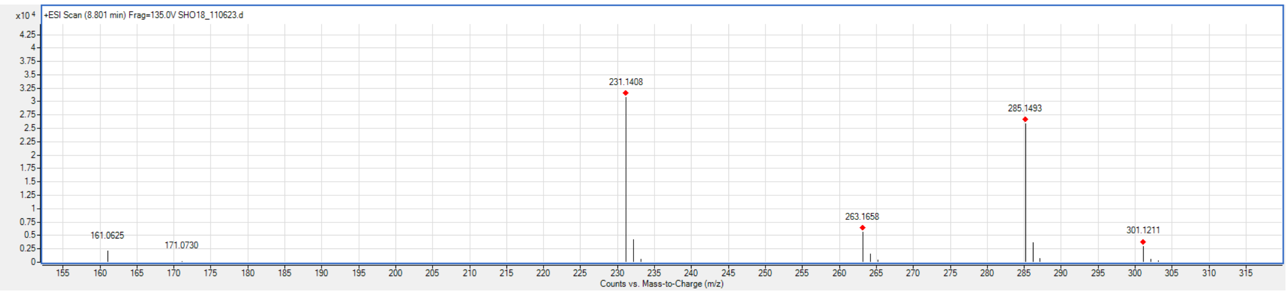

Figure S24 – HRESIMS analysis of **2**

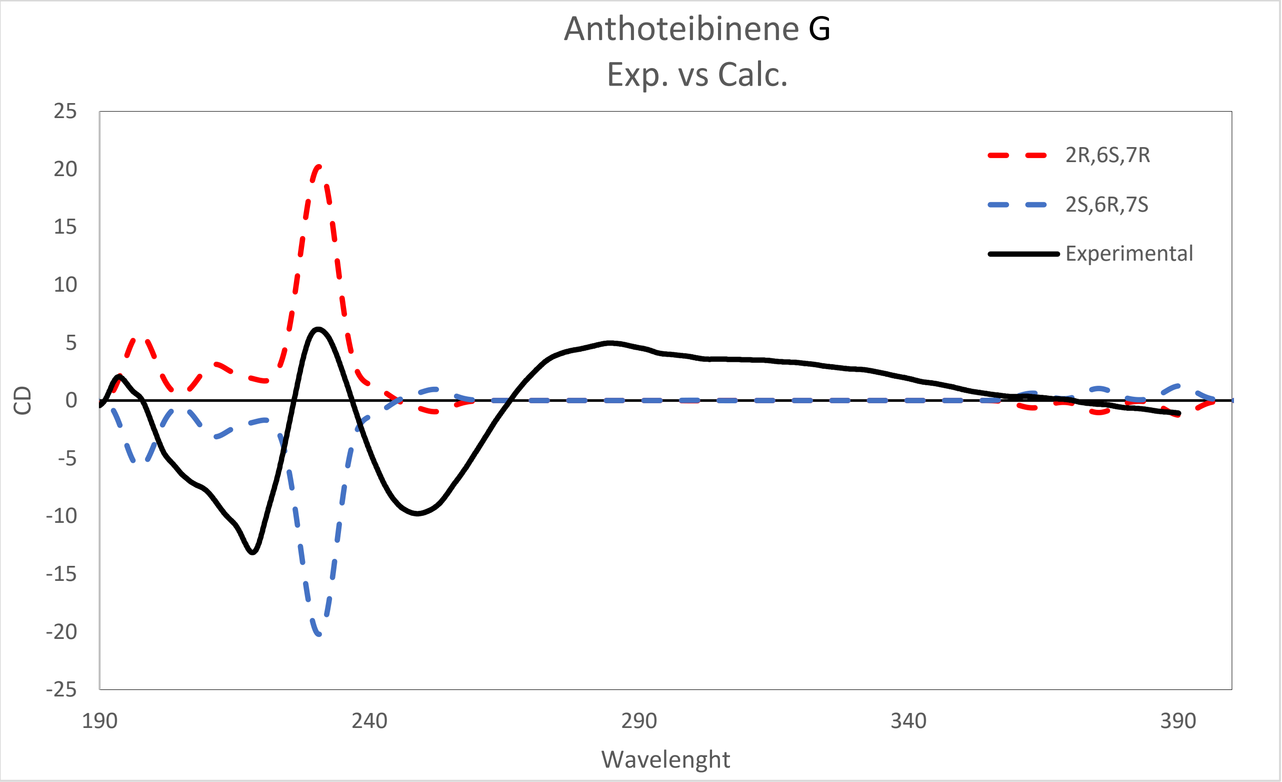

Figure S25 – ECD spectra of **2**

Table S3 – NMR Data for Anthoteibinene H (**3**) (400 (^{1}H) and 100 (^{13}C) MHz, $CDCl_3$).

pos	δ_C, type	δ_H (*J* in Hz)	gCOSY	gHMBC	Key NOESY
**1**	120.4, C				
**2**	153.4, C				
**3**	113.7, CH	6.17, s	5, 15	1, 2, 5, 15	
**4**	135.9, C				
**5**	120.8, CH	6.60, s	3, 7, 15	1, 3, 7,15	
**6**	141.7, C				
**7**	43.3, CH	2.64, ddd (5.0, 6.5, 6.4)	5, 8a, 8b, 11	5, 6, 11, 13	9a, 12
**8a**	21.2, CH_2	1.94, dddd (3.0, 5.0, 8.5, 13.0)	7, 8b		12
**8b**		1.56, dddd (2.8, 6.5, 9.8, 13.0)	7, 8a		10, 13
**9a**	24.8, CH_2	1.74, dddd (3.0, 6.5, 9.8, 13.0)	9a, 10		7
**9b**		2.14, o/l*	9a, 10	7	
**10**	37.4, CH	4.05, t (7.0)	9a, 9b	1, 2, 6, 8, 9, 14	8b
**11**	30.7, CH	2.21, octet (6.4)	7, 12, 13	6, 7, 12, 13	
**12**	21.7, CH_3	1.02, d (6.8)	11	7, 11, 13	7, 8a
**13**	18.0, CH_3	0.76, d (6.8)	11	7, 11, 12	8b
**14**	177.8, C				
**15**	21.2, CH_3	2.13, s	3, 5	3, 4, 5	

*Overlapping ^{1}H NMR signals, 2D assignments based on proximity likelihood

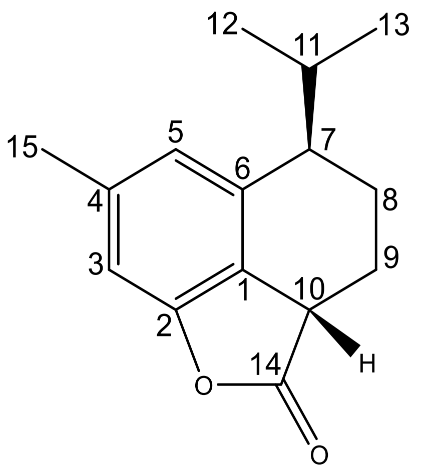

Figure S26 – Structure of **3**

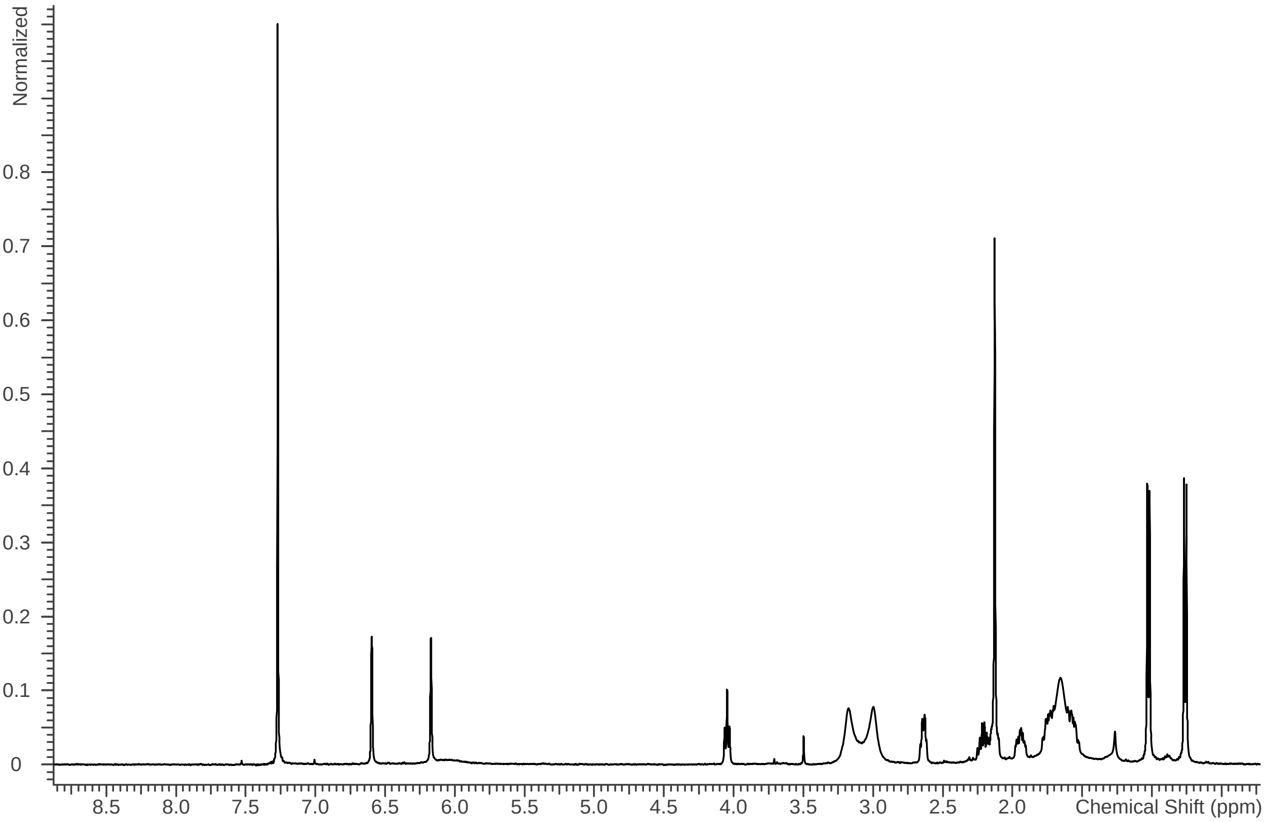

Figure S27 – ^{1}H NMR spectrum (400 MHz, $CDCl_3$) of **3**

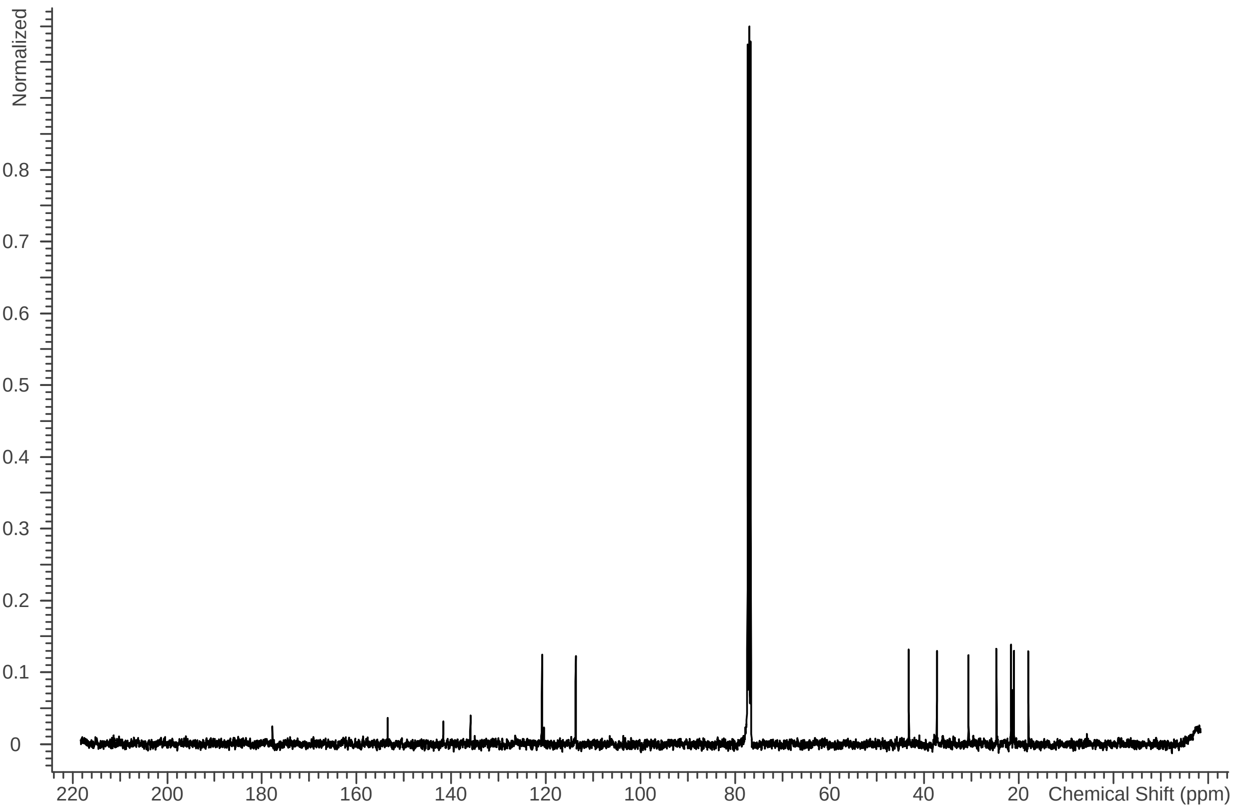

Figure S28 – ^{13}C NMR spectrum (100 MHz, $CDCl_3$) of **3**

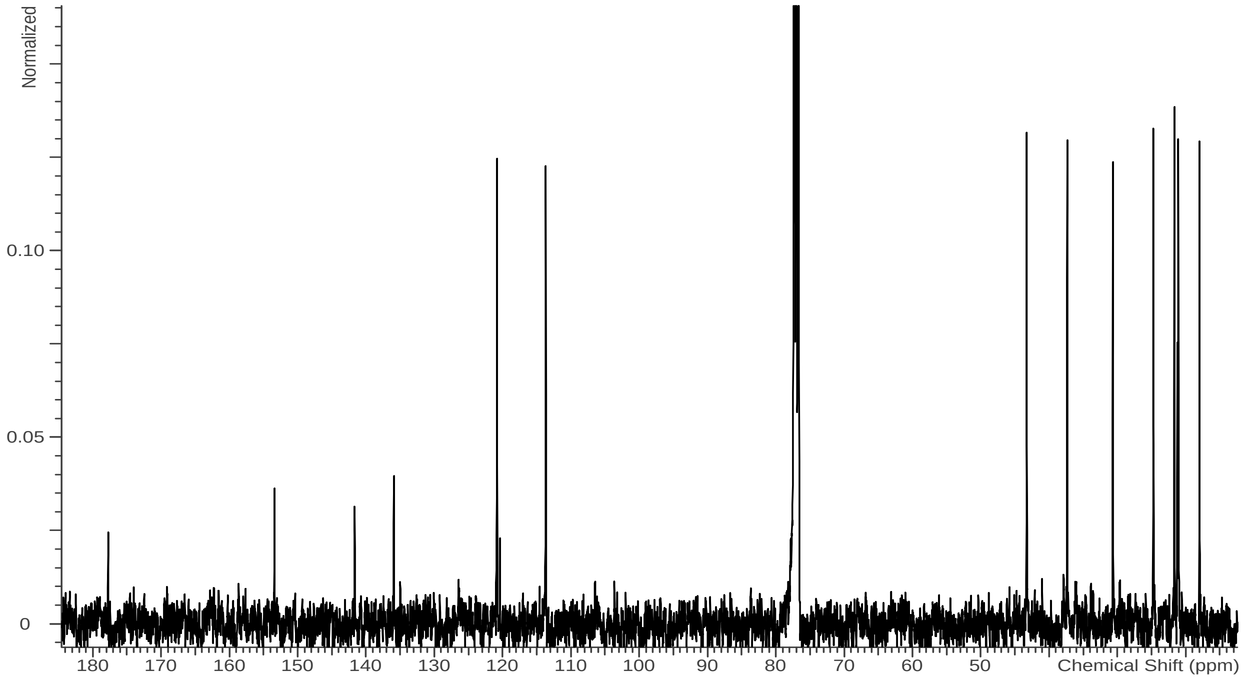

Figure S29 – ^{13}C NMR spectrum zoomned (100 MHz, $CDCl_3$) of **3**

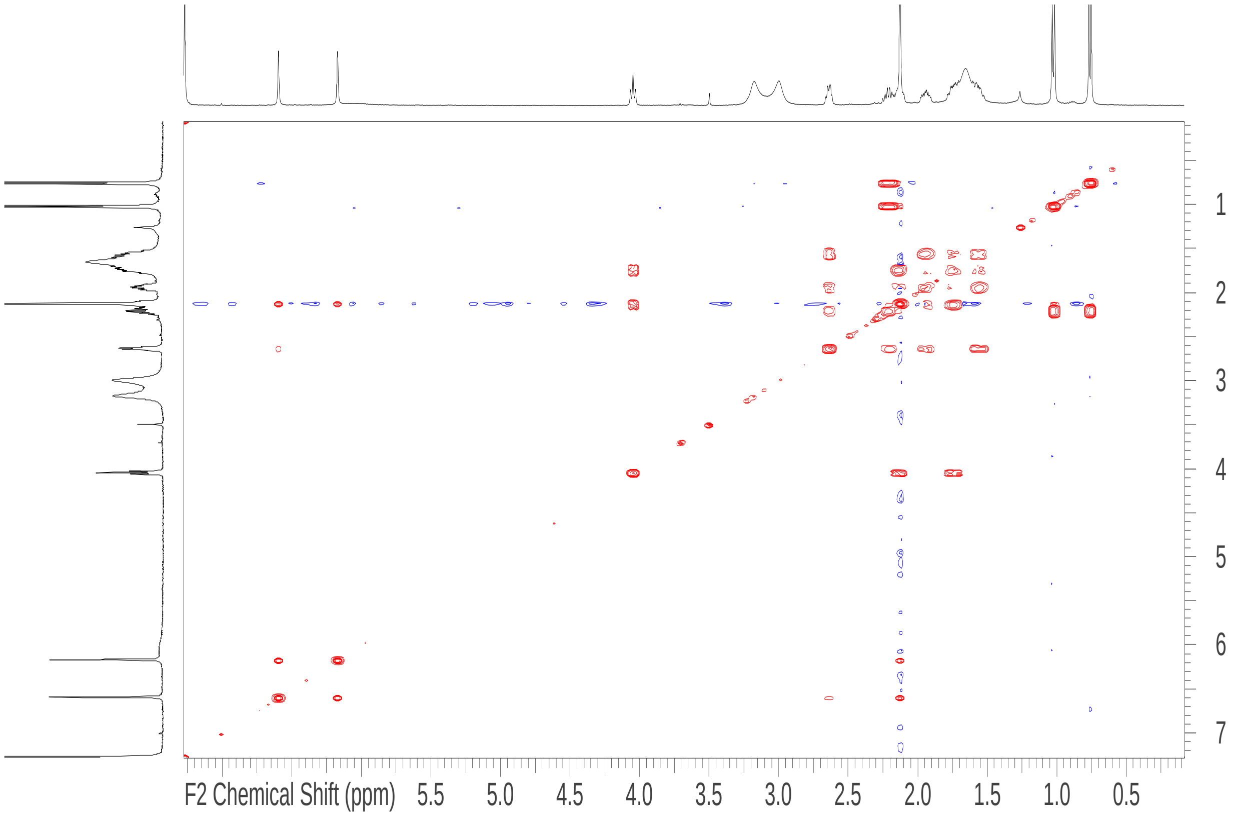

Figure S30 – COSY NMR spectrum (400 MHz, $CDCl_3$) of **3**

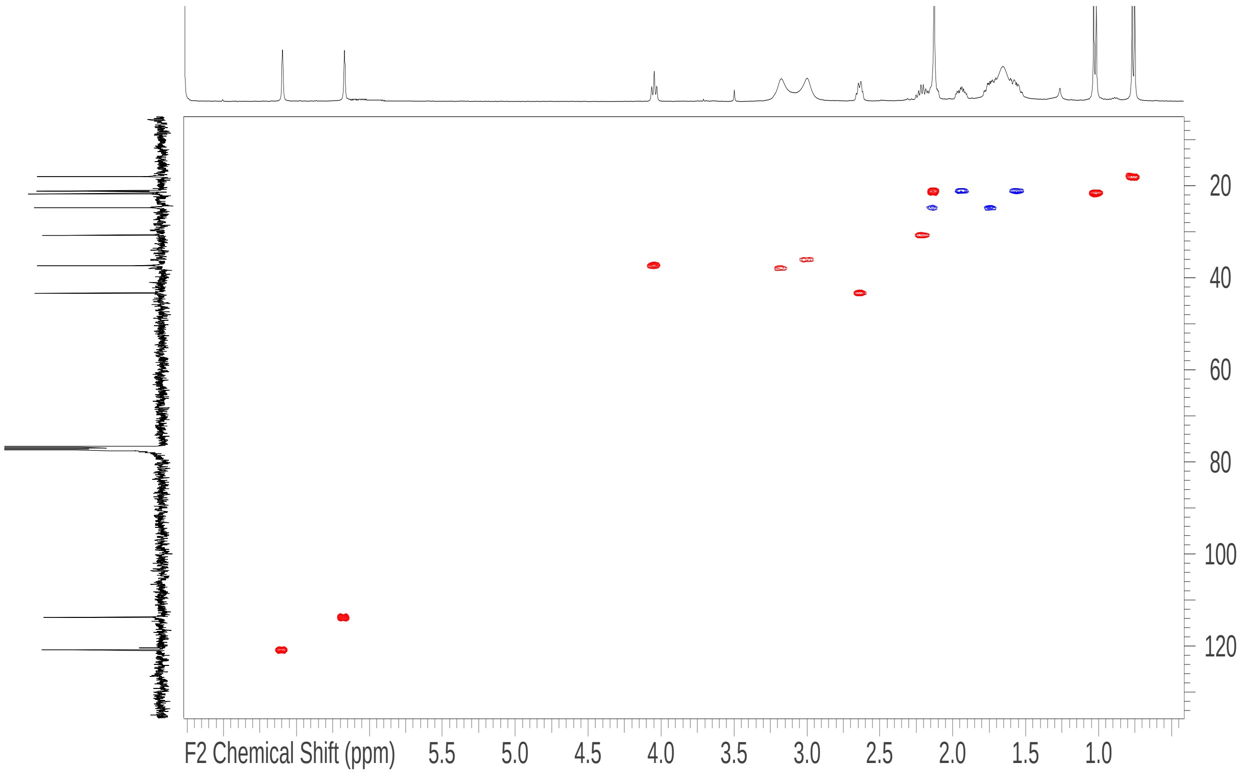

Figure S31 – HSQC NMR spectrum (400 MHz, $CDCl_3$) of **3**

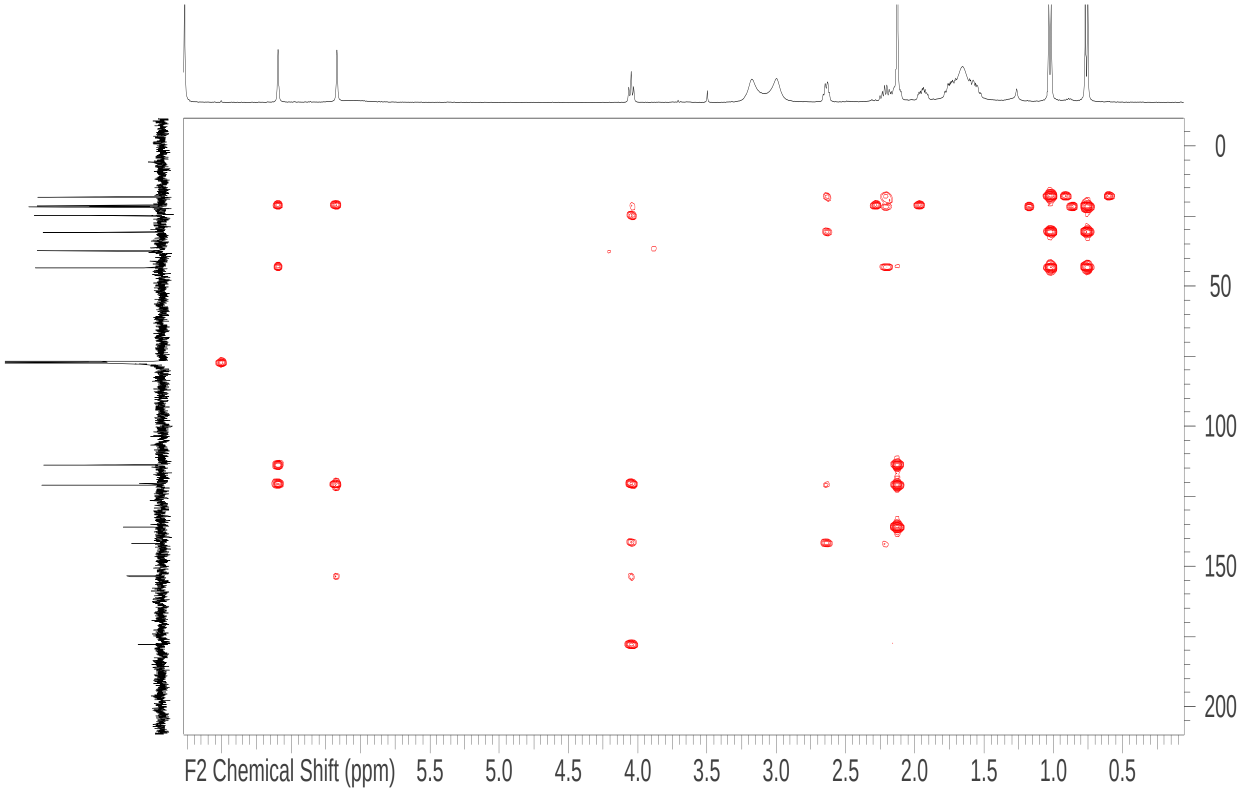

Figure S32 – HMBC NMR spectrum (400 MHz, $CDCl_3$) of **3**

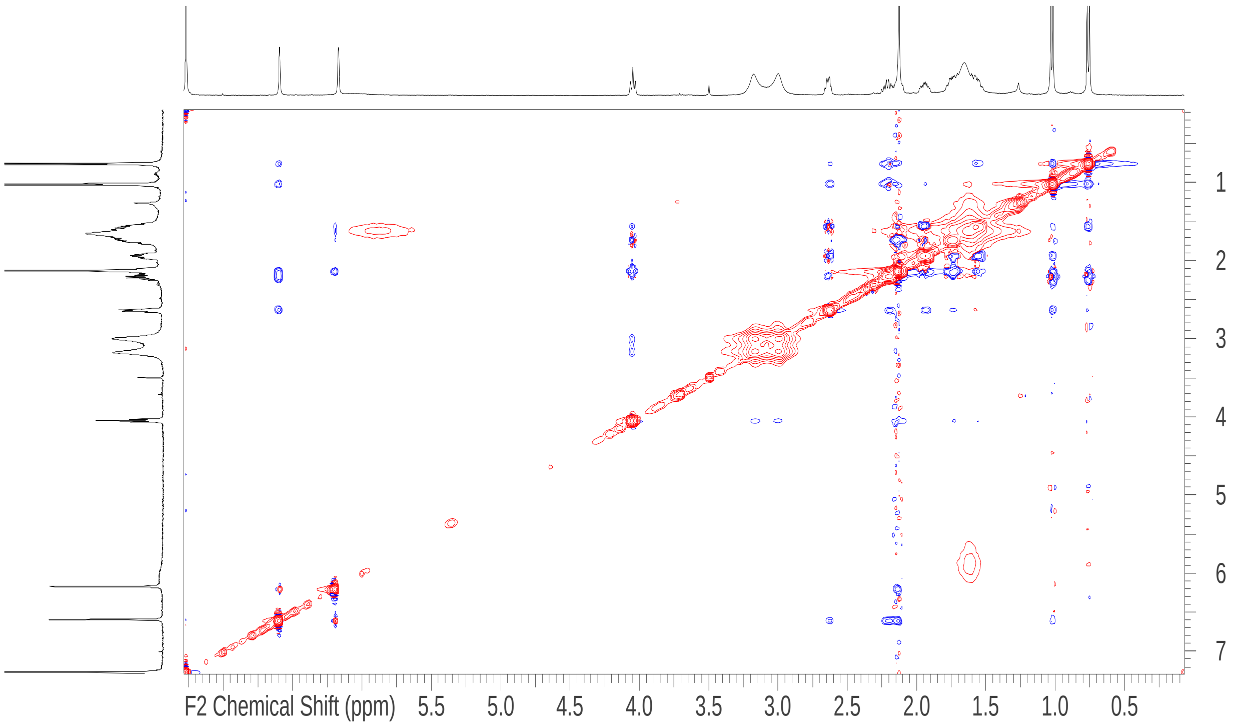

Figure S33 – NOESY NMR spectrum (400 MHz, $CDCl_3$) of **3**

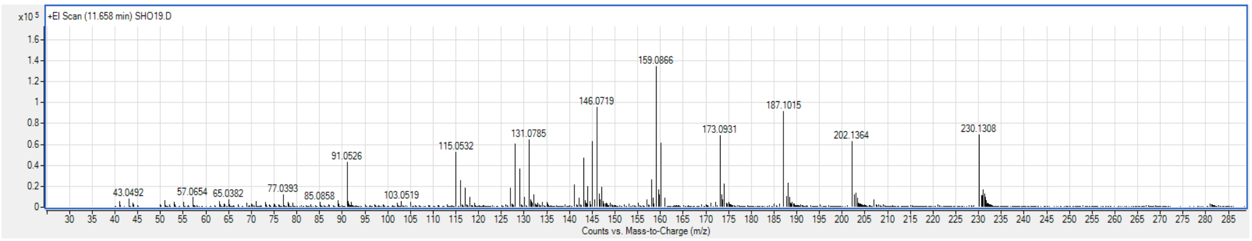

Figure S34 – GC-MS analysis of **3**

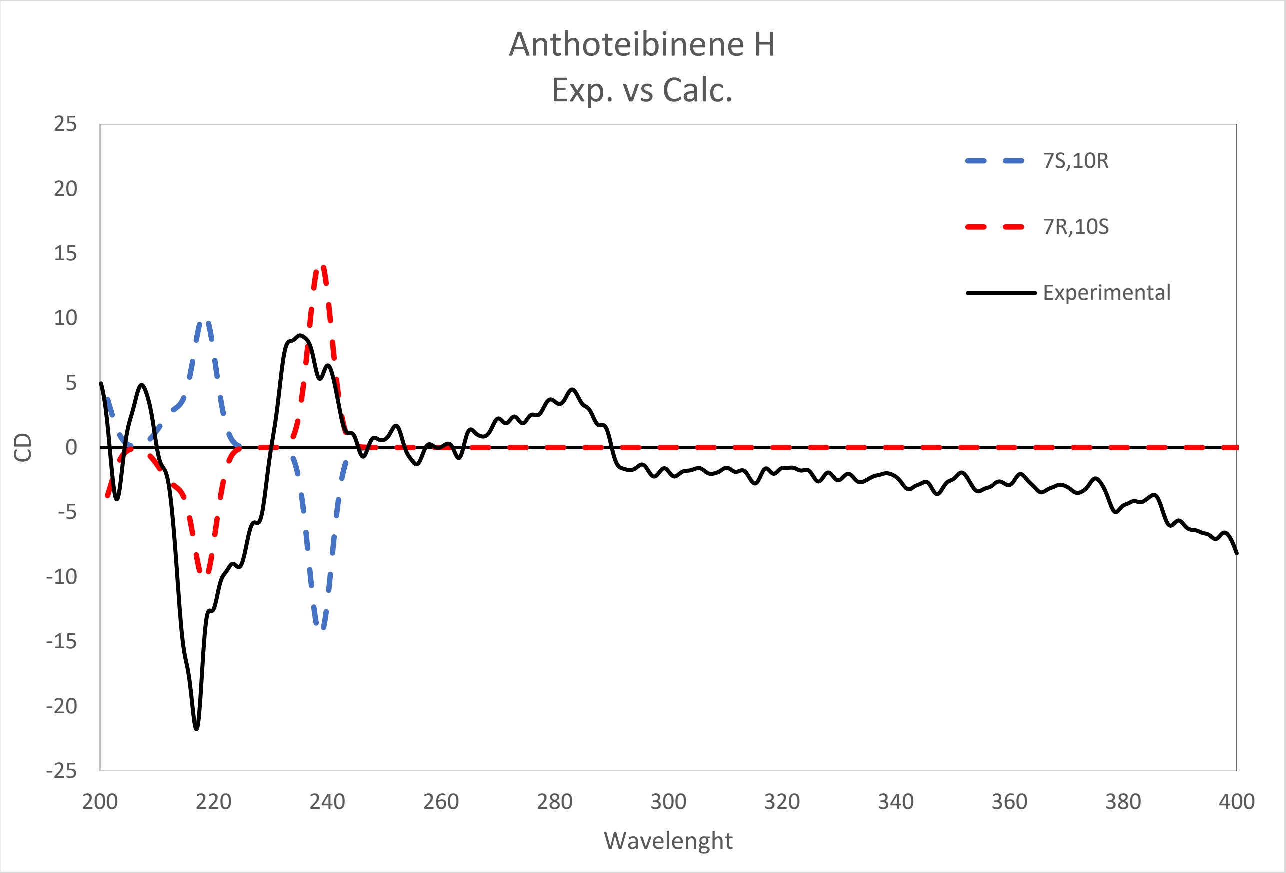

Figure S35 – ECD analysis of **3**

Table S4 – NMR Data for Anthoteibinene I (**4**) (600 (^{1}H) and 150 (^{13}C) MHz, $(CD_3)_2SO$)

pos	δ_C, type	δ_H	gCOSY	gHMBC	Key NOESY
**1**	125.2, C				
**2**	146.8, C				
**3**	109.4, CH	7.05, s	15	1, 4, 5, 6, 15	
**4**	123.1, C				
**5**	147.0, C				
**6**	120.1, C				
**7**	37.5, CH	2.98, ddd (4.8, 5.2, 6.8)	8a, 8b, 11	1, 5, 6, 9, 8	OH
**8a**	25.7 CH_2	2.23, o/l*	7, 8b, 9		OH
**8b**		1.54, dddd (4.8, 6.0, 12.5, 13.0)	7, 8a, 9	7, 9, 11	
**9**	15.9, CH_2	2.66, m (2H)	8a, 8b, 14	1, 7, 8, 10,14	
**10**	116.2, C				
**11**	31.1, C	1.72, octet (6.8)	7, 12, 13	7	OH
**12**	20.7, CH_3	0.94, d (6.7)	11	7, 11, 13	
**13**	21.2, CH_3	0.91, d (6.7)	11	7, 11, 12	OH
**14**	137.6, CH	7.44, s	9	2, 6, 10	
**15**	17.8, CH_3	2.25, s*	3	3, 4, 5	OH
**OH**		7.92, s		4, 5, 6	7, 8a, 11, 13, 15

*Overlapping ^{1}H NMR signals, 2D assignments based on proximity likelihood

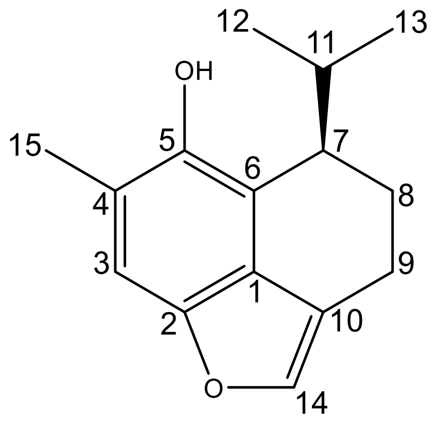

Figure S36 – Structure of **4**

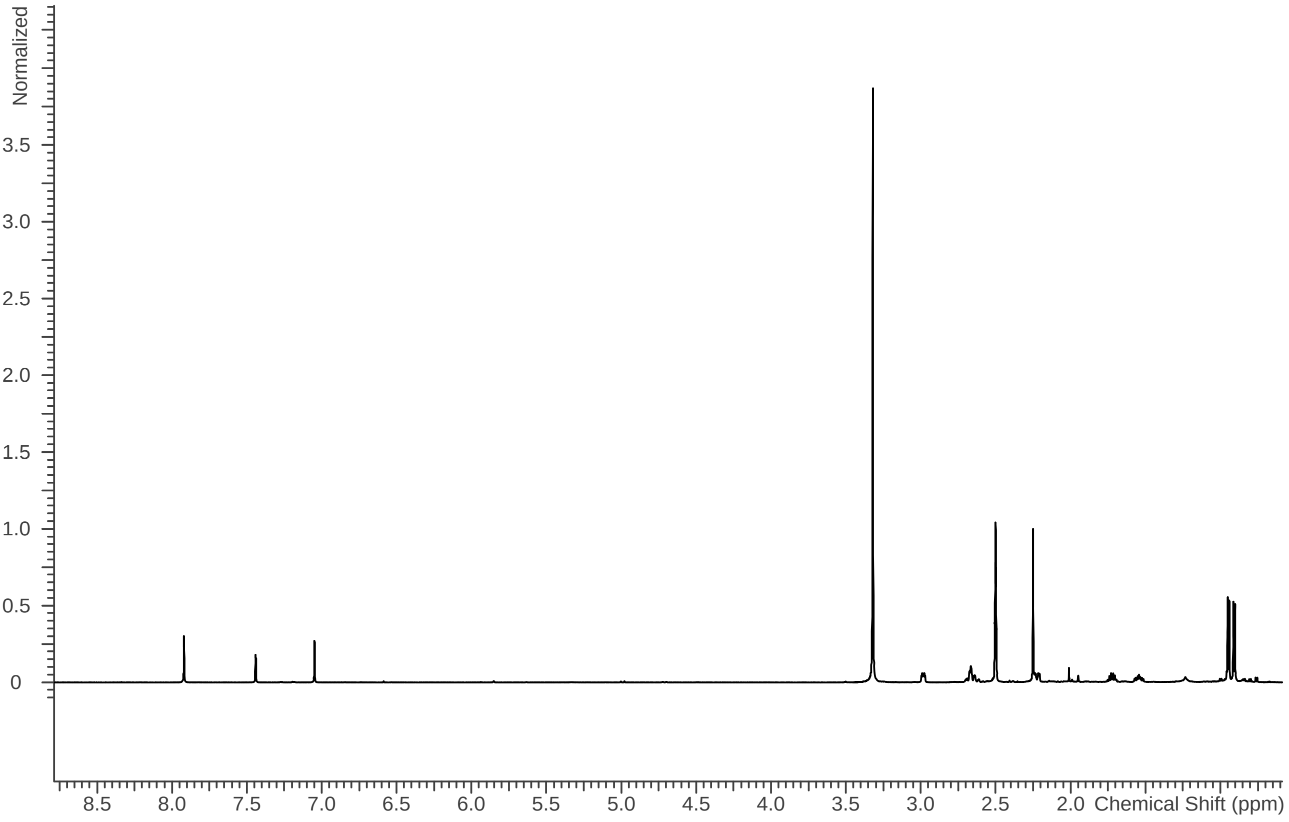

Figure S37 – ^{1}H NMR spectrum (600 MHz, $(CD_3)_2SO$) of **4**

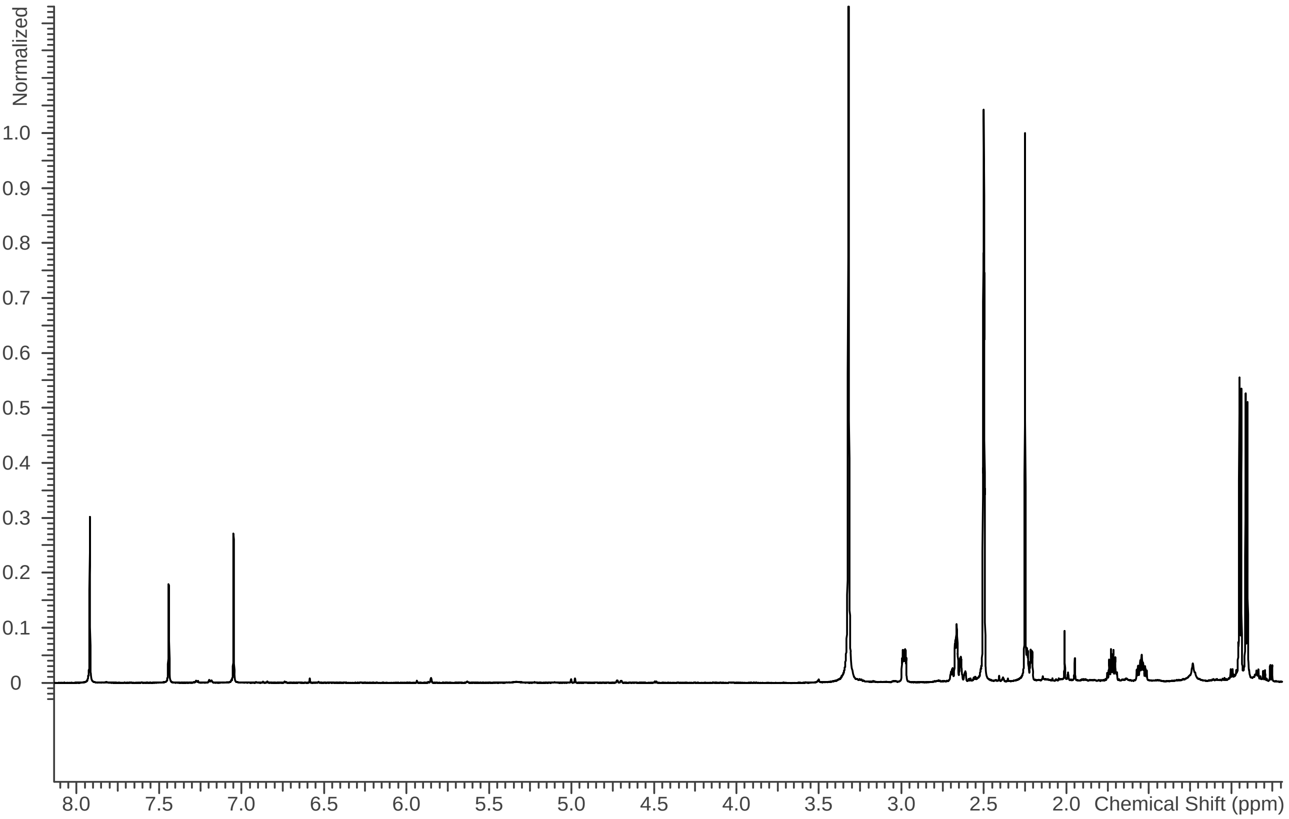

Figure S38 – ^{1}H NMR spectrum zoomed (600 MHz, $(CD_3)_2SO$) of **4**

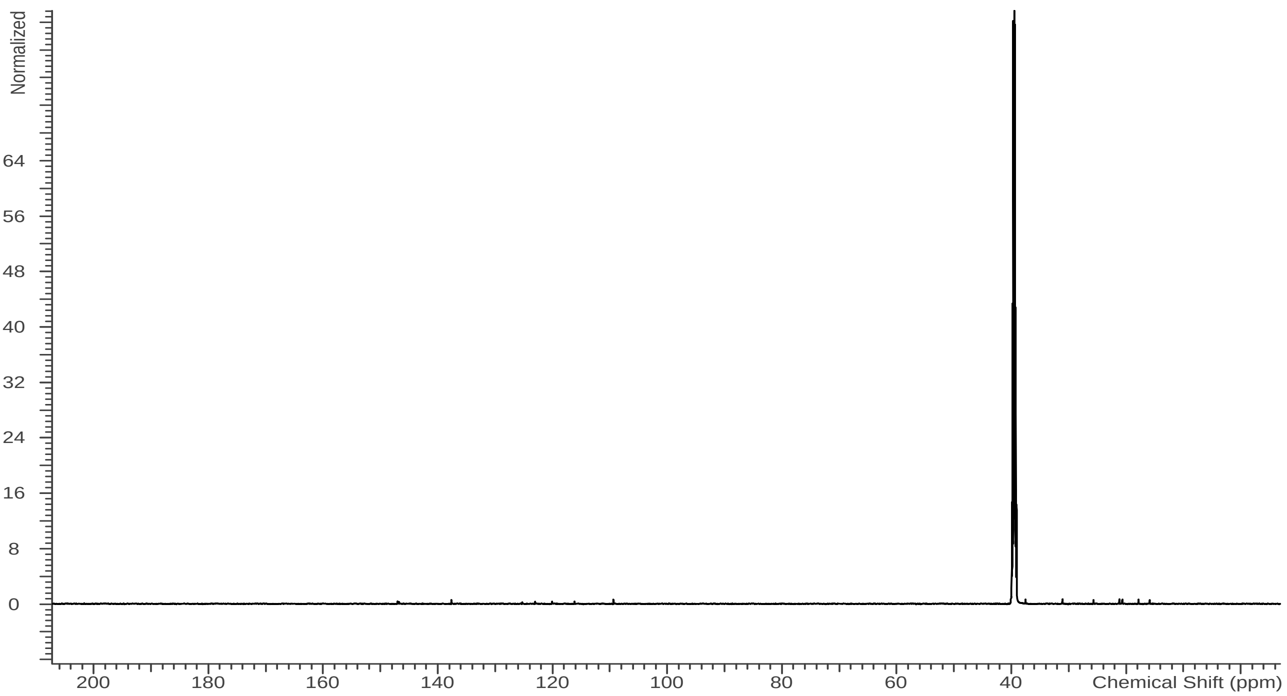

Figure S39 – ^{13}C NMR spectrum (150 MHz, $(CD_3)_2SO$) of **4**

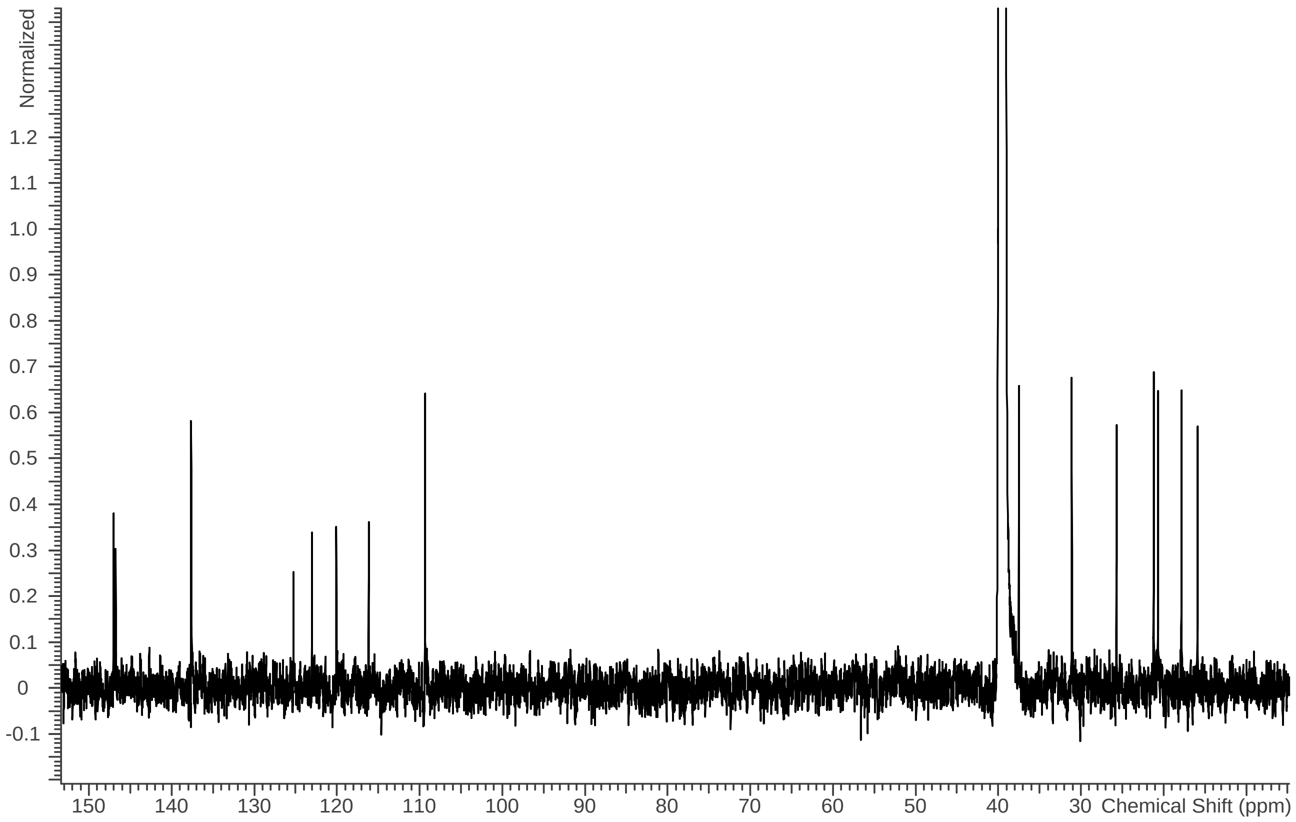

Figure S40 – ^{13}C NMR spectrum zoomed (150 MHz, $(CD_3)_2SO$) of **4**

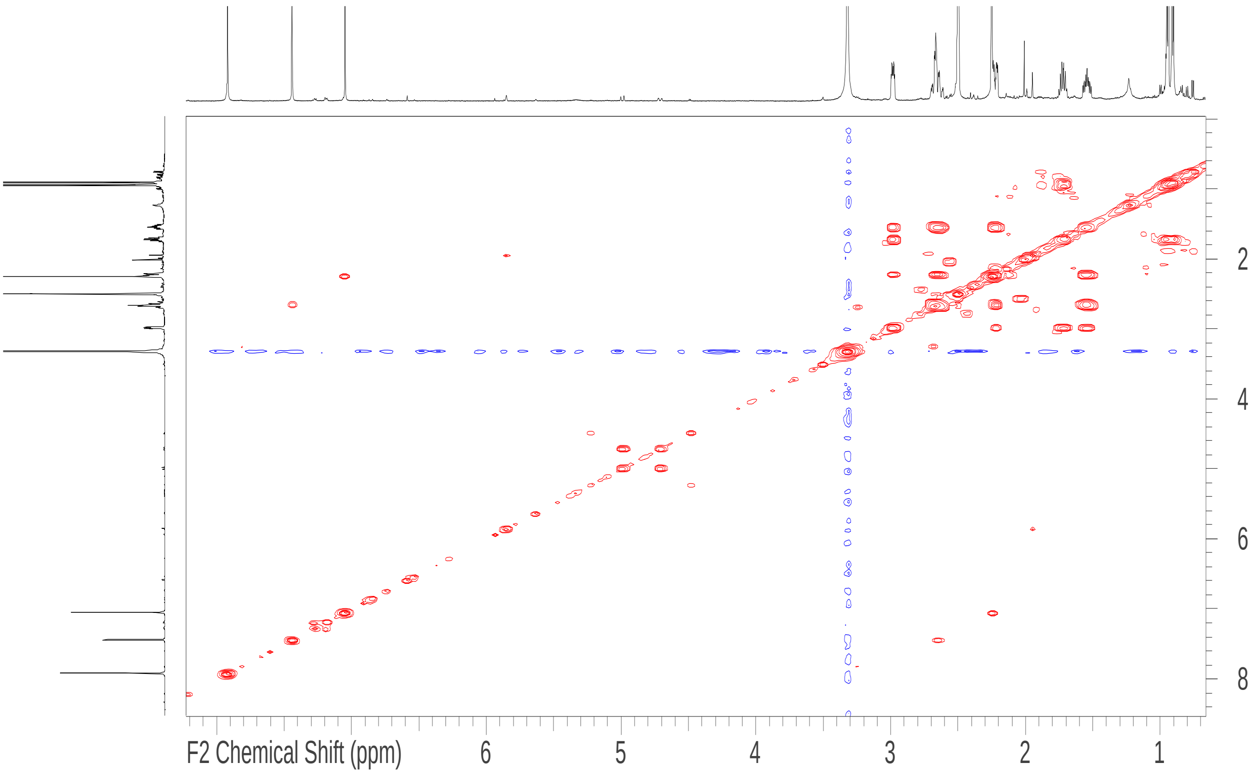

Figure S41 – COSY NMR spectrum (600 MHz, $(CD_3)_2SO$) of **4**

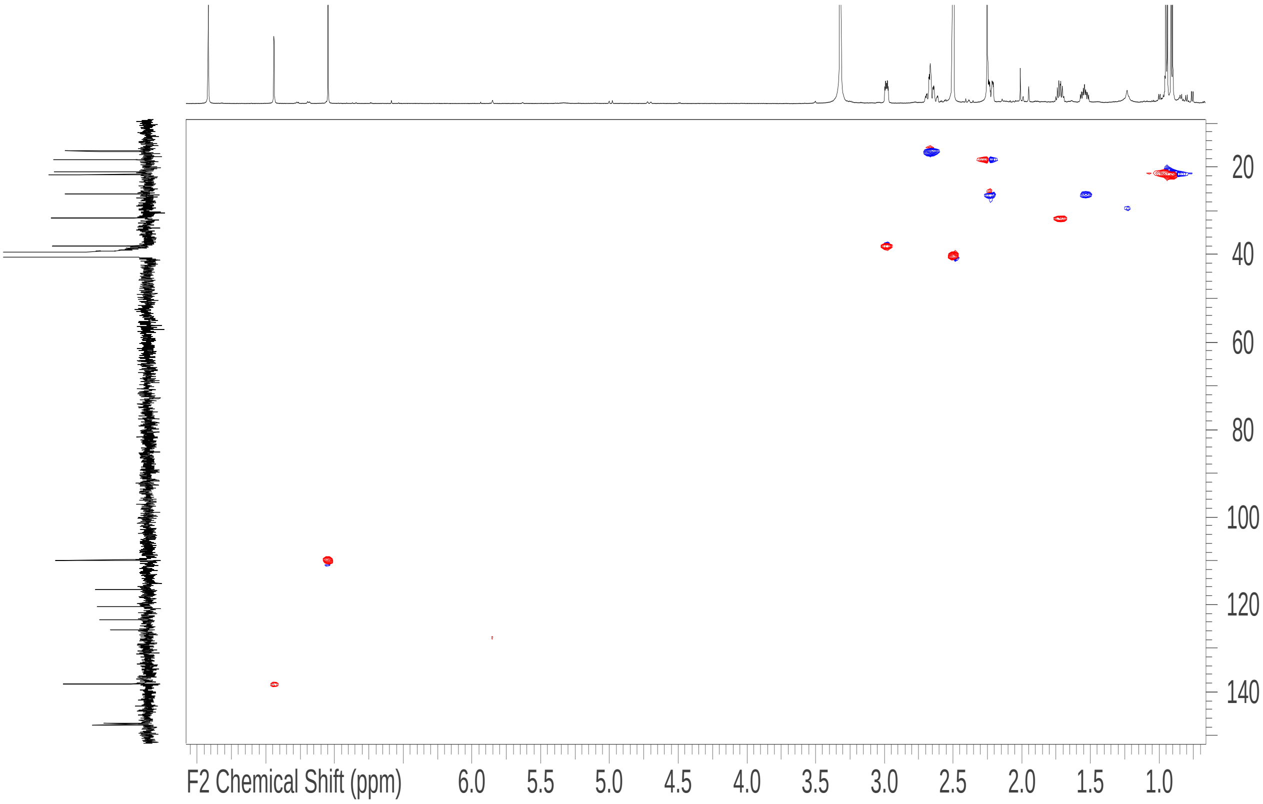

Figure S42 – HSQC NMR spectrum (600 MHz, $(CD_3)_2SO$) of **4**

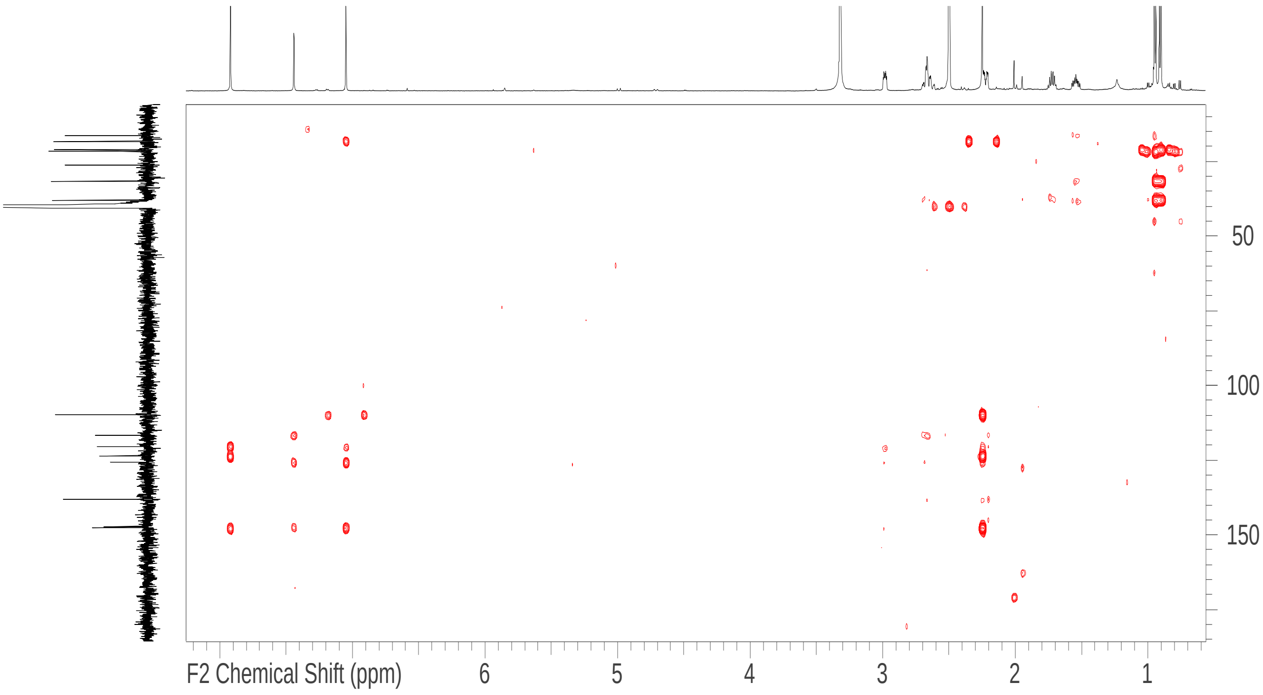

Figure S43 – HMBC NMR spectrum (600 MHz, $(CD_3)_2SO$) of **4**

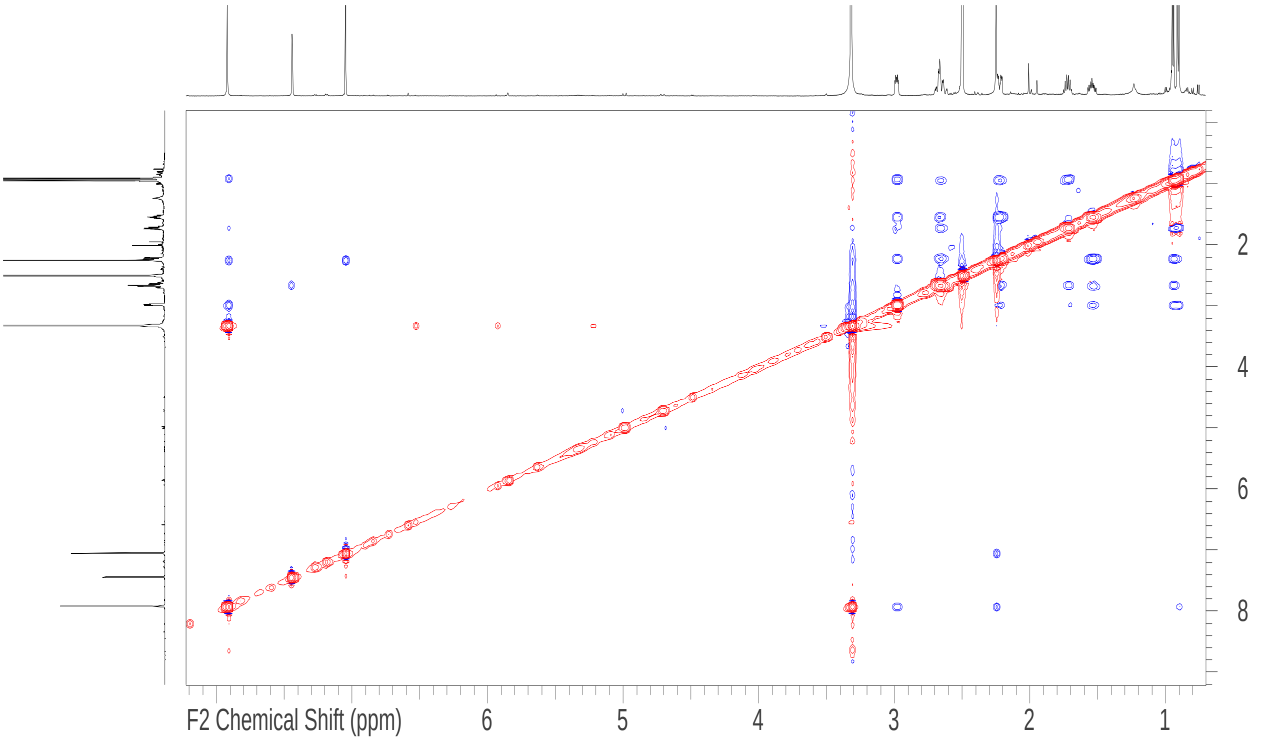

Figure S44 – NOESY NMR spectrum (600 MHz, $(CD_3)_2SO$) of **4**

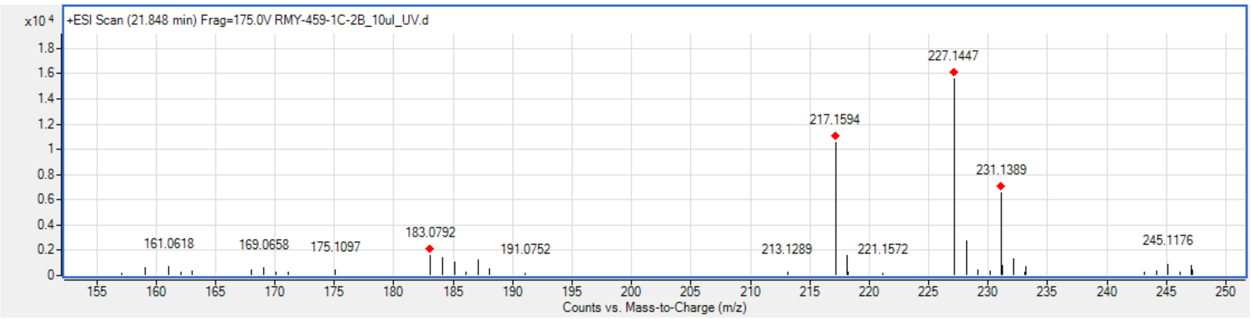

Figure S45 – HRESIMS analysis of **4**

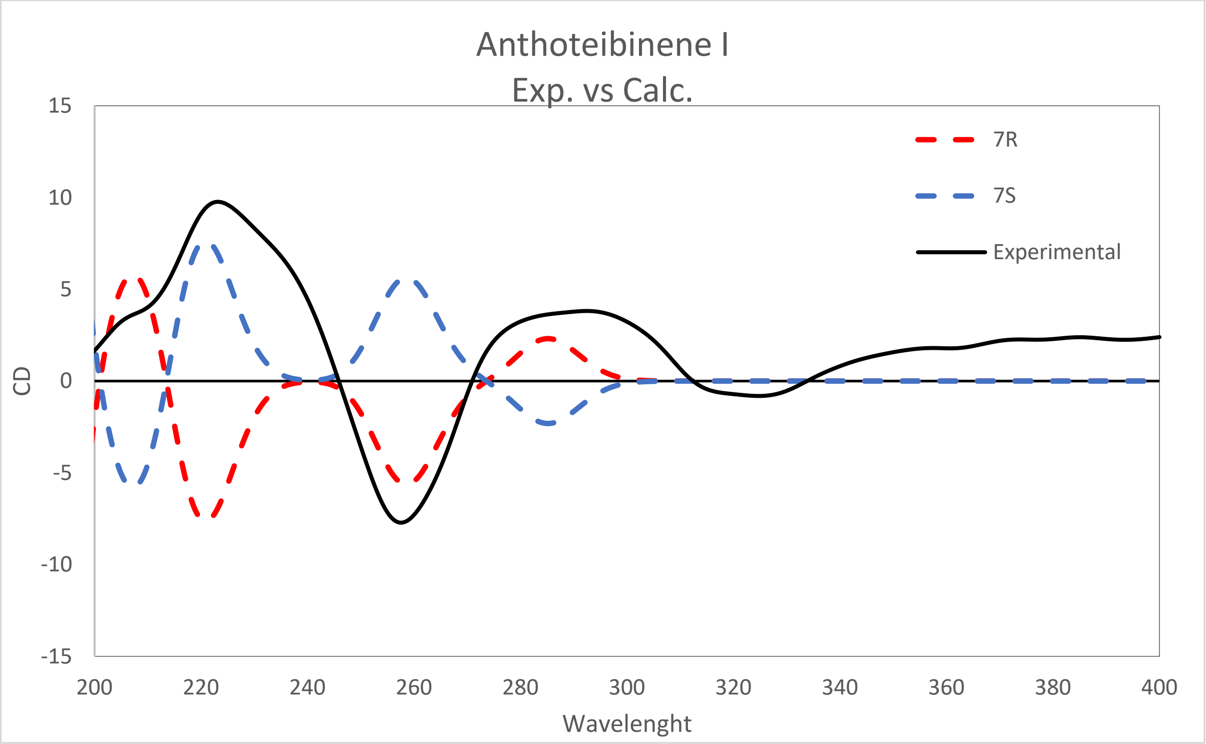

Figure S46 – ECD analysis of **4**

Table S5 – NMR Data for Anthoteibinene J (**5**) (600 (^{1}H) and 150 (^{13}C) MHz, $(CD_3)_2SO$)

pos	δ_C, type	δ_H	gCOSY	gHMBC	Key NOESY
**1**	126.7, C				
**2**	141.7, C				
**3**	137.5, C				
**4**	119.9, C				
**5**	122.2, C	6.74, s	7, 15	1, 2, 3, 4, 7, 10, 15	7, 11, 12,13, 15
**6**	125.0, C				
**7**	41.9, CH	2.59, ddd (4.6, 5.8, 6.6)	5, 8a, 8b, 11	1, 5, 6, 8, 11, 12, 13	5
**8a**	25.1 CH_2	1.86, o/l*	7, 9a, 9b	6, 7, 9, 10, 11	
**8b**		1.81, o/l*	7, 9a, 9b	6, 7, 9, 10, 11	
**9a**	17.7, CH_2	2.65, ddd (5.6, 5.8, 16.1)	8b, 9b, 14	1, 7, 8, 10, 14	
**9b**		2.76, ddd (4.9, 8.0, 16.0)	8b, 9a, 14	1, 7, 8, 10, 14	11, 13
**10**	117.1, C				
**11**	29.0, C	2.03, octet (6.6)	7, 12, 13	6, 7, 8, 12, 13	5, 9a
**12**	21.3, CH_3	0.97, d (6.8)	11	7, 11, 13	5
**13**	18.9, CH_3	0.89, d (6.8)	11	7, 11, 12	5, 9a
**14**	137.1, CH	7.50, s	9a, 9b	1, 2, 10	
**15**	16.2, CH_3	2.24, s	5	1, 2, 3, 4, 5	5
**OH**		9.29, br s		4	

*Overlapping ^{1}H NMR signals, 2D assignments based on proximity likelihood

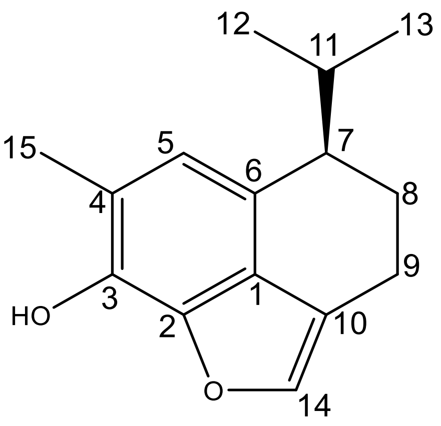

Figure S47 – Structure of **5**

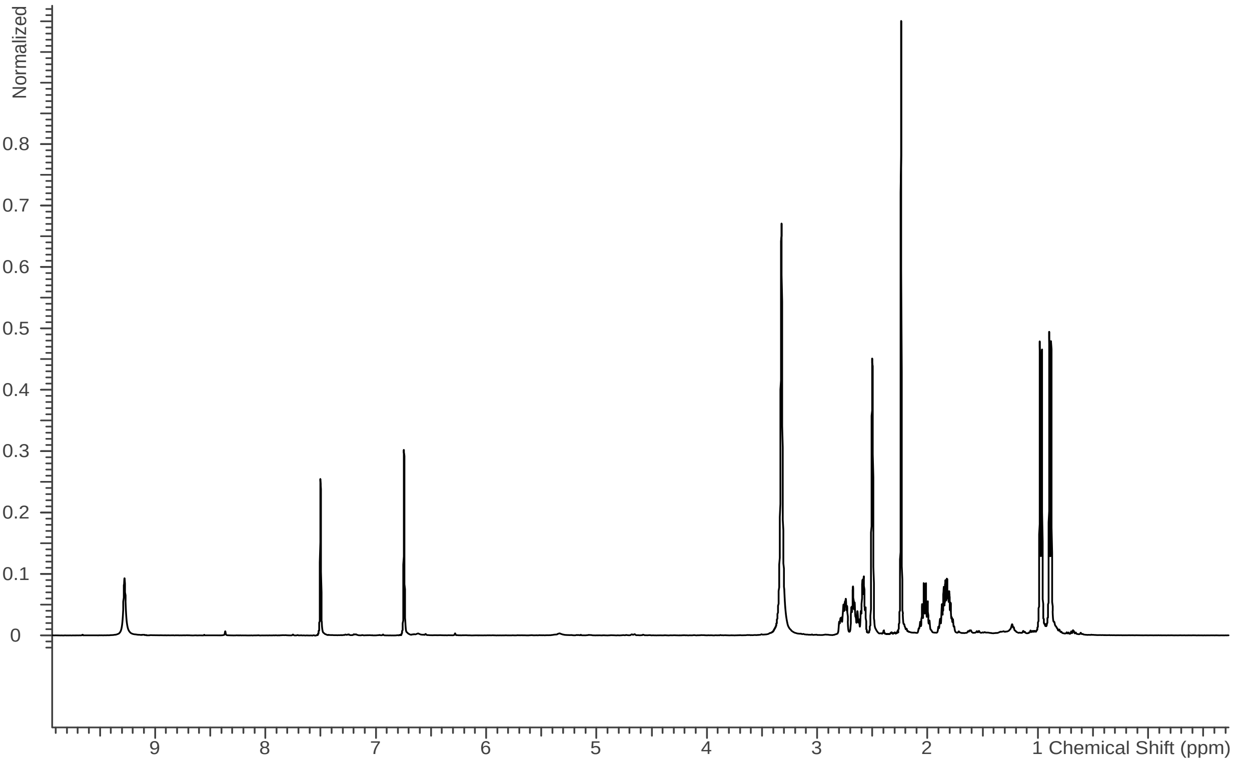

Figure S48 – ^{1}H NMR spectrum (600 MHz, $(CD_3)_2SO$) of **5**

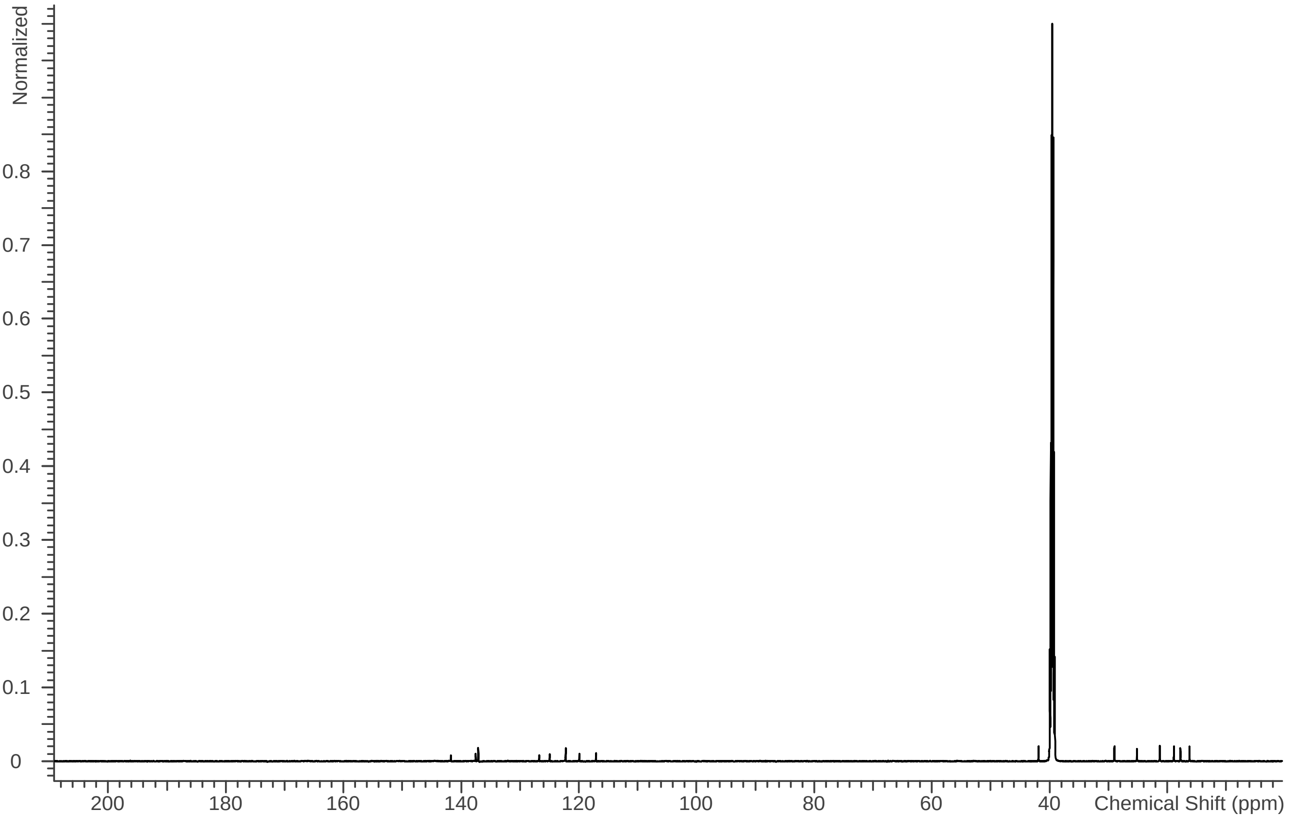

Figure S49 – ^{13}C NMR spectrum (150 MHz, $(CD_3)_2SO$) of **5**

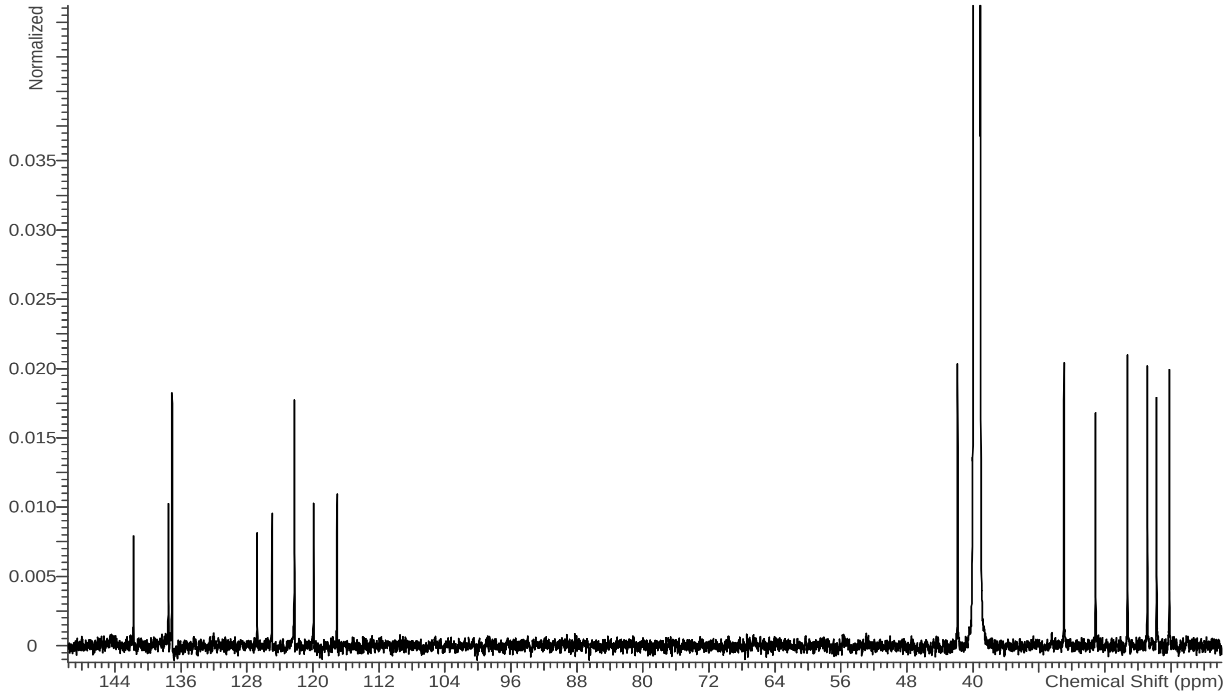

Figure S50 – ^{13}C NMR spectrum zoomed (150 MHz, $(CD_3)_2SO$) of **5**

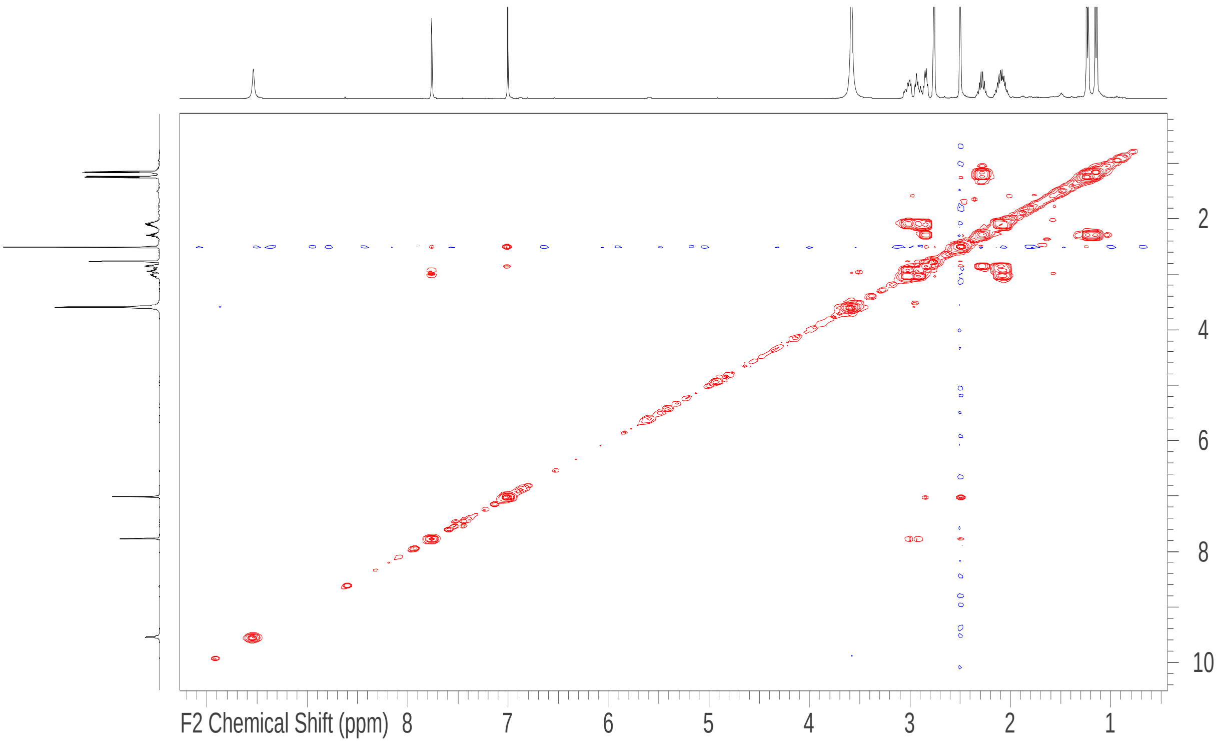

Figure S51 – COSY NMR spectrum (600 MHz, $(CD_3)_2SO$) of **5**

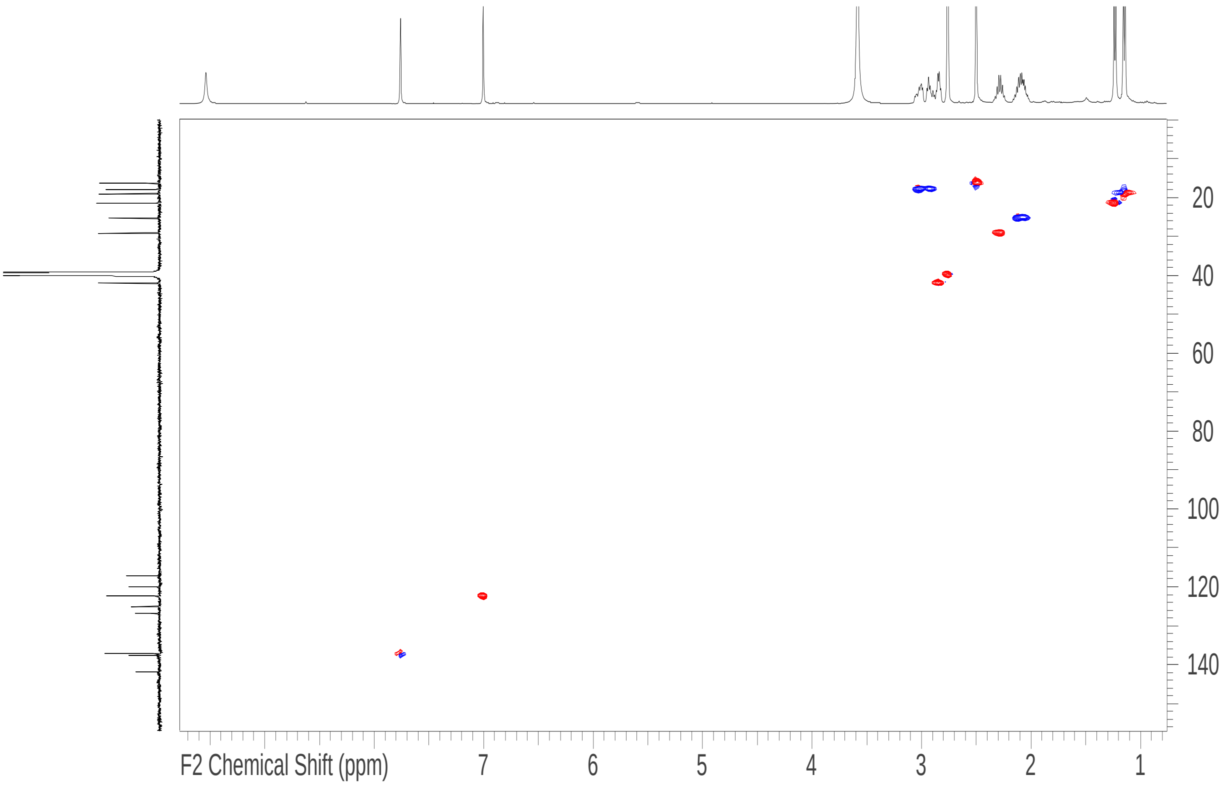

Figure S52 – HSQC NMR spectrum (600 MHz, $(CD_3)_2SO$) of **5**

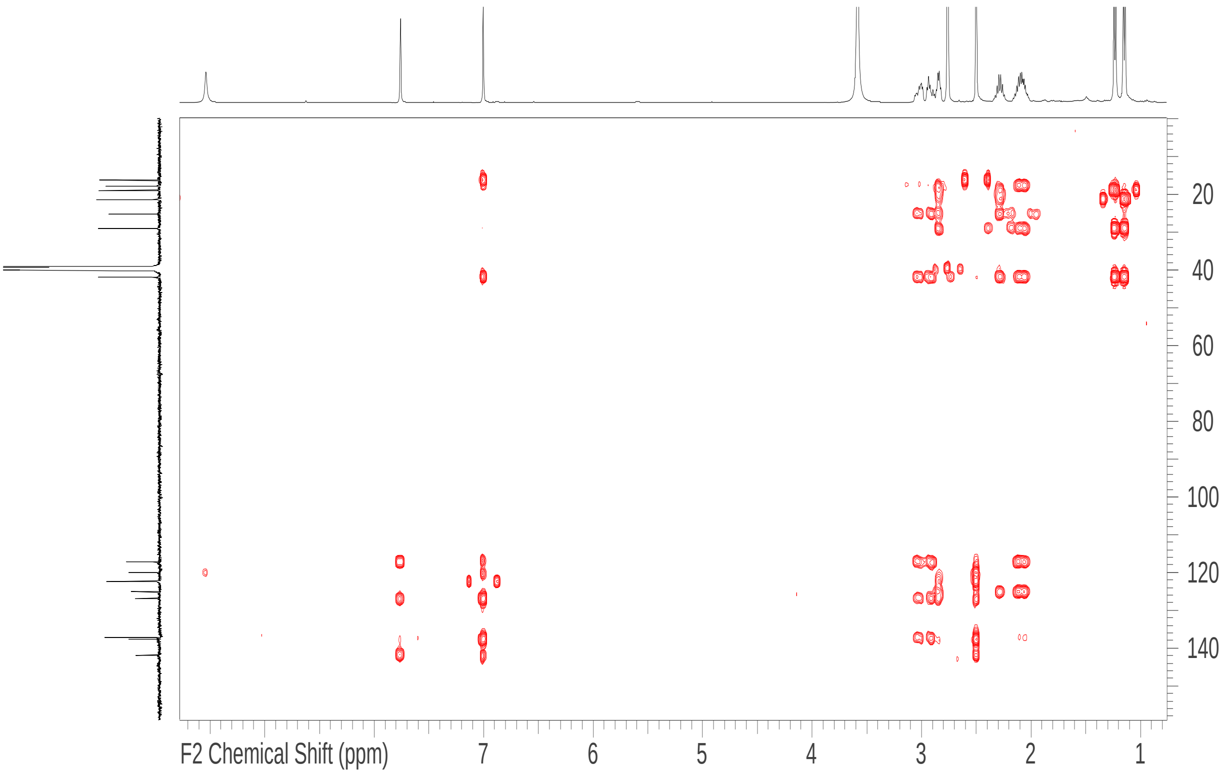

Figure S53 – HMBC NMR spectrum (600 MHz, $(CD_3)_2SO$) of **5**

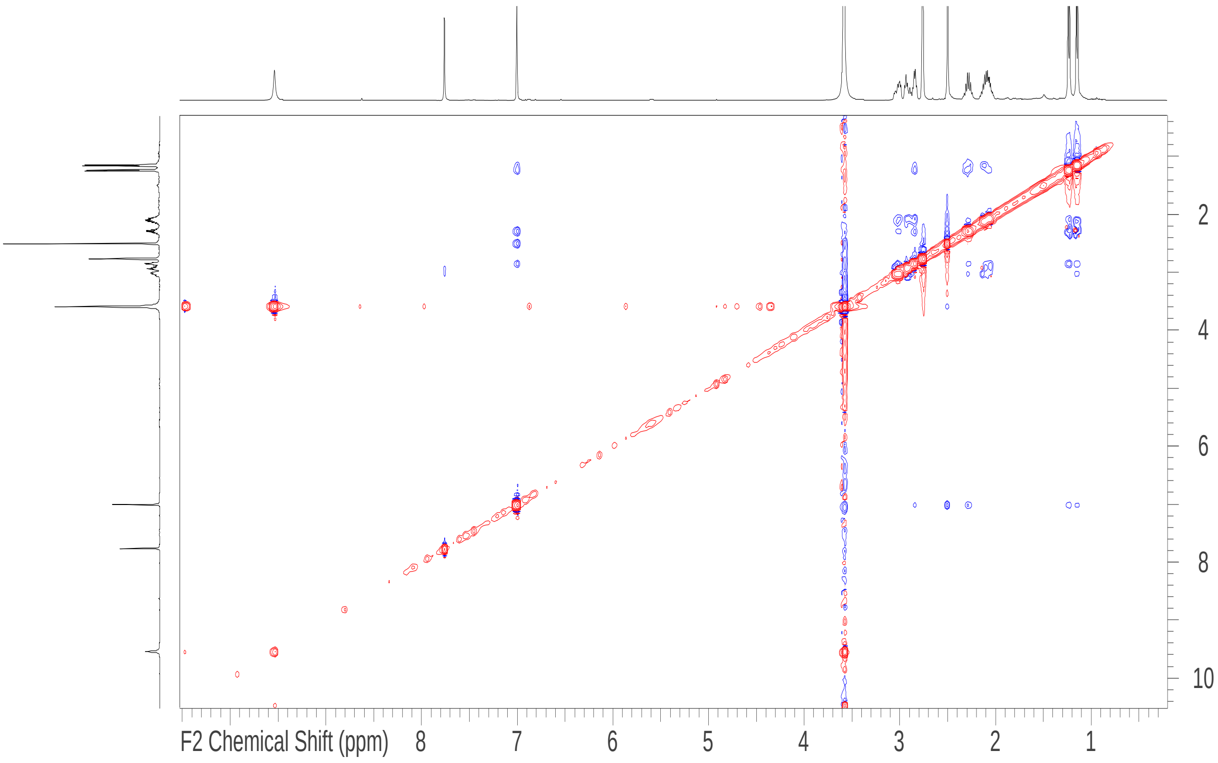

Figure S54 – NOESY NMR spectrum (600 MHz, $(CD_3)_2SO$) of **5**

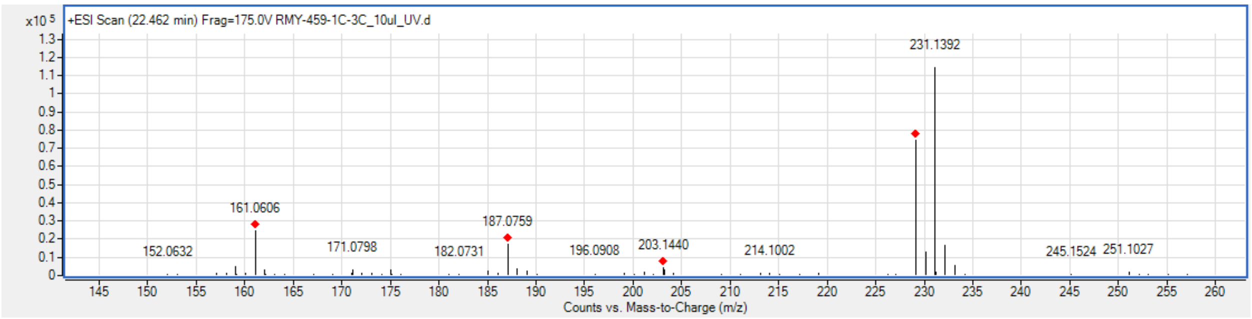

Figure S55 – HRESIMS analysis of **5**

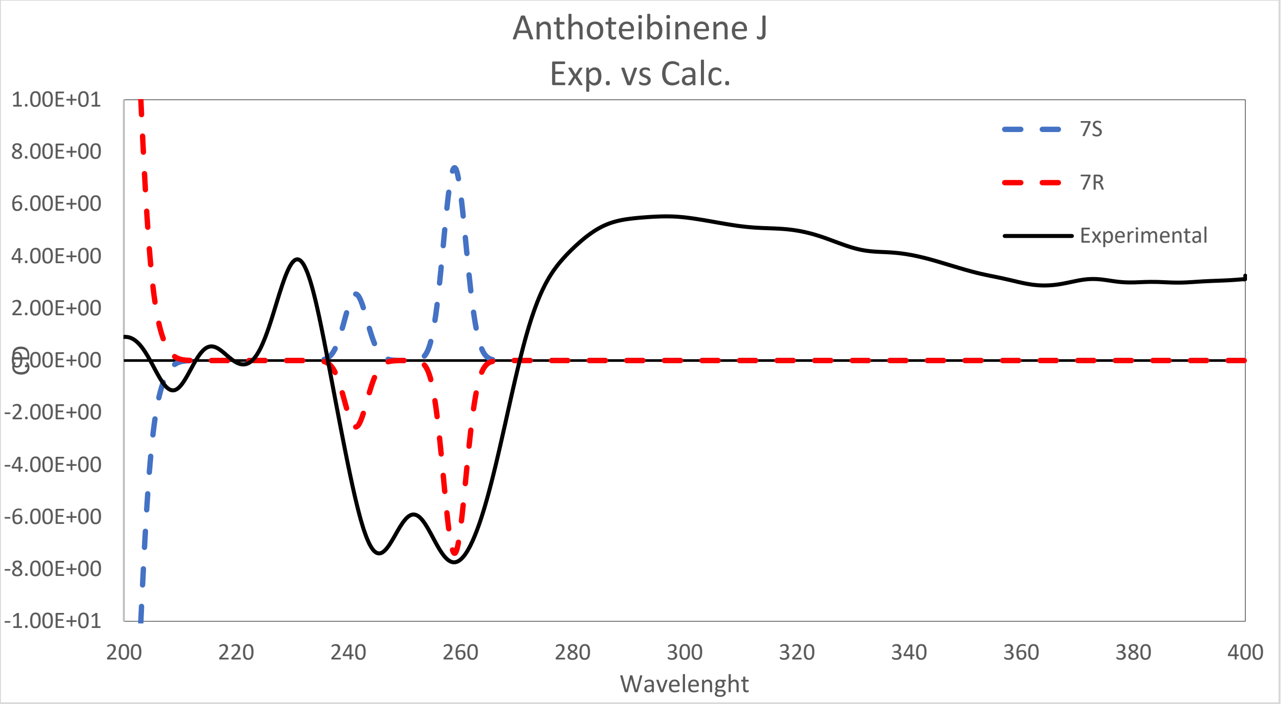

Figure S56 – ECD analysis of **5**

Table S6 – NMR Data for Anthoteibinene K (**6**) (400 (^{1}H) and 100 (^{13}C) MHz, $(CH_3)_2SO$)

pos	δ_C, type	δ_H (*J* in Hz)	gCOSY	gHMBC
**1**	124.9, C			
**2**	152.9, C			
**3**	108.4, CH	7.19, s		1, 2, 5, 15
**4**	134.0, C			
**5**	120.6, CH	6.95, s	15	1, 3, 4, 7, 15
**6**	135.1, C			
**7**	42.4, CH	2.76, o/l*	11	5, 6, 8, 9, 11, 12
**8**	24.9, CH_2	1.96, m (2H)	9a, 9b	6, 7, 9, 10, 11
**9a**	17.7, CH_2	2.88, m	8	1, 7, 8, 10, 14
**9b**		2.78, o/l*	8	7, 8, 10
**10**	116.5, C			
**11**	28.8, CH	2.17, octet (6.9)	7, 12, 13	6, 7, 8, 12, 13
**12**	21.2, CH_3	1.08, d (6.8)	11	7, 11, 13
**13**	18.8, CH_3	0.99, d (6.8)	11	7, 11, 12
**14**	137.3, CH	7.60, s		1, 2, 10
**15**	21.8, CH_3	2.50, s		3, 4, 5

*Overlapping ^{1}H NMR signals, 2D assignments based on proximity likelihood

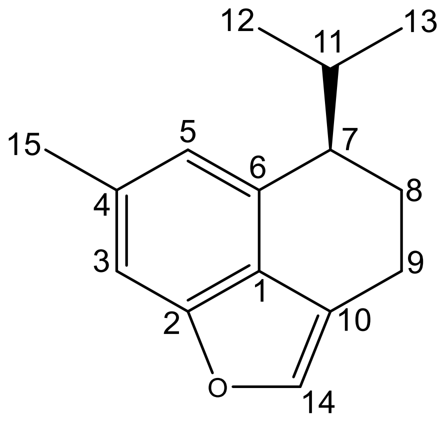

Figure S57 – Structure of **6**

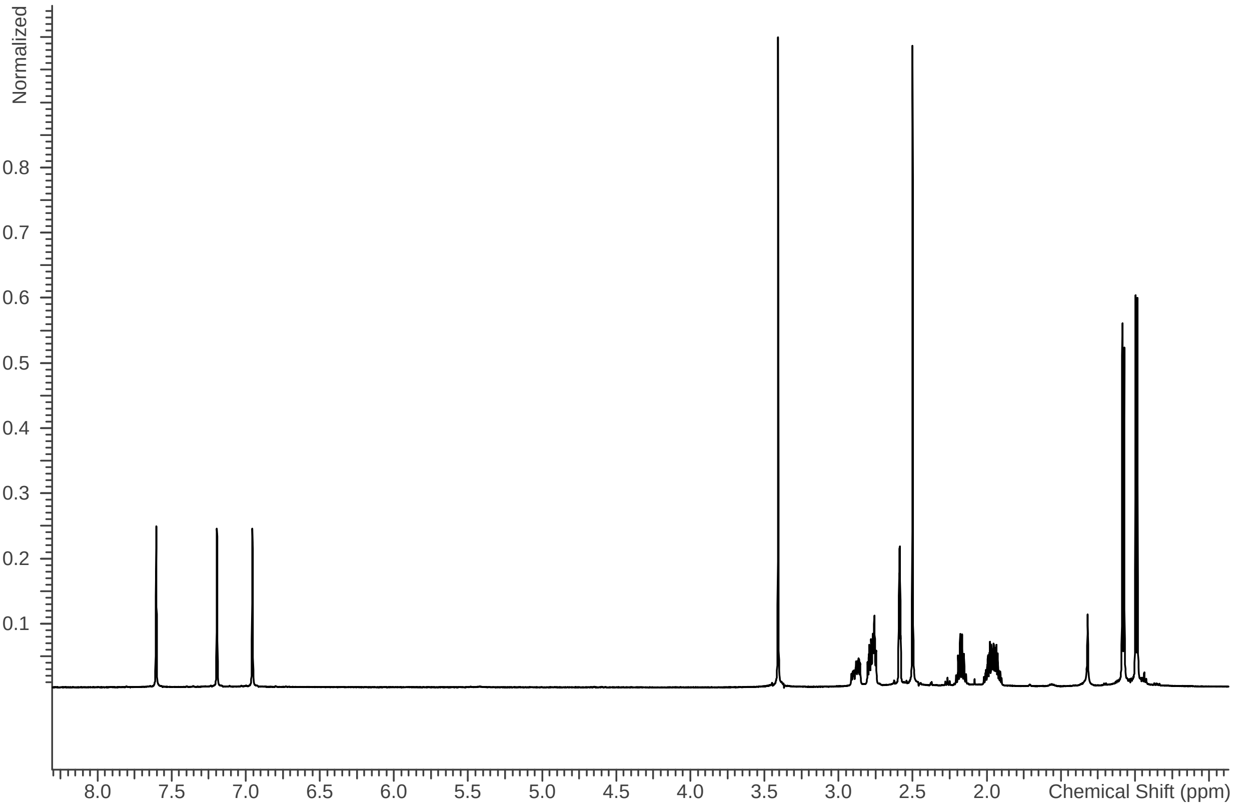

Figure S58 – ^{1}H NMR spectrum (400 MHz, $(CH_3)_2SO$) of **6**

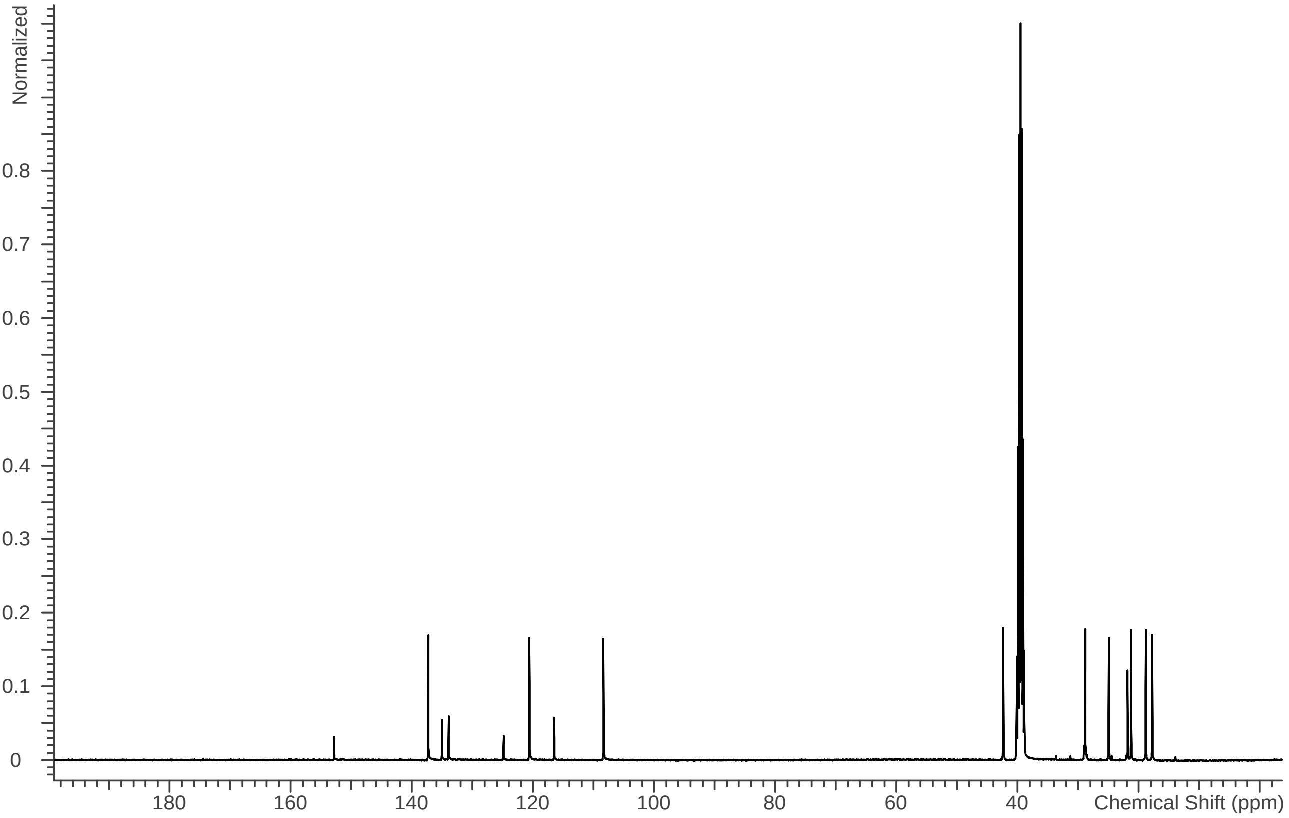

Figure S59 – ^{13}C NMR spectrum (100 MHz, $(CH_3)_2SO$) of **6**

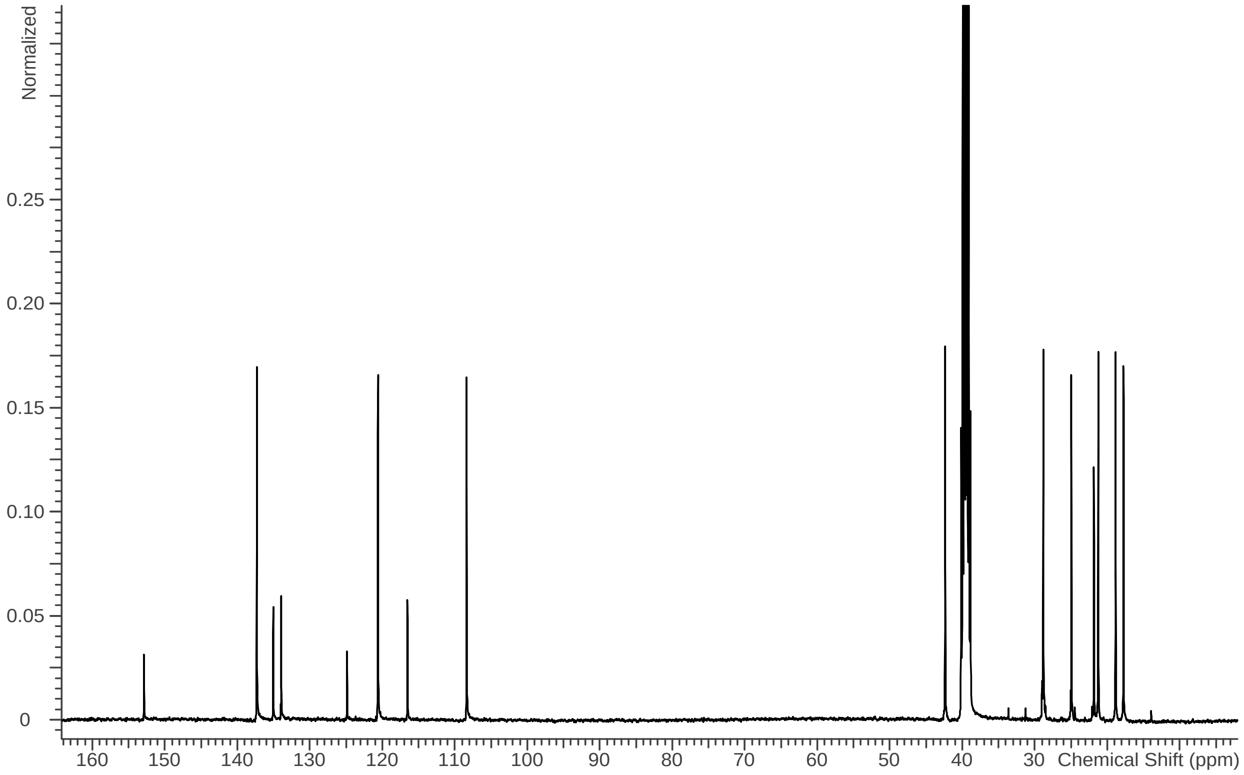

Figure S60 – ^{13}C NMR spectrum zoomed (100 MHz, $(CH_3)_2SO$) of **6**

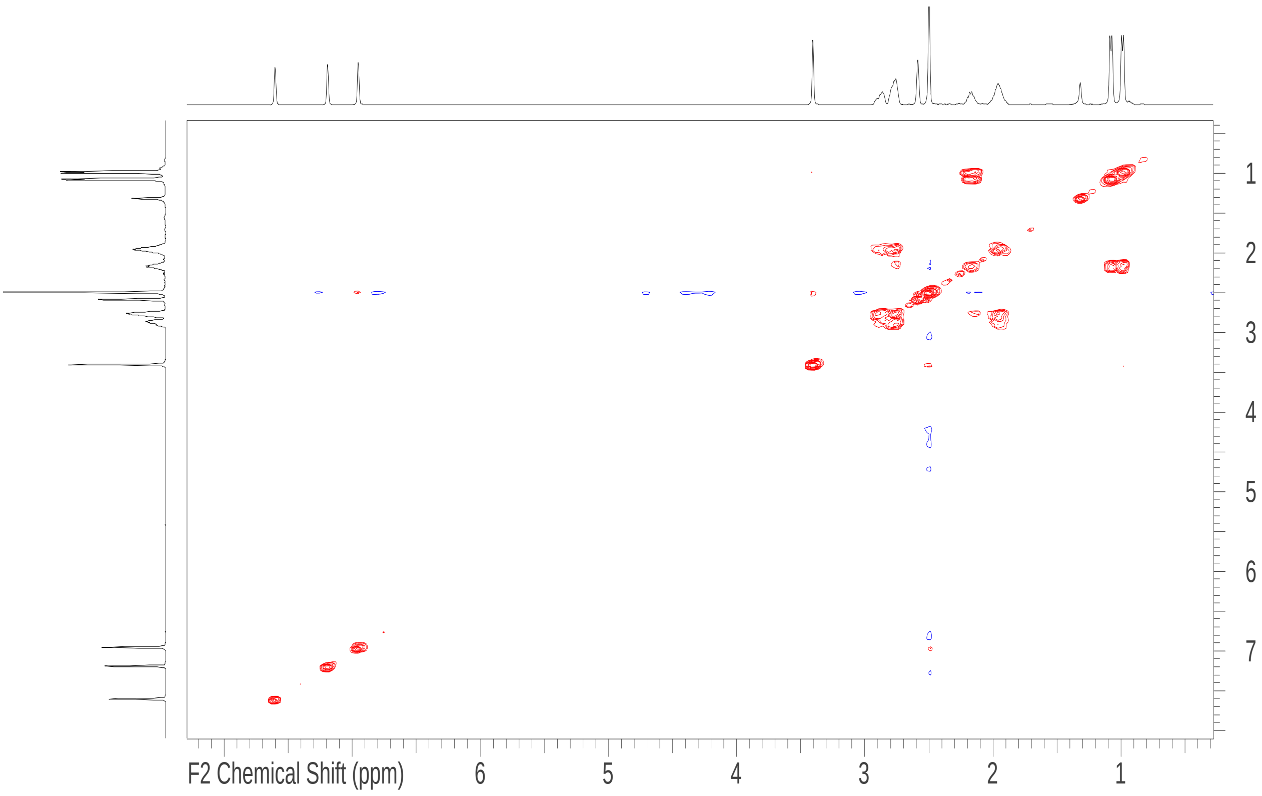

Figure S61 – COSY NMR spectrum (400 MHz, $(CH_3)_2SO$) of **6**

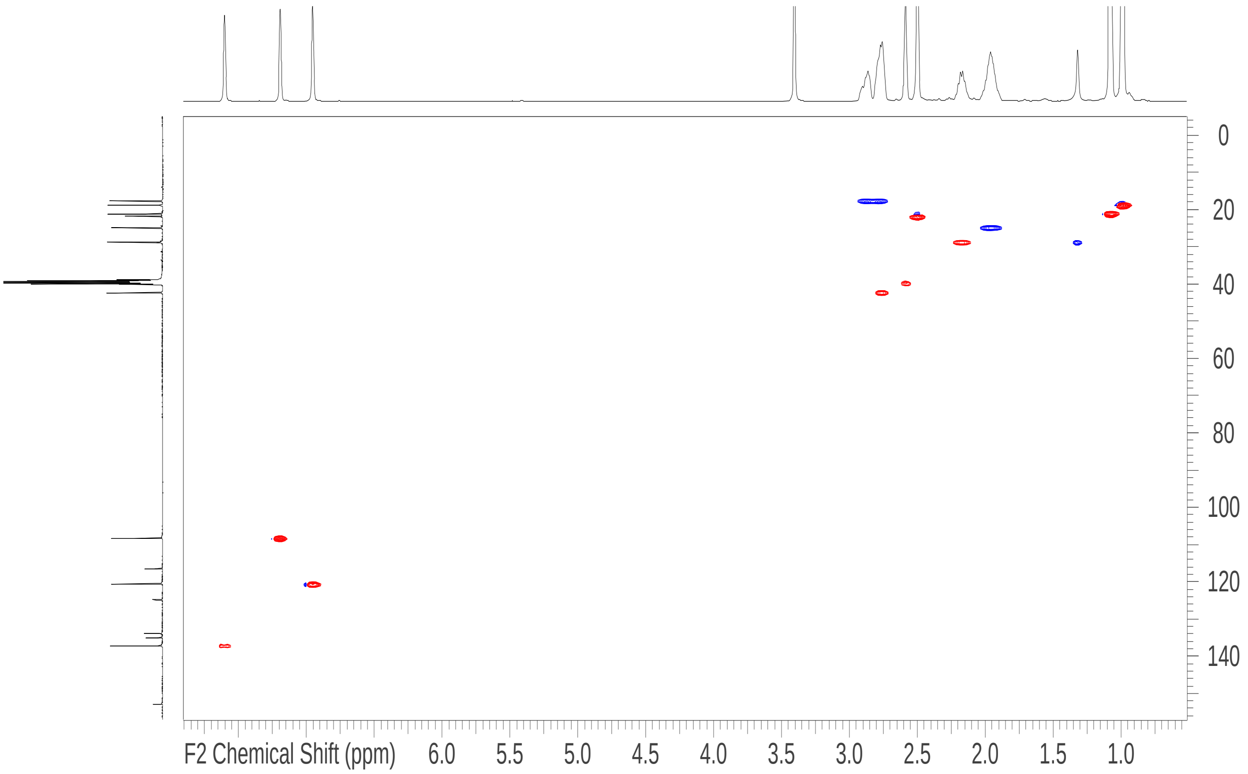

Figure S62 – HSQC NMR spectrum (400 MHz, $(CH_3)_2SO$) of **6**

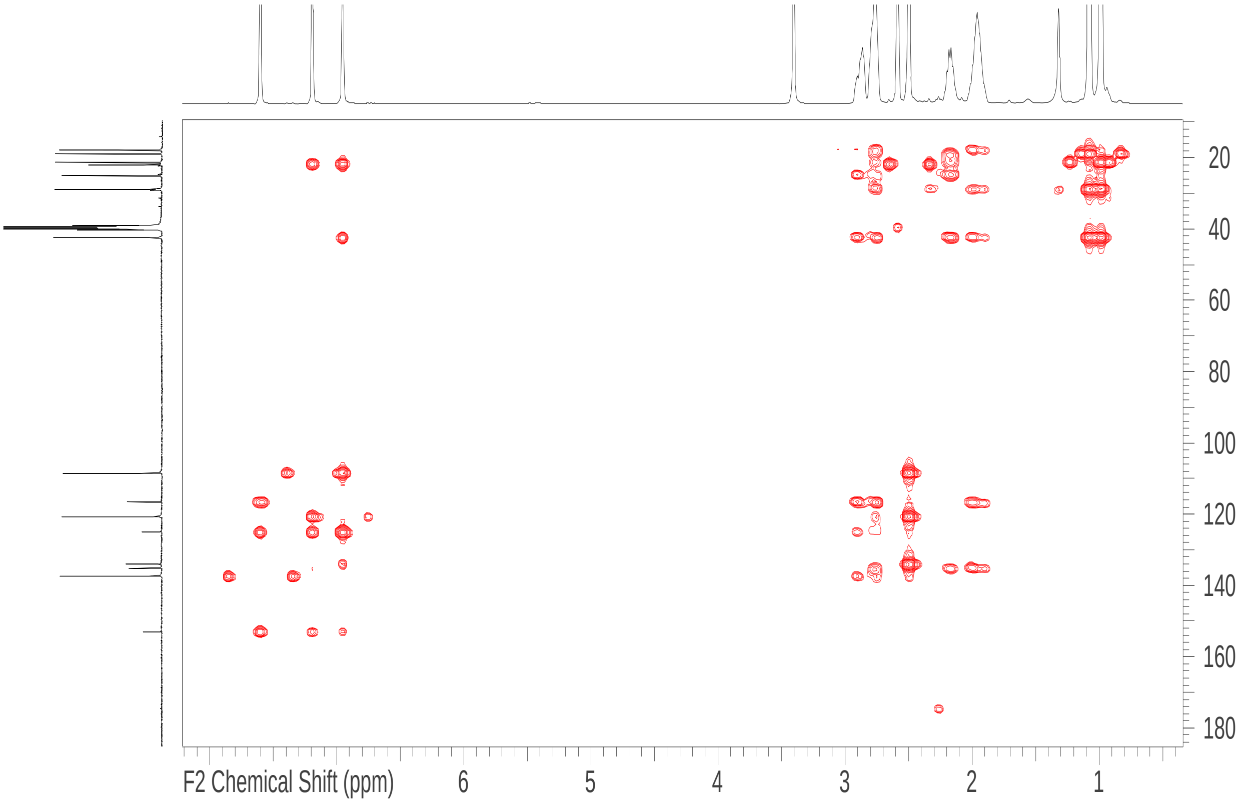

Figure S63 – HMBC NMR spectrum (400 MHz, $(CH_3)_2SO$) of **6**

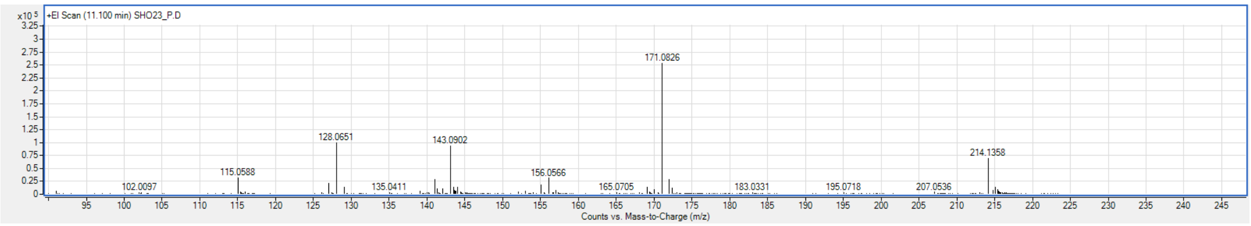

Figure S64 – GC-MS analysis of **6**

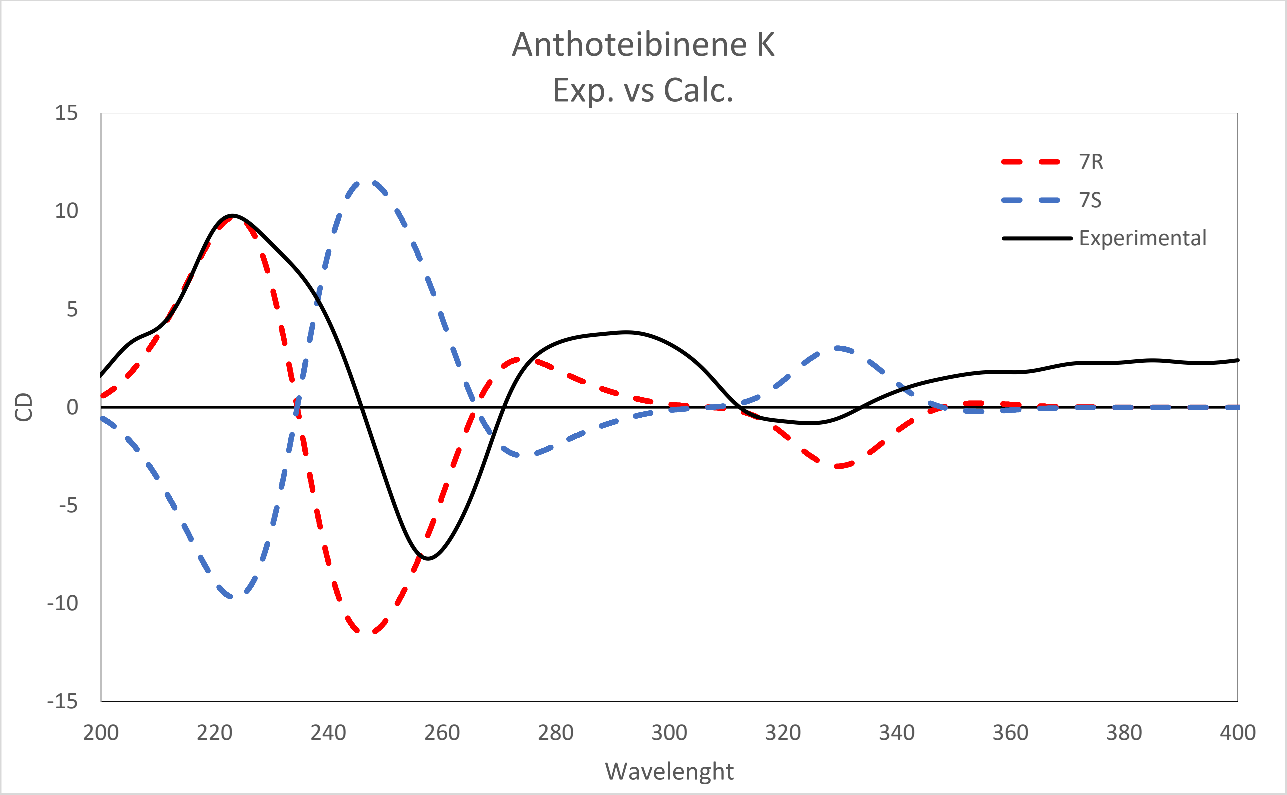

Figure S65 – ECD analysis of **6**

Table S7 – NMR Data for Anthoteibinene L (**7**) (600 (^{1}H) and 150 (^{13}C) MHz, $CDCl_3$)

pos	δ_C, type	δ_H	gCOSY	gHMBC	Key NOESY
**1**	140.0, C				
**2**	142.0, C				
**3**	177.4, C				
**4**	69.2, C				
**5**	78.6, CH	4.35, br d (2.9)	6	1, 3, 4	7, 11, 13, 15
**6**	36.7, C	3.49, dd (2.8, 11.6)	5, 7	1, 7	8b, 11, 13
**7**	39.9, CH	1.70, dddd (1.9, 2.2, 11.6, 11.6)	6, 8a, 8b, 11	6, 12, 13	5, 12
**8a**	22.3, CH_2	2.01, br dddd (1.9, 5.4, 11.6, 12.1)	7, 8b, 9a	6, 7, 9, 10, 11	12
**8b**		1.53, dddd (5.0, 11.6, 12.1, 12.6)	7, 8a, 9a, 9b	6, 7, 9	6, 13
**9a**	19.4, CH_2	2.83, br ddd (5.4, 12.6, 16.6)	8a, 8b, 9b	1, 7, 8, 10, 14	
**9b**		2.49, ddd (5.0, 12.1, 16.6)	8a, 8b, 9a	1, 8, 10, 14	
**10**	123.3, C				
**11**	26.3, CH	2.09, d sept (2.2, 6.9)	7, 12, 13	6, 7, 8, 12, 13	5, 6
**12**	21.2, CH_3	1.07, d (6.9)	11	7, 11, 13	7, 8a
**13**	15.8, CH_3	0.97, d (6.9)	11	7, 11, 12	5, 6, 8b
**14**	145.2, CH_3	7.45, s	9a, 9b	1, 2, 10	
**15**	22.8, CH_3	1.88, s		3, 4, 5	5

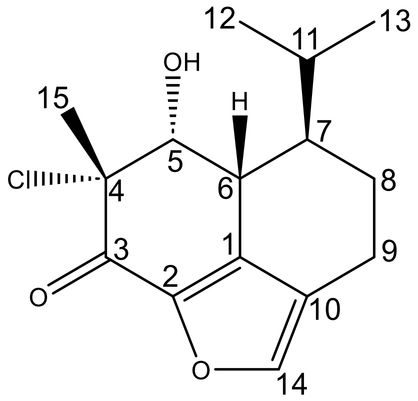

Figure S66 – Structure of **7**

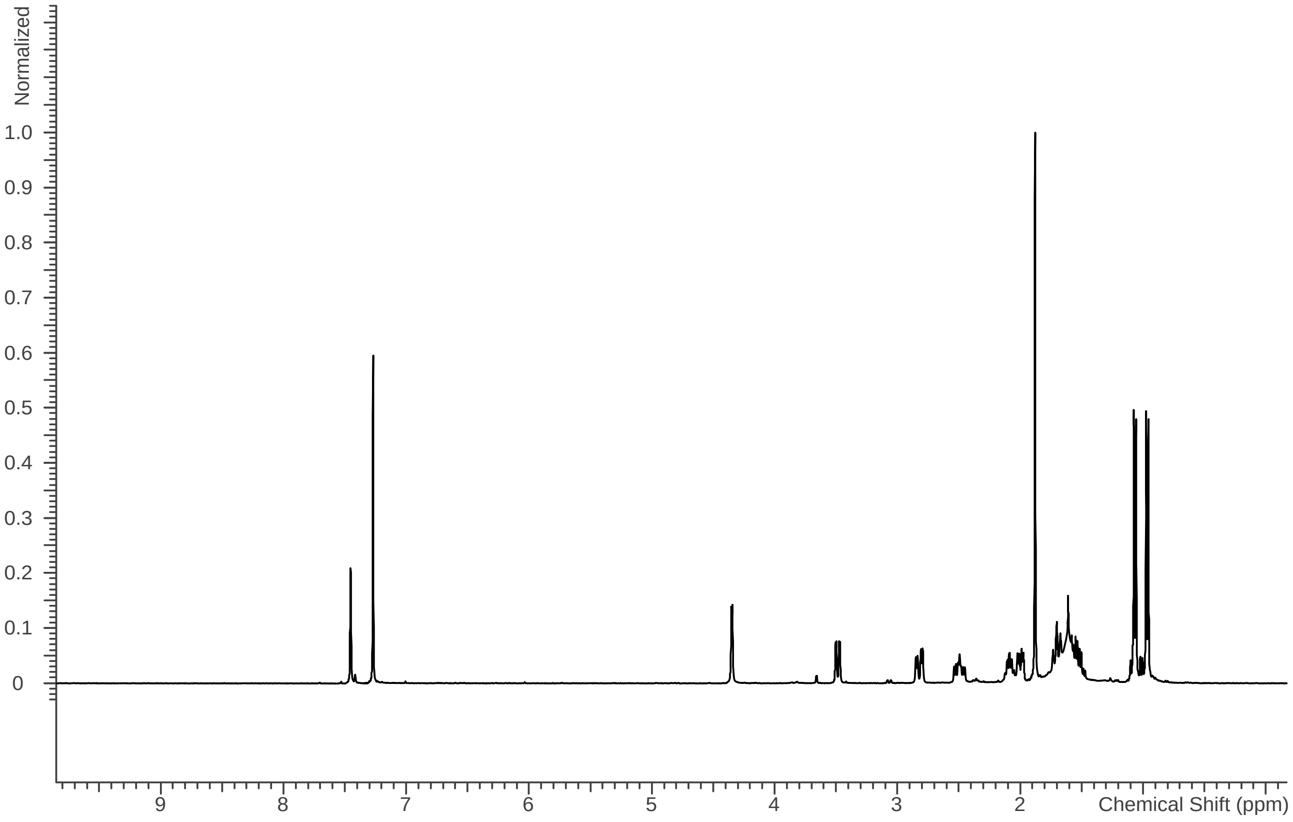

Figure S67 – ^{1}H NMR spectrum (600 MHz, $CDCl_3$) of **7**

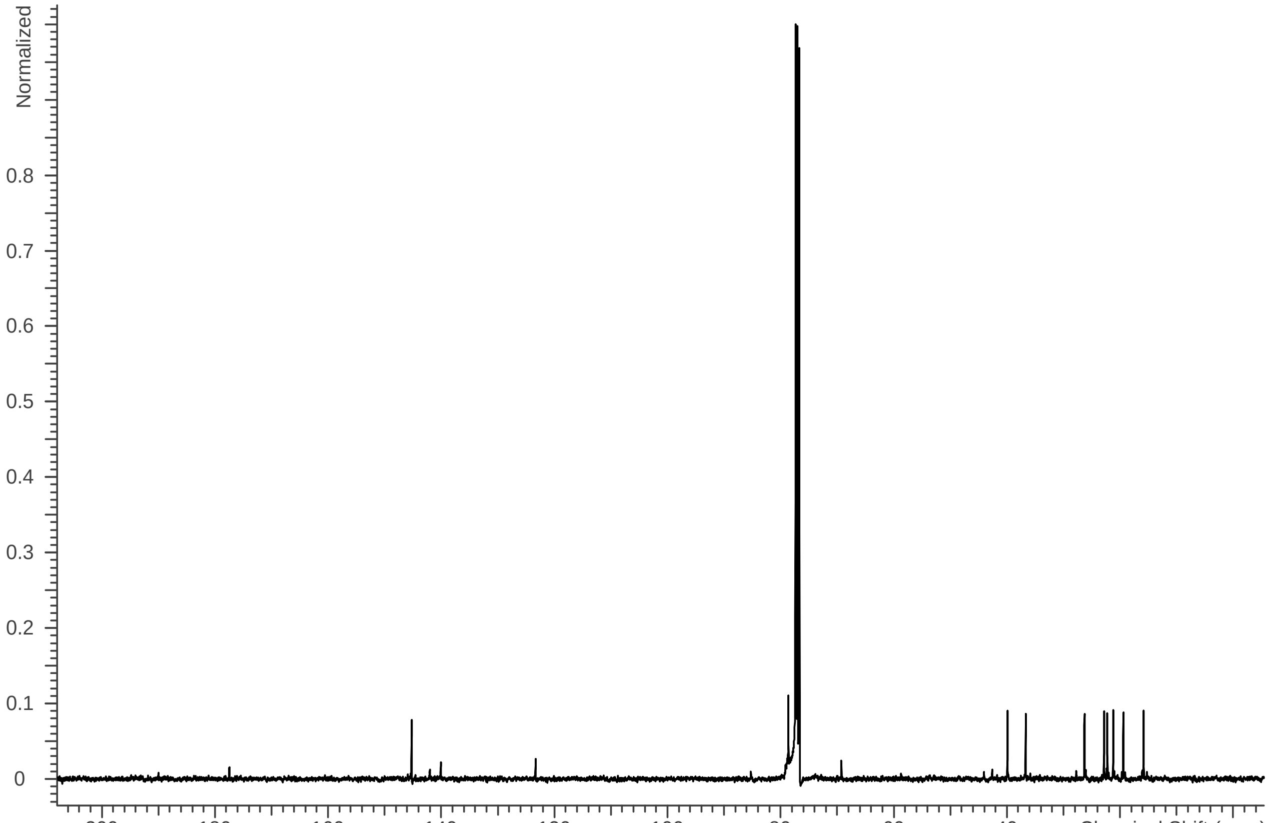

Figure S68 – ^{13}C NMR spectrum (150 MHz, $CDCl_3$) of **7**

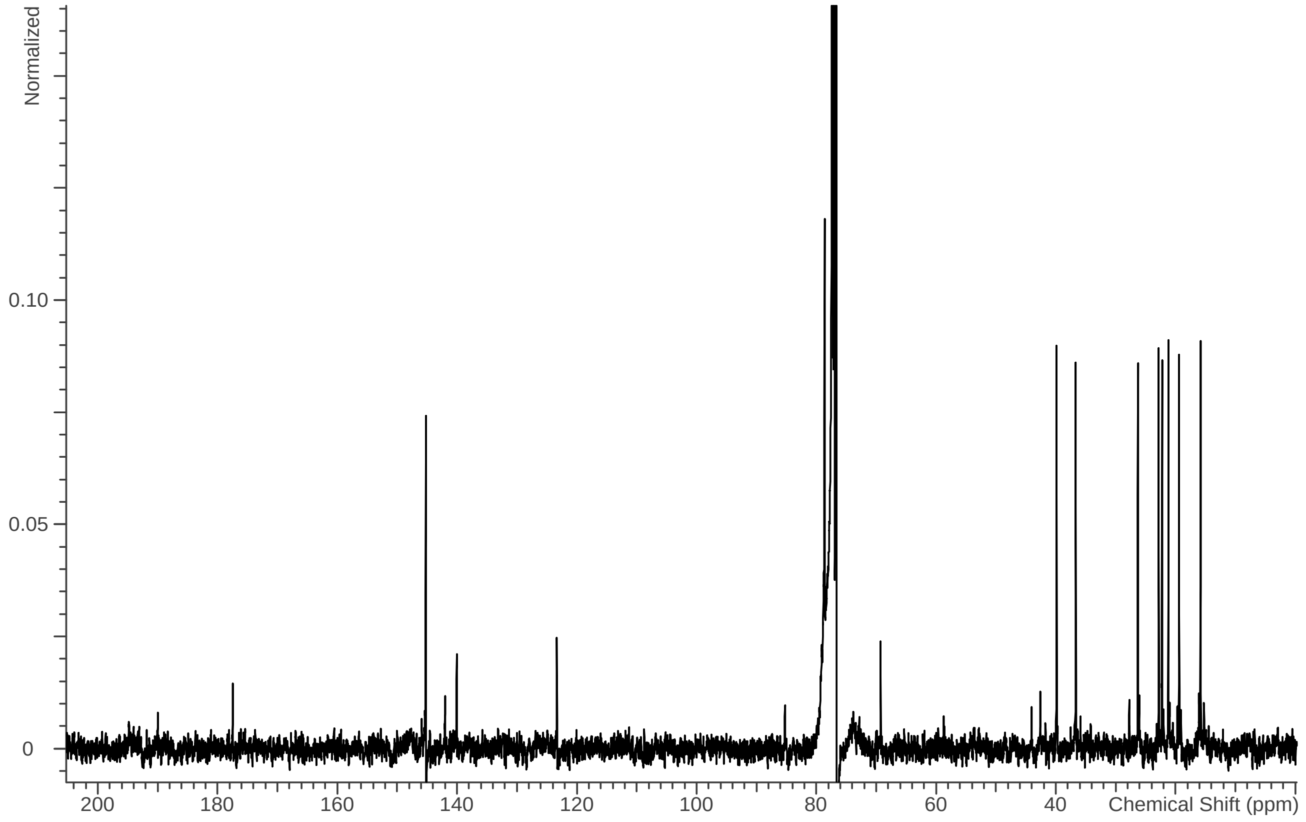

Figure S69 – ^{13}C NMR spectrum zoomed (150 MHz, $CDCl_3$) of **7**

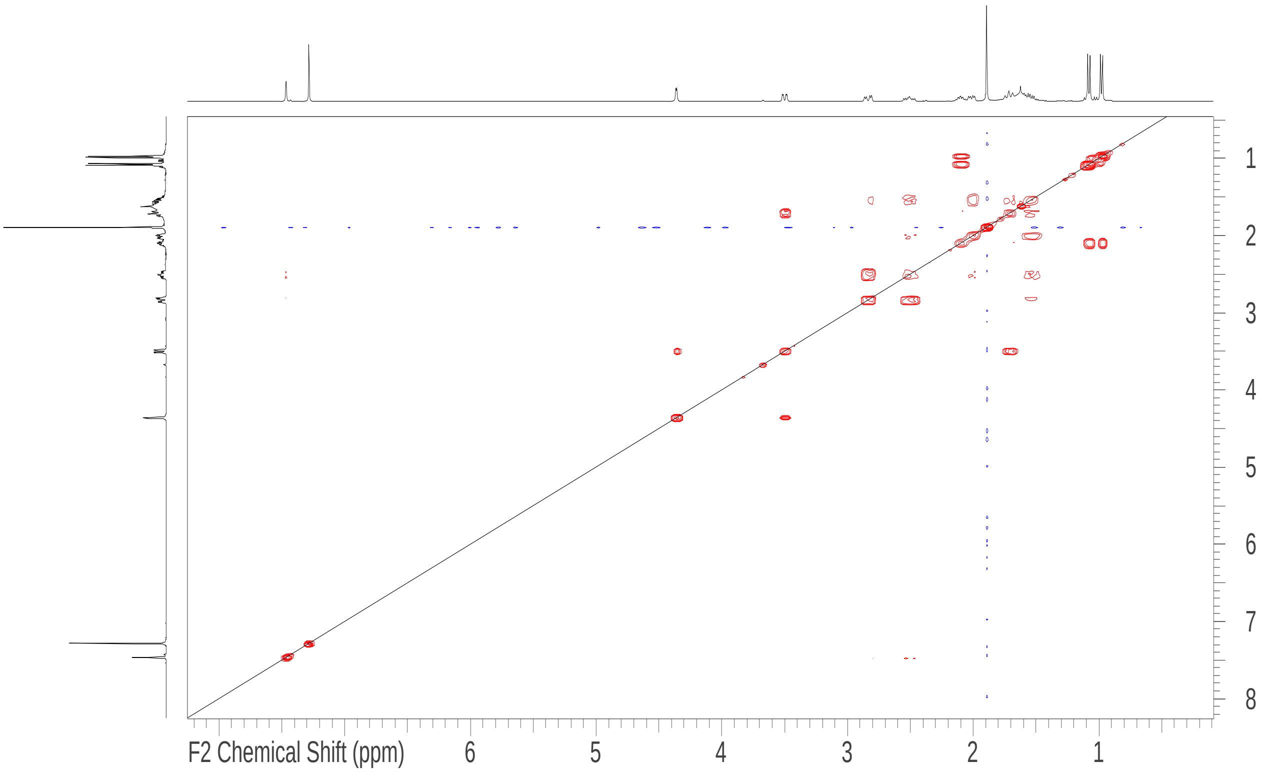

Figure S70 – COSY NMR spectrum (600 MHz, $CDCl_3$) of **7**

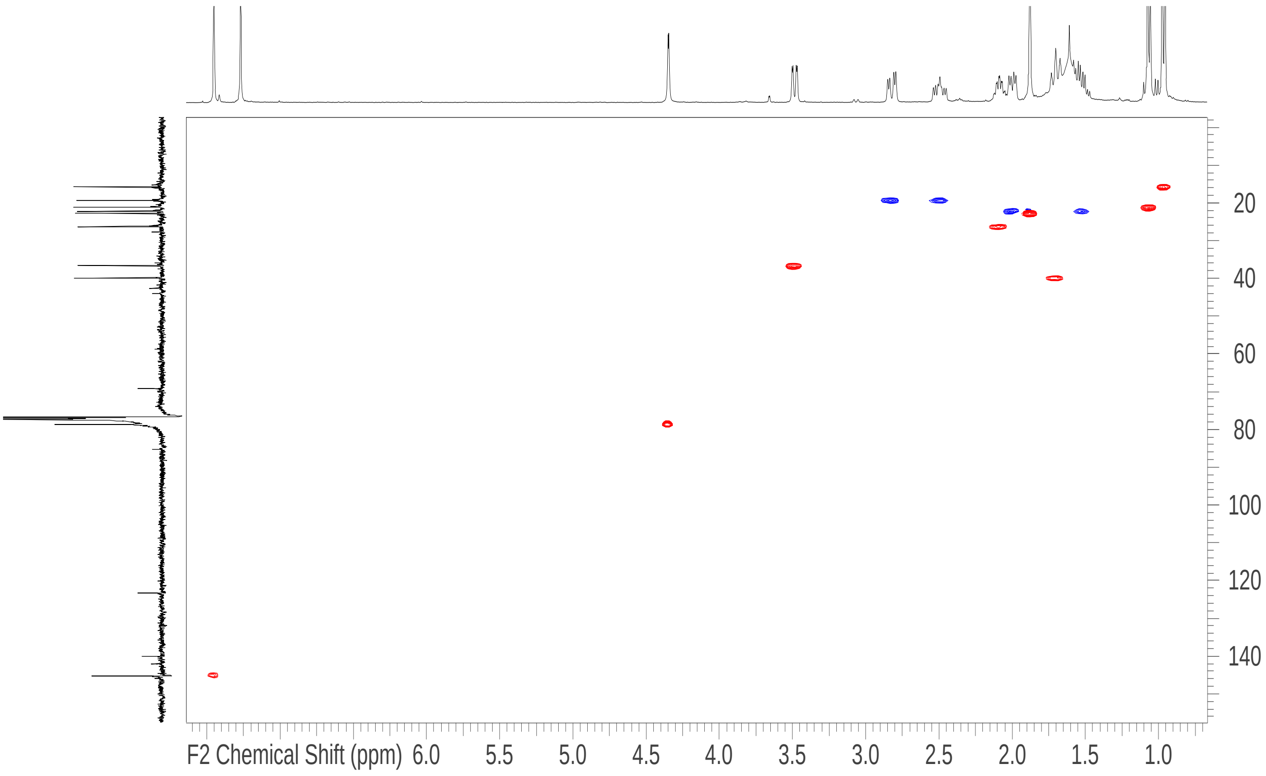

Figure S71 – HSQC NMR spectrum (600 MHz, $CDCl_3$) of **7**

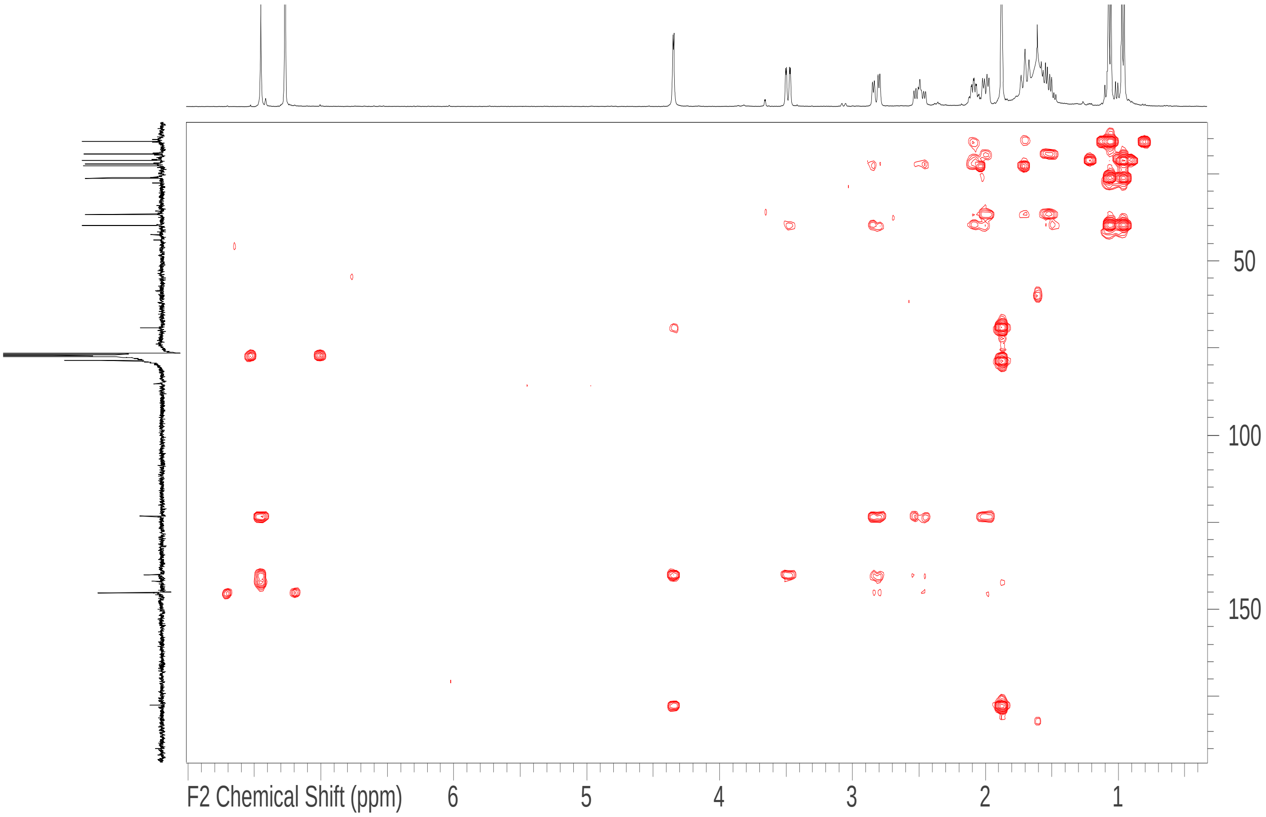

Figure S72 – HMBC NMR spectrum (600 MHz, $CDCl_3$) of **7**

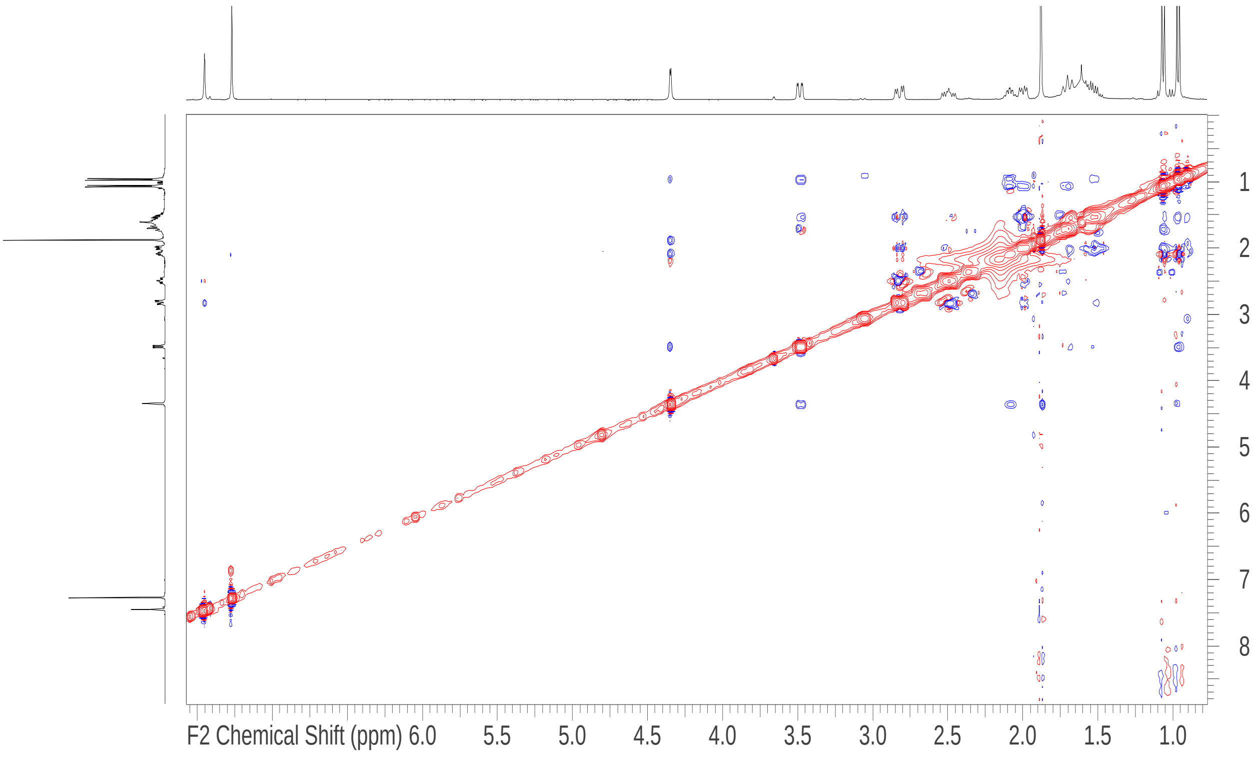

Figure S73 – NOESY NMR spectrum (600 MHz, $CDCl_3$) of **7**

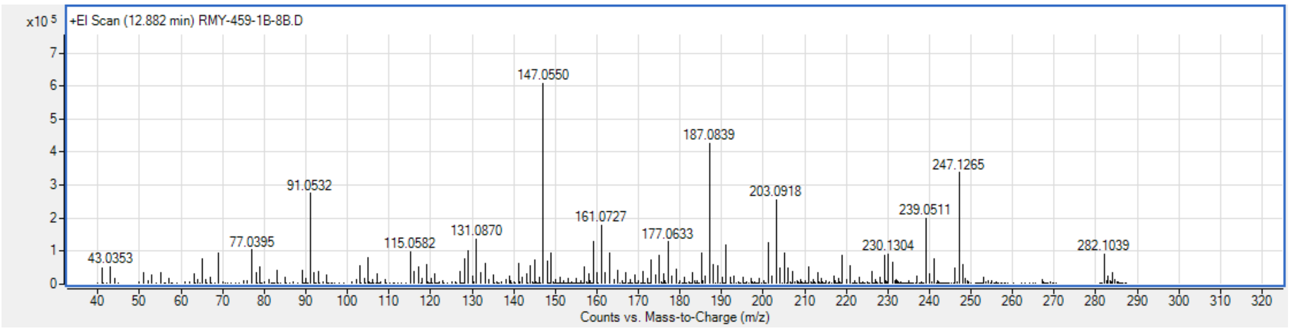

Figure S74 – HREIMS analysis of **7**

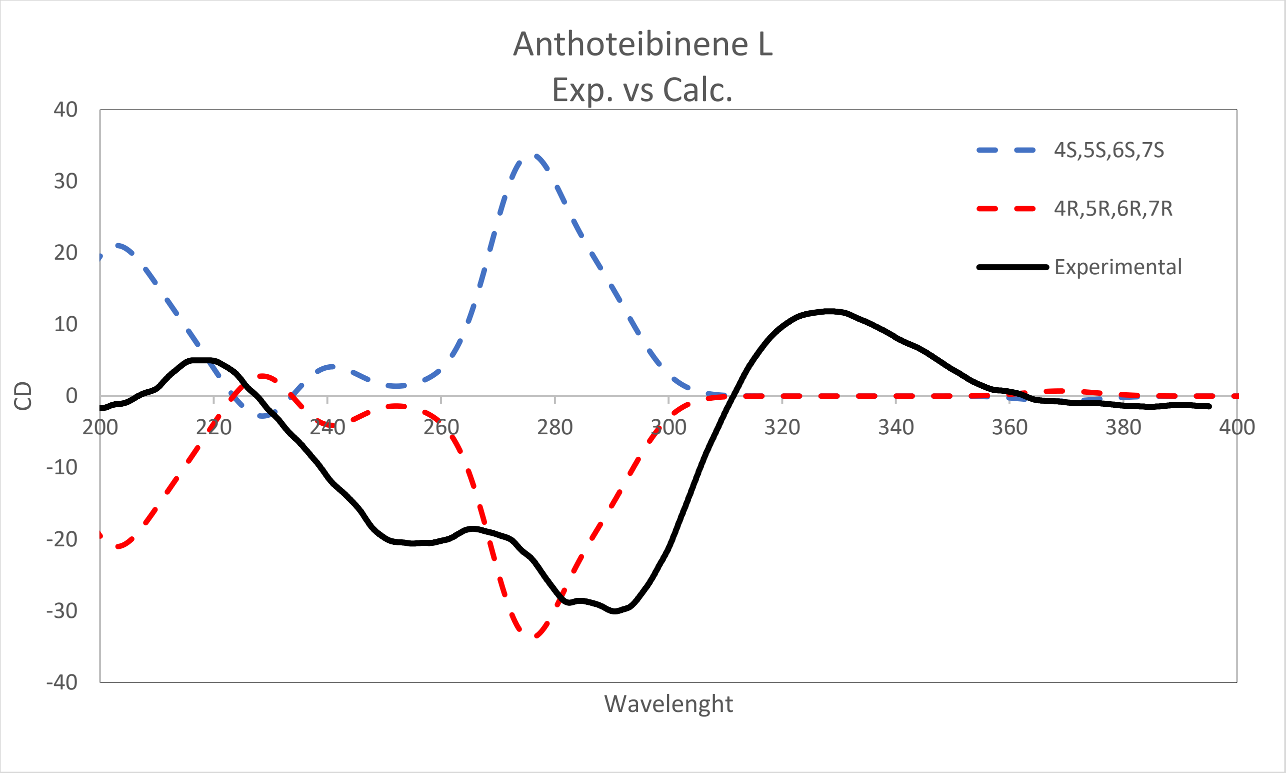

Figure S75 – ECD analysis of **7**

Table S8 – NMR Data for Anthoteibinene M (**8**) (600 (^{1}H) and 150 (^{13}C) MHz, $CDCl_3$)

pos	δ$_C$, type	δ$_H$	gCOSY	gHMBC	Key NOESY
**1**	137.0, C				
**2**	190.6, C				
**3**	127.0, CH	6.05, s	5b, 15	2, 4, 5, 15	
**4**	165.0, C				
**5a**	37.5, CH_2	2.59, dd (5.3, 17.8)*	5b, 6	1, 3, 4, 6, 7, 15	11
**5b**		2.18, dd (11.3, 17.8)	3, 5a	3, 4, 6, 7	6
**6**	39.3, CH	2.65, o/l*	5a, 7*	10	5b, 13
**7**	44.9, CH	1.34, o/l*	6, 11		
**8a**	19.9, CH_2	1.75, br m	8b, 9	6, 10, 11	12
**8b**		1.31, o/l*	8a	7	
**9**	28.7, CH_2	2.53, o/l*	8a	7, 8, 10	
**10**	140.4, C				
**11**	27.1, CH		7, 12, 13	6, 12, 13	5a
**12**	21.5, CH_3	1.90, dsept (2.0, 6.7)	11	7, 11, 13	8a
**13**	16.2, CH_3	1.01, d (6.7)	11	7, 11, 12	6
**14**	171.4, C	0.82, d (6.7)			
**15**	24.5, CH_3		3	2, 3, 4, 5	
		2.03, s			

*Overlapping ^{1}H NMR signals, 2D assignments based on proximity likelihood

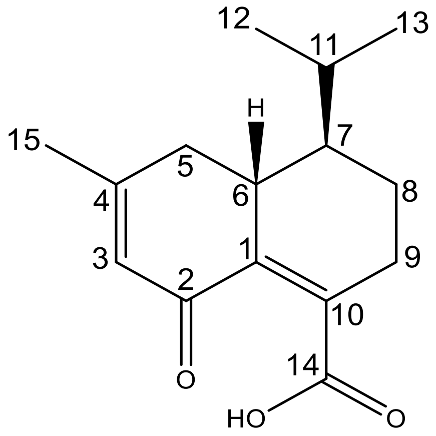

Figure S76 – Structure of **8**

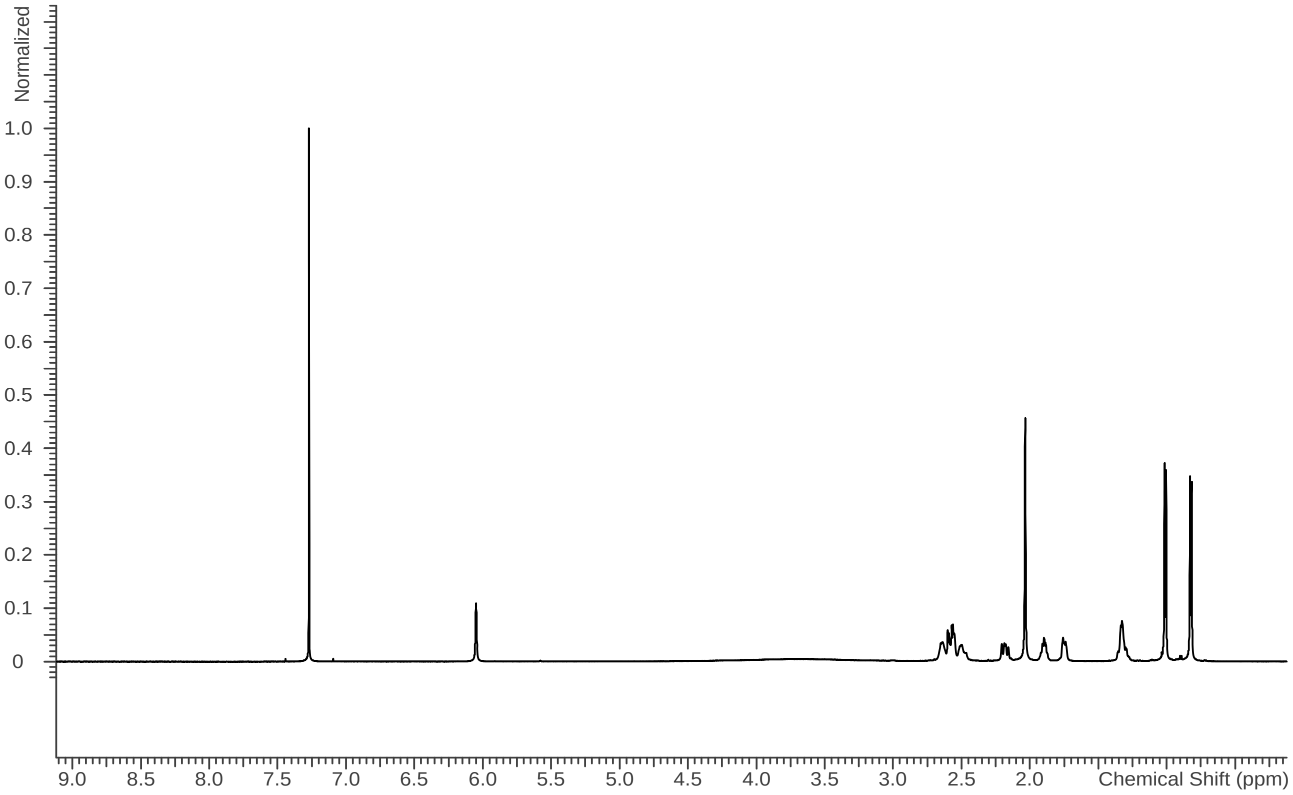

Figure S77 – ^{1}H NMR spectrum (600 MHz, $CDCl_3$) of **8**

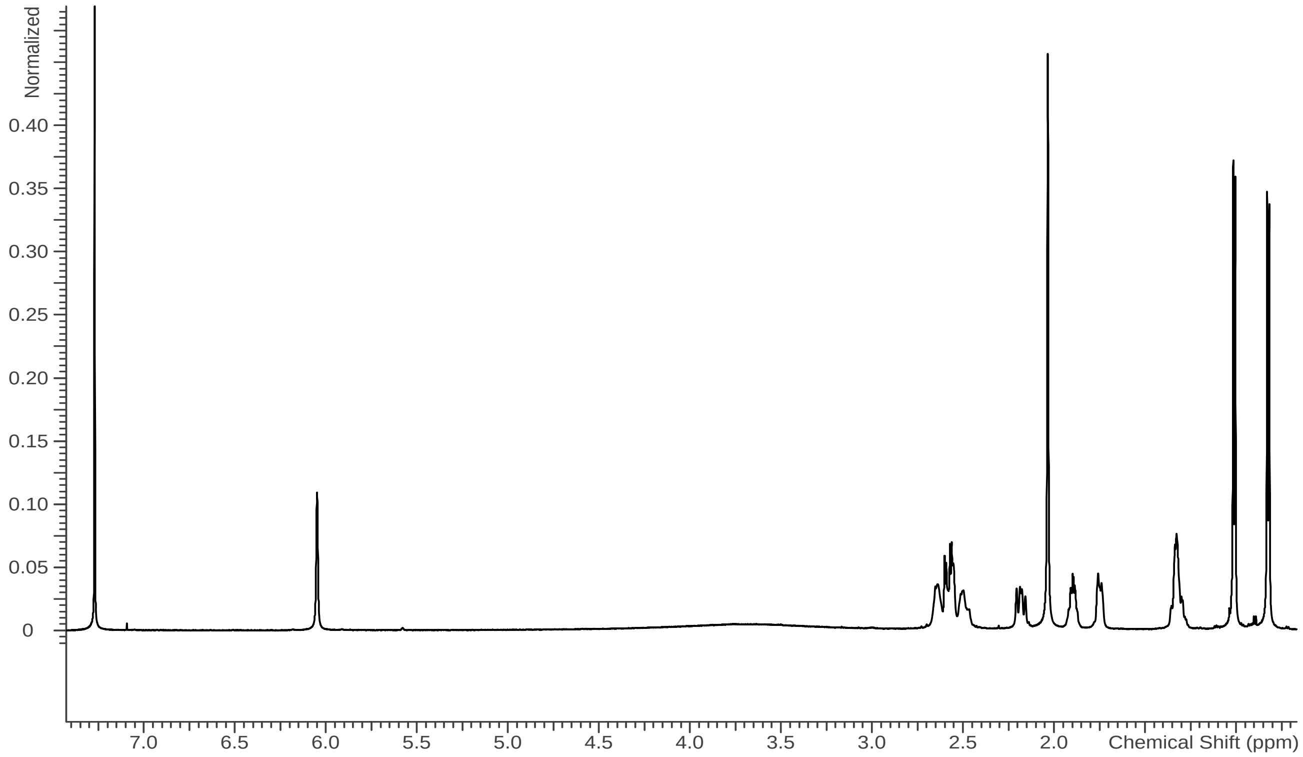

Figure S78 – ^{1}H NMR spectrum zoomed (600 MHz, $CDCl_3$) of **8**

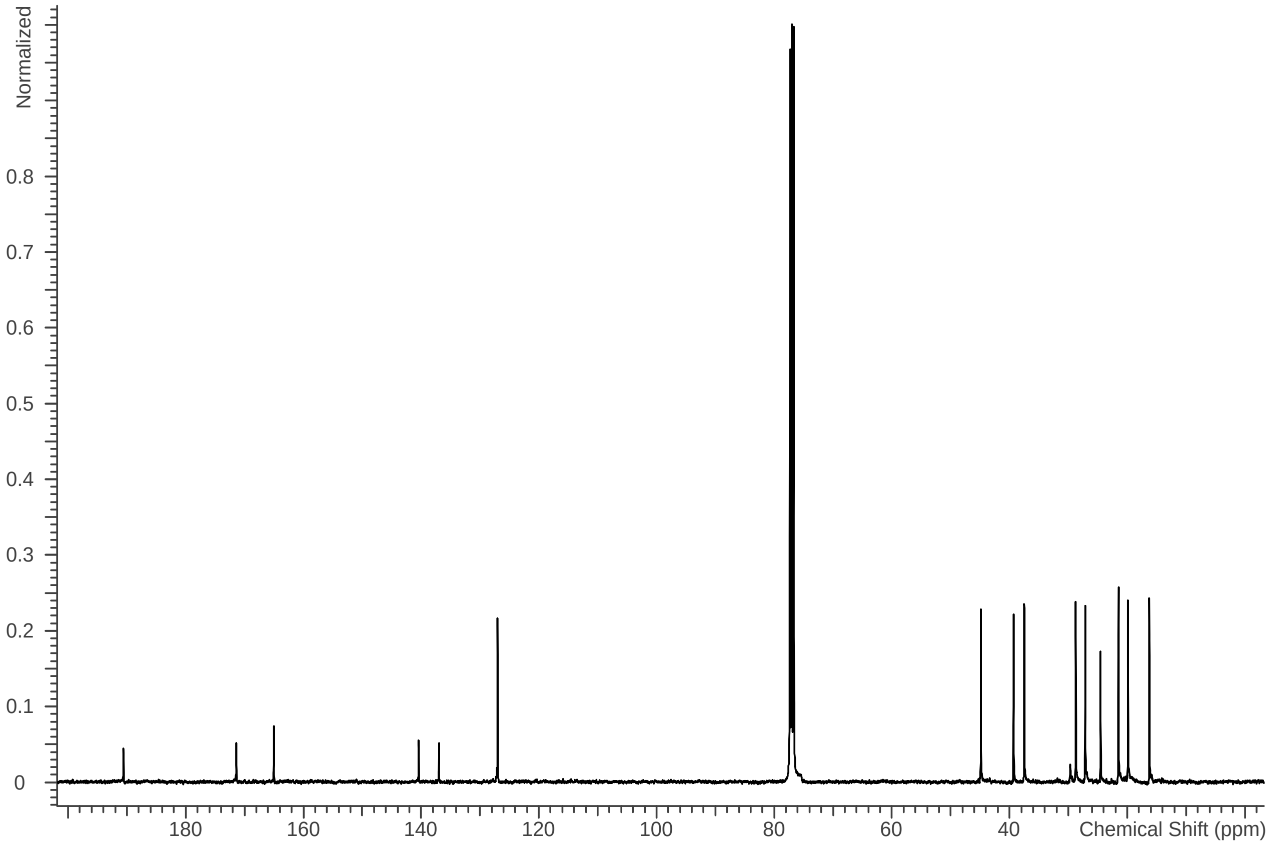

Figure S79 – ^{13}C NMR spectrum (150 MHz, $CDCl_3$) of **8**

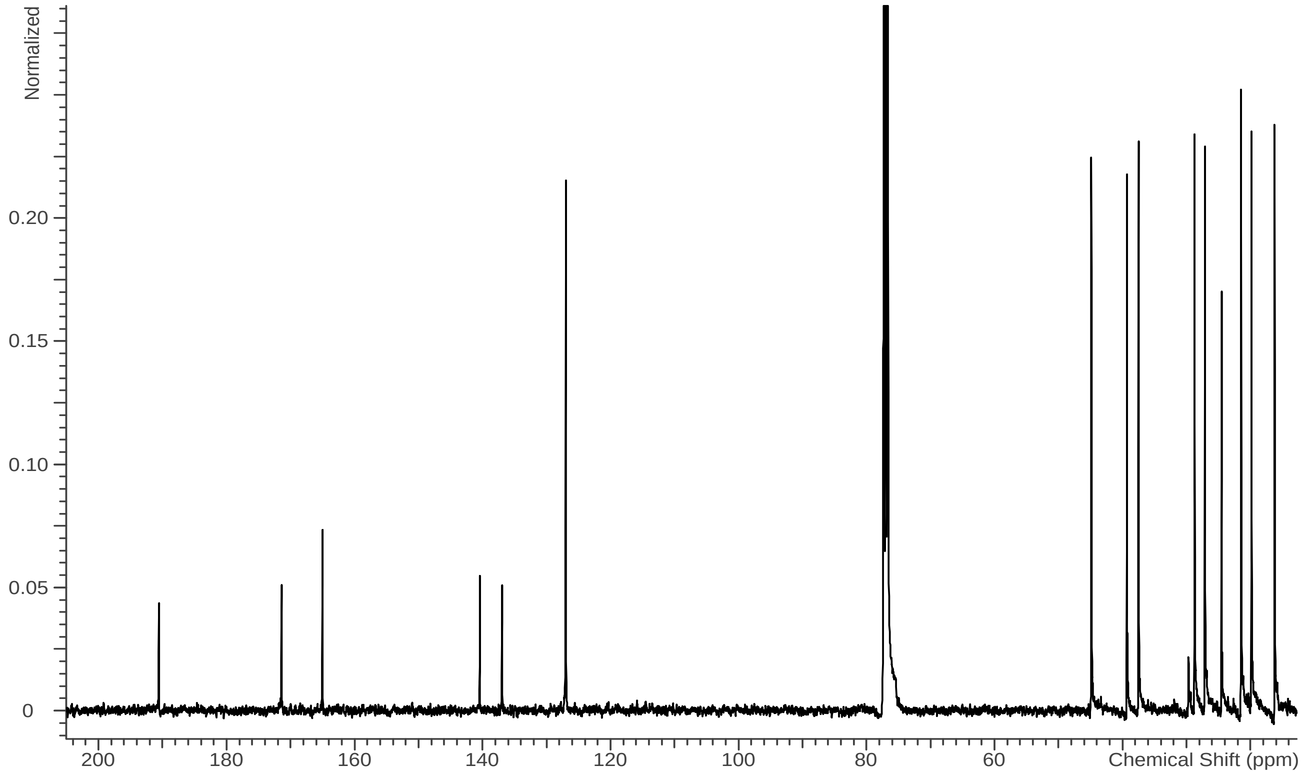

Figure S80 – ^{13}C NMR spectrum zoomed (150 MHz, $CDCl_3$) of **8**

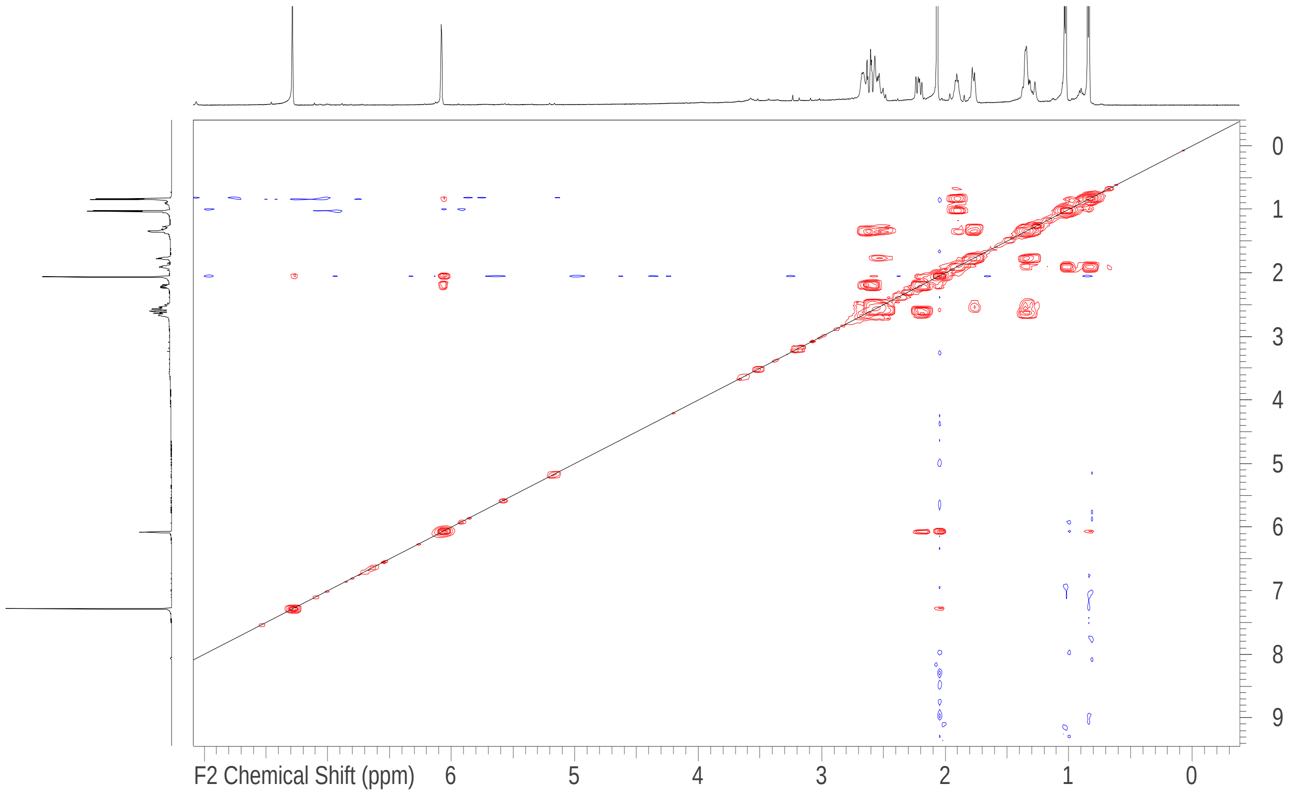

Figure S81 – COSY NMR spectrum (600 MHz, $CDCl_3$) of **8**

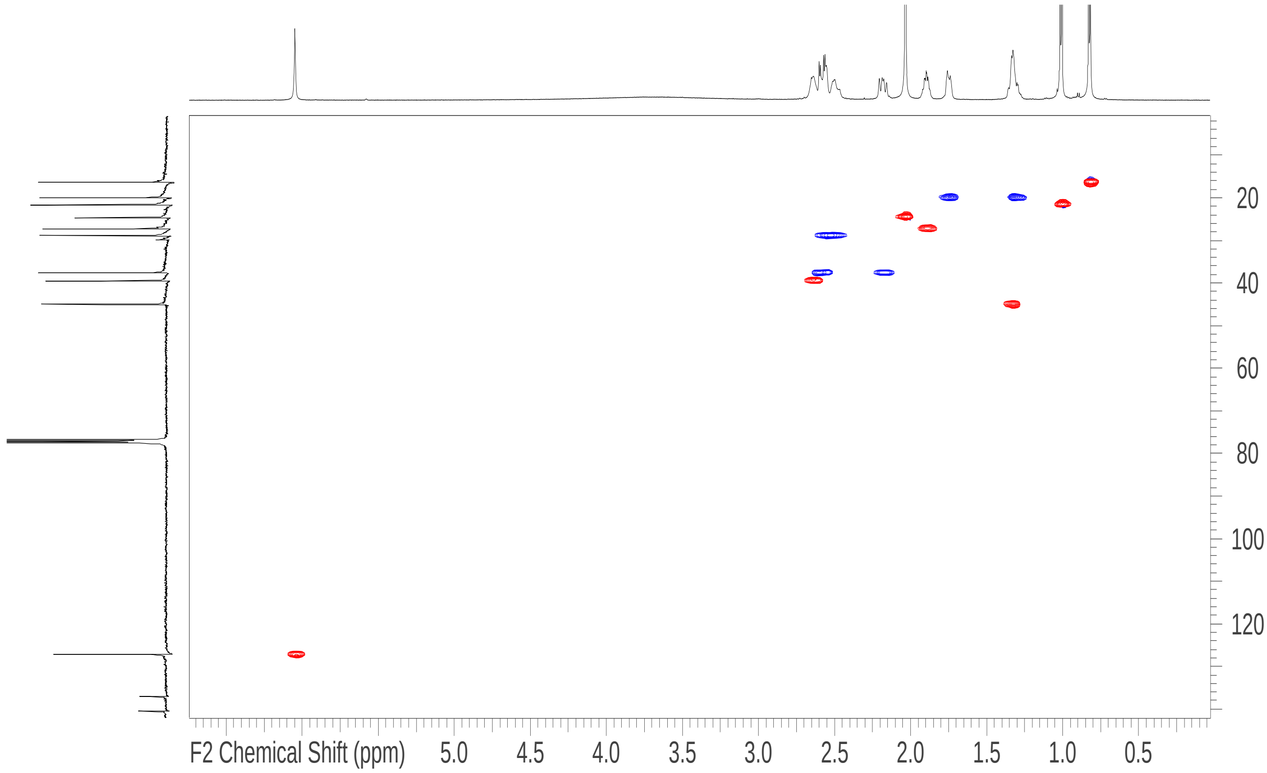

Figure S82 – HSQC NMR spectrum (600 MHz, $CDCl_3$) of **8**

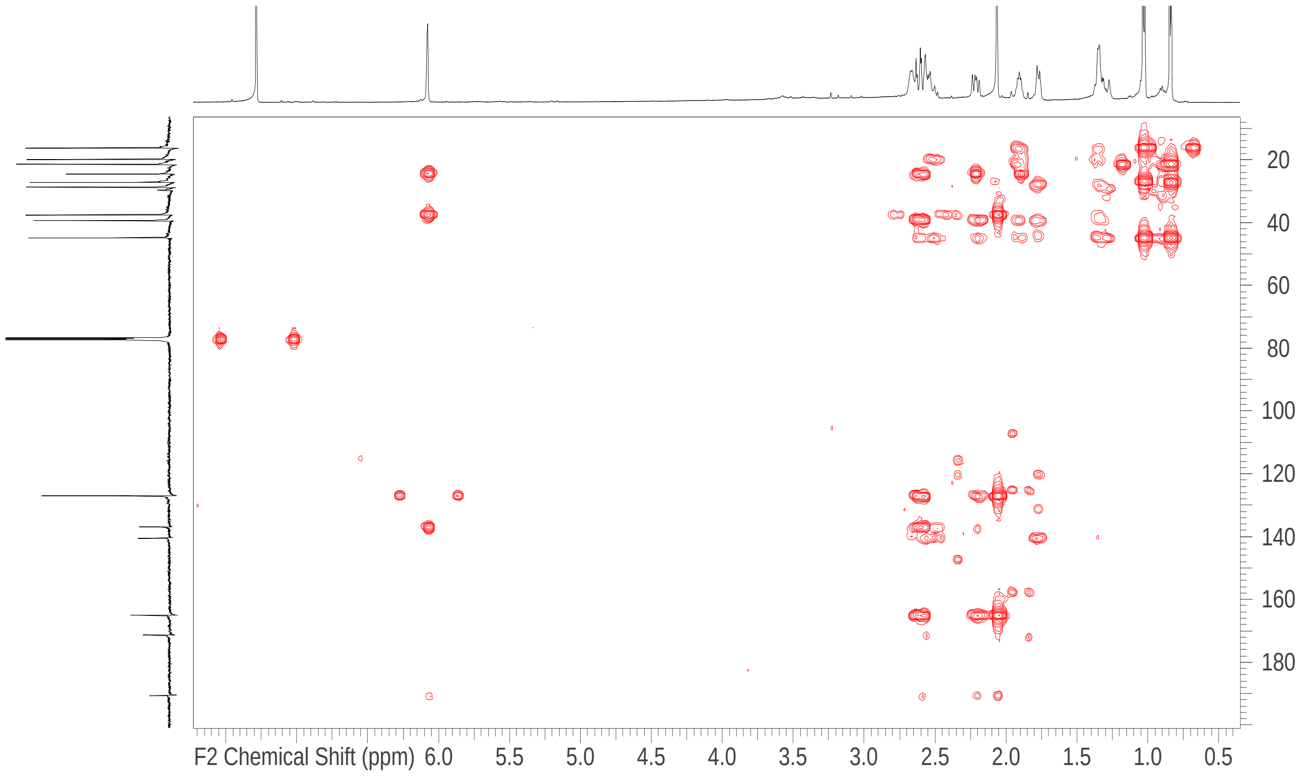

Figure S83 – HMBC NMR spectrum (600 MHz, $CDCl_3$) of **8**

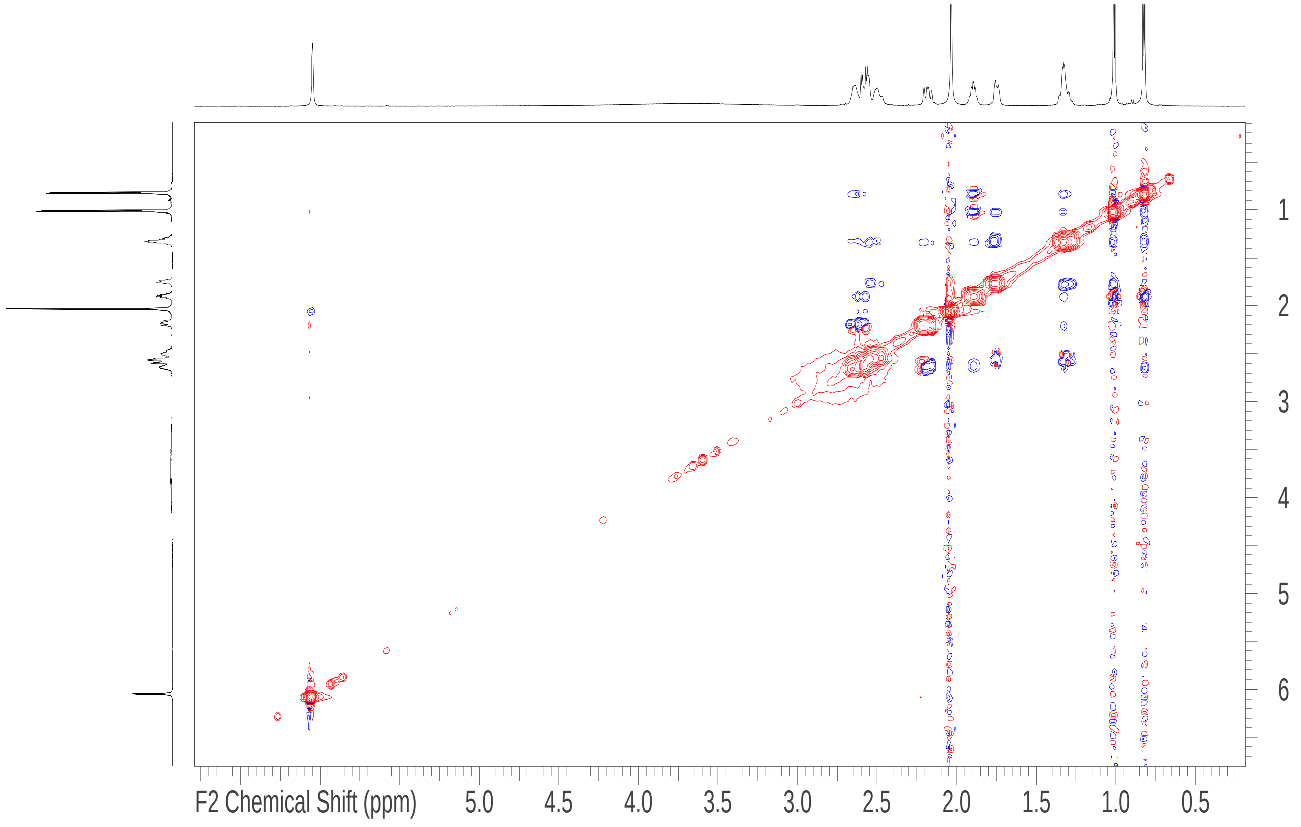

Figure S84 – NOESY NMR spectrum (600 MHz, $CDCl_3$) of **8**

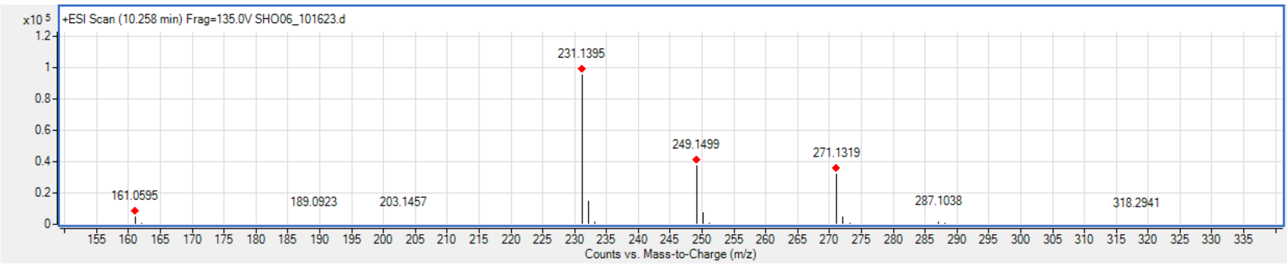

Figure S85 – HRESIMS analysis of **8**

Table S9 – Crystal Data and Structure Refinement for Anthoteibinene M (**8**)

Identification code	Anthoteibinene M
Empirical formula	$C_{30}H_{45}NaO_9$
Moiety formula	$(C_{15}H_{20}O_3)(C_{15}H_{19}O_3)^{-}Na^{+}(H_2O)_3$
Formula weight	572.65
Temperature/K	100.00
Crystal system	monoclinic
Space group	C2
a/Å	30.6022(7)
b/Å	6.08140(10)
c/Å	21.1089(5)
α/°	90
β/°	129.2120(10)
γ/°	90
Volume/Å^3	3043.81(12)
Z	4
ρ_{calc}g/cm^3	1.250
μ/mm^{-1}	0.868
F(000)	1232.0
Crystal size/mm^3	0.12 × 0.04 × 0.02
Radiation	CuKα (λ = 1.54178)
2Θ range for data collection/°	5.404 to 159.048
Index ranges	-37 ≤ h ≤ 38, -7 ≤ k ≤ 7, -26 ≤ l ≤ 26
Reflections collected	42098
Independent reflections	6351 [R_{int} = 0.0698, R_{sigma} = 0.0427]
Data/restraints/parameters	6351/1/395
Goodness-of-fit on F^2	1.031
Final R indexes [I>=2σ (I)]	R_1 = 0.0372, wR_2 = 0.0921
Final R indexes [all data]	R_1 = 0.0441, wR_2 = 0.0968
Largest diff. peak/hole / e Å^{-3}	0.26/-0.28

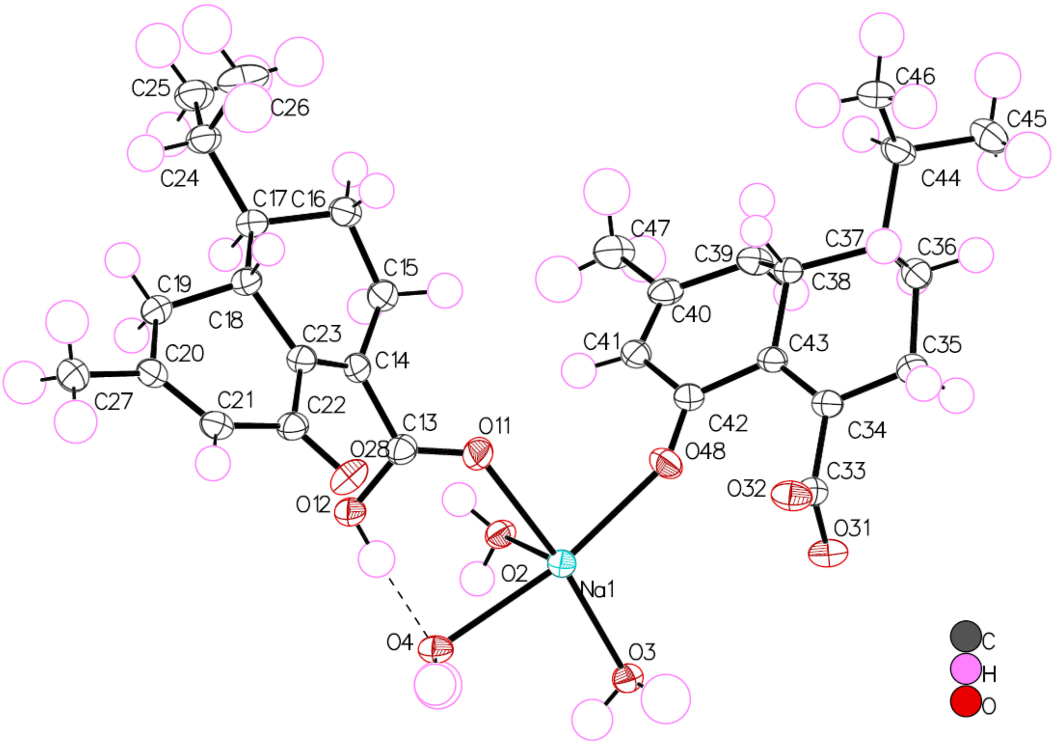

Figure S86 – Ellipsoid plot of **8**. Anisotropic displacement parameters were drawn at 50% probability level.

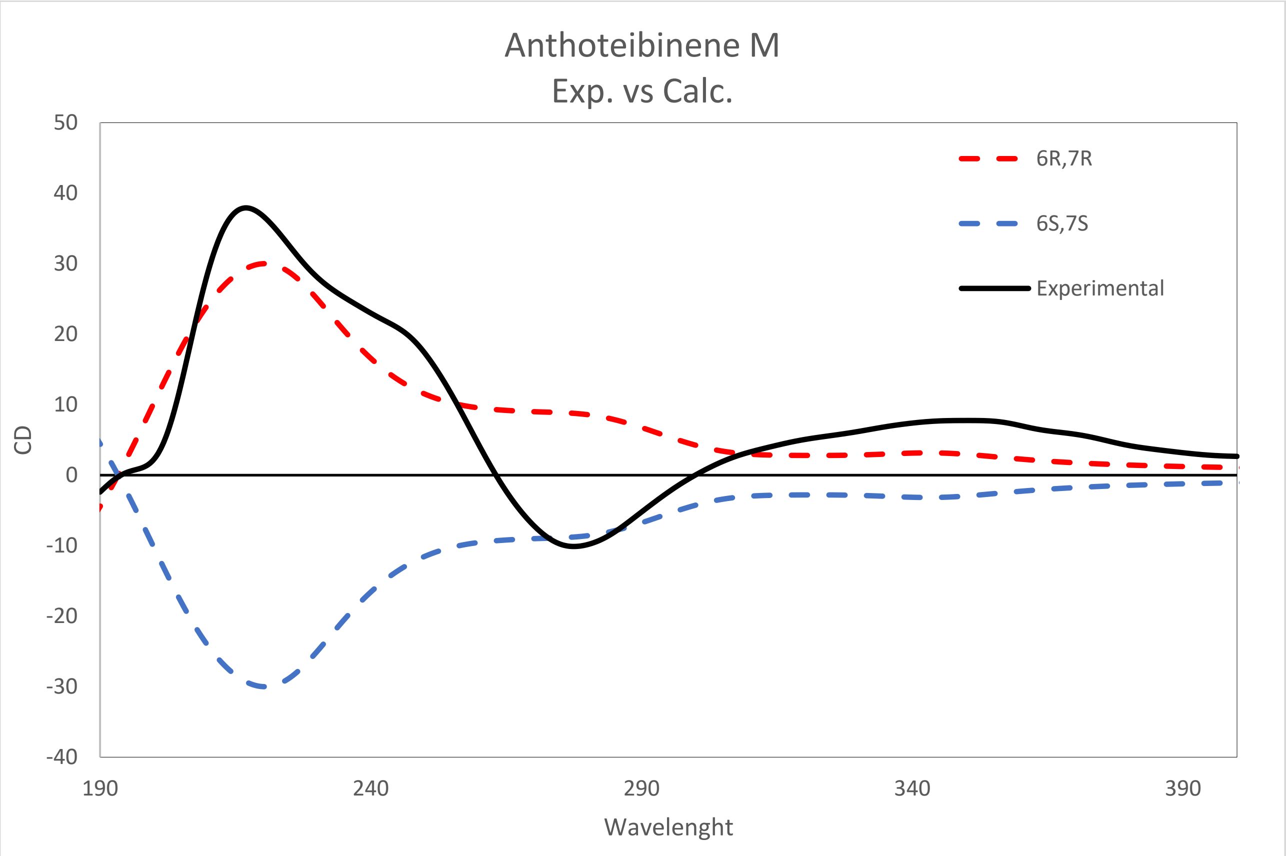

Figure S87 – ECD analysis of **8**

Table S10 – NMR Data for Anthoteibinene N (**9**) (400 (^{1}H) and 100 (^{13}C) MHz, $CDCl_3$)

pos	δ$_C$, type	δ$_H$ (*J* in Hz)	gCOSY	gHMBC	Key NOESY
**1**	47.1, CH	3.63, br d (3.9)	6	2, 6, 7, 9, 10, 14	5a, 7
**2**	199.3, C				
**3**	125.6, CH	5.89, s	5a, 15	1, 5, 15	
**4**	159.3, C				
**5a**	32.2, CH_2	2.62, br d (18.5)	3, 5b, 6, 15	3, 4, 7	
**5b**		2.48, d (18.5)	5a, 6	1, 3, 4, 6, 15	11, 13
**6**	36.8, CH	2.18, o/l*	1, 5a, 5b, 7	1, 2, 4, 5	13
**7**	35.1, CH	1.67, dddd (4.6, 4.6, 11.0, 11.0)	6, 8b, 11	1, 6, 11, 13	12
**8a**	25.2, CH_2	2.22, o/l*	8b, 9	6, 9, 10	12
**8b**		1.97, o/l*	7, 8a, 9	6, 9, 10, 11	8b
**9**	141.4, CH	7.10, br s	8a, 8b	1, 7, 8, 14	
**10**	129.3, C				
**11**	27.1, CH	1.83, octet (6.6)	7, 12, 13	12, 13	5b
**12**	20.6, CH_3	0.89, d (6.9)	11	7, 11, 13	7, 8a
**13**	14.1, CH_3	0.78, d (6.8)	11	7, 11, 12	5b, 6, 8b
**14**	170.9, C				
**15**	24.5, CH_3	1.99, s*	3, 5a	3, 4, 5	

*Overlapping ^{1}H NMR signals, 2D assignments based on proximity likelihood

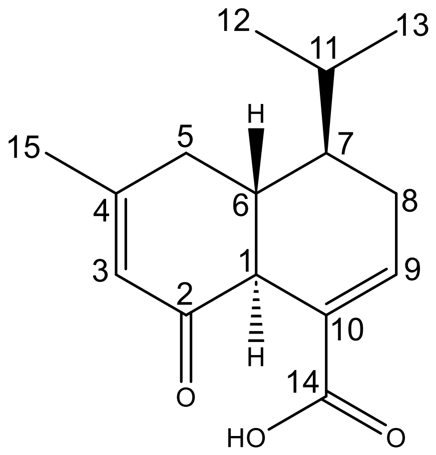

Figure S88 – Structure of **9**

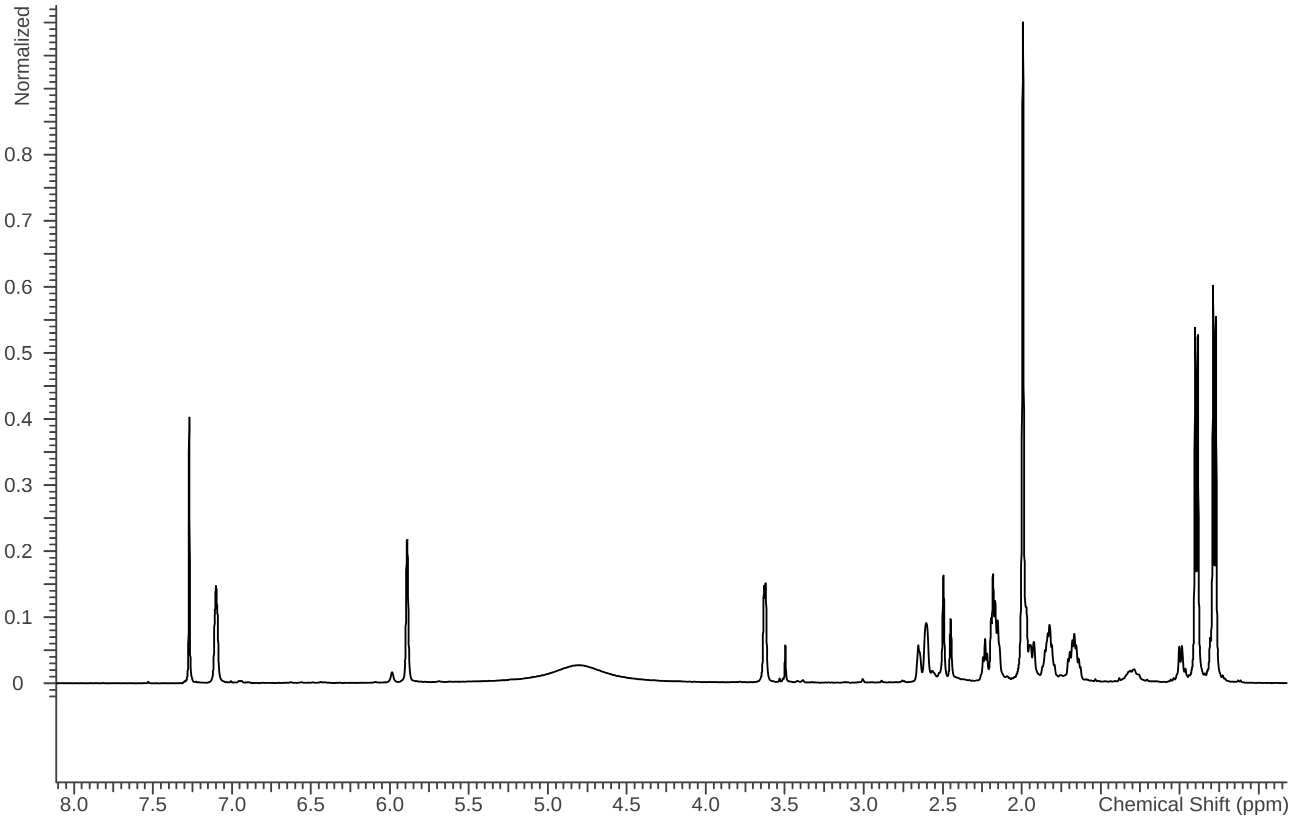

Figure S89 – ^{1}H NMR spectrum (400 MHz, $CDCl_3$) of **9**

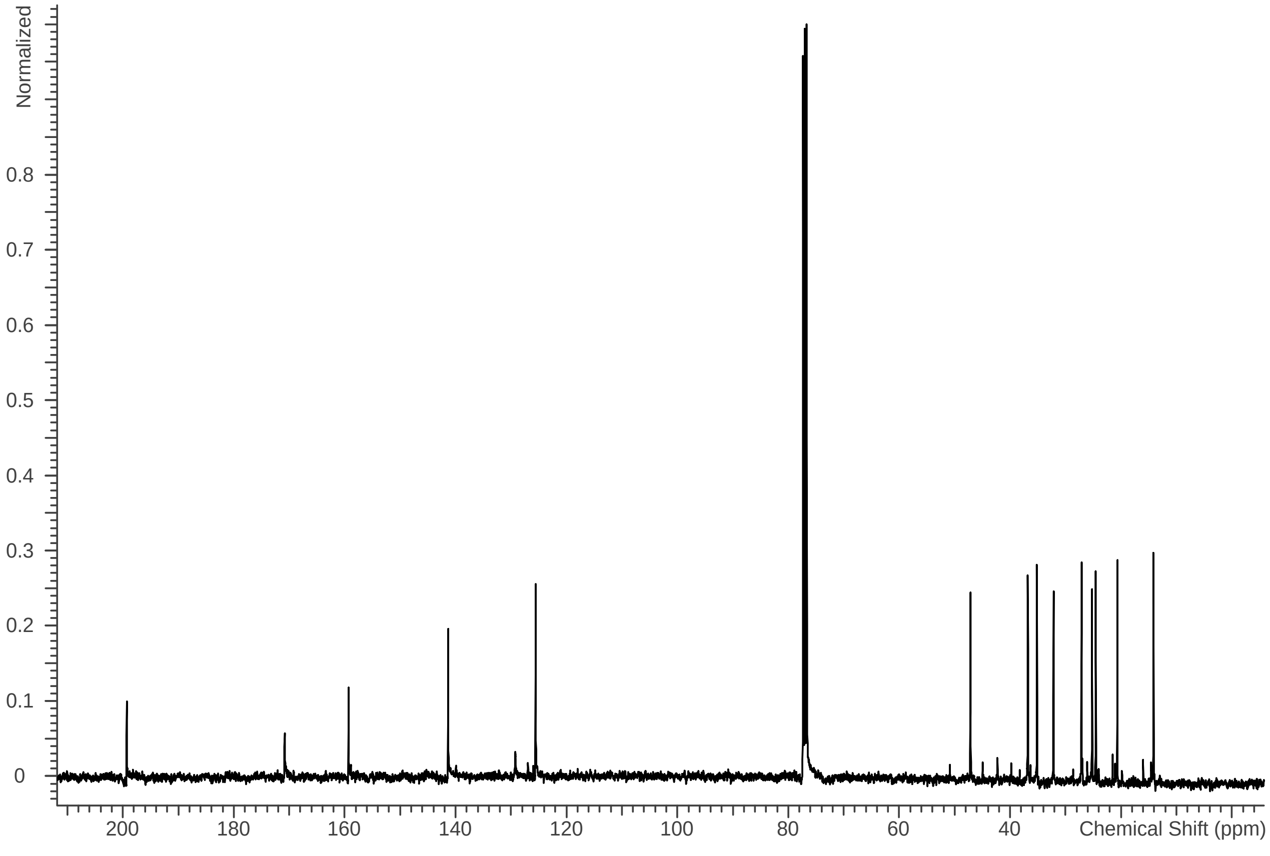

Figure S90 – ^{13}C NMR spectrum (100 MHz, $CDCl_3$) of **9**

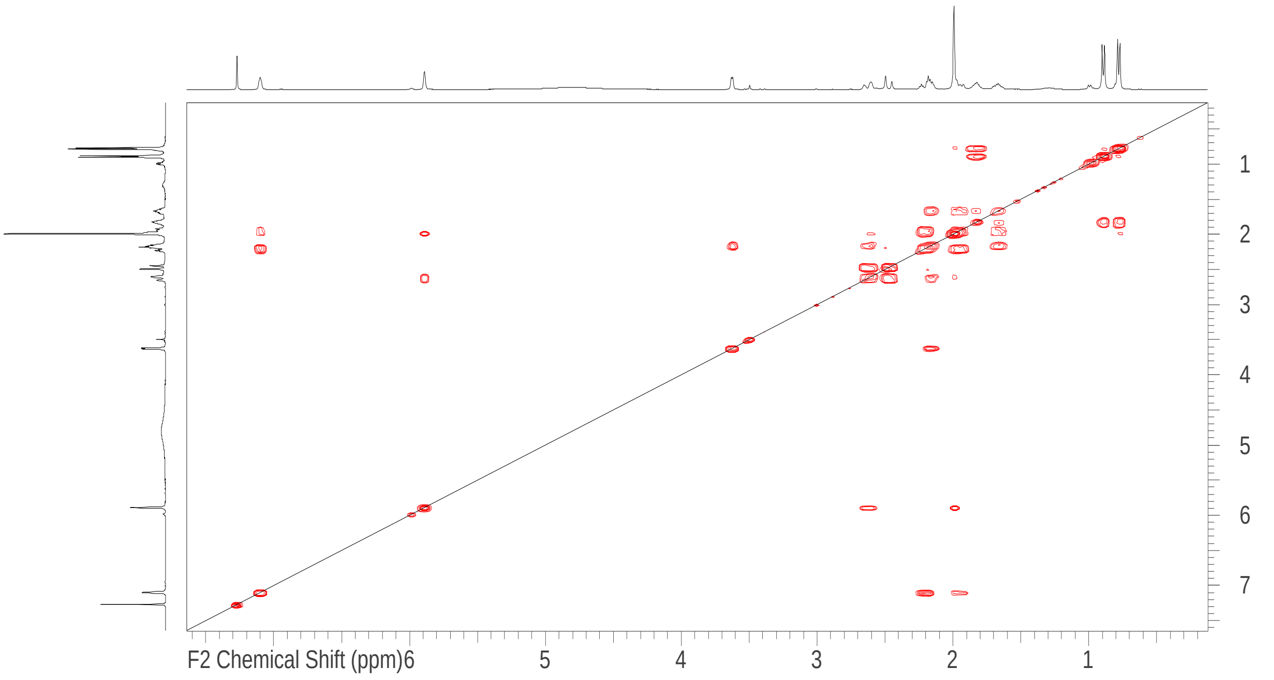

Figure S91 – COSY NMR spectrum (400 MHz, $CDCl_3$) of **9**

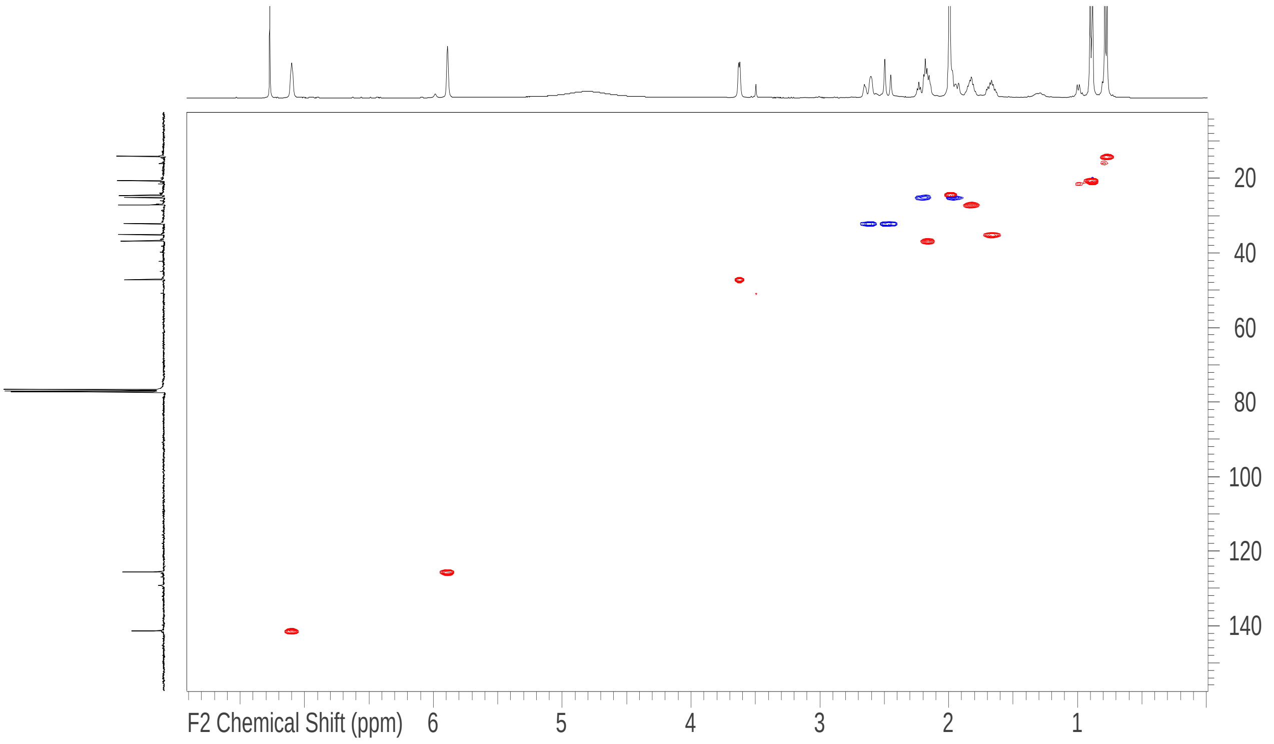

Figure S92 – HSQC NMR spectrum (400 MHz, $CDCl_3$) of **9**

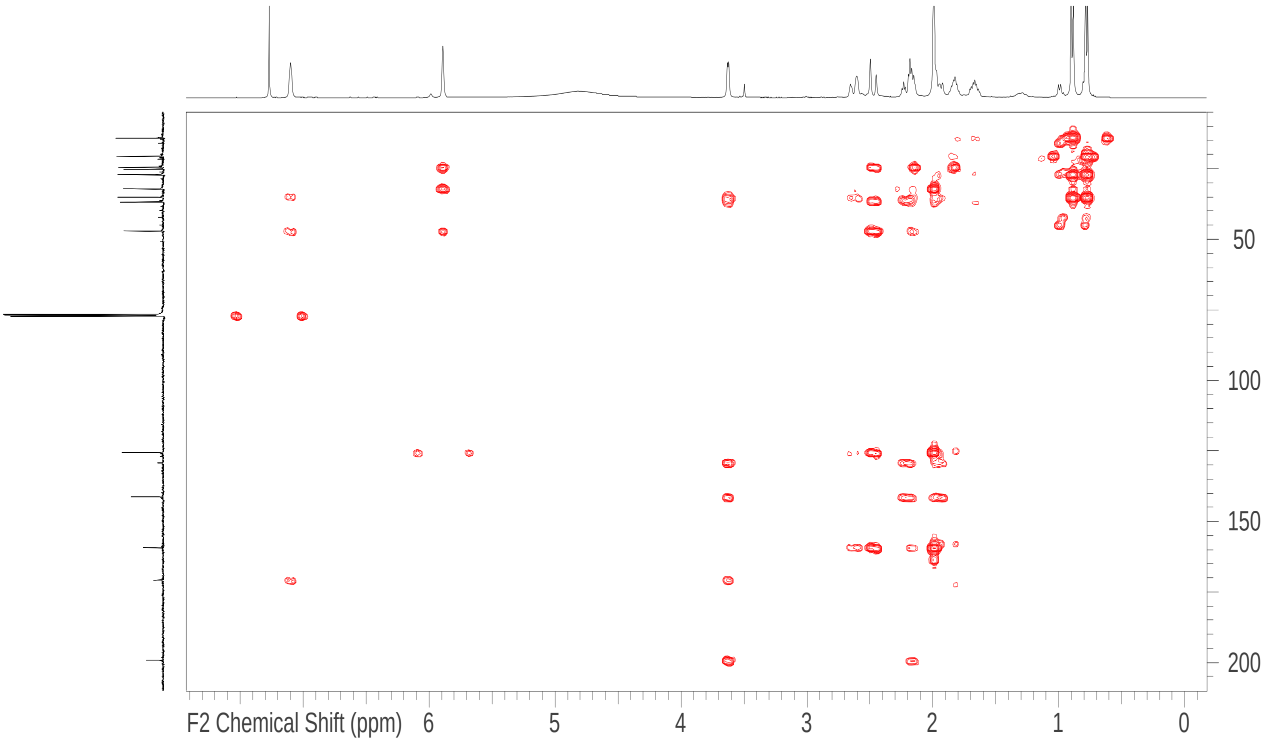

Figure S93 – HMBC NMR spectrum (400 MHz, $CDCl_3$) of **9**

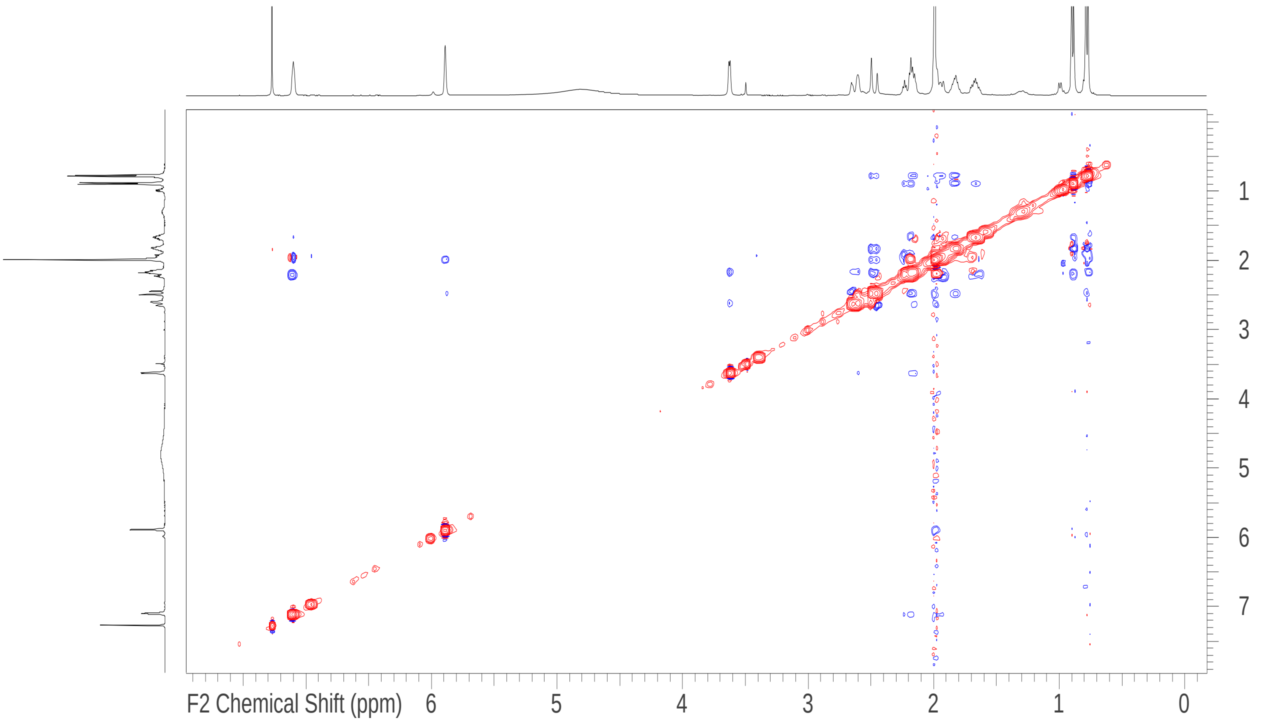

Figure S94 – NOESY NMR spectrum (400 MHz, $CDCl_3$) of **9**

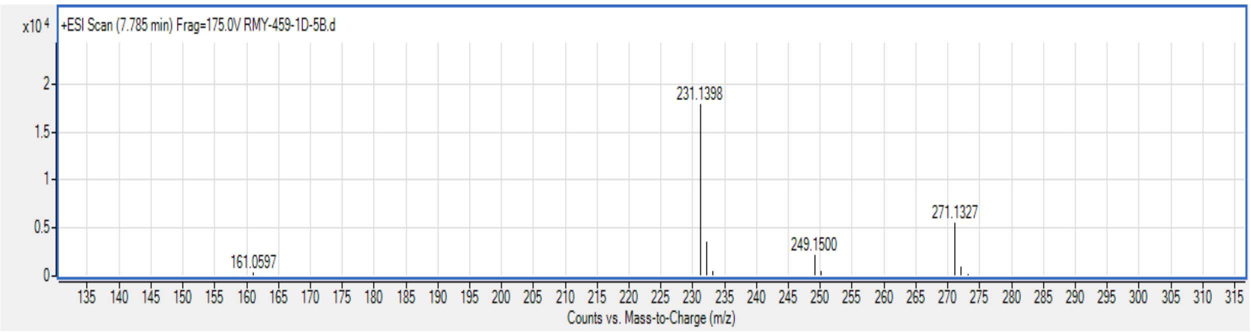

Figure S95 – HRESIMS analysis of **9**

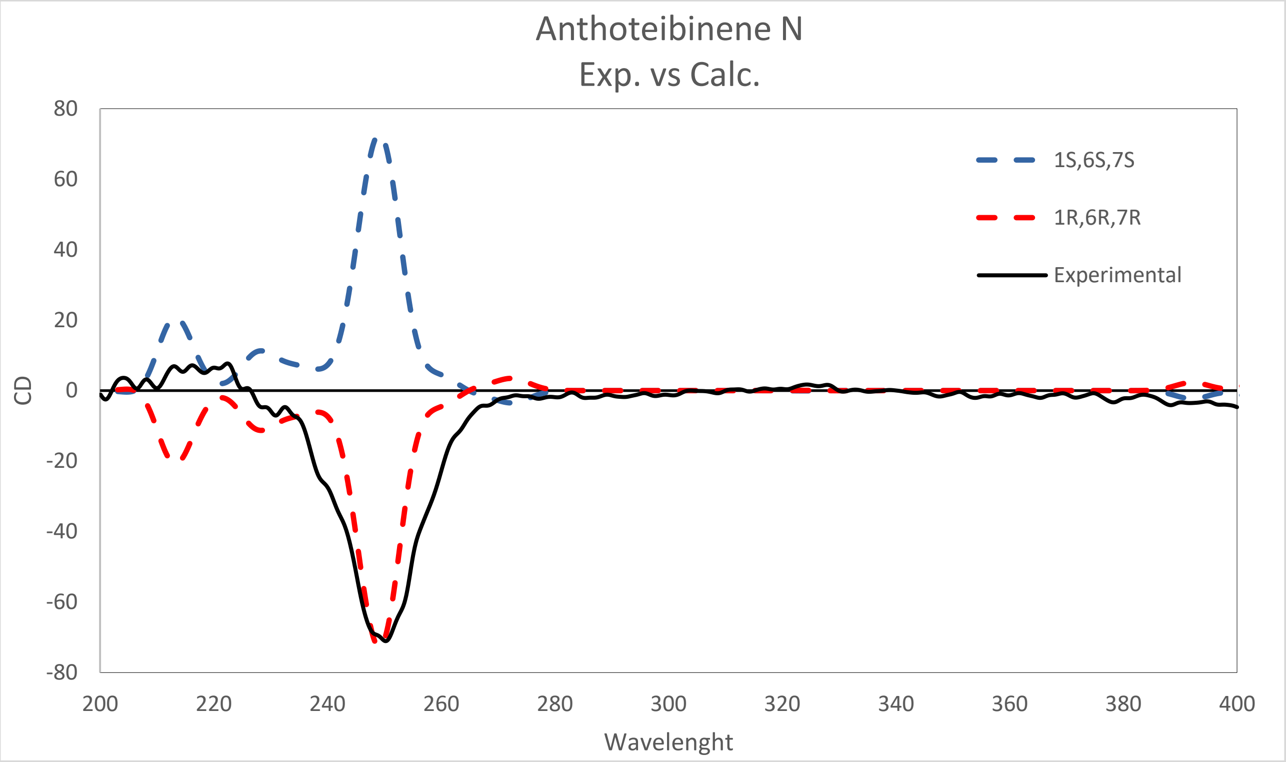

Figure S96 – ECD analysis of **9**

Table S11 – NMR Data for Anthoteibinene O (**10**) (400 (^{1}H) and 100 (^{13}C) MHz, $CDCl_3$)

pos	δ_C, type	δ_H (*J* in Hz)	gCOSY	gHMBC	Key NOESY
**1**	118.6, C				
**2**	153.8, C				
**3**	113.9, CH	6.41, s	5, 15	1, 2, 5, 15	
**4**	136.9, C				
**5**	121.8, CH	6.63, s	3, 7, 15	1, 2, 3, 6, 7, 15	
**6**	141.8, C				
**7**	42.8, CH	2.55, ddd (2.0, 5.1, 10.5)	5, 8a, 8b, 11	1, 5, 6, 9, 11, 12, 13	12
**8a**	20.8, CH_2	1.87, br m	7, 8b	11	
**8b**		1.65, br m	7, 8a	6, 9, 10	13
**9a**	24.1, CH_2	1.93, o/l*	9b, 10	7, 8	
**9b**		2.09, o/l	9a, 10		
**10**	40.2, CH	3.74, br t (5.8)	9a, 9b	1, 6, 8, 9, 14	
**11**	31.6, CH	2.13, o/l	7, 12, 13	6, 7, 12, 13	
**12**	21.8, CH_3	1.00, d (6.8)	11	7, 11, 13	7
**13**	18.5, CH_3	0.76, d (6.8)	11	7, 11, 12	8b
**14**	181.7, C				
**15**	21.3, CH_3	2.19, s	3, 5	3, 4, 5	

*Overlapping ^{1}H NMR signals, 2D assignments based on proximity likelihood

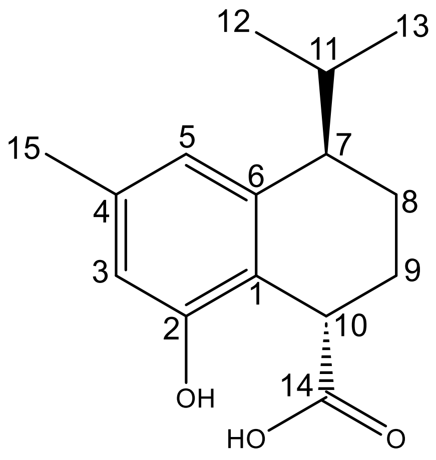

Figure S97 – Structure of **10**

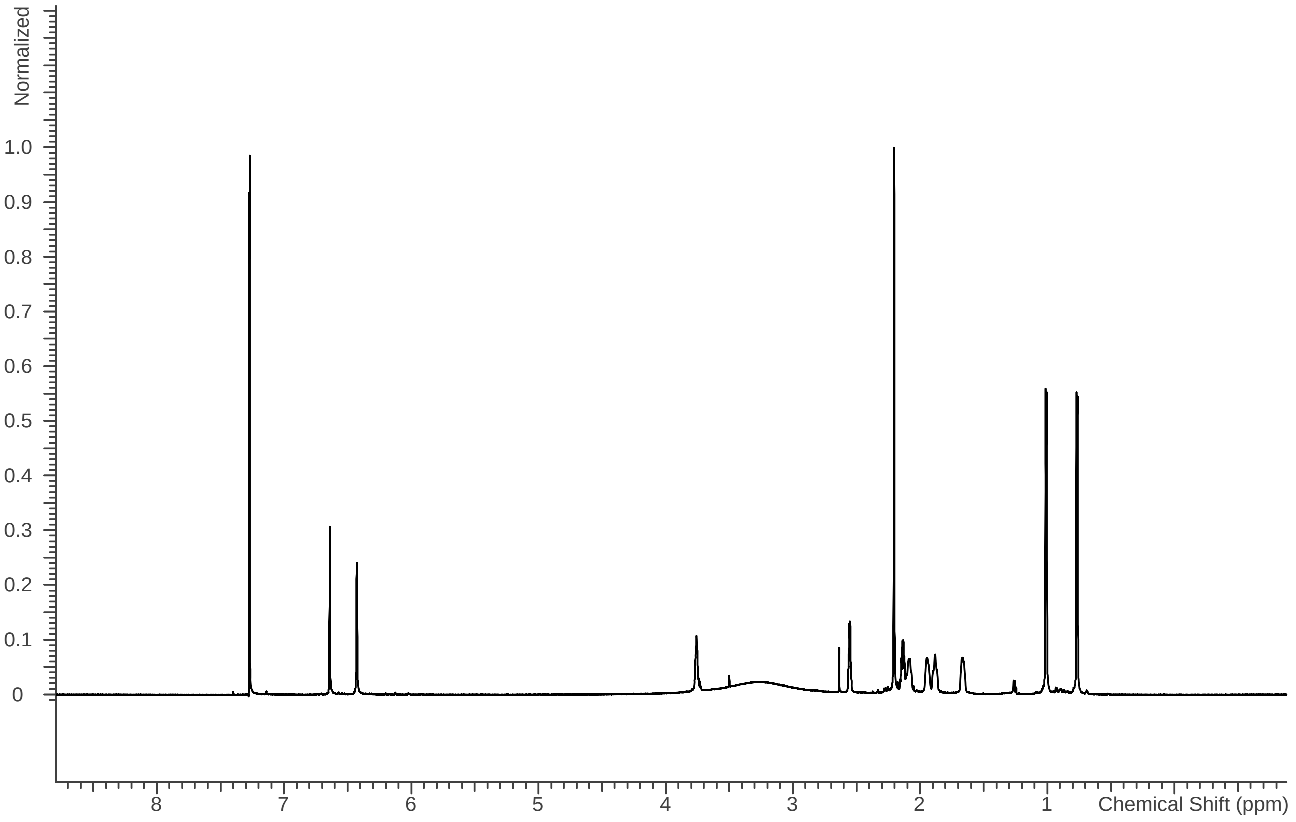

Figure S98 – ^{1}H NMR spectrum (400 MHz, $CDCl_3$) of **10**

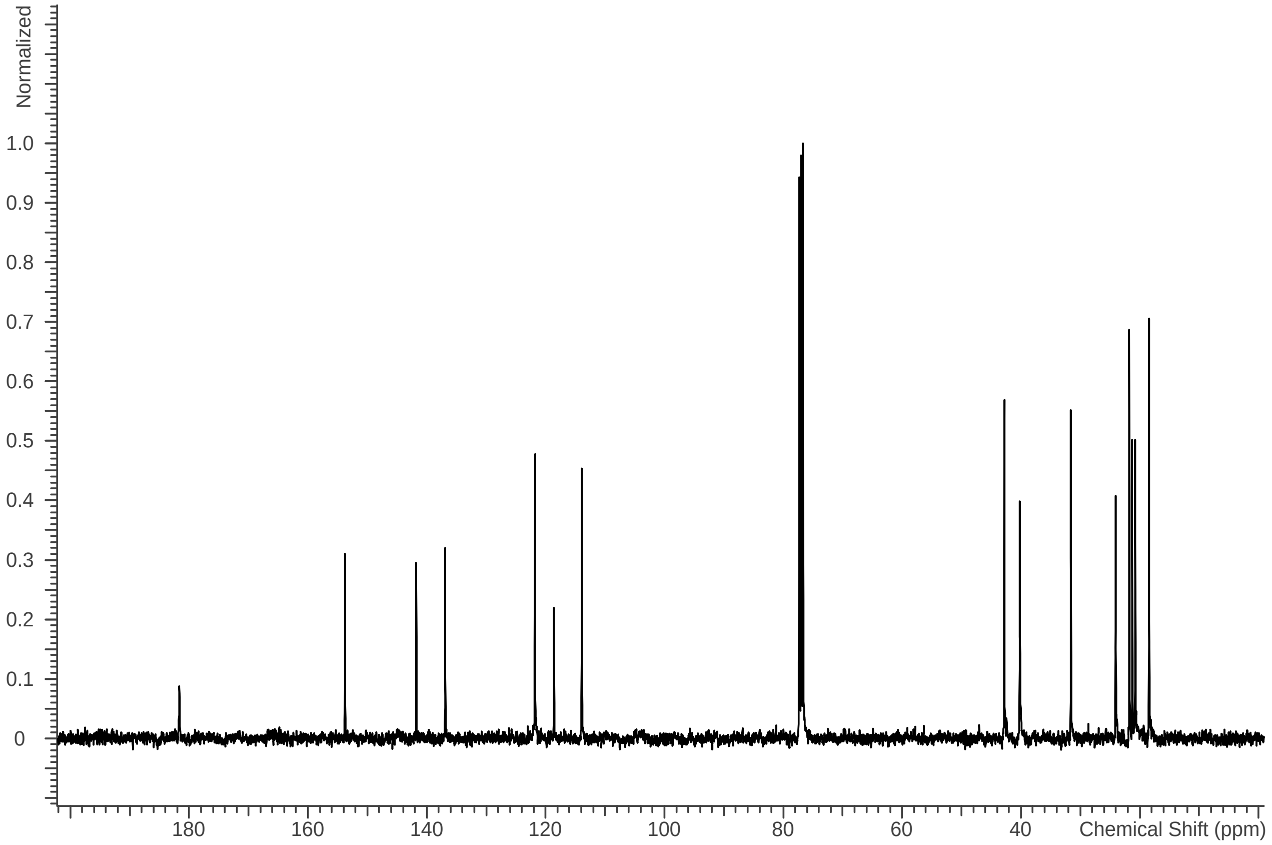

Figure S99 – ^{13}C NMR spectrum (100 MHz, $CDCl_3$) of **10**

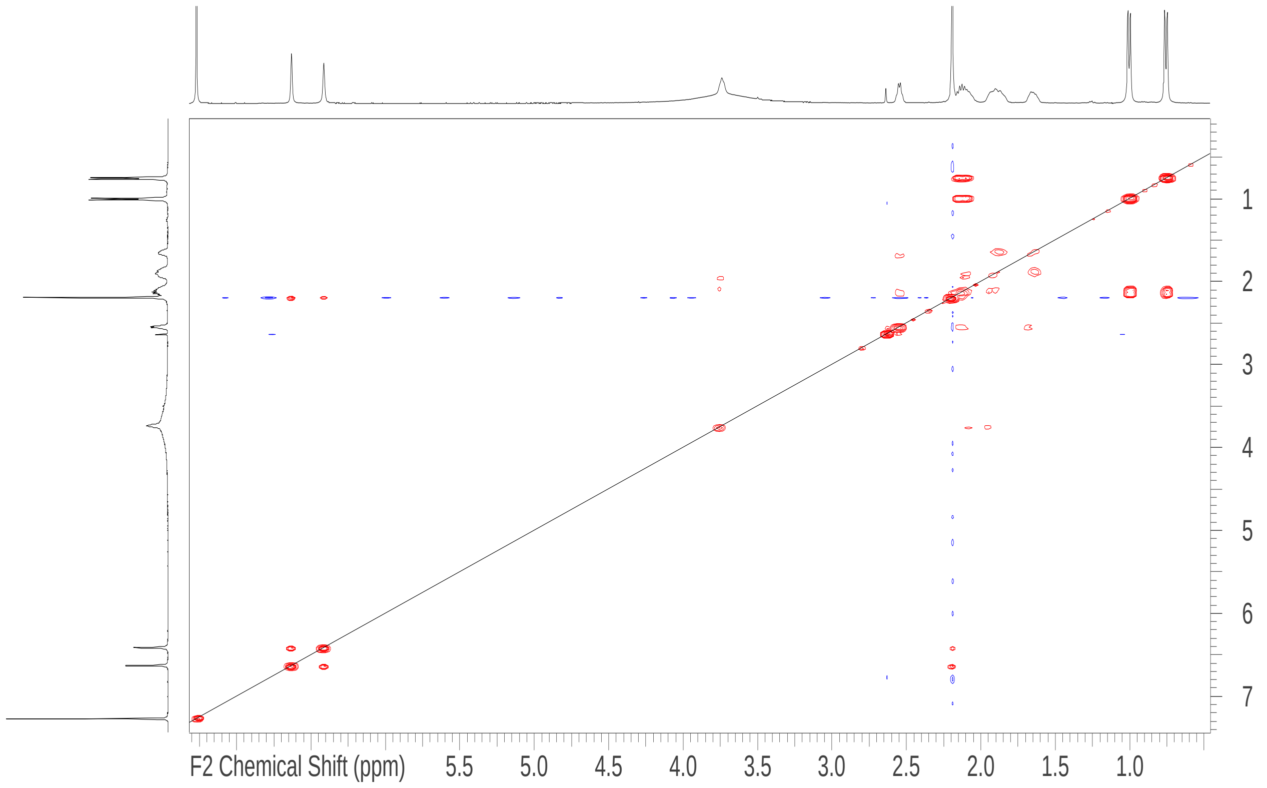

Figure S100 – COSY NMR spectrum (400 MHz, $CDCl_3$) of **10**

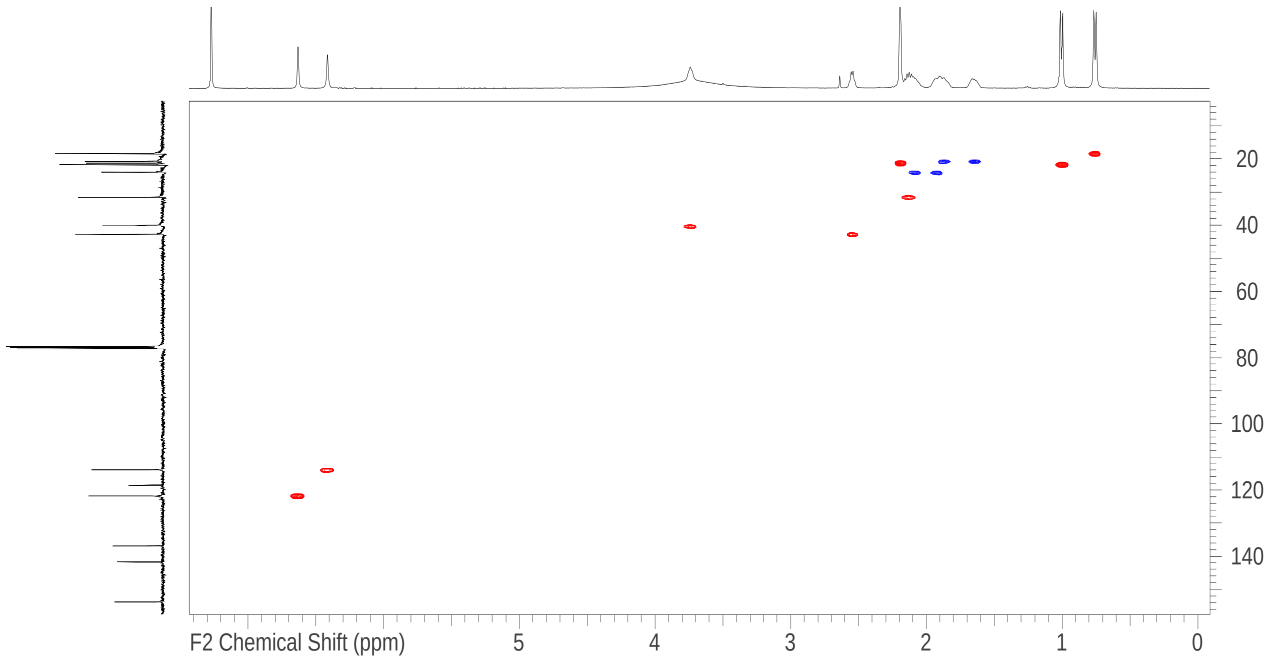

Figure S101 – HSQC NMR spectrum (400 MHz, $CDCl_3$) of **10**

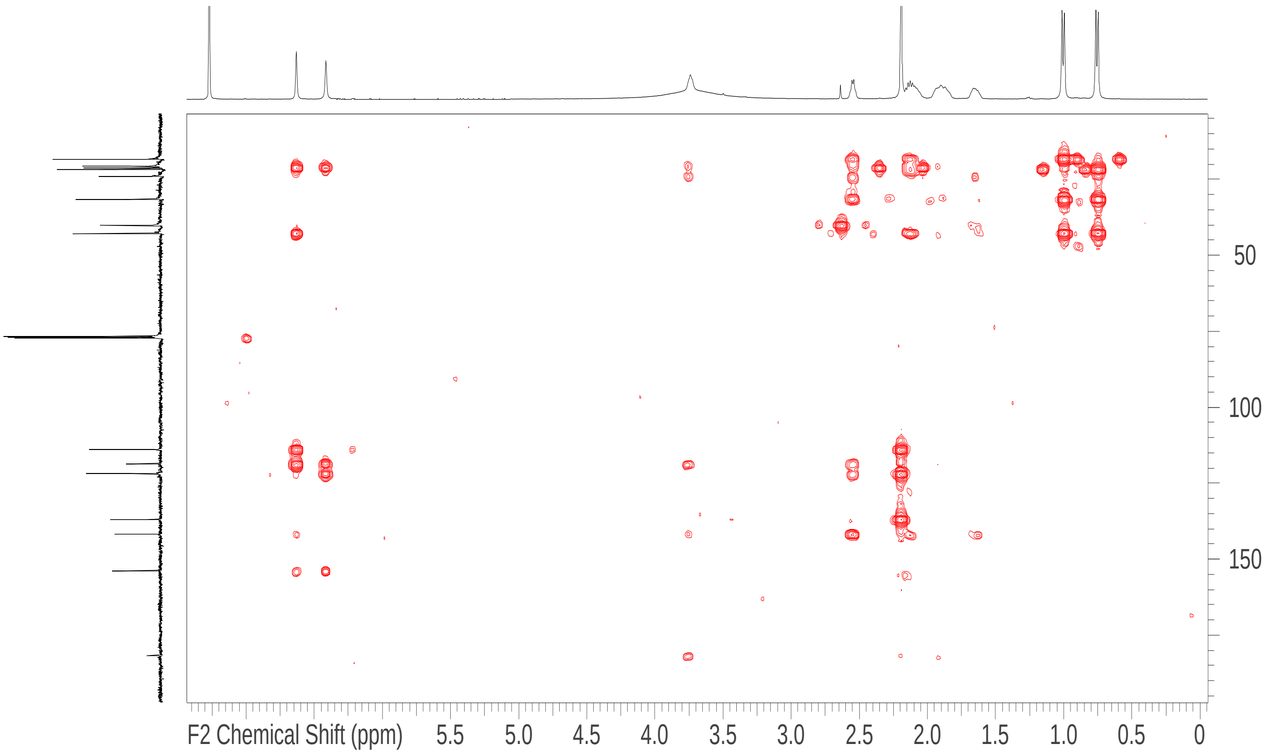

Figure S102 – HMBC NMR spectrum (400 MHz, $CDCl_3$) of **10**

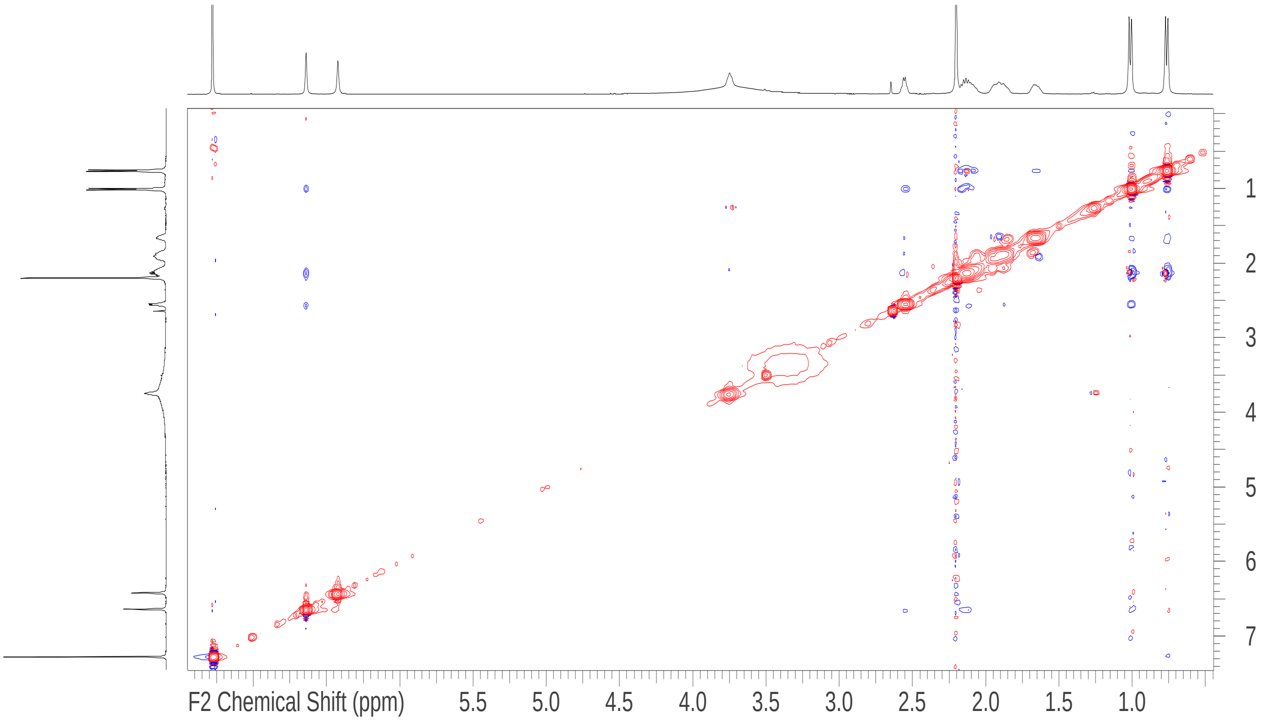

Figure S103 – NOESY NMR spectrum (400 MHz, $CDCl_3$) of **10**

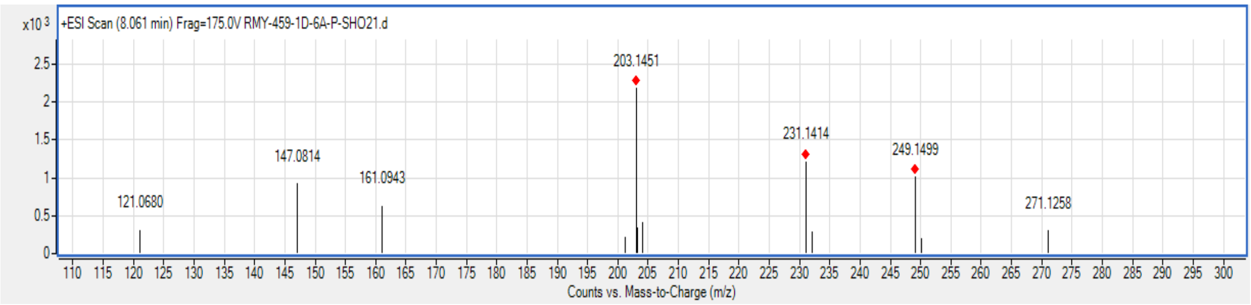

Figure S104 – HRESIMS analysis of **10**

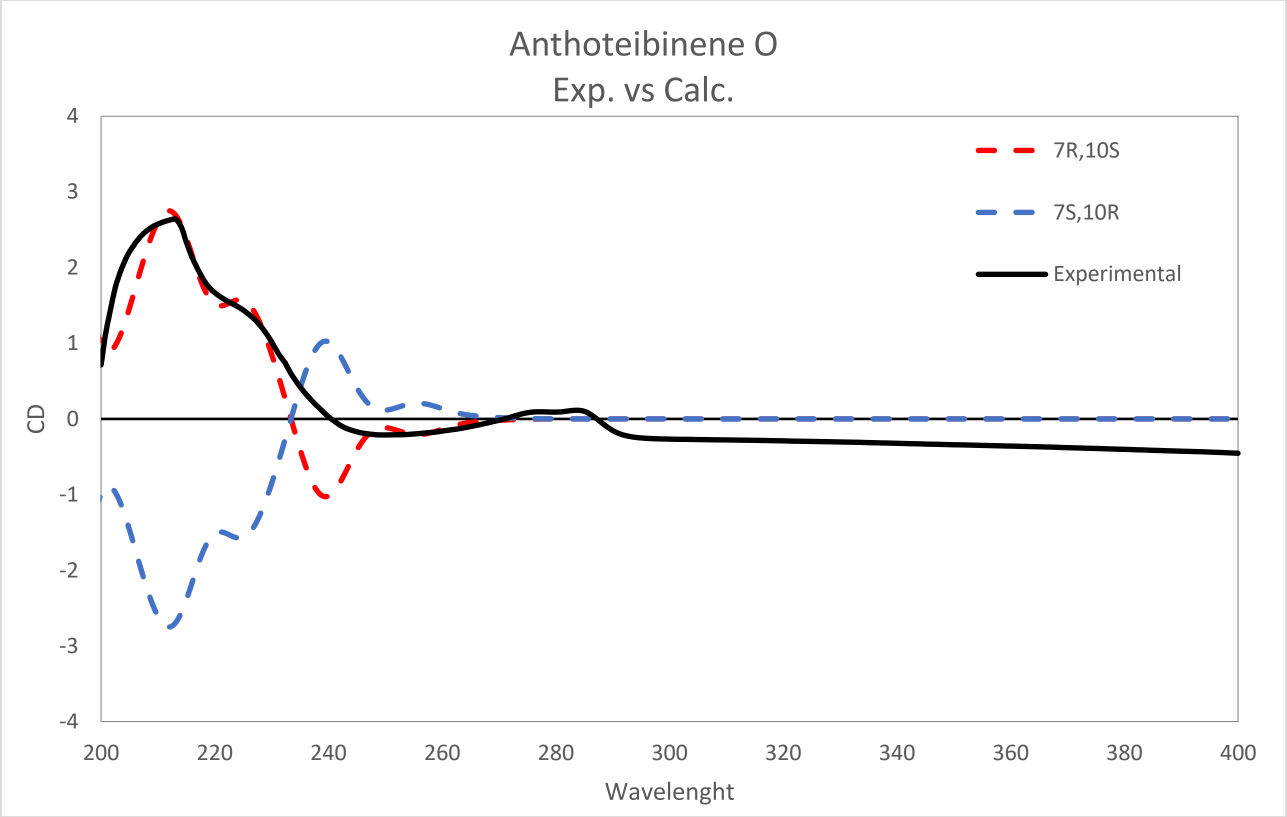

Figure S105 – ECD analysis of **10**

Table S12 – NMR Data for Anthoteibinene P (**11**) (400 (^{1}H) and 100 (^{13}C) MHz, $^{a}CDCl_3$, $^{b}CD_3COCD$)

pos	$^{a}\delta_C$, type	$^{a}\delta_H$	gCOSY	gHMBC	Key NOESY	$^{b}\delta_C$ Tatarinowin A	$^{b}\delta_H$ Tatarinowin A
**1**	125.7, C					127.7, C	
**2**	190.6, C					190.6, C	
**3**	129.4, CH	5.94, s	5, 15	1, 5, 15		129.3, CH	5.78, s
**4**	158.5, C					158.3, C	
**5**	68.6, CH	4.01, br d (2.6)	3, 6	1, 3, 4, 15	7, 11, 15	68.1, CH	4.07, d (3.3)
**6**	44.2, CH	2.59, br dd (2.6, 9.8)	5, 7, 9		11, 8b, 13	44.8, CH	2.50, m
**7**	39.4, CH	1.80, dddd (2.5, 2.8, 9.8, 12.5)	6, 8a, 8b	1, 11, 13	12	40.1, CH	1.87, m
**8a**	19.9, CH_2	1.72, dddd (2.5, 5.4, 11.0, 12.5)	7, 8b, 9	10	5, 12	20.7, CH_2	1.72, m
**8b**		1.22, dddd (5.5, 11.0, 12.5, 12.5)	7, 8a, 9	6, 7, 9	6, 13		1.17, m
**9**	34.2, CH_2	2.22, m	8a, 8b	7, 10		34.8, CH_2	2.15, m
**10**	153.1, C					150.1, C	
**11**	27.2, CH	1.95, d sept (2.8, 6.9)	7, 12, 13	7, 8	5, 6	27.5, CH	2.02, m
**12**	21.6, CH_3	1.03, d (6.9)	11	7, 11, 13	7, 8a	16.4, CH_3	0.99, d (6.8)
**13**	16.2, CH_3	0.82, d (6.9)	11	7, 11, 12	6, 8b	21.9, CH_3	0.80, d (6.8)
**14**	22.5, CH_3	2.10, s*		2, 1, 9, 10		22.5, CH_3	1.97, s
**15**	22.0, CH_3	2.10, s*	3	2, 3, 4, 5	5	22.0, CH_3	2.03, s

*Overlapping ^{1}H NMR signals, 2D assignments based on proximity likelihood

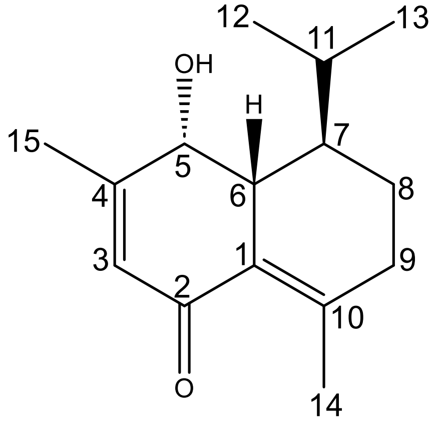

Figure S106 – Structure of **11**

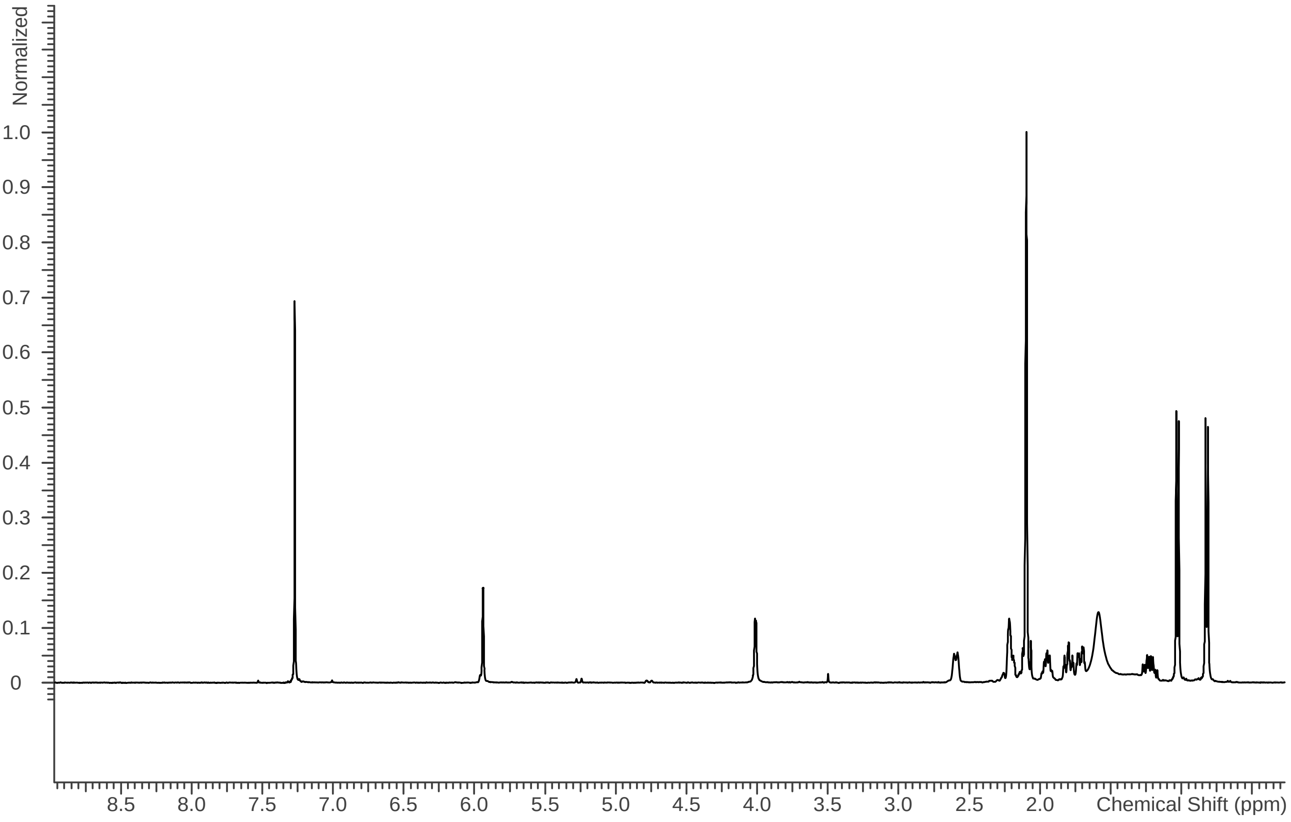

Figure S107 – ^{1}H NMR spectrum (400 MHz, $CDCl_3$) of **11**

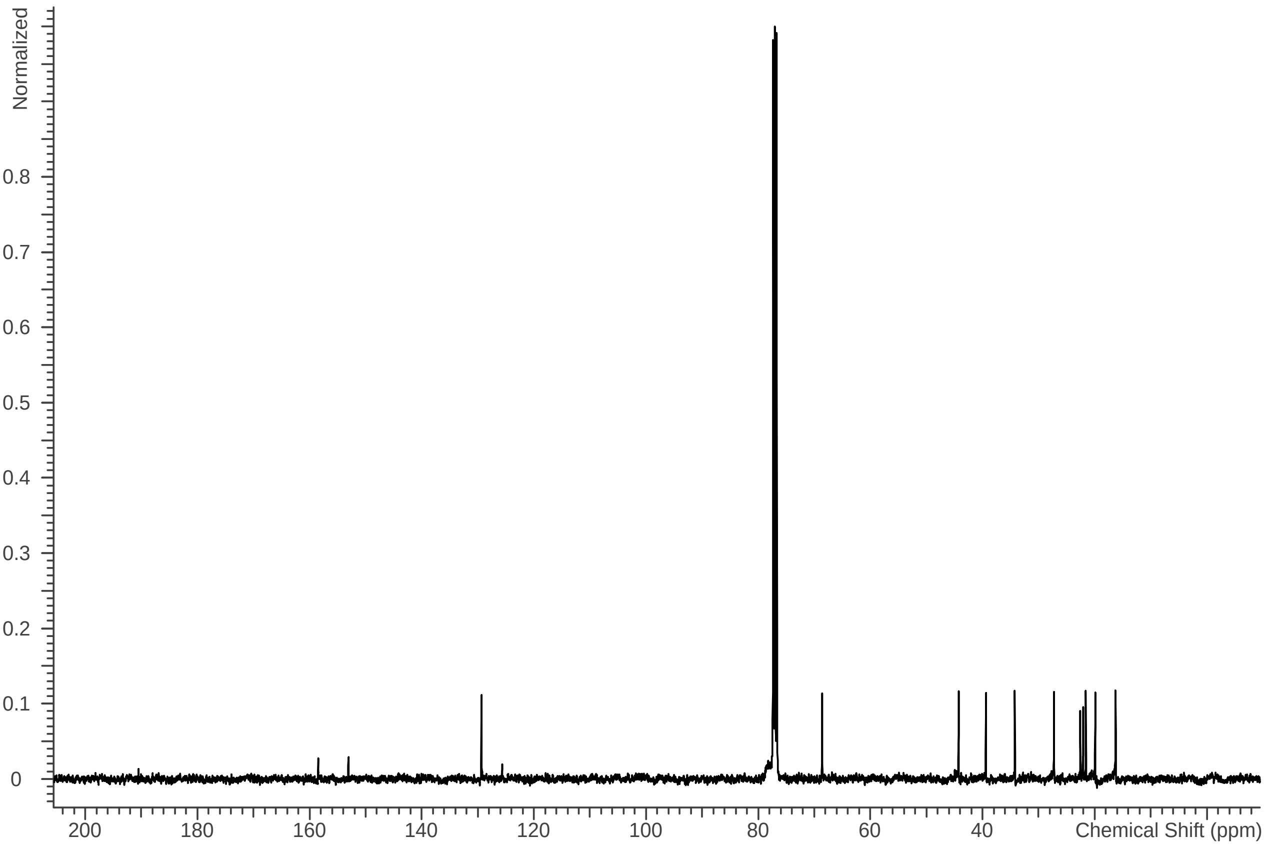

Figure S108 – ^{13}C NMR spectrum (100 MHz, $CDCl_3$) of **11**

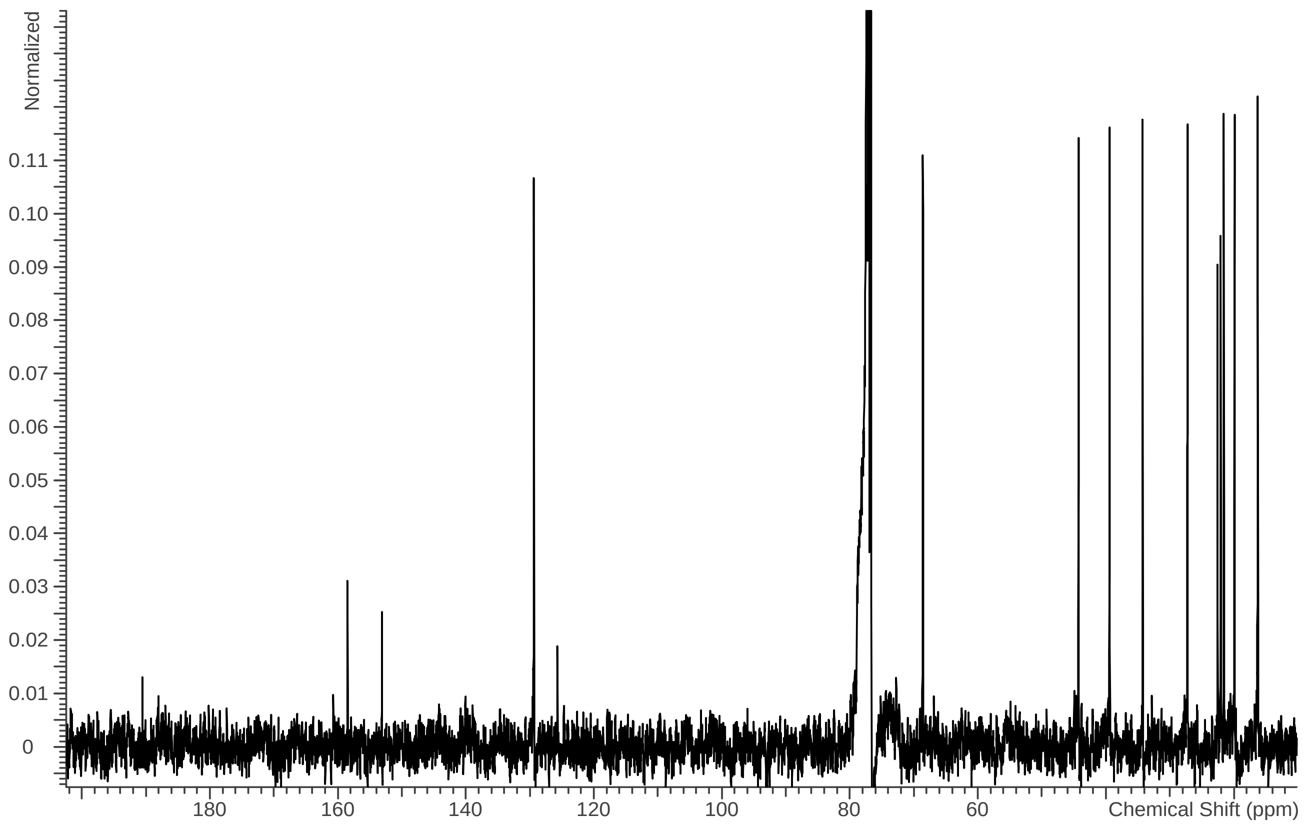

Figure S109 – ^{13}C NMR spectrum zoomed (100 MHz, $CDCl_3$) of **11**

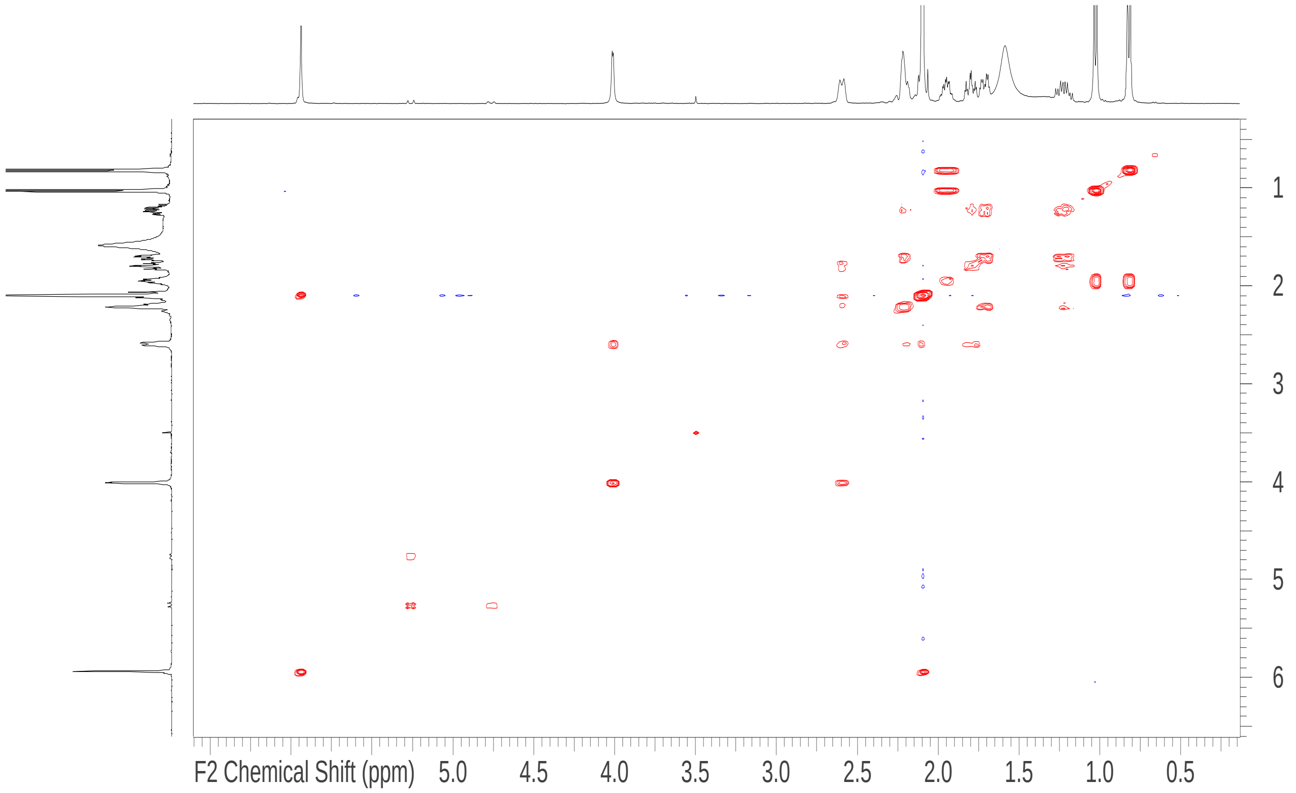

Figure S110 – COSY NMR spectrum (400 MHz, $CDCl_3$) of **11**

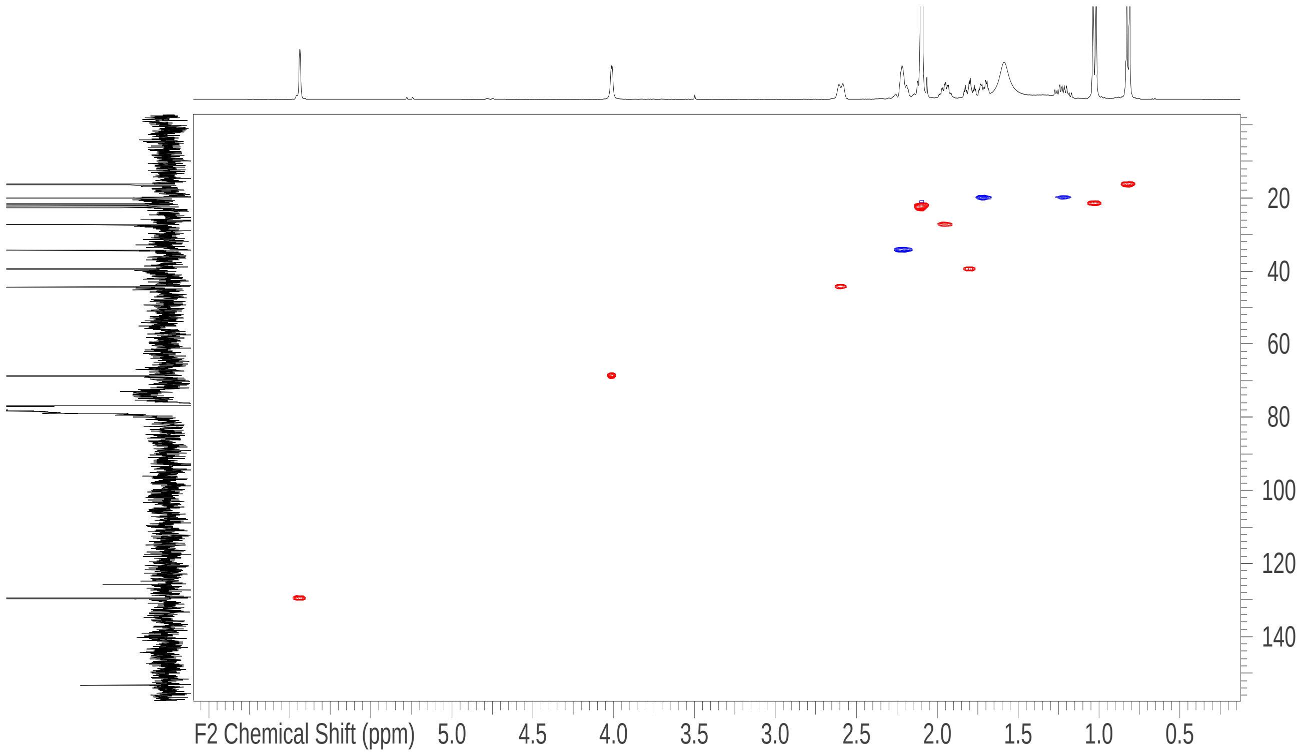

Figure S111 – HSQC NMR spectrum (400 MHz, $CDCl_3$) of **11**

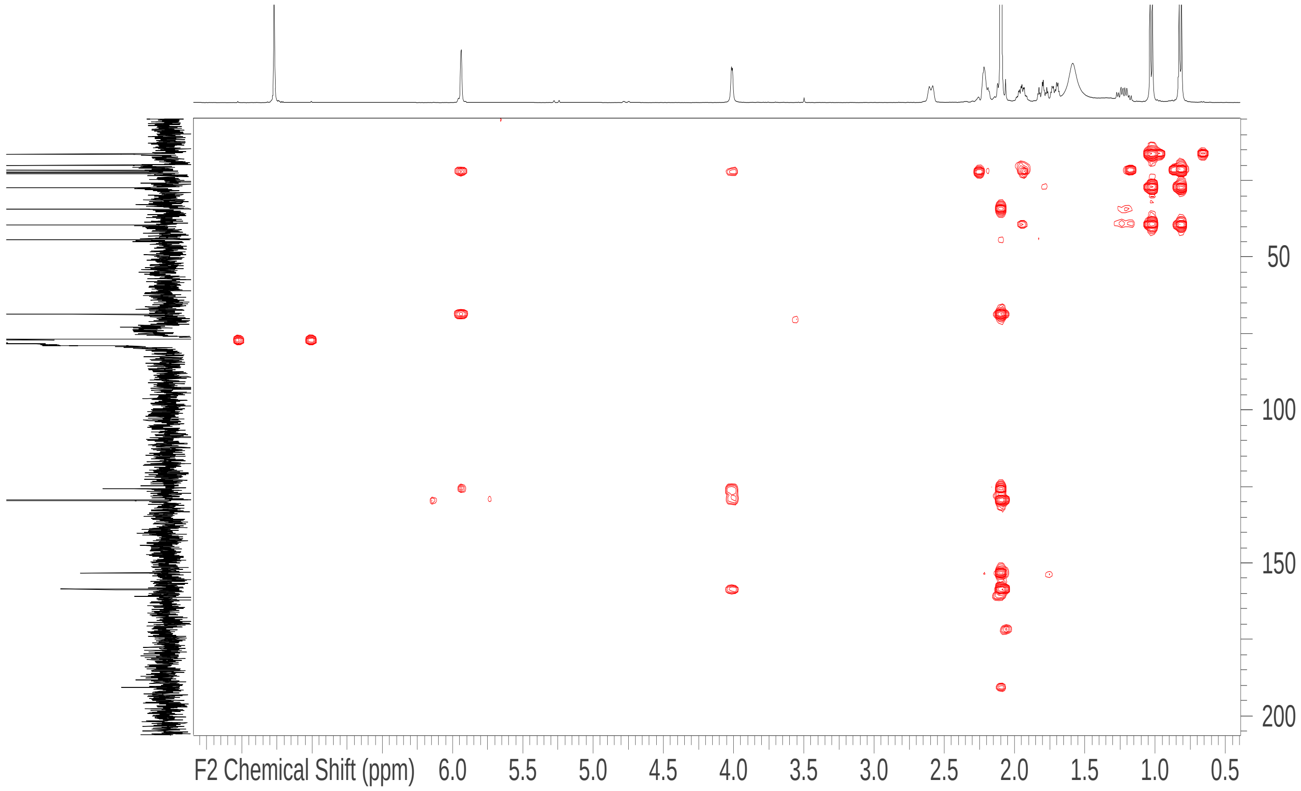

Figure S112 – HMBC NMR spectrum (400 MHz, $CDCl_3$) of **11**

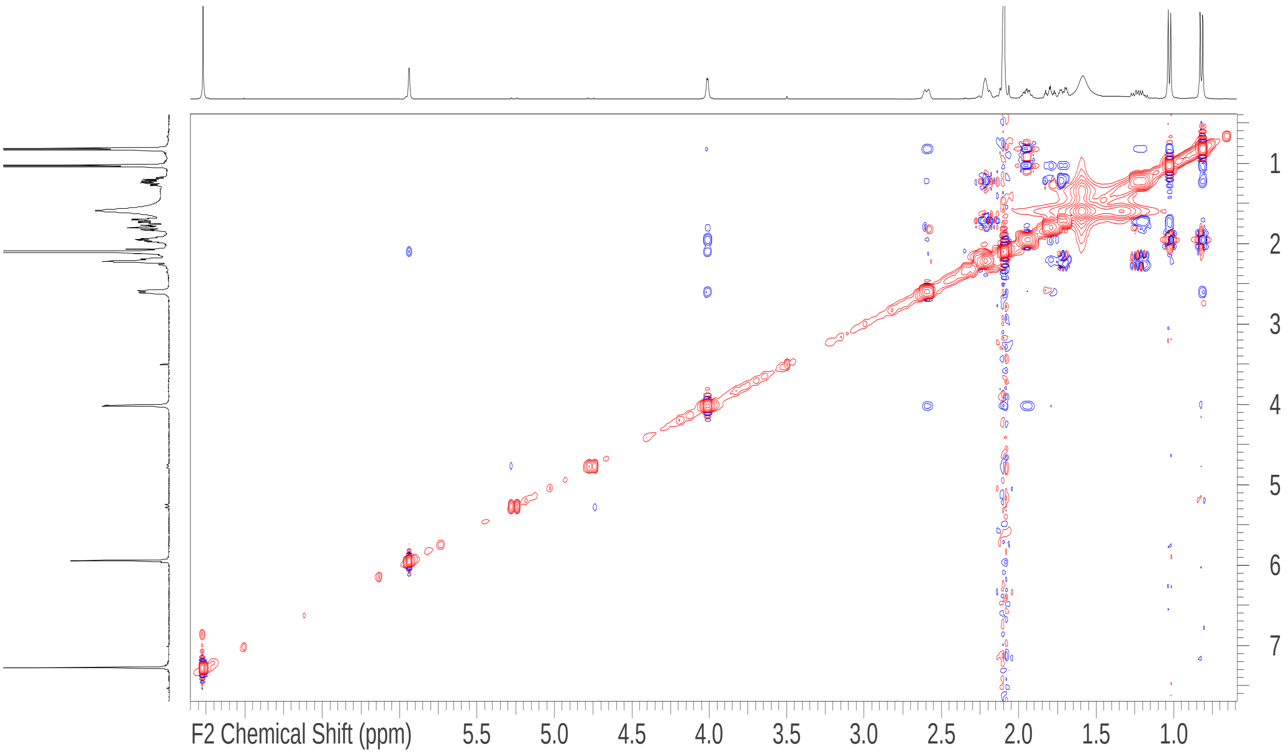

Figure S113 – NOESY NMR spectrum (400 MHz, $CDCl_3$) of **11**

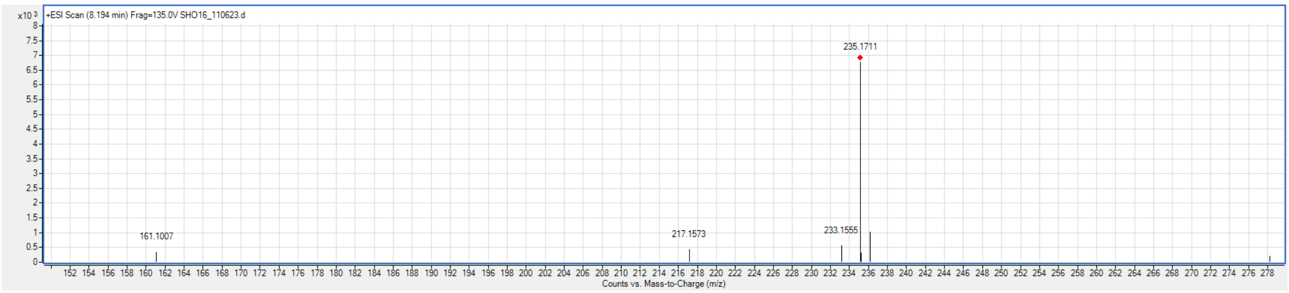

Figure S114 – HRESIMS analysis of **11**

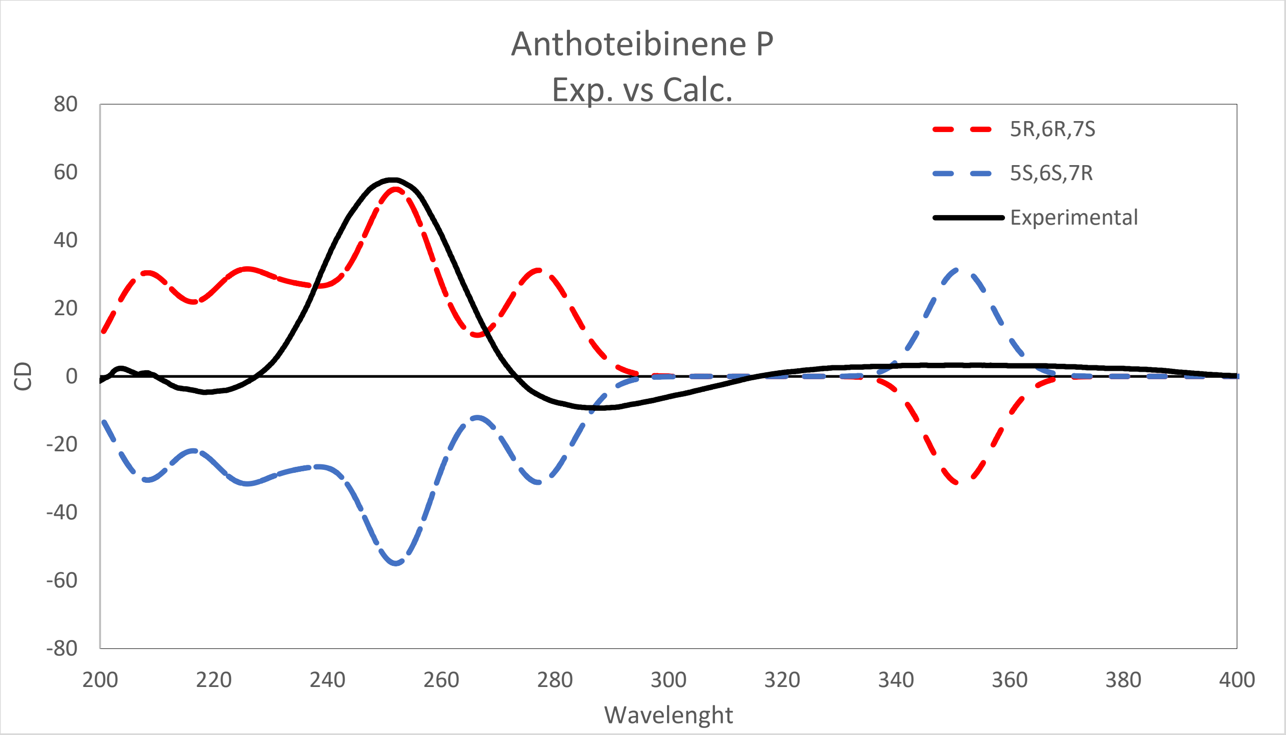

Figure S115 – ECD analysis of **11**

Table S13 – NMR Data for Anthoteibinene Q (**12**) (600 (^{1}H) and 150 (^{13}C) MHz, $CDCl_3$)

pos	δ_C, type	δ_H	gCOSY	gHMBC
**1**	114.6, C			
**2**	157.2, C			
**3**	116.9, CH	6.65, s	14	1, 2, 5, 14
**4**	135.3, C			
**5**	143.0, C			
**6**	132.6, C			
**7**	38.1, CH	2.87, ddd (3.1, 3.1, 9.5)	9b, 11	1, 5, 6, 8, 12/13
**8a**	24.7, CH_2	2.29, o/l*	8b, 9a, 9b	1, 6, 7, 9, 10
**8b**		2.04, dddd (5.3, 5.3, 14.0, 14.0)	8a, 9a, 9b	6, 7, 9, 10
**9a**	33.3, CH_2	2.79, ddd (5.8, 14.0, 19.1)	8a, 8b, 9b	7, 8, 10
**9b**		2.55, ddd (1.6, 5.3, 19.1)	8a, 8b, 9a	1, 7, 8, 10
**10**	204.4, C			
**11**	31.3, CH	1.87, d sept (2.4, 6.7)	7, 12, 13	6, 12/13
**12**	21.3, CH_3	1.10, d (6.5)	11	7, 11, 13
**13**	21.2, CH_3	0.91, d (6.7)	11	7, 11, 12
**14**	17.4, CH_3	2.29, s*	3	3, 4, 5
**OH**		12.21		1, 2, 3, 4, 10

*Overlapping ^{1}H NMR signals, 2D assignments based on proximity likelihood

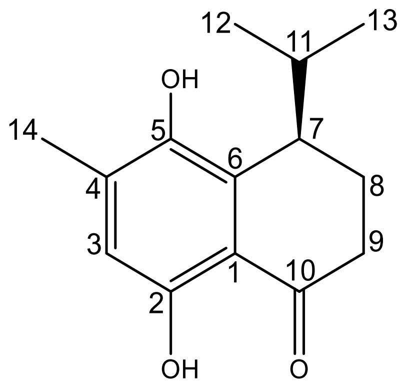

Figure S116 – Structure of **12**

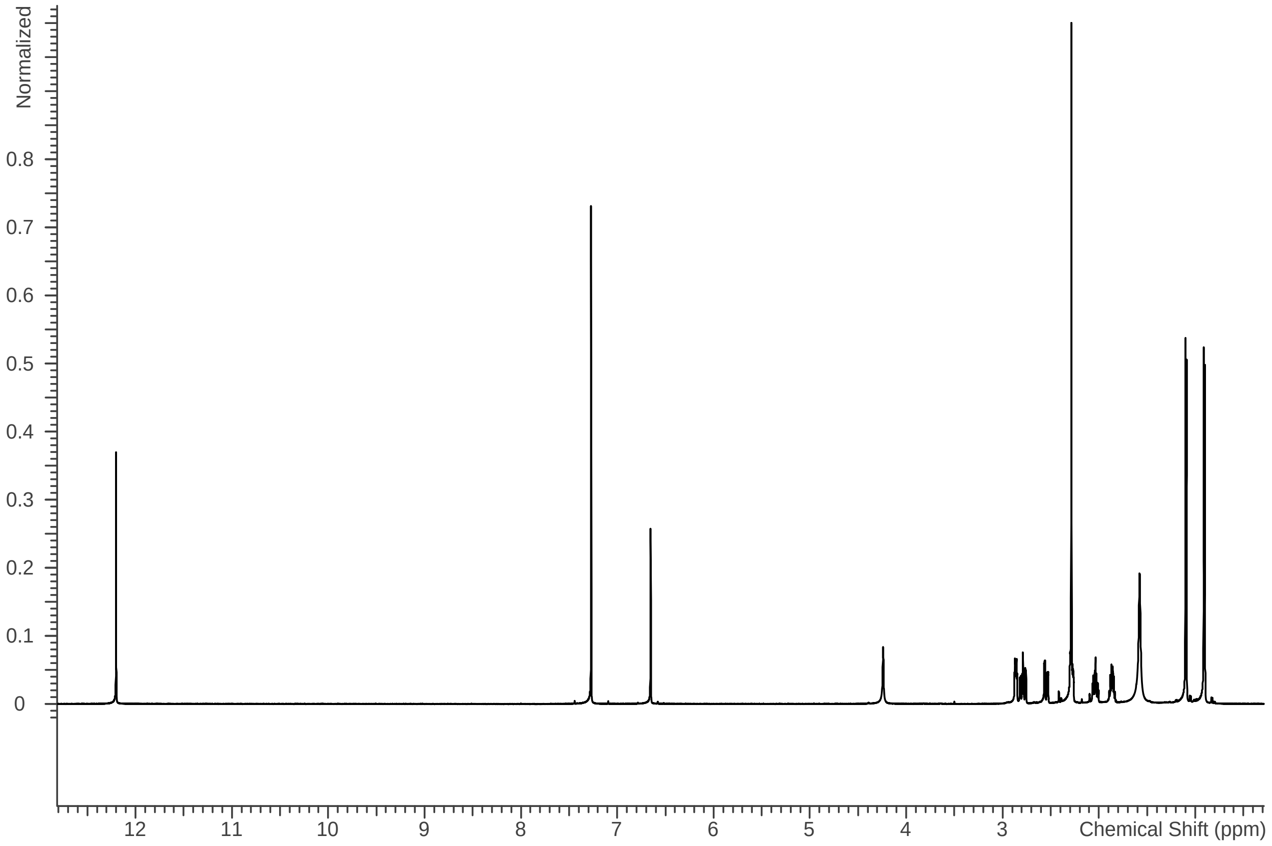

Figure S117 – ^{1}H NMR spectrum (600 MHz, $CDCl_3$) of **12**

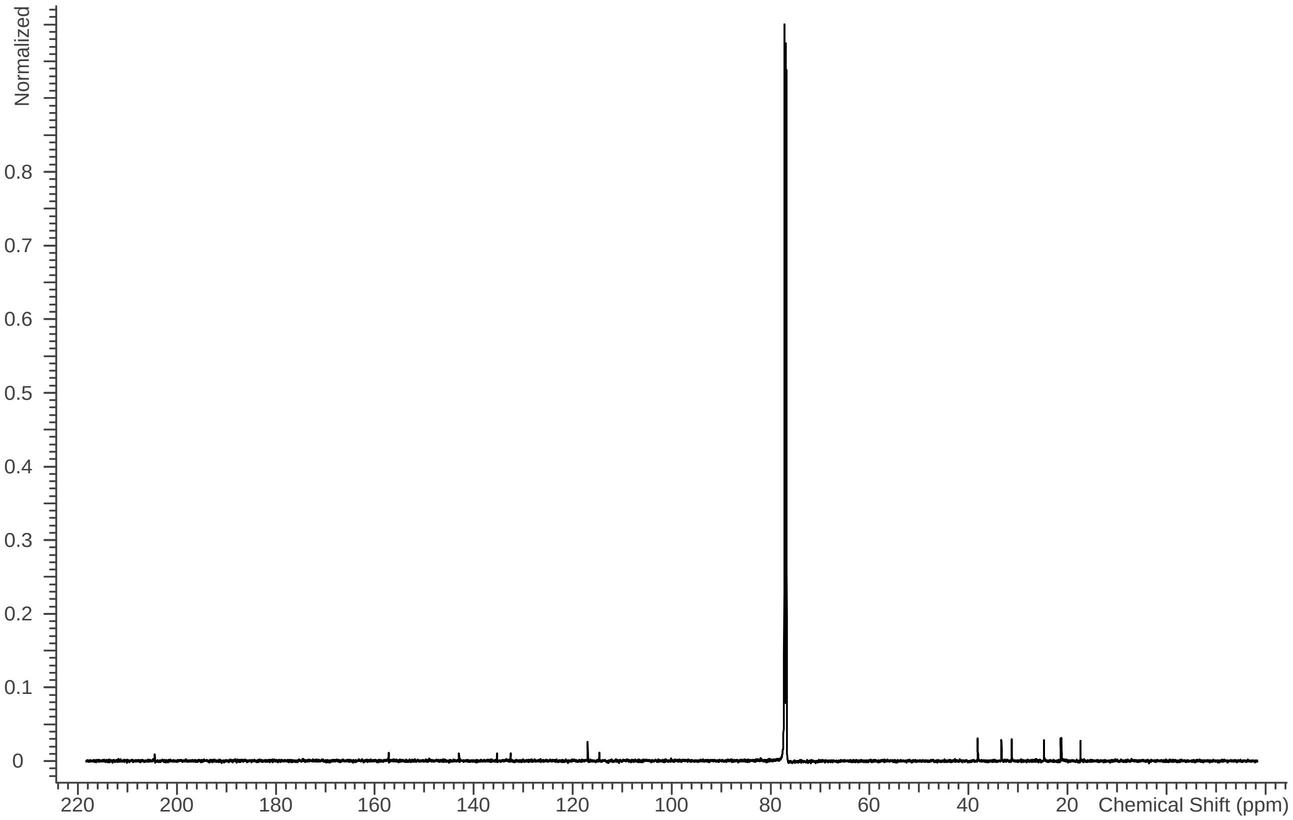

Figure S118 – ^{13}C NMR spectrum (150 MHz, $CDCl_3$) of **12**

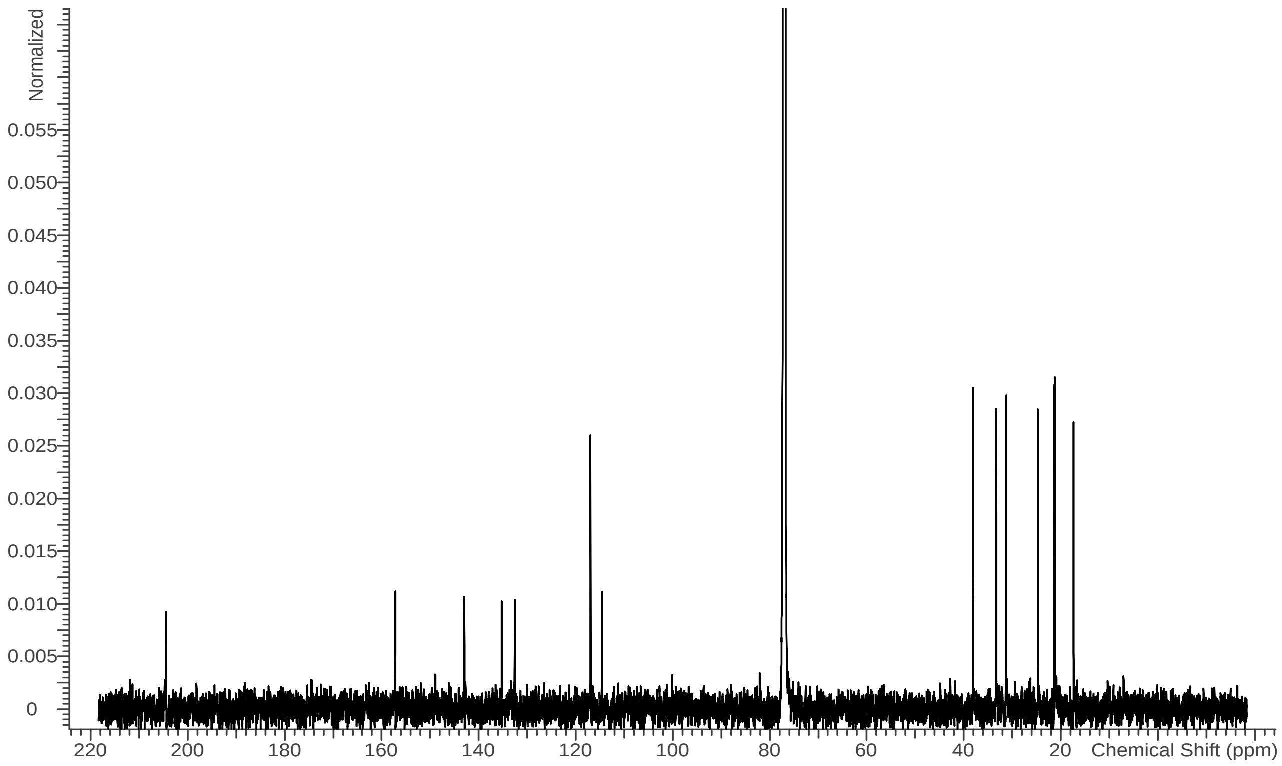

Figure S119 – ^{13}C NMR spectrum zoomed (150 MHz, $CDCl_3$) of **12**

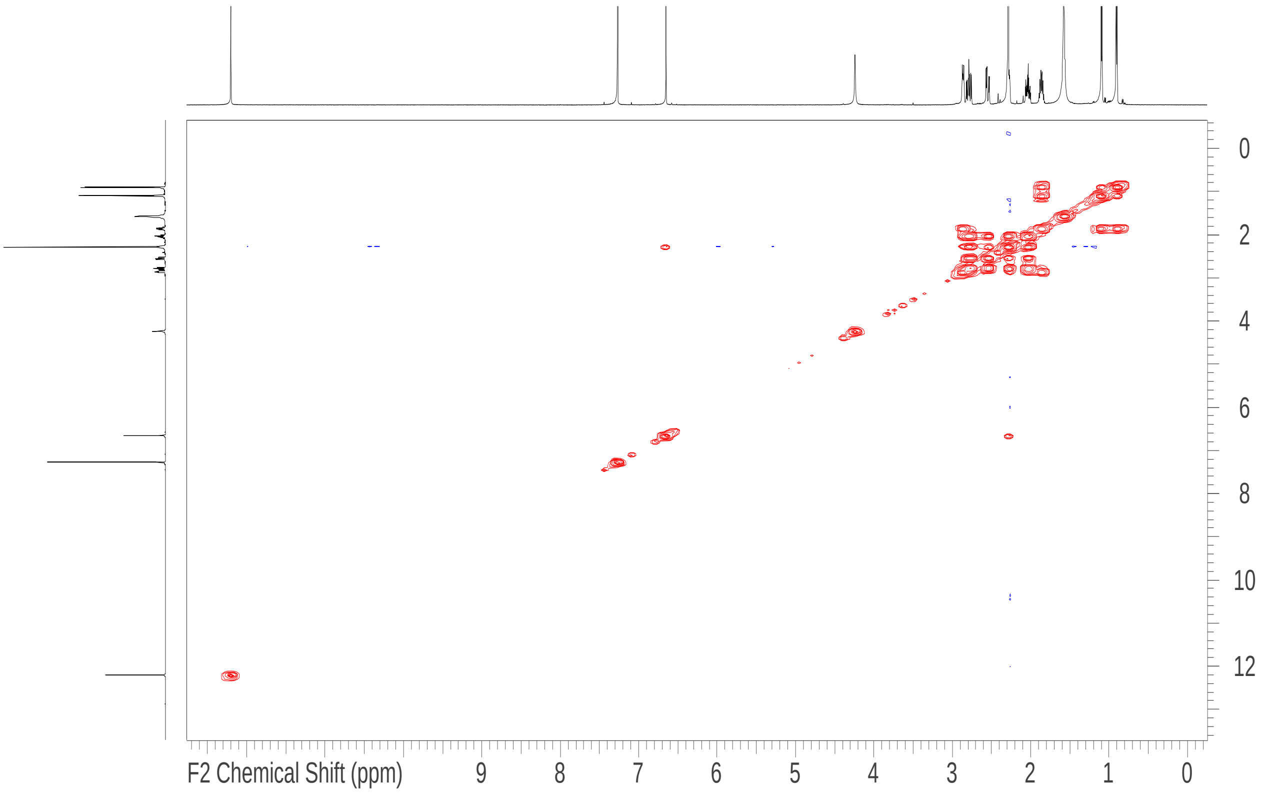

Figure S120 – COSY NMR spectrum (600 MHz, $CDCl_3$) of **12**

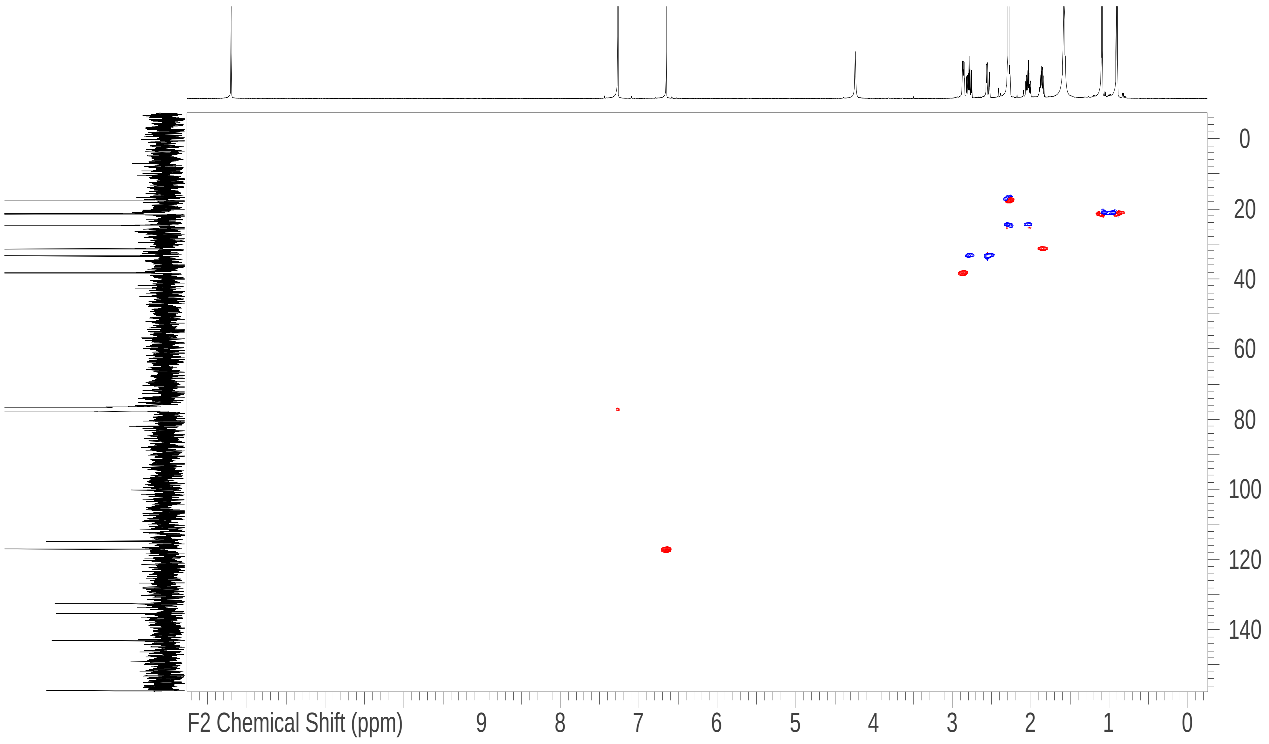

Figure S121 – HSQC NMR spectrum (600 MHz, $CDCl_3$) of **12**

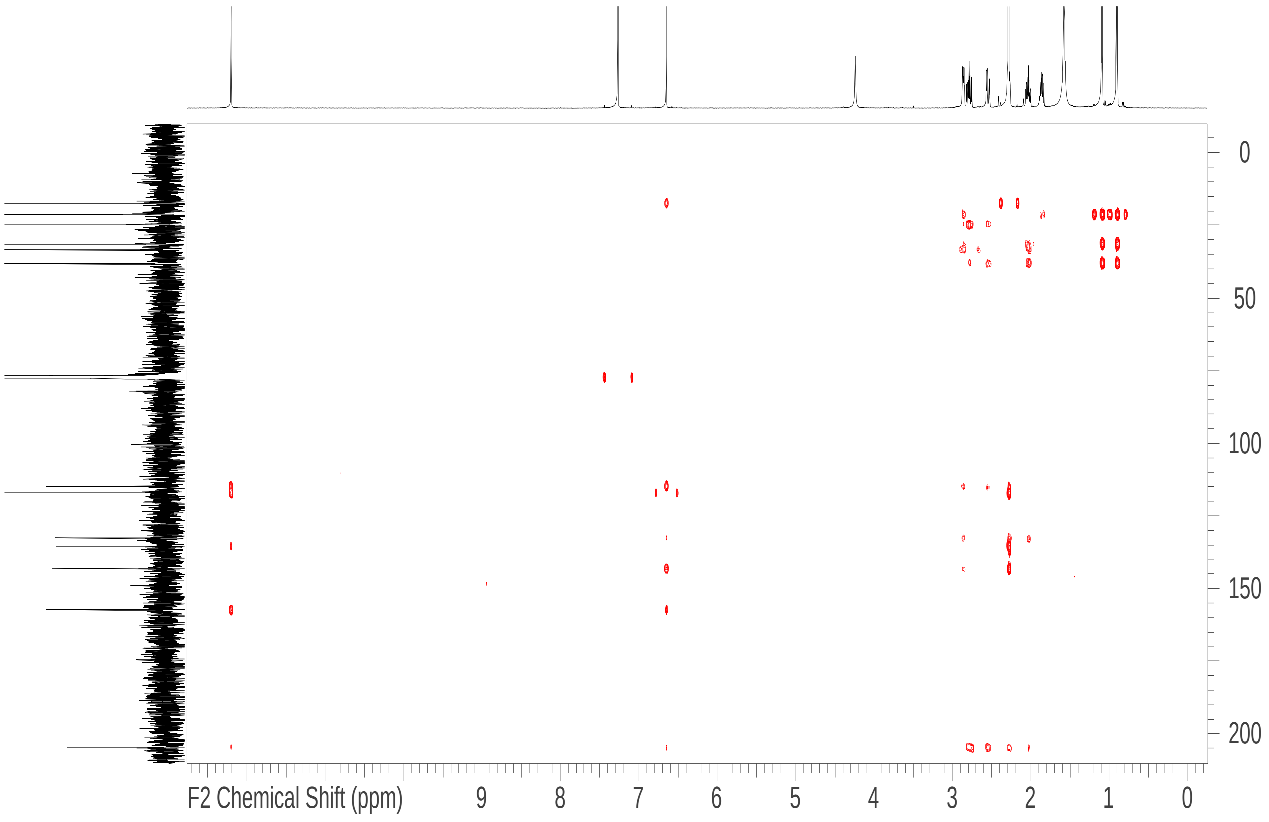

Figure S122 – HMBC NMR spectrum (600 MHz, $CDCl_3$) of **12**

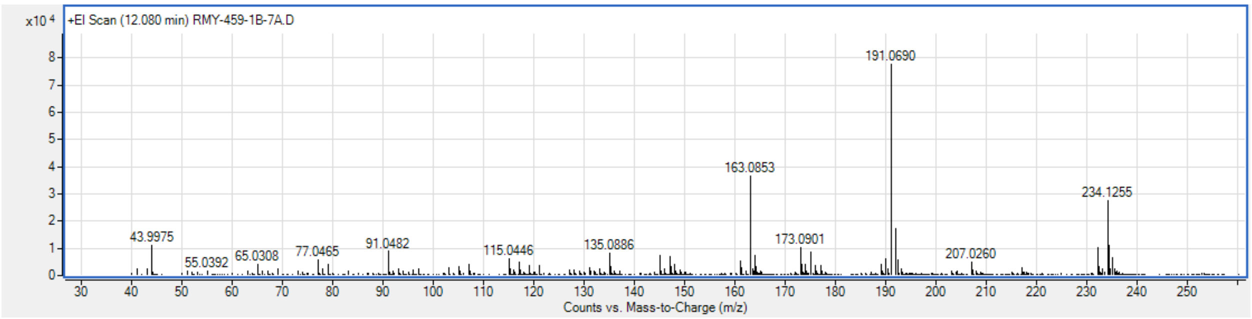

Figure S123 – HREIMS analysis of **12**

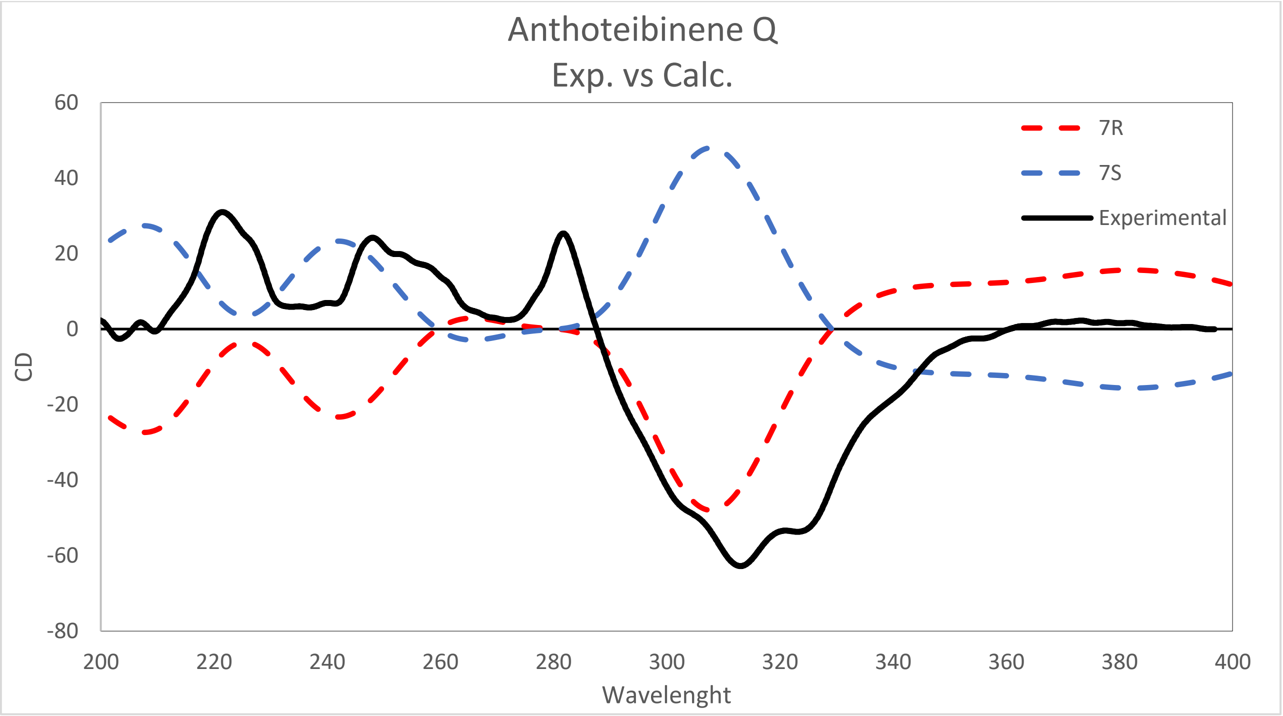

Figure S124 – ECD analysis of **12**

Table S14 – Predicted chemical shifts of possible anthoteibinene F (**1**) diastereomers

**Isomer 1**	**2R-6S-7R**	**Isomer 2**	**2S-6S-7R**	**Experimental**	
**Atom ID**	**Chemical shift** δ_H or δ_C	**Atom ID**	**Chemical shift** δ_H or δ_C	**Atom ID**	**Chemical shift** δ_H or δ_C
C1	105.164	C1	104.497	C-2	102
C2	168.167	C2	174.565	C-1	162
C3	37.872	C3	40.071	C-6	43.4
C4	122.991	C4	120.173	C-5	120.3
C5	138.885	C5	143.217	C-4	131.1
C6	44.524	C6	37.231	C-3	35.5
C7	130.493	C7	125.528	C-7	127.1
C8	21.833	C8	21.57	C-8	20.3
C9	22.422	C9	24.326	C-9	21.1
C10	48.024	C10	46.211	C-10	45.3
C11	29.539	C11	30.356	C-11	26.8
C12	20.053	C12	19.4	C-12	21.6
C13	13.87	C13	13.577	C-13	15.8
C14	22.978	C14	24.52	C-15	23.5
C16	172.142	C16	173.972	C-14	170.2
H19	3.254	H19	3.264	H-6	3
H20	5.893	H20	6.042	H-5	5.58
H22	2.803	H22	2.661	H-3b	2.71
H21	2.492	H21	2.573	H-3a	2.5
H23	2.415	H23	2.307	H-9b	2.42
H24	2.16	H24	2.567	H-9a	2.09
H25	1.414	H25	2.018	H-8b	1.35
H26	1.929	H26	1.739	H-8a	1.91
H27	1.26	H27	1.648	H-7	1.19
H28	2.231	H28	2.281	H-11	2.12
H29	1.188	H29	1.188	H-12	1.03
H30	1.188	H30	1.188	H-12	1.03
H31	1.188	H31	1.188	H-12	1.03
H32	1.049	H32	1.049	H-13	0.89
H33	1.049	H33	1.049	H-13	0.89
H34	1.049	H34	1.049	H-13	0.89
H35	1.932	H35	2.054	H-15	1.77
H36	1.932	H36	2.054	H-15	1.77
H37	1.932	H37	2.054	H-15	1.77

Table S15 – DP4+ probabilities of anthoteibinene F (**1**) diastereomers

Settings	Type of data (shifts)
Default	Unscaled shifts

Functional	Solvent?	Basis Set
B3LYP	PCM	6-311G(d,p)

TMS 1H	31.913	TMS 13C	184.350
Default	μ	σ	ν
13Cu,sp2	7.809	2.409	8.781
13Cu,sp3	5.678	2.271	17.407
1Hu,sp2	0.206	0.124	5.987
1Hu,sp3	-0.022	0.112	2.756
13Cs	-	1.738	6.366
1Hs	-	0.095	2.850

Isomer Nº		1	2	3	4	5	6	7	8
DP4+ (%)	H data	100.00%	0.00%	-	-	-	-	-	-
	C data	100.00%	0.00%	-	-	-	-	-	-
	All data	100.00%	0.00%	-	-	-	-	-	-

Type	sp2?	Exp	1	2	3	4	5	6	7	8
c	x	102	105.164	104.497						
c	x	162	168.167	174.565						
c		43.4	37.872	40.071						
c	x	120.3	122.991	120.173						
c	x	131.1	138.885	143.217						
c		35.5	44.524	37.231						
c	x	127.1	130.493	125.528						
c		20.3	21.833	21.57						
c		21.1	22.422	24.326						
c		45.3	48.024	46.211						
c		26.8	29.539	30.356						
c		21.6	20.053	19.4						
c		15.8	13.87	13.577						
c		23.5	22.978	24.52						
c	x	170.2	172.142	173.972						
H		3	3.254	3.264						
H	x	5.58	5.893	6.042						
H		2.71	2.803	2.661						
H		2.5	2.492	2.573						
H		2.42	2.415	2.307						
H		2.09	2.16	2.567						
H		1.35	1.414	2.018						
H		1.91	1.929	1.739						
H		1.19	1.26	1.648						
H		2.12	2.231	2.281						
H		1.03	1.188	1.188						
H		1.03	1.188	1.188						
H		1.03	1.188	1.188						
H		0.89	1.049	1.049						
H		0.89	1.049	1.049						
H		0.89	1.049	1.049						
H		1.77	1.932	2.054						
H		1.77	1.932	2.054						
H		1.77	1.932	2.054						

Default parameters	1	2
sDP4+ (H data)	100.00%	0.00%
sDP4+ (C data)	99.97%	0.03%
sDP4+ (all data)	100.00%	0.00%
uDP4+ (H data)	100.00%	0.00%
uDP4+ (C data)	99.70%	0.30%
uDP4+ (all data)	100.00%	0.00%
DP4+ (H data)	100.00%	0.00%
DP4+ (C data)	100.00%	0.00%
DP4+ (all data)	100.00%	0.00%

Table S16 – Predicted chemical shifts of possible anthoteibinene H (**3**) diastereomers

Isomer 1	7R-10R	Isomer 2	7R-10S	Experimental	
**Atom ID**	**Chemical shift** δH or δC	**Atom ID**	**Chemical shift** δH or δC	**Atom ID**	**Chemical shift** δH or δC
C1	41.584	C1	41.613	10	37.4
C2	22.789	C2	25.337	9	24.8
C3	24.443	C3	25.458	8	21.2
C4	42.858	C4	45.888	7	43.3
C5	142.42	C5	142.925	6	141.7
C6	126.256	C6	126.832	5	120.4
C7	123.281	C7	124.133	4	120.8
C8	143.517	C8	143.778	3	135.9
C9	108.609	C9	108.646	2	113.7
C10	155.564	C10	156.135	1	153.4
C12	181.351	C12	181.157	14	177.8
C14	21.451	C14	21.593	15	21.2
C16	36.665	C15	35.252	11	30.7
C17	20.543	C16	19.148	12	21.7
C18	14.932	C17	14.599	13	18
H15	3.545	H18	3.474	H-10	4.05
H19	1.455	H19	2.385	H-9b	2.14
H20	2.251	H20	1.439	H-9a	1.74
H21	1.974	H21	1.774	H-8b	1.56
H22	1.861	H22	2.027	H-8a	1.94
H23	2.902	H23	2.899	H-7	2.64
H24	7.083	H24	7.039	H-5	6.6
H25	6.947	H25	6.921	H-3	6.17
H26	2.519	H26	2.499	H-15	2.13
H27	2.519	H27	2.499	H-15	2.13
H28	2.519	H28	2.499	H-15	2.13
H29	2.364	H29	2.419	H-11	2.21
H30	1.271	H30	1.207	H-12	1.02
H31	1.271	H31	1.207	H-12	1.02
H32	1.271	H32	1.207	H-12	1.02
H33	0.718	H33	0.997	H-13	0.76
H34	0.718	H34	0.997	H-13	0.76
H35	0.718	H35	0.997	H-13	0.76

Table S17 – DP4+ probabilities of anthoteibinene H (**3**) diastereomers

Settings	Type of data (shifts)
Default	Unscaled shifts

Functional	Solvent?	Basis Set
B3LYP	PCM	6-311G(d,p)

TMS 1H	31.913	TMS 13C	184.350
Default	μ	σ	ν
13Cu,sp2	7.809	2.409	8.781
13Cu,sp3	5.678	2.271	17.407
1Hu,sp2	0.206	0.124	5.987
1Hu,sp3	-0.022	0.112	2.756
13Cs	-	1.738	6.366
1Hs	-	0.095	2.850

Isomer Nº			1	2	3	4	5	6	7	8
DP4+ (%)		H data	0.00%	100.00%	-	-	-	-	-	-
		C data	0.47%	99.53%	-	-	-	-	-	-
		All data	0.00%	100.00%	-	-	-	-	-	-
**Type**	**sp2?**	**Exp**	**1**	**2**	**3**	**4**	**5**	**6**	**7**	**8**
c		37.4	41.584	41.613						
c		24.8	22.789	25.337						
c		21.2	24.443	25.458						
c		43.3	42.858	45.888						
c	x	141.7	142.42	142.925						
c	x	120.4	126.256	126.832						
c	x	120.8	123.281	124.133						
c	x	135.9	143.517	143.778						
c	x	113.7	108.609	108.646						
c	x	153.4	155.564	156.135						
c	x	177.8	181.351	181.157						
c		21.2	21.451	21.593						
c		30.7	36.665	35.252						
c		21.7	20.543	19.148						
c		18	14.932	14.599						
h		4.05	3.545	3.474						
h		2.14	1.455	2.385						
h		1.74	2.251	1.439						
h		1.56	1.974	1.774						
h		1.94	1.861	2.027						
h		2.64	2.902	2.899						
h	x	6.6	7.083	7.039						
h	x	6.17	6.947	6.921						
h		2.13	2.519	2.499						
h		2.13	2.519	2.499						
h		2.13	2.519	2.499						
h		2.21	2.364	2.419						
h		1.02	1.271	1.207						
h		1.02	1.271	1.207						
h		1.02	1.271	1.207						
h		0.76	0.718	0.997						
h		0.76	0.718	0.997						
h		0.76	0.718	0.997						

Default parameters	1	2
sDP4+ (H data)	0.00%	100.00%
sDP4+ (C data)	42.96%	57.04%
sDP4+ (all data)	0.00%	100.00%
uDP4+ (H data)	60.39%	39.61%
uDP4+ (C data)	0.63%	99.37%
uDP4+ (all data)	0.96%	99.04%
DP4+ (H data)	0.00%	100.00%
DP4+ (C data)	0.47%	99.53%
DP4+ (all data)	0.00%	100.00%

Table S18 – Predicted chemical shifts of possible anthoteibinene O (**10**) diastereomers

Isomer 1	7R-10R	Isomer 2	7R-10S	Experimental	
**Atom ID**	**Chemical shift** δ_H or δ_C	**Atom ID**	**Chemical shift** δ_H or δ_C	**Atom ID**	**Chemical shift** δ_H or δ_C
C1	156.11	C1	157.944	C-2	153.8
C2	121.45	C2	119.849	C-1	118.6
C3	148.16	C3	147.536	C-6	141.8
C4	123.79	C4	125.199	C-5	121.8
C5	142.39	C5	141.772	C-4	136.9
C6	111.55	C6	115.008	C-3	113.9
C7	20.02	C7	19.947	C-10	21.3
C9	39.73	C9	39.065	C-9	40.2
C10	24.85	C10	23.168	C-9	24.1
C11	18.23	C11	20.047	C-7	20.8
C12	44.71	C12	44.63	C-11	42.8
C13	36.06	C13	38.834	C-12	31.6
C14	19.49	C14	20.707	C-13	21.8
C15	12.86	C15	17.005	C-14	18.5
C16	176.45	C16	177.317	C-15	181.7
H19	7.11	H19	6.903	H-6	6.64
H20	6.61	H20	6.757	H-3	6.43
H21	2.423	H21	2.408	H-15	2.21
H22	2.423	H22	2.408	H-15	2.21
H23	2.423	H23	2.408	H-15	2.21
H25	3.86	H25	3.926	H-10	3.77
H26	2.3	H26	1.814	H-9b	2.11
H27	1.49	H27	2.17	H-9a	1.94
H28	2.2	H28	1.942	H-8b	1.66
H29	1.7	H29	2.324	H-8a	1.89
H30	2.93	H30	2.667	H-7	2.56
H31	2.47	H31	2.129	H-11	2.14
H32	1.207	H32	1.211	H-12	1.01
H33	1.207	H33	1.211	H-12	1.01
H34	1.207	H34	1.211	H-12	1.01
H35	0.68	H35	0.898	H-13	0.76
H36	0.68	H36	0.898	H-13	0.76
H37	0.68	H37	0.898	H-13	0.76

Table S19 – DP4+ probabilities of anthoteibinene O (**10**) diastereomers

Settings	Type of data (shifts)
Default	Unscaled shifts

Functional	Solvent?	Basis Set
B3LYP	PCM	6-311G(d,p)

TMS 1H	31.913	TMS 13C	184.350
Default	μ	σ	ν
13Cu,sp2	7.809	2.409	8.781
13Cu,sp3	5.678	2.271	17.407
1Hu,sp2	0.206	0.124	5.987
1Hu,sp3	-0.022	0.112	2.756
13Cs	-	1.738	6.366
1Hs	-	0.095	2.850

Isomer Nº		1	2	3	4	5	6	7	8
DP4+ (%)	H data	0.00%	100.00%	-	-	-	-	-	-
	C data	0.00%	100.00%	-	-	-	-	-	-
	All data	0.00%	100.00%	-	-	-	-	-	-

Type	sp2?	Exp	1	2	3	4	5	6	7	8
c	x	153.8	156.11	157.944						
c	x	118.6	121.45	119.849						
c	x	141.8	148.16	147.536						
c	x	121.8	123.79	125.199						
c	x	136.9	142.39	141.772						
c	x	113.9	111.55	115.008						
c		21.3	20.02	19.947						
c		40.2	39.73	39.065						
c		24.1	24.85	23.168						
c		20.8	18.23	20.047						
c		42.8	44.71	44.63						
c		31.6	36.06	38.834						
c		21.8	19.49	20.707						
c		18.5	12.86	17.005						
c	x	181.7	176.45	177.317						
h	x	6.64	7.11	6.903						
h	x	6.43	6.61	6.757						
h		2.21	2.423	2.408						
h		2.21	2.423	2.408						
h		2.21	2.423	2.408						
h		3.77	3.86	3.926						
h		2.11	2.3	1.814						
h		1.94	1.49	2.17						
h		1.66	2.2	1.942						
h		1.89	1.7	2.324						
h		2.56	2.93	2.667						
h		2.14	2.47	2.129						
h		1.01	1.207	1.211						
h		1.01	1.207	1.211						
h		1.01	1.207	1.211						
h		0.76	0.68	0.898						
h		0.76	0.68	0.898						
h		0.76	0.68	0.898						

Default parameters	1	2
sDP4+ (H data)	0.00%	100.00%
sDP4+ (C data)	1.33%	98.67%
sDP4+ (all data)	0.00%	100.00%
uDP4+ (H data)	7.54%	92.46%
uDP4+ (C data)	0.07%	99.93%
uDP4+ (all data)	0.01%	99.99%
DP4+ (H data)	0.00%	100.00%
DP4+ (C data)	0.00%	100.00%
DP4+ (all data)	0.00%	100.00%

Table S20 – Predicted conformers of anthoteibinene F (**1**), with calculated properties for ECD spectra predictions

Compound	G (Hartree)	G (kcal/mol)	ΔG(kcal/mol)	Boltzmann Dist (%)	Num Negative Frequencies
Anthoteibinene F-a	-809.1039	-507720.84	-2.74	0.6	0
Anthoteibinene F-b	-809.1084	-507723.58	0.00	65.7	0
Anthoteibinene F-c	-809.1075	-507723.10	-0.48	29.5	0
Anthoteibinene F-d	-809.1050	-507721.53	-2.05	2.1	0
Anthoteibinene F-e	-809.1010	-507718.98	-4.60	0	0
Anthoteibinene F-f	-809.1042	-507721.02	-2.56	0.9	0
Anthoteibinene F-g	-809.1037	-507720.71	-2.87	0.5	0
Anthoteibinene F-h	-809.1036	-507720.64	-2.94	0.5	0
Anthoteibinene F-i	-809.1030	-507720.27	-3.31	0.2	0

Title: Anthoteibinene_F-a

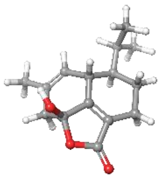

Title: Anthoteibinene_F-b

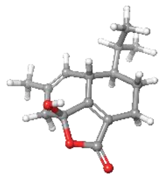

Title: Anthoteibinene_F-c

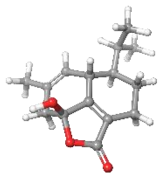

Title: Anthoteibinene_F-d

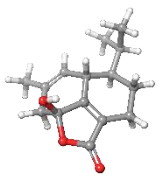

Title: Anthoteibinene_F-e

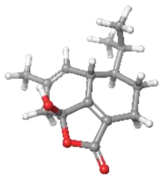

Title: Anthoteibinene_F-f

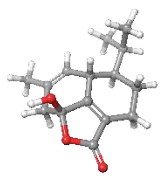

Title: Anthoteibinene_F-g

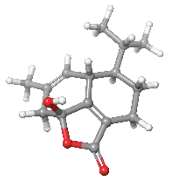

Title: Anthoteibinene_F-h

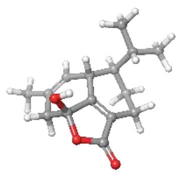

Title: Anthoteibinene_F-j

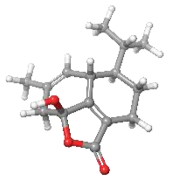

Table S21 – Predicted conformers of anthoteibinene G (**2**), with calculated properties for ECD spectra predictions

Compound	G (Hartree)	G (kcal/mol)	ΔG(kcal/mol)	Boltzmann Dist (%)	Num Negative Frequencies
Anthoteibinene G-a	-848.39	-532371.00	0.00	93.48	0
Anthoteibinene G-b	-848.38	-532368.45	-2.55	1.27	1
Anthoteibinene G-c	-848.38	-532368.87	-2.13	2.57	0
Anthoteibinene G-d	-848.38	-532368.20	-2.80	0.83	0
Anthoteibinene G-e	-848.38	-532368.39	-2.61	1.15	0
Anthoteibinene G-f	-848.38	-532366.50	-4.50	0.05	1
Anthoteibinene G-g	-848.38	-532367.42	-3.58	0.22	0
Anthoteibinene G-h	-848.38	-532367.29	-3.71	0.18	0
Anthoteibinene G-i	-848.38	-532367.48	-3.52	0.25	0

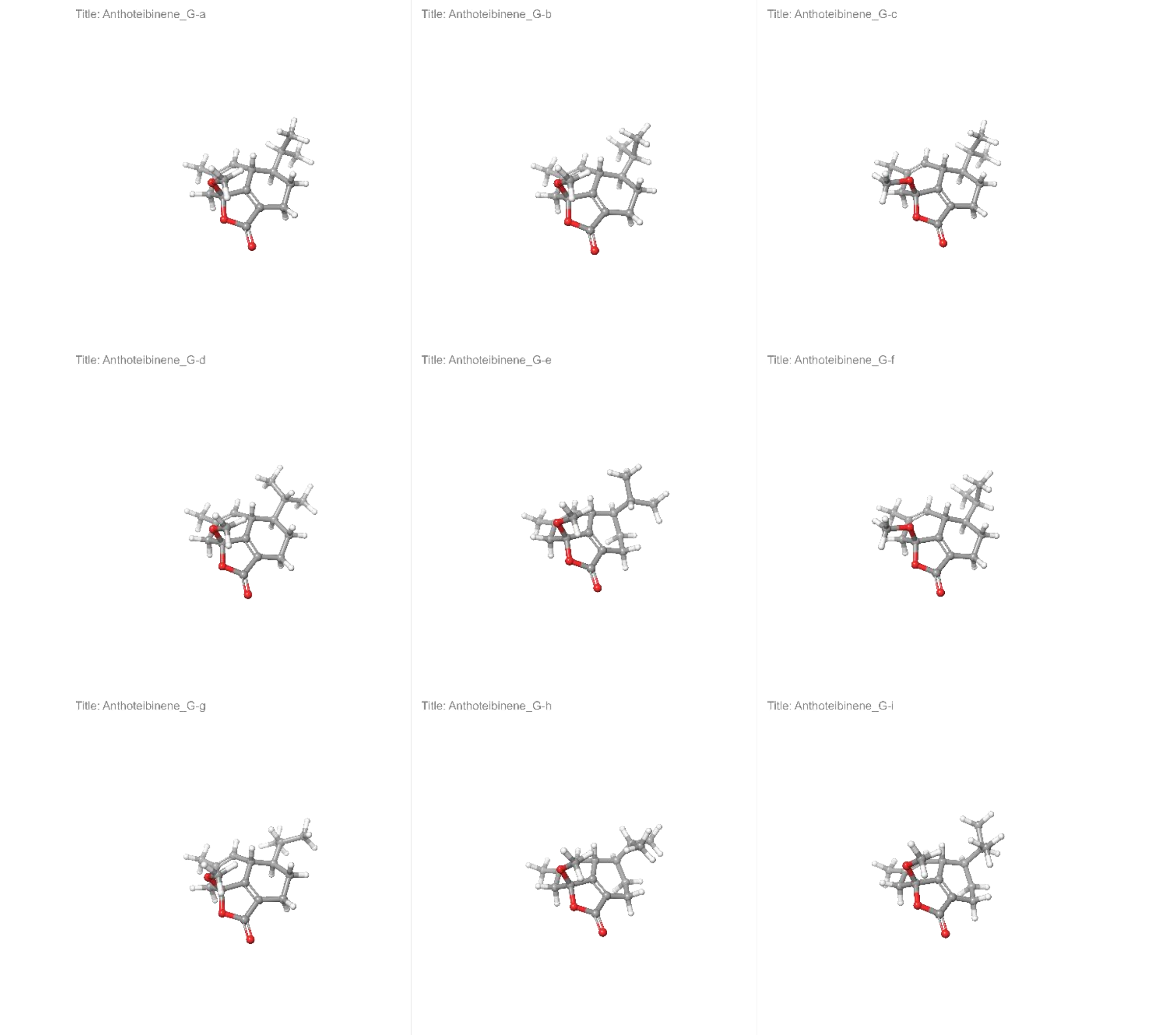

Table S22 – Predicted conformers of anthoteibinene H (**3**), with calculated properties for ECD spectra predictions

Compound	G (Hartree)	G (kcal/mol)	ΔG(kcal/mol)	Boltzmann Dist (%)	Num Negative Frequencies
Anthoteibinene H-a	-732.71	-459783.24	0.00	71.95	0
Anthoteibinene H-b	-732.71	-459782.33	-0.91	15.36	0
Anthoteibinene H-c	-732.71	-459781.99	-1.25	8.70	0
Anthoteibinene H-d	-732.71	-459781.50	-1.74	3.81	0
Anthoteibinene H-e	-732.70	-459779.67	-3.57	0.17	0

Title: Anthoteibinene_H-a

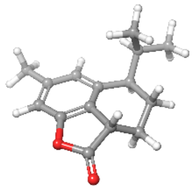

Title: Anthoteibinene_H-b

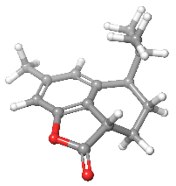

Title: Anthoteibinene_H-c

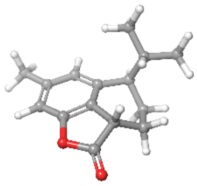

Title: Anthoteibinene_H-d

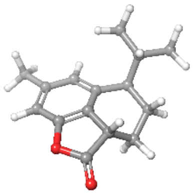

Title: Anthoteibinene_H-e

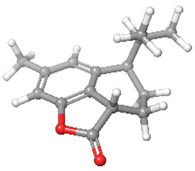

Table S23 – Predicted conformers of anthoteibinene I (**4**), with calculated properties for ECD spectra predictions

Compound	G (Hartree)	G (kcal/mol)	ΔG(kcal/mol)	Boltzmann Dist (%)	Num Negative Frequencies
Anthoteibinene I-a	-732.86	-459876.62	-0.45	17.87	0
Anthoteibinene I-b	-732.86	-459876.73	-0.34	21.59	0
Anthoteibinene I-c	-732.86	-459877.07	0.00	38.32	0
Anthoteibinene I-d	-732.86	-459876.45	-0.62	13.47	0
Anthoteibinene I-e	-732.86	-459875.48	-1.59	2.63	0
Anthoteibinene I-f	-732.86	-459875.88	-1.19	5.13	0
Anthoteibinene I-g	-732.86	-459874.84	-2.23	0.89	0
Anthoteibinene I-h	-732.85	-459873.50	-3.57	0.09	1

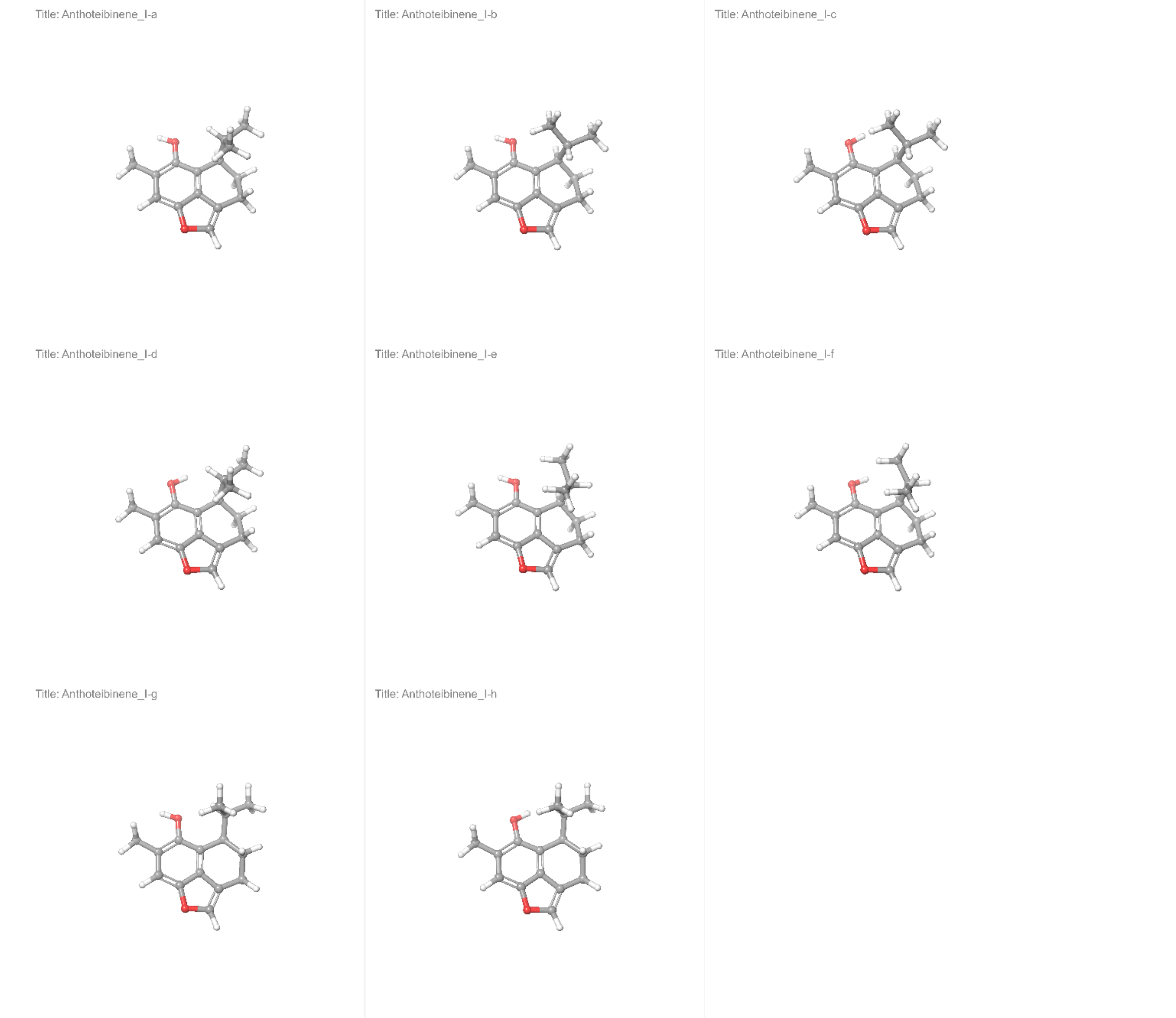

Table S23 – Predicted conformers of anthoteibinene J (**5**), with calculated properties for ECD spectra predictions

Compound	G (Hartree)	G (kcal/mol)	ΔG(kcal/mol)	Boltzmann Dist (%)	Num Negative Frequencies
Anthoteibinene_J-a	-732.856949	-459875.06	-0.03	48.7	0
Anthoteibinene_J-b	-732.856997	-459875.09	0.00	51.3	0

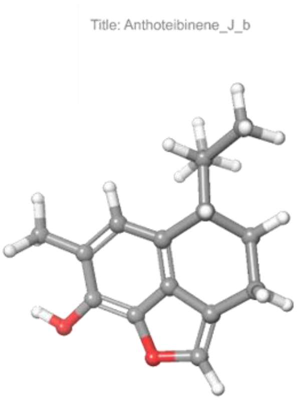

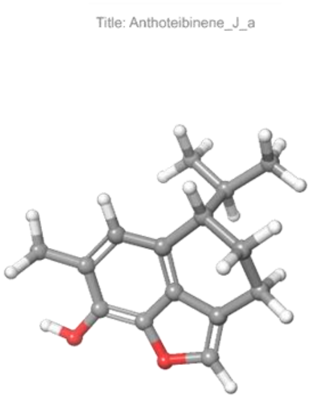

Table S25 – Predicted conformers of anthoteibinene K (**6**), with calculated properties for ECD spectra predictions

Compound	G (Hartree)	G (kcal/mol)	ΔG(kcal/mol)	Boltzmann Dist (%)	Num Negative Frequencies
Anthoteibinene K-a	-657.46	-412565.23	0.00	41.42	0
Anthoteibinene K-b	-657.46	-412565.17	-0.07	37.11	0
Anthoteibinene K-c	-657.46	-412564.46	-0.78	11.13	0
Anthoteibinene K-d	-657.46	-412564.22	-1.01	7.52	0
Anthoteibinene K-e	-657.46	-412563.55	-1.69	2.39	0
Anthoteibinene K-f	-657.46	-412562.53	-2.70	0.43	0

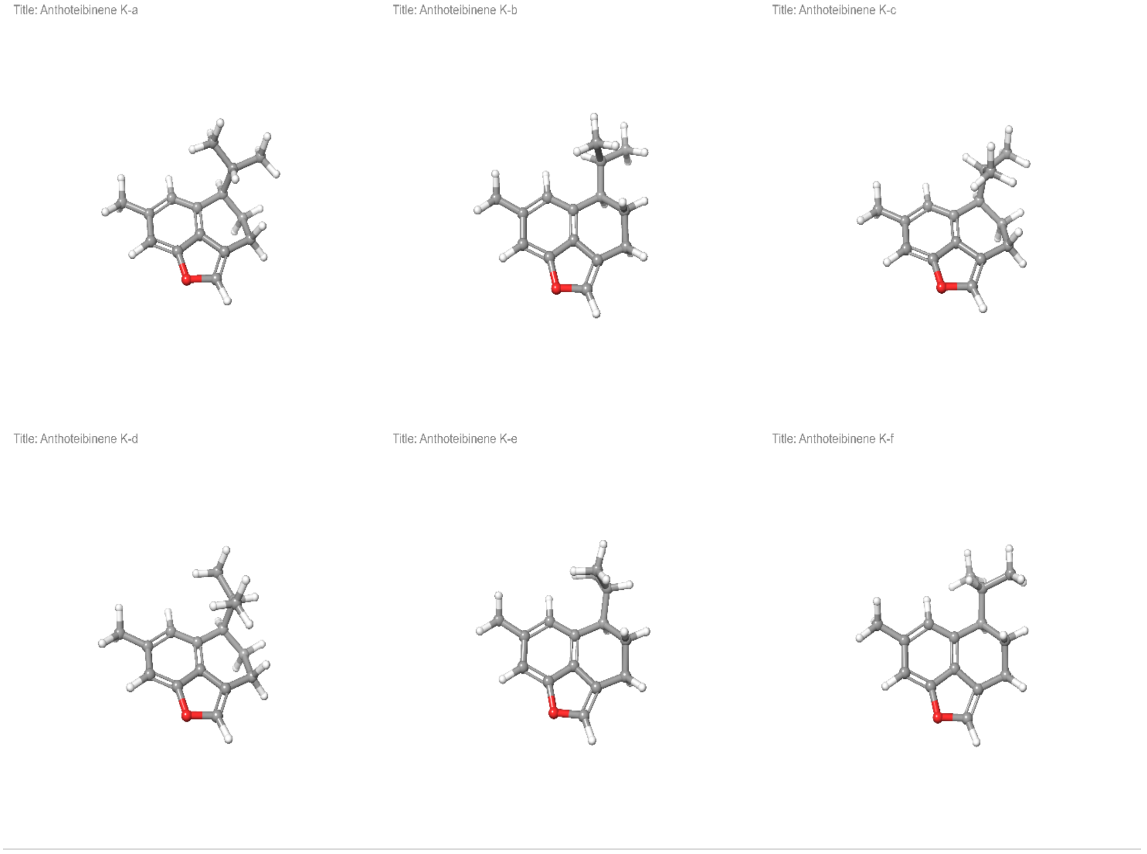

Table S26 – Predicted conformers of anthoteibinene L (**7**), with calculated properties for ECD spectra predictions

Compound	G (Hartree)	G (kcal/mol)	ΔG(kcal/mol)	Boltzmann Dist (%)	Num Negative Frequencies
Anthoteibinene L-a	-1268.69	-796114.69	0.00	73.37	0
Anthoteibinene L-b	-1268.69	-796113.53	-1.16	10.39	0
Anthoteibinene L-c	-1268.69	-796113.56	-1.14	10.78	0
Anthoteibinene L-d	-1268.69	-796112.87	-1.82	3.41	0
Anthoteibinene L-e	-1268.68	-796112.20	-2.49	1.09	0
Anthoteibinene L-f	-1268.68	-796111.47	-3.22	0.32	0
Anthoteibinene L-g	-1268.68	-796111.82	-2.88	0.57	0
Anthoteibinene L-h	-1268.68	-796110.61	-4.08	0.08	0

Title: Anthoteibinene_L-a

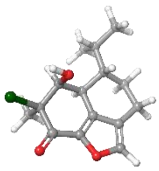

Title: Anthoteibinene_L-b

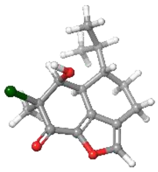

Title: Anthoteibinene_L-c

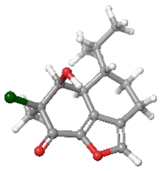

Title: Anthoteibinene_L-d

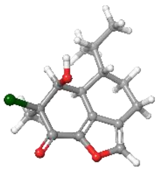

Title: Anthoteibinene_L-e

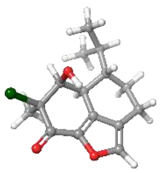

Title: Anthoteibinene_L-f

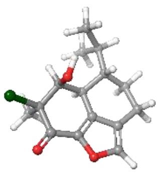

Title: Anthoteibinene_L-g

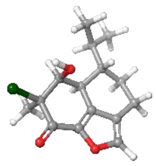

Title: Anthoteibinene_L-h

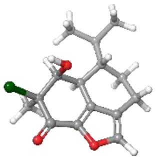

Table S27 – Predicted conformers of anthoteibinene M (**8**), with calculated properties for ECD spectra predictions

Compound	G (Hartree)	G (kcal/mol)	ΔG(kcal/mol)	Boltzmann Dist (%)	Num Negative Frequencies
Anthoteibinene M-a	-809.10	-507717.19	-2.19	1.70	1
Anthoteibinene M-b	-809.10	-507718.12	-1.25	8.10	0
Anthoteibinene M-c	-809.10	-507718.05	-1.33	7.10	0
Anthoteibinene M-d	-809.10	-507715.39	-3.98	0.10	0
Anthoteibinene M-e	-809.10	-507719.38	0.00	66.90	0
Anthoteibinene M-f	-809.10	-507717.23	-2.14	1.80	0
Anthoteibinene M-g	-809.10	-507717.45	-1.92	2.60	1
Anthoteibinene M-h	-809.10	-507717.69	-1.68	3.90	0
Anthoteibinene M-i	-809.10	-507715.72	-3.66	0.10	0
Anthoteibinene M-j	-809.10	-507715.80	-3.58	0.20	0
Anthoteibinene M-k	-809.10	-507717.96	-1.42	6.10	0
Anthoteibinene M-l	-809.10	-507717.07	-2.31	1.40	0

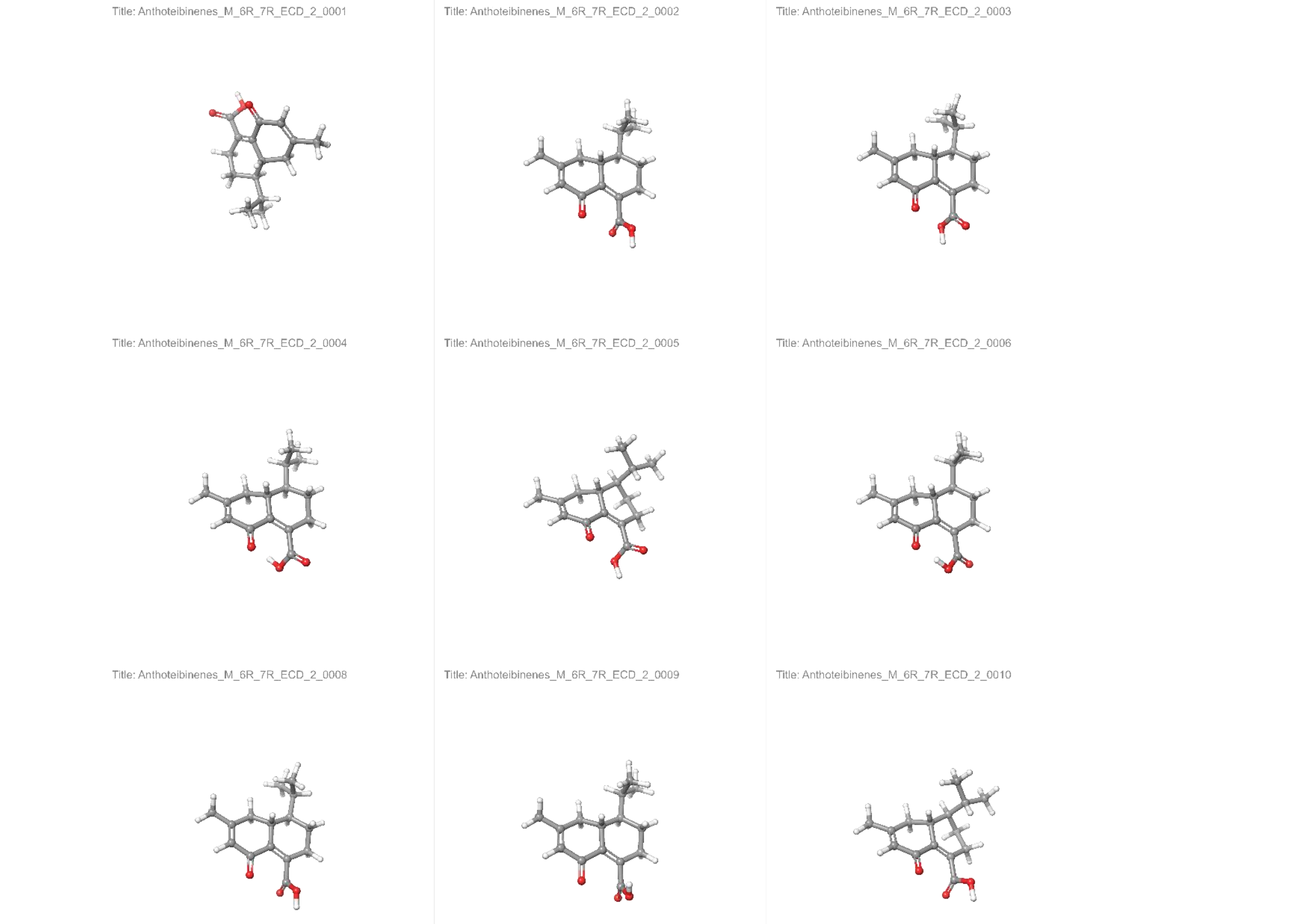

Table S28 – Predicted conformers of anthoteibinene N (**9**), with calculated properties for ECD spectra predictions

Compound	G (Hartree)	G (kcal/mol)	ΔG(kcal/mol)	Boltzmann Dist (%)	Num Negative Frequencies
Anthoteibinene N-a	-809.12	-507728.20	-0.21	34.54	0
Anthoteibinene N-b	-809.12	-507728.41	0.00	49.06	0
Anthoteibinene N-c	-809.11	-507725.77	-2.63	0.58	0
Anthoteibinene N-d	-809.11	-507725.12	-3.29	0.19	0
Anthoteibinene N-e	-809.11	-507725.09	-3.31	0.18	0
Anthoteibinene N-f	-809.11	-507727.50	-0.91	10.63	0
Anthoteibinene N-g	-809.11	-507725.27	-3.14	0.24	0
Anthoteibinene N-h	-809.11	-507726.50	-1.90	1.98	0
Anthoteibinene N-i	-809.11	-507726.67	-1.74	2.61	0

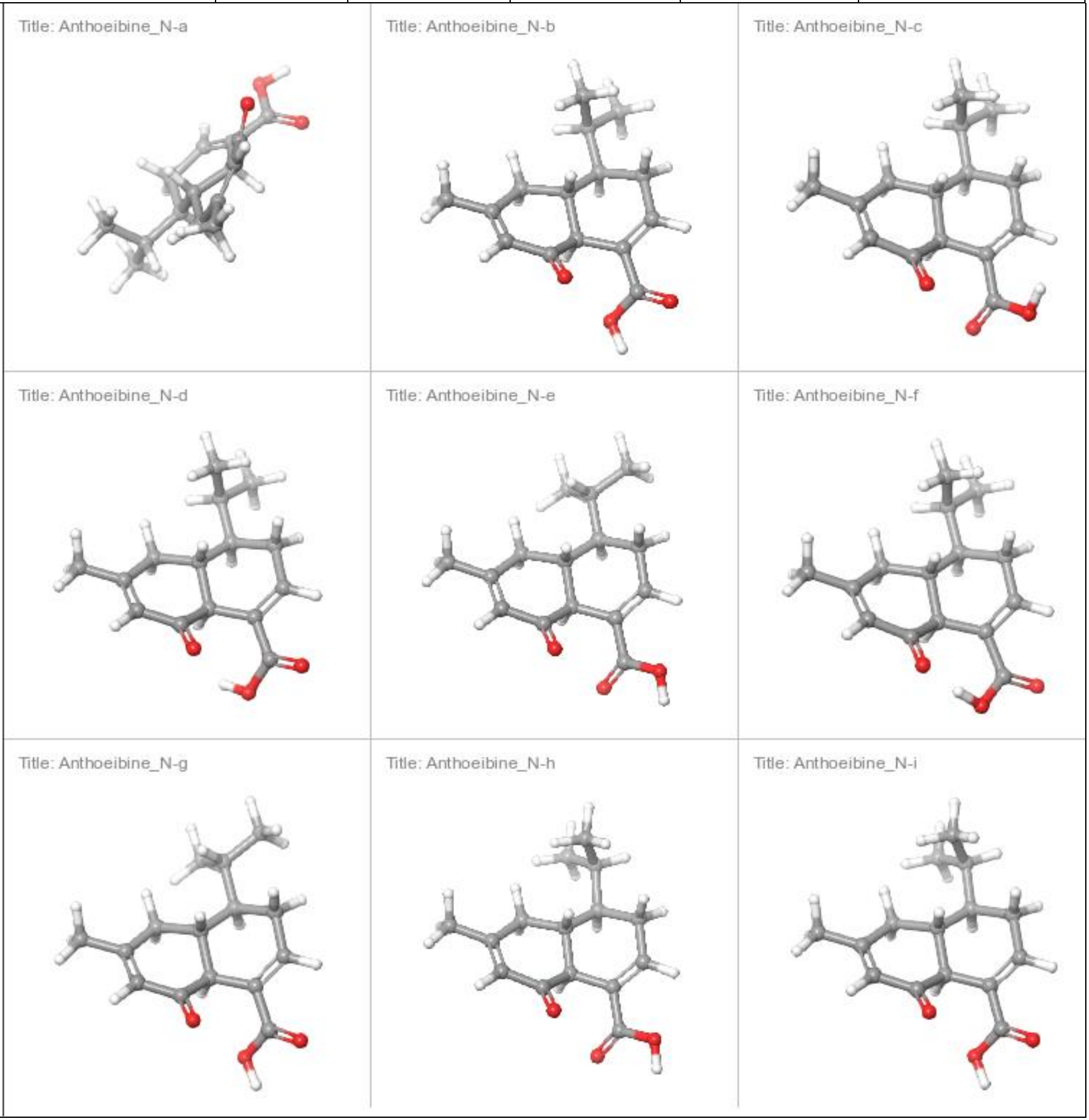

Table S29 – Predicted conformers of anthoteibinene O (**10**), with calculated properties for ECD spectra predictions

Compound	G (Hartree)	G (kcal/mol)	ΔG(kcal/mol)	Boltzmann Dist (%)	Num Negative Frequencies
Anthoteibinene O-a	-809.14	-507744.85	0.00	33.89	0
Anthoteibinene O-b	-809.14	-507743.88	-0.97	6.58	0
Anthoteibinene O-c	-809.14	-507743.33	-1.52	2.61	0
Anthoteibinene O-d	-809.14	-507742.69	-2.15	0.89	0
Anthoteibinene O-e	-809.14	-507744.43	-0.42	16.82	0
Anthoteibinene O-f	-809.14	-507742.55	-2.30	0.70	0
Anthoteibinene O-g	-809.14	-507743.38	-1.47	2.86	0
Anthoteibinene O-h	-809.14	-507743.96	-0.89	7.58	0
Anthoteibinene O-i	-809.14	-507744.52	-0.33	19.45	0
Anthoteibinene O-j	-809.14	-507742.62	-2.23	0.78	0
Anthoteibinene O-k	-809.14	-507743.98	-0.87	7.84	0
Anthoteibinene O-l	-809.13	-507740.26	-4.59	0.01	0

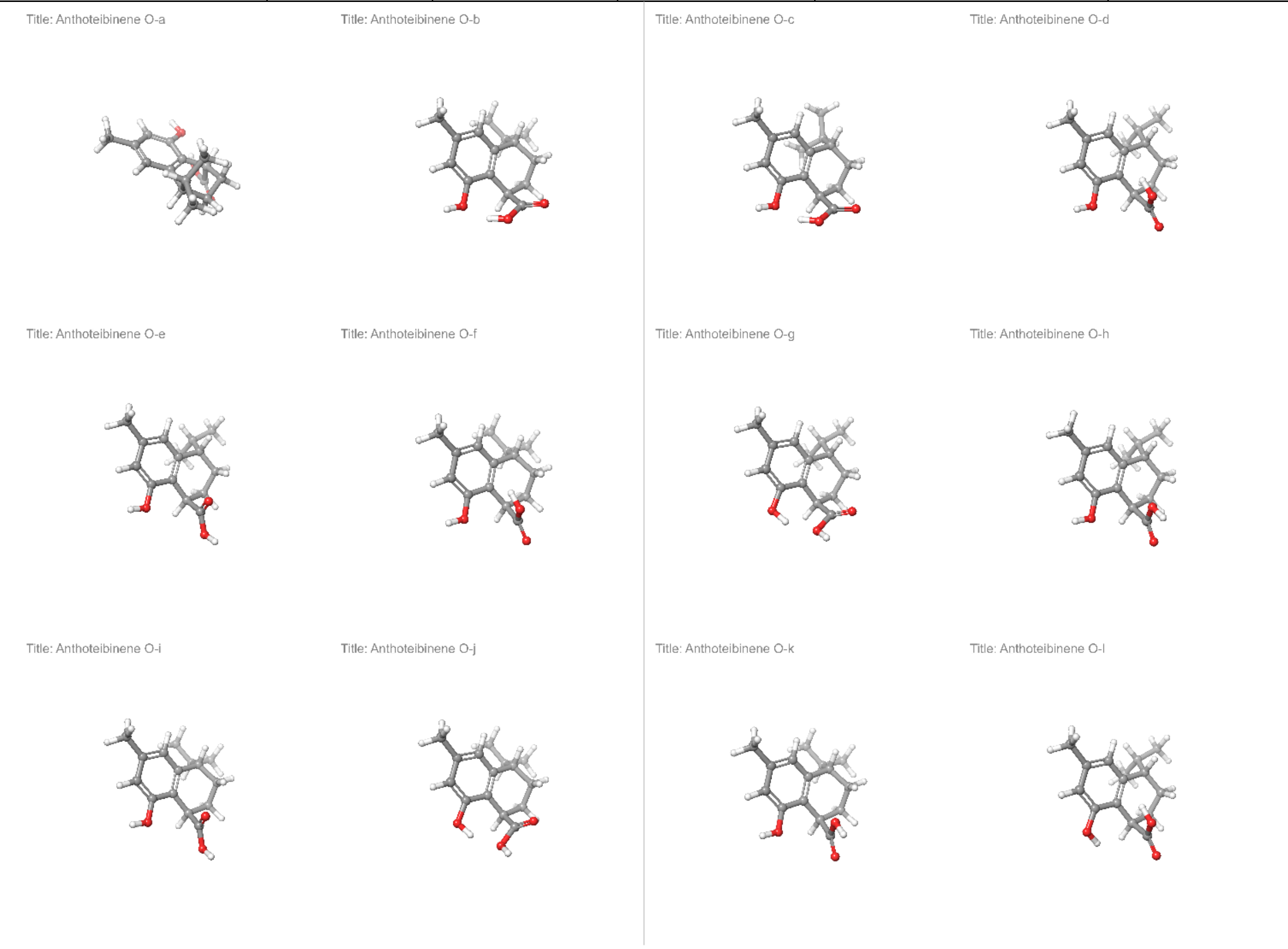

Table S30 – Predicted conformers of anthoteibinene P (**11**), with calculated properties for ECD spectra predictions

Compound	G (Hartree)	G (kcal/mol)	ΔG(kcal/mol)	Boltzmann Dist (%)	Num Negative Frequencies
Anthoteibinene P-a	-774.34	-485906.01	-0.39	25.73	0
Anthoteibinene P-b	-774.34	-485906.40	0.00	49.48	0
Anthoteibinene P-c	-774.34	-485905.86	-0.54	19.89	0
Anthoteibinene P-d	-774.34	-485905.03	-1.37	4.89	0

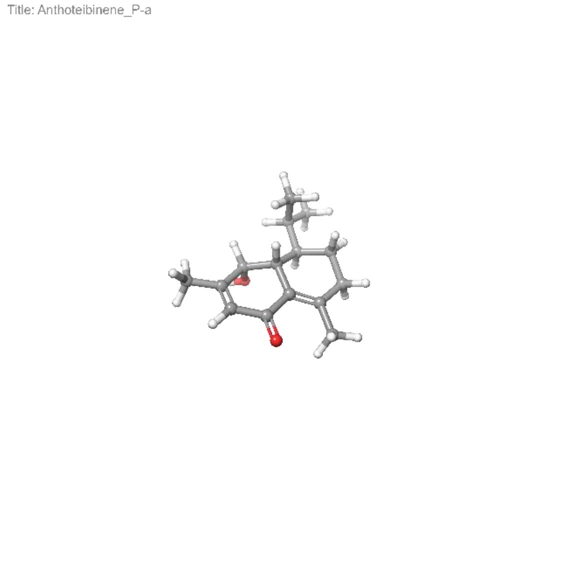

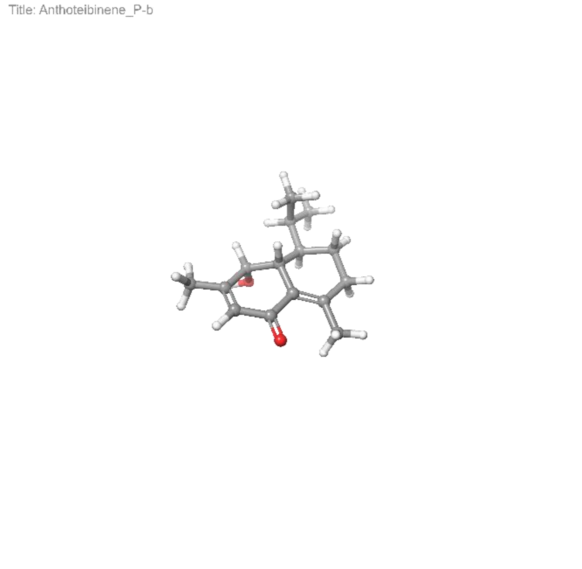

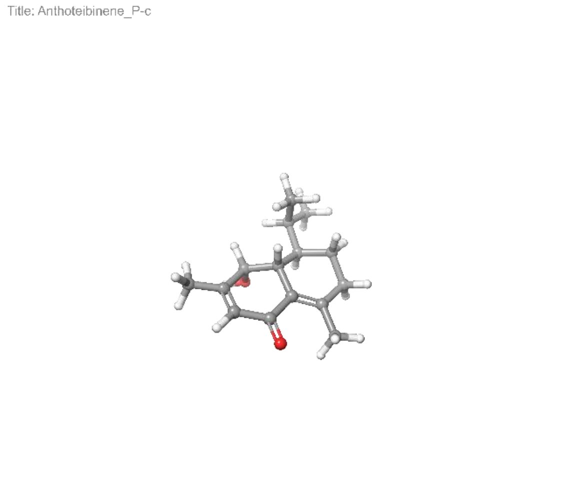

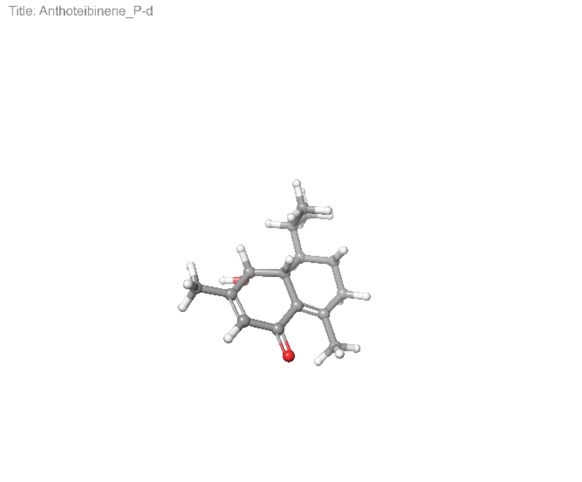

Table S31 – Predicted conformers of anthoteibinene Q (**12**), with calculated properties for ECD spectra predictions

Compound	G (Hartree)	G (kcal/mol)	ΔG(kcal/mol)	Boltzmann Dist (%)	Num Negative Frequencies
Anthoteibinene_Q-a	-769.84	-483082.75	-0.96	9.26	0
Anthoteibinene_Q-b	-769.84	-483083.71	0.00	47.00	0
Anthoteibinene_Q-c	-769.84	-483082.39	-1.32	5.03	0
Anthoteibinene_Q-d	-769.84	-483083.54	-0.17	35.28	0
Anthoteibinene_Q-e	-769.84	-483081.71	-2.00	1.60	0
Anthoteibinene_Q-f	-769.84	-483080.69	-3.02	0.29	0
Anthoteibinene_Q-g	-769.84	-483081.61	-2.11	1.34	0
Anthoteibinene_Q-h	-769.84	-483080.45	-3.26	0.19	0

Title: Anthoteibinene_Q-a

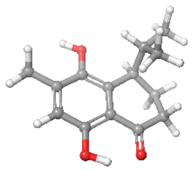

Title: Anthoteibinene_Q-b

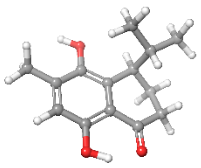

Title: Anthoteibinene_Q-c

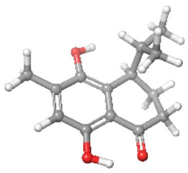

Title: Anthoteibinene_Q-d

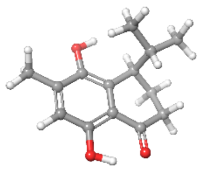

Title: Anthoteibinene_Q-e

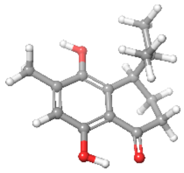

Title: Anthoteibinene_Q-f

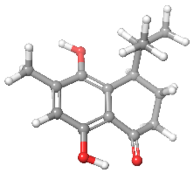

Title: Anthoteibinene_Q-g

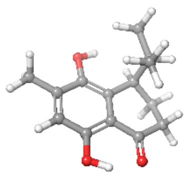

Title: Anthoteibinene_Q-h

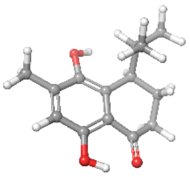

Table S32 – Predicted conformers properties of Anthoteibinene F (**1**), for NMR chemical shift calculations

Compound	Chiral Centers	G (Hartree)	G(kcal/mol)	ΔG(kcal/mol)	Boltzmann Dist (%)
Isomer 1					
Anthoteibinene F-1-a	2S-6S-7R	-809.29	-507834.87	-1.20	7.7
Anthoteibinene F-1-b	2S-6S-7R	-809.29	-507836.07	0.00	58.9
Anthoteibinene F-1-c	2S-6S-7R	-809.28	-507832.24	-3.83	0.1
Anthoteibinene F-1-d	2S-6S-7R	-809.28	-507833.59	-2.49	0.9
Anthoteibinene F-1-e	2S-6S-7R	-809.28	-507834.01	-2.06	1.8
Anthoteibinene F-1-f	2S-6S-7R	-809.29	-507835.53	-0.55	23.4
Anthoteibinene F-1-g	2S-6S-7R	-809.28	-507831.05	-5.02	0.0
Anthoteibinene F-1-h	2S-6S-7R	-809.29	-507834.82	-1.25	7.1
Isomer 2					
Anthoteibinene F-2-a	2R-6S-7R	-809.31	-507847.82	0.00	69.6
Anthoteibinene F-2-b	2R-6S-7R	-809.30	-507845.08	-2.74	0.7
Anthoteibinene F-2-c	2R-6S-7R	-809.31	-507847.23	-0.59	25.6
Anthoteibinene F-2-d	2R-6S-7R	-809.30	-507845.66	-2.15	1.8
Anthoteibinene F-2-e	2R-6S-7R	-809.30	-507842.89	-4.92	0.0
Anthoteibinene F-2-f	2R-6S-7R	-809.30	-507844.77	-3.05	0.4
Anthoteibinene F-2-g	2R-6S-7R	-809.30	-507845.20	-2.62	0.8
Anthoteibinene F-2-h	2R-6S-7R	-809.30	-507845.00	-2.81	0.6
Anthoteibinene F-2-i	2R-6S-7R	-809.30	-507841.72	-6.10	0.0
Anthoteibinene F-2-j	2R-6S-7R	-809.30	-507844.10	-3.72	0.1

Table S33 – Predicted conformers properties of Anthoteibinene H (**3**), for NMR chemical shift calculations

Compound	Chiral Centers	G (Hartree)	G(kcal/mol)	ΔG(kcal/mol)	Boltzmann Dist (%)
Isomer 1					
Anthoteibinene H-1-a	7R-10R	-732.85	-459869.67	0.00	82.3
Anthoteibinene H-1-b	7R-10R	-732.85	-459868.58	-1.09	13.1
Anthoteibinene H-1-c	7R-10R	-732.84	-459867.41	-2.27	1.8
Anthoteibinene H-1-d	7R-10R	-732.84	-459867.00	-2.67	0.9
Anthoteibinene H-1-e	7R-10R	-732.84	-459867.32	-2.36	1.5
Anthoteibinene H-1-f	7R-10R	-732.84	-459866.46	-3.22	0.4
Isomer 2					
Anthoteibinene H-2-a	7R-10S	-732.85	-459870.93	0.00	81.9
Anthoteibinene H-2-b	7R-10S	-732.85	-459869.56	-1.37	8.1
Anthoteibinene H-2-c	7R-10S	-732.85	-459869.37	-1.56	5.9
Anthoteibinene H-2-d	7R-10S	-732.85	-459869.13	-1.80	3.9
Anthoteibinene H-2-e	7R-10S	-732.84	-459867.00	-3.93	0.1
Anthoteibinene H-2-f	7R-10S	-732.84	-459866.47	-4.46	0.0

Table S34 – Predicted conformers properties of Anthoteibinene O (**10**), for NMR chemical shift calculations

Compound	Chiral Centers	G (Hartree)	G(kcal/mol)	ΔG(kcal/mol)	Boltzmann Dist (%)
Isomer 1					

Anthoteibinene O-1-a	7R-10S	-809.30	-507844.10	0.00	37.6
Anthoteibinene O-1-b	7R-10S	-809.30	-507842.93	-1.17	5.2
Anthoteibinene O-1-c	7R-10S	-809.30	-507842.09	-2.00	1.3
Anthoteibinene O-1-d	7R-10S	-809.30	-507842.75	-1.34	3.9
Anthoteibinene O-1-e	7R-10S	-809.30	-507843.96	-0.13	30.1
Anthoteibinene O-1-f	7R-10S	-809.30	-507842.81	-1.29	4.3
Anthoteibinene O-1-g	7R-10S	-809.30	-507843.59	-0.50	16.0
Anthoteibinene O-1-h	7R-10S	-809.30	-507842.23	-1.86	1.6
Isomer 2					
Anthoteibinene O-2-a	7R-10R	-809.30	-507845.24	-0.83	14.2
Anthoteibinene O-2-b	7R-10R	-809.30	-507844.22	-1.86	2.5
Anthoteibinene O-2-c	7R-10R	-809.30	-507843.07	-3.00	0.4
Anthoteibinene O-2-d	7R-10R	-809.30	-507845.41	-0.66	18.9
Anthoteibinene O-2-e	7R-10R	-809.30	-507843.27	-2.81	0.5
Anthoteibinene O-2-f	7R-10R	-809.30	-507846.08	0.00	57.8
Anthoteibinene O-2-g	7R-10R	-809.30	-507844.70	-1.37	5.7
Anthoteibinene O-2-h	7R-10R	-809.30	-507840.87	-5.20	0.0
Anthoteibinene O-2-i	7R-10R	-809.30	-507842.06	-4.02	0.1
Anthoteibinene O-2-j	7R-10R	-809.30	-507840.75	-5.32	0.0
